# Lignocellulosic Films: Preparation, Properties, and Applications

**DOI:** 10.1021/acs.chemrev.5c00267

**Published:** 2025-12-10

**Authors:** Haishun Du, Kun Liu, Ting Xu, Chao Xu, Minsheng Lin, Zhiqiang Fang, Sang-Woo Kim, Ji-Young Seo, Jiansong Chen, Hongyang Ma, Benjamin S. Hsiao, Lisa Wasko DeVetter, Zhengyin Piao, Chuanling Si, Chaoji Chen, Qiang Yang, Sang-Young Lee, Yuan Yao, Xuejun Pan

**Affiliations:** † Department of Biological Systems Engineering, 5228 University of Wisconsin-Madison, Madison, Wisconsin 53706, United States; ‡ State Key Laboratory of Bio-Based Fiber Materials, 66345 Tianjin University of Science and Technology, Tianjin 300457, China; § Hubei Biomass-Resource Chemistry and Environmental Biotechnology Key Laboratory, Hubei Provincial Engineering Research Center of Emerging Functional Coating Materials, School of Resource and Environmental Sciences, Wuhan University, Wuhan 430079, China; ∥ School of Packaging, 3078 Michigan State University, East Lansing, Michigan 48824, United States; ⊥ State Key Laboratory of Advanced Papermaking and Paper-Based Materials, School of Light Industry and Engineering, 26467 South China University of Technology, Wushan Road 381, Guangzhou 510640, China; # Department of Chemical and Biomolecular Engineering, 26721 Yonsei University, Seoul 03722, Korea; ∇ Department of Battery Engineering, Yonsei University, Seoul 03722, Korea; ○ State Key Laboratory of Organic−Inorganic Composites, College of Materials Science and Engineering, Beijing University of Chemical Technology, Beijing 100029, China; ◆ Department of Chemistry, Stony Brook University, Stony Brook, New York 11794-3400, United States; ¶ Department of Horticulture, Washington State University, Northwestern Washington Research and Extension Center, Mount Vernon, Washington 98273, United States; □ Center for Industrial Ecology, Yale School of the Environment, Yale University, New Haven, Connecticut 06511, United States

## Abstract

Lignocellulosic films (LCFs) have garnered significant attention due to their unique combination of flexibility, functionality, cost-effectiveness, and eco-friendliness. Defined as thin, compact, and continuous sheets with a typical thickness in the range of 10–100 μm, LCFs have been used in various fields, including packaging, flexible electronics, energy storage and harvesting, sensing, water treatment, and agriculture. Based on preparation strategies and chemical compositions, LCFs can be categorized into cellulose derivative films, regenerated cellulose films, nanocellulose films, hemicellulose films, lignin-based films, and whole lignocellulosic biomass films. While previous reviews often focus on specific types of LCFs, e.g., nanocellulose films, a comprehensive review covering all categories and their recent advancements is still lacking. This review aims to address this gap by providing a thorough overview of the basic structure and chemistry of lignocellulosic biomass, preparation strategies, functionalization methods, and the broad spectrum of applications of LCFs. Additionally, it examines the environmental and economic feasibility of LCFs and identifies strategies to overcome existing challenges, offering valuable insights for advancing the field and supporting future innovation in sustainable material science.

## Graphical abstract

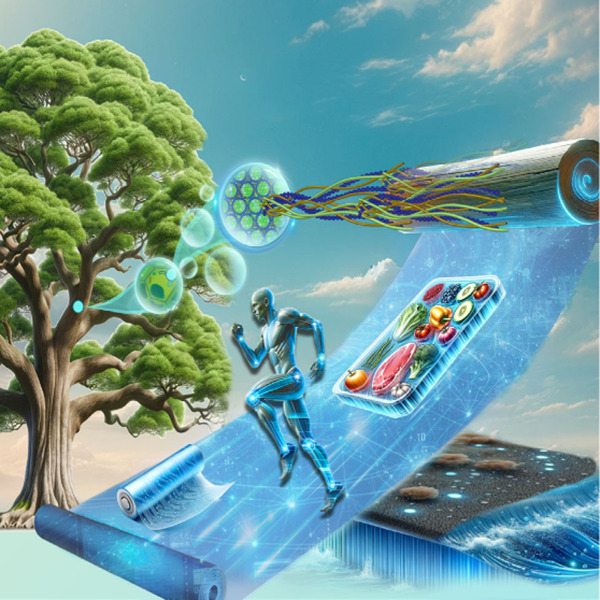

## 1 Introduction

The increasing environmental concerns and sustainability challenges associated with fossil-based products, such as synthetic polymer films used in packaging and agriculture, have emphasized the urgent need for eco-friendly alternatives.^1,2^ Synthetic plastics, while indispensable in modern life, contribute significantly to global pollution and waste management issues due to their nonbiodegradable nature and reliance on nonrenewable resources.^3^ These pressing concerns have catalyzed a global shift toward the development and adoption of materials derived from renewable and sustainable resources.^4^

Among these alternatives, lignocellulosic biomass has emerged as a highly promising candidate for future material production. With an estimated annual production of 200 billion metric tons, lignocellulosic biomass is one of the most abundant natural resources on Earth.^5^ Its three primary components, cellulose, hemicellulose, and lignin, offer unique structural and chemical properties,^6^ and therefore lignocellulosic biomass has been processed into a diverse array of functional materials (e.g., films) suitable for various applications.^7^ The inherent advantages of lignocellulosic biomass, such as renewability, biodegradability, nontoxicity, and cost-effectiveness, further underscore its potential to replace fossil-based materials across multiple industries.^8^

Over the past few decades, lignocellulosic films (LCFs) have garnered significant attention as versatile and eco-friendly materials, particularly within academia, as reflected in the rapid increase in related publications (Figure 1). LCFs are characterized by exceptional mechanical properties, tunable optical properties, low thermal expansion, excellent barrier performance, and strong environmental compatibility. These attributes enable LCFs to be utilized across a diverse range of applications, extending beyond traditional packaging to emerging fields such as flexible and wearable sensors, energy storage devices, and water purification technologies.^9–12^ The growing interest in LCFs highlights their potential as sustainable alternatives to conventional synthetic materials, aligning with global efforts toward greener and more resource-efficient solutions.^13^

**Figure 1:**
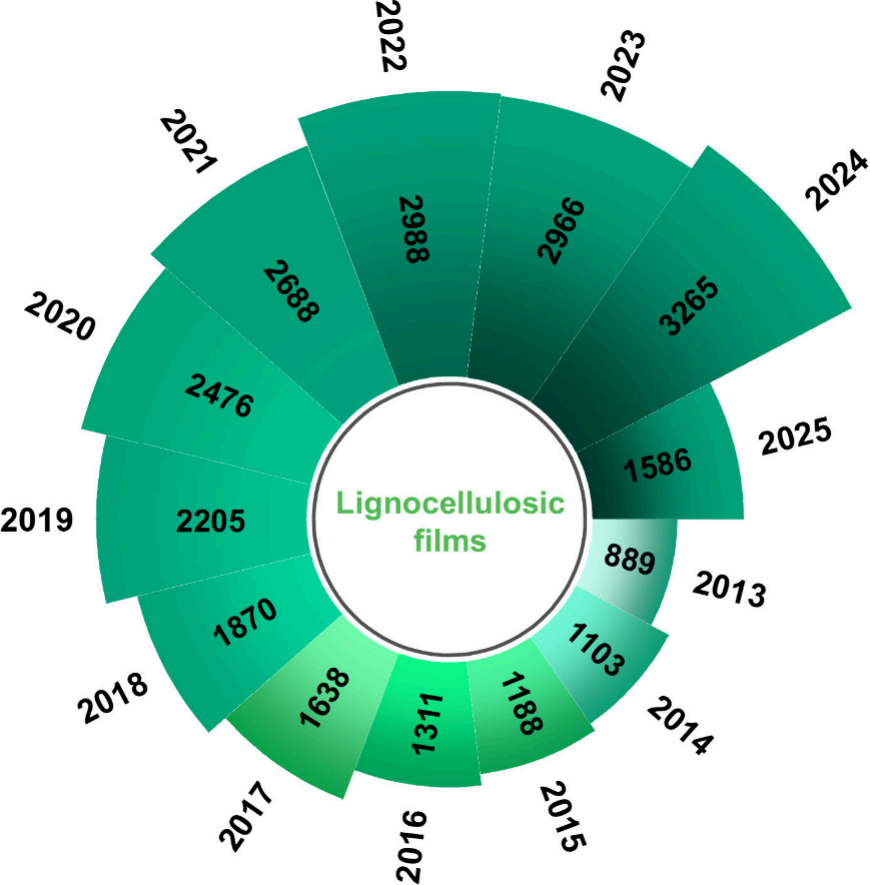
Annual number of publications on LCFs from Web of Science by June 2025, which is calculated as the sum of publications containing the keywords “cellulose film”, “hemicellulose film”, “lignin film”, and “lignocellulosic film”.

It is important to distinguish “lignocellulosic films” from other related lignocellulose-based materials such as hydrogels, aerogels, paper or nonwoven sheets. In this review, LCFs are defined as thin, flexible, compact, and continuous layers or sheets composed primarily of lignocellulosic components, such as cellulose, hemicellulose, lignin, or their combinations, with typical thicknesses in the range of 10–100 μm. This definition also includes membranes or mats formed from mechanically disintegrated or electrospun nanofibers derived primarily from lignocellulose, which are commonly categorized as thin films in the literature. Unlike paper and nonwoven sheets, which are fibrous, porous, and structurally discontinuous, LCFs are engineered for applications that often, but not always, require flexibility, optical transparency, and barrier performance. It should be noted that properties such as high transparency and gas/moisture barrier functionality are specific to certain types of cellulose-based films and do not apply universally across all categories of LCFs. This review focuses exclusively on LCFs as defined above and does not include traditional paper or nonwoven materials, aiming to provide an in-depth analysis of their preparation methods, functionalization strategies, and diverse applications.

Based on processing strategies and chemical compositions, LCFs can be categorized into cellulose derivative films, regenerated cellulose (RC) films, nanocellulose films, hemicellulose films, lignin films, and whole lignocellulosic biomass films. It is important to note that current industrial utilization is largely limited to cellulose-based films. In contrast, LCFs incorporating hemicellulose, lignin, or other functional components remain primarily at the research and development stage. As summarized in Figure 2, the development of LCFs has spanned over a century, with cellophane, one of the earliest commercial cellulose films, invented in 1908, marking a significant milestone in the utilization of renewable materials for packaging. Over the decades, advancements in cellulose processing, such as the development of RC films and nanocellulose-based films, have expanded the scope of LCF applications. More recently, the incorporation of functional polymers and nanomaterials into LCF structures has enabled the fabrication of multifunctional films, greatly enhancing their potential for advanced applications. Despite these advancements, many existing reviews focus narrowly on specific types of LCFs, such as nanocellulose films or RC films,^14,15^ without capturing the full spectrum and the diversity of this material class. Additionally, recent breakthroughs in LCF processing and functionalization, as well as their emerging applications in energy storage, energy harvesting, and flexible electronics, have not been comprehensively summarized. Addressing these gaps is critical for advancing the field and unlocking the full potential of LCFs for sustainable development.

**Figure 2:**
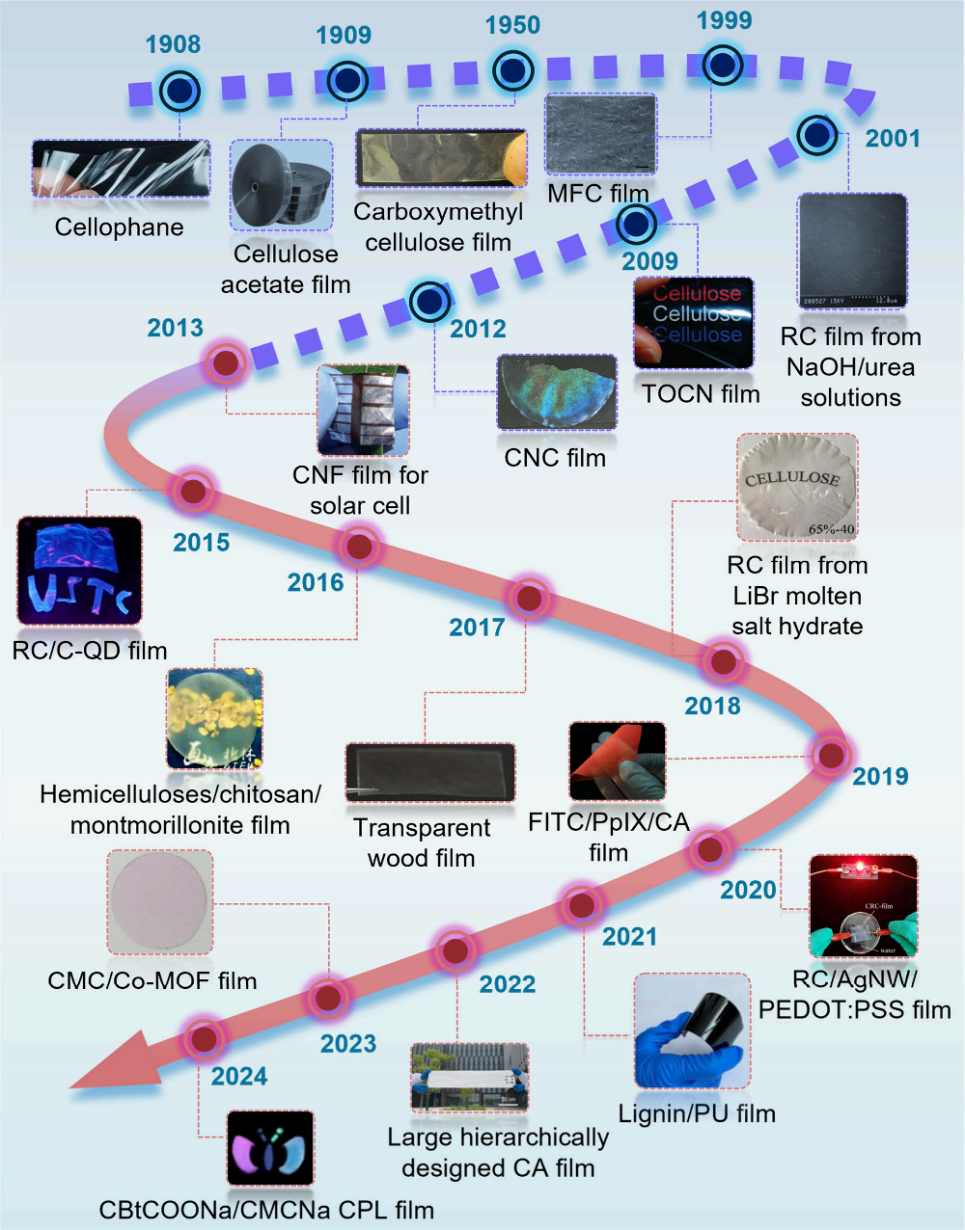
Evolution of key milestones in LCFs. SEM image of the surface of microfibrillated cellulose (MFC) film. Adapted with permission from ref 16. Copyright 1998 John Wiley and Sons. SEM image of the surface of RC film from NaOH/urea solutions. Adapted with permission from ref 17. Copyright 2001 American Chemical Society. Photograph of TEMPO-oxidized cellulose nanofibers (TOCNs). Adapted with permission from ref 18. Copyright 2008 American Chemical Society. Photograph of CNC iridescent film. Adapted with permission from ref 19. Copyright 2014 Springer Nature under a Creative Commons Attribution-NonCommercial-ShareAlike 3.0 Unported License. An organic solar cell on conductive nanopaper. Adapted with permission from ref 20. Copyright 2012 Royal Society of Chemistry. RC/carbon quantum dots (C-QD) films under visible light or a UV lamp. Adapted with permission from ref 21. Copyright 2014 Springer Science Business Media Dordrecht. Photograph of hemicellulose based composite films. Adapted with permission from ref 22. Copyright 2016 American Chemical Society. Photo of transparent wood film. Adapted with permission from ref 23. Copyright 2017 Wiley-VCH. Photo of cellulose film prepared in 65 wt % LiBr molten salt hydrate. Adapted with permission from ref 24. Copyright 2018 MDPI under CC BY 4.0 (https://creativecommons.org/licenses/by/4.0/). Image of a cellulose acetate (CA)-based dual-emission fluorescent film with a red initial fluorescence. Adapted with permission from ref 25. Copyright 2019 Springer Nature under CC BY 4.0 (https://creativecommons.org/licenses/by/4.0/). The conductive RC-based (CRC)-film as the conductor in a closed circuit. Adapted with permission from ref 26. Copyright 2019 Elsevier. Picture of the lignin-based polyurethane film (LPF). Adapted with permission from ref 27. Copyright 2021 Elsevier. A photograph of the hierarchically designed CA film. Adapted with permission from ref 28. Copyright 2022 The American Association for the Advancement of Science under CC BY 4.0 (https://creativecommons.org/licenses/by/4.0/). Color response of CMC/Co-MOF composite films toward ammonia. Adapted with permission from ref 29. Copyright 2023 Elsevier. Phosphorescence photograph of circularly polarized room temperature phosphorescence film. Adapted with permission from ref 30. Copyright 2024 Springer Nature under CC BY 4.0 (https://creativecommons.org/licenses/by/4.0/).

This review aims to provide a comprehensive and critical analysis of the current state-of-the-art in LCFs, encompassing their structure, preparation strategies, functionalization methods, and diverse applications. It highlights the sustainability advantages of LCFs while addressing the challenges associated with their large-scale production and integration into commercial markets. By summarizing the latest developments and identifying areas for future research directions, this review seeks to facilitate the broader adoption and further innovation of LCFs in both established and emerging applications. The scope of literature primarily spans publications from 2014 to 2025.

To guide the reader through the organization of this review, we include a schematic figure summarizing its main contents (Figure 3). Section 1 introduces the central theme and significance of LCFs. Section 2 presents the structural and chemical characteristics of lignocellulosic biomass, which provide the theoretical foundation for LCF fabrication. Section 3 explores the major types of LCFs and their preparation techniques and properties, while section 4 discusses functionalization strategies aimed at enhancing performance. Section 5 reviews the diverse applications of LCFs in fields such as food packaging, flexible electronics, energy, sensing, and water treatment. Section 6 assesses the economic and environmental feasibility of LCFs. Finally, section 7 identifies key technical challenges and offers perspectives for advancing the scalability and functionality of these sustainable materials. Through this structure, we aim to offer a logical and in-depth exploration of LCFs that will support further research and development in the field.

**Figure 3:**
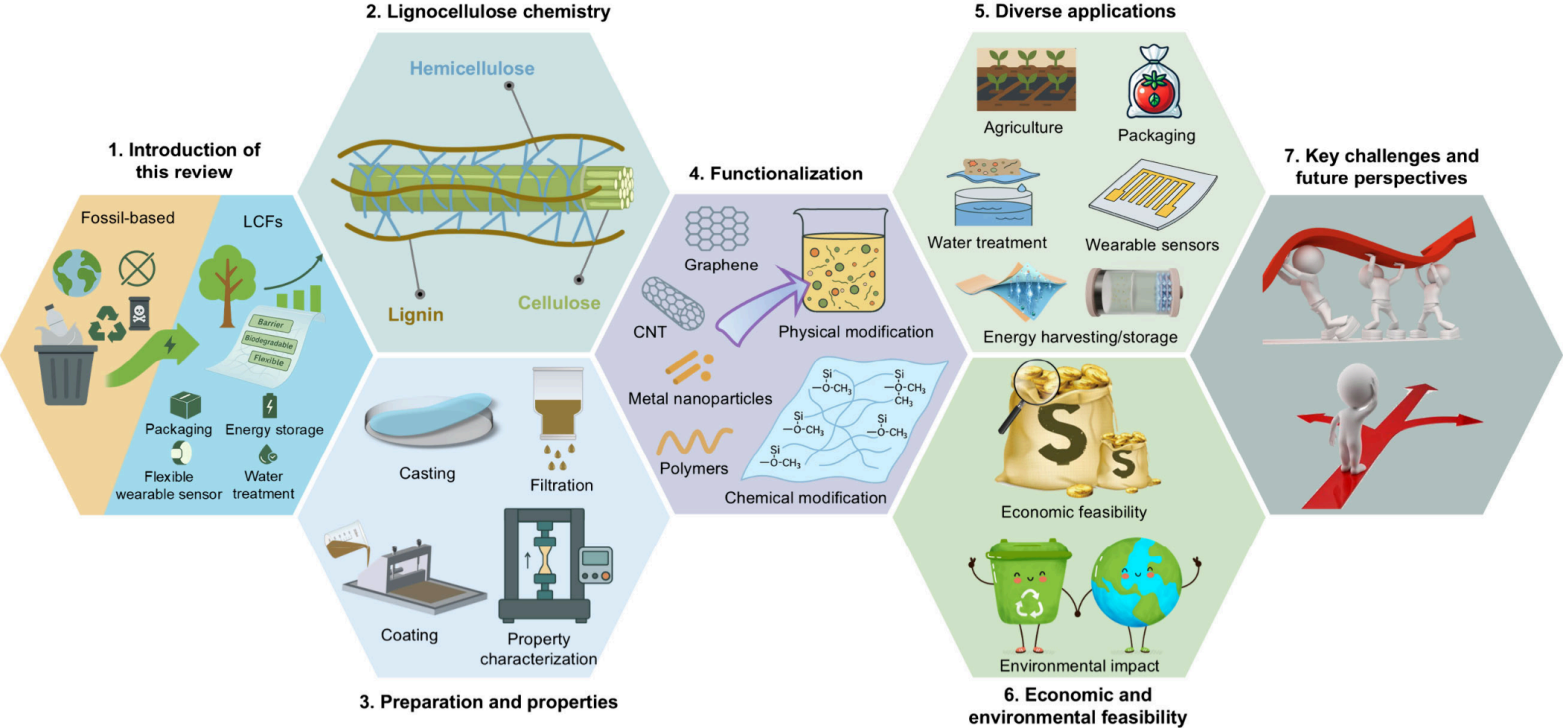
Structure of this review, covering: (1) introduction; (2) structure and chemistry of lignocellulosic biomass; (3) category, preparation, and properties of LCFs; (4) functionalization strategies; (5) applications in agriculture, packaging, electronics, energy, sensing, and water treatment; (6) economic and environmental feasibility; and (7) challenges and future directions.

## 2 Lignocellulose: Structure and Chemistry

### 2.1 Chemical Composition of Lignocellulose

As shown in Figure 4, the lignocellulosic biomass (e.g., wood, bagasse, grass, and agricultural wastes) is composed primarily of three constituents (cellulose, hemicellulose, and lignin), along with some minor constituents, such as water and solvent soluble organic matters (extractives) and inorganic matters (ashes or minerals), as further discussed below. Table 1 summarizes the representative contents of cellulose, hemicellulose, and lignin in different types of lignocellulosic biomass.

**Figure 4:**
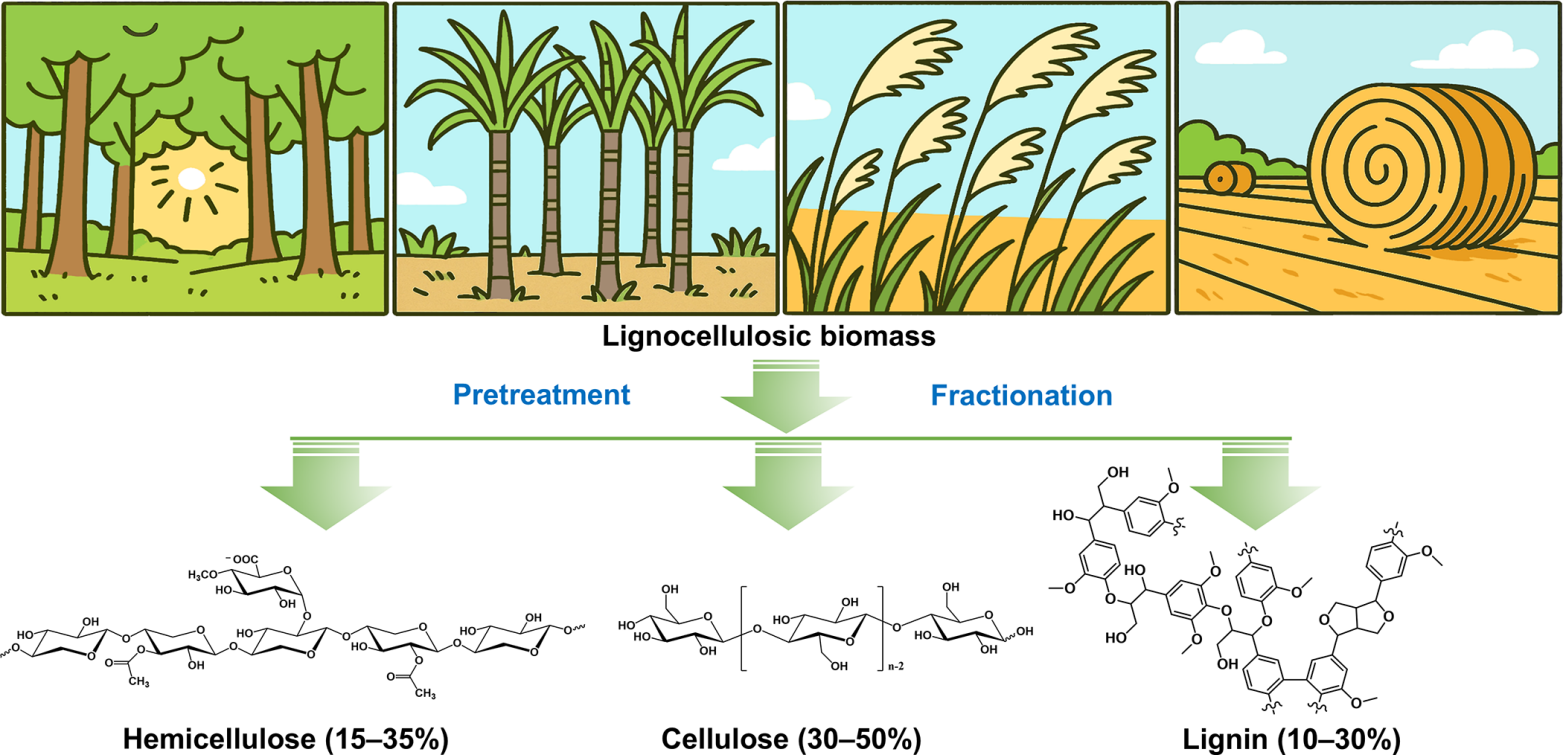
Various lignocellulosic biomass and the main constituents (cellulose, hemicellulose, and lignin).

**Table 1:** Main Composition of Lignocellulosic Biomass^31,32^

feedstock	cellulose (%)	hemicellulose (%)	lignin (%)
hardwood	43–47	25–35	16–24
softwood	40–44	25–29	25–31
bagasse	42–45	25–28	14–21
coir	32–43	10–20	43–49
cotton	95	2	1
flax	63–71	12–21	2–3
hemp	70	22	6
jute	71	14	13
sisal	73	14	11
rice straw	32–47	19–27	5–24
wheat straw	35–45	20–30	8–15
corn stover	21–37	22–31	14–20
switchgrass	35–40	25–30	15–20

#### 2.1.1 Cellulose

Cellulose is the structural or skeletal component of lignocellulosic biomass, comprising 30–50% of its dry weight, and is the most abundant natural polymer on Earth. The global annual production of cellulose is estimated at 150 billion tons.^33,34^ In certain plant species such as cotton, hemp, and jute, the cellulose content is significantly high, ranging from 60–95%.^31^ Since Anselme Payen first discovered and isolated cellulose in 1838, extensive efforts have been made on its biosynthetic process, physical and chemical properties, structural characteristics, chemical derivation of cellulose, and diverse applications, which have greatly advanced related disciplines.^35–37^ As a renewable and sustainable material, cellulose has attracted a great deal of attention from a wide variety of fields.^38–40^ The fascination of cellulose is due to its specific structure, which will be described in more detail in section 2.3.

#### 2.1.2 Hemicellulose

Hemicellulose is a group of heteropolysaccharides found in plants, making up 15–35% of plant cell walls, primarily in the primary and secondary cell walls of various plants.^41^ As the second most abundant polysaccharide in nature, the annual production of hemicelluloses is estimated at 60–70 billion tons.^42,43^ Depending on the plant species, hemicellulose comprises various sugars, including pentoses (d-xylose, l-arabinose), hexoses (d-glucose, d-galactose, d-mannose), and sugar acids such as d-glucuronic and d-galacturonic acids.^44,45^ The hemicellulose’s backbone can consist of single or multiple sugar units, such as xylose or a combination of xylose and mannose. Additional units like 4-*O*-methylglucuronic acid, galactose, arabinose, and acetyl group are often present as side groups of the hemicellulose backbone.^31^ Hemicellulose normally consists of 50–3000 sugar units and has a branched, random, and amorphous structure, contributing little to structural strength. It can be easily hydrolyzed by acids, bases, and hemicellulases into sugars.^46^ These sugars have commercial applications or potential as intermediates for synthesizing pharmaceuticals and health-promoting agents.^47^ Additionally, hemicellulose sugars can be converted into fuel ethanol and other high value-added chemicals (e.g., xylitol, lactic acid, furfural, and levulinic acid) through biological and chemical transformations.^48^

#### 2.1.3 Lignin

Lignin is a generic term for the aromatic polymers in plants.^49^ More specifically, lignin is biosynthesized by radical coupling reactions of three main monolignols: *p*-coumaryl (4-hydroxycinnamyl), coniferyl (4-hydroxy-3-methoxycinnamyl), and sinapyl (4-hydroxy-3,5-dimethoxycinnamyl) alcohols, which originate from phenylalanine via different pathways.^50^ These monolignols lead to hydroxyl-phenyl lignin (H-lignin), guaiacyl lignin (G-lignin) and syringyl lignin (S-lignin), respectively, and they are linked via C–O–C and C–C bonds.^31^ Lignin is the second most abundant organic polymer in nature after cellulose, constituting 30% of nonfossil organic carbon.^51^ The lignin content in wood species ranges from 20–35%, while aquatic and herbaceous angiosperms and many monocotyledons contain less lignin.^52^ Lignin is mainly located in the secondary cell walls, serving as an embedding material for cellulosic polymers, and it is also the major polymer in the middle lamellae between adjacent cell walls.^53^ Importantly, lignin provides the hydrophobic surface and stiffness of tracheid lumens, enabling plants to transport water to heights over 100 m, and contributes to the mechanical strength that can support trees weighing more than 2000 tons.^54^ Moreover, its unique physical and chemical properties provide a barrier against the invasion of pests and pathogens.^55^ However, lignin is the major barrier for the efficient extraction of cellulose fibers in the pulp and paper industry and poses a significant hurdle to the saccharification of cellulose and hemicellulose for the production of liquid biofuel in the bioenergy industry.^56,57^ Lignin is produced as a byproduct in the manufacturing of paper and biofuels. Globally, around 100 million tons of lignin are generated each year, with 70–90% originating from the pulp and paper industry.^58^ Traditionally, lignin has been considered a low-value waste product and is often burned for process heat because of the challenges in developing high-value lignin products due to its inherent heterogeneity and recalcitrance.^59^ However, with intensive studies of lignocellulosic biorefinery in recent years, various promising approaches have been developed to valorize lignin and produce high-value products such as carbon fiber, multifunctional hydrocarbons, syngas, and various platform chemicals (e.g., aromatic aldehydes, vanillic acid, syringic acid).^60–63^

#### 2.1.4 Minor Components

In addition to cellulose, hemicellulose and lignin, lignocellulosic biomass contains a small portion of other constituents such as extractives and ash. Extractives are nonstructural components of biomass, including fats, fatty acids, fatty alcohols, phenolics, waxes, proteins, essential oils, pectins, rosin, and other organic compounds, which can be extracted by solvents such as water, ethanol, acetone, and toluene.^64^ Each of these extractives has specific functions for the plant. The content and properties of the extractives vary from biomass to biomass and even vary between different parts of the same plant.^65^ The ash in lignocellulosic biomass typically comprises inorganic salts of calcium, potassium, magnesium, manganese, and sodium, oxides of iron and aluminum, and silica.^66^

### 2.2 Hierarchical Structure of Lignocellulose

Lignocellulosic biomass exhibits a hierarchical structure spanning from the macroscopic down to the molecular scale. In this section, we use wood as a representative example to illustrate the hierarchical structure of lignocellulose. Wood is the most abundant and industrially relevant lignocellulosic resource, and wood itself or wood pulp serve as the primary raw materials for producing most LCFs. Please note that nonwoody biomass, e.g., straw and bamboo, has distinct anatomic structure from woody biomass. Understanding the structural complexity of wood provides essential insights into how its composition and morphology influence film preparation and performance, and the principles described here are broadly applicable to other plant-derived lignocellulosic feedstocks.

Wood is composed of a diverse mixture of living and dead cells, with the cell wall serving as the supportive framework and housing the majority of wood components.^67^ Wood can be broadly classified into two categories: hardwood (e.g., birch, aspen, eucalyptus, and oak) and softwood (e.g., pine, spruce, cedar, and redwood). The key distinction between these two types lies in their reproductive processes: hardwood trees, scientifically known as angiosperms, are flowering plants with seeds within a protective structure, while softwood trees, categorized as gymnosperms, are coniferous plants whose seeds lack such enclosures.^68^ In general, hardwood is denser than softwood due to their structural composition. However, there are notable exceptions: balsa, a hardwood, is much less dense and softer than most softwoods, while yew, a softwood, is denser than many hardwoods. Softwoods account for approximately 80% of all timber used worldwide, as they are easier to cultivate, harvest, and process, making them the preferred choice for construction, paper, and general-purpose lumber.^69^

#### 2.2.1 Anatomic Structure of Wood

From a microstructural perspective, the anatomical structures of hardwoods and softwoods differ significantly. As illustrated in Figure 5a, hardwoods consist of three cell types: fibers (approximately 50%), vessels (about 30%), and parenchyma cells (around 20%).^70^ Fiber cells or tracheids, with a long and slender shape, provide mechanical support and are organized into a dense and honeycomb-like structure. Vessels, typically wider in diameter and shorter than fibers, are responsible for the transport of water and nutrients throughout the plant. Parenchyma cells, found as either box-like cells within the rays or elongated tubular cells associated with vessels, primarily functioning in storage and transport support.^71^ Differently, as shown in Figure 5b, softwoods consist predominantly of longitudinal tracheids (90–95%) and a small proportion of parenchyma and ray cells (5–10%).^69^ Tracheids serve a dual role, providing structural support while facilitating the transport of water and nutrients. Unlike hardwoods, softwoods completely lack vessels, making this a key anatomical distinction. Some softwoods, such as pine, may contain resin canals lined with epithelial cells, which are involved in resin production and defense. In papermaking, tracheids and fibers in softwood and hardwood are the most valuable components due to their long and robust structures, which enhance the strength and durability of paper products. In contrast, nonfibrous cells such as vessels and parenchyma tend to complicate processing by reducing drainage speed during pulp washing and paper formation and weakening paper structure. Given the uniformity and abundance of long tracheids, softwoods are generally considered superior feedstocks for pulp and paper production.

**Figure 5:**
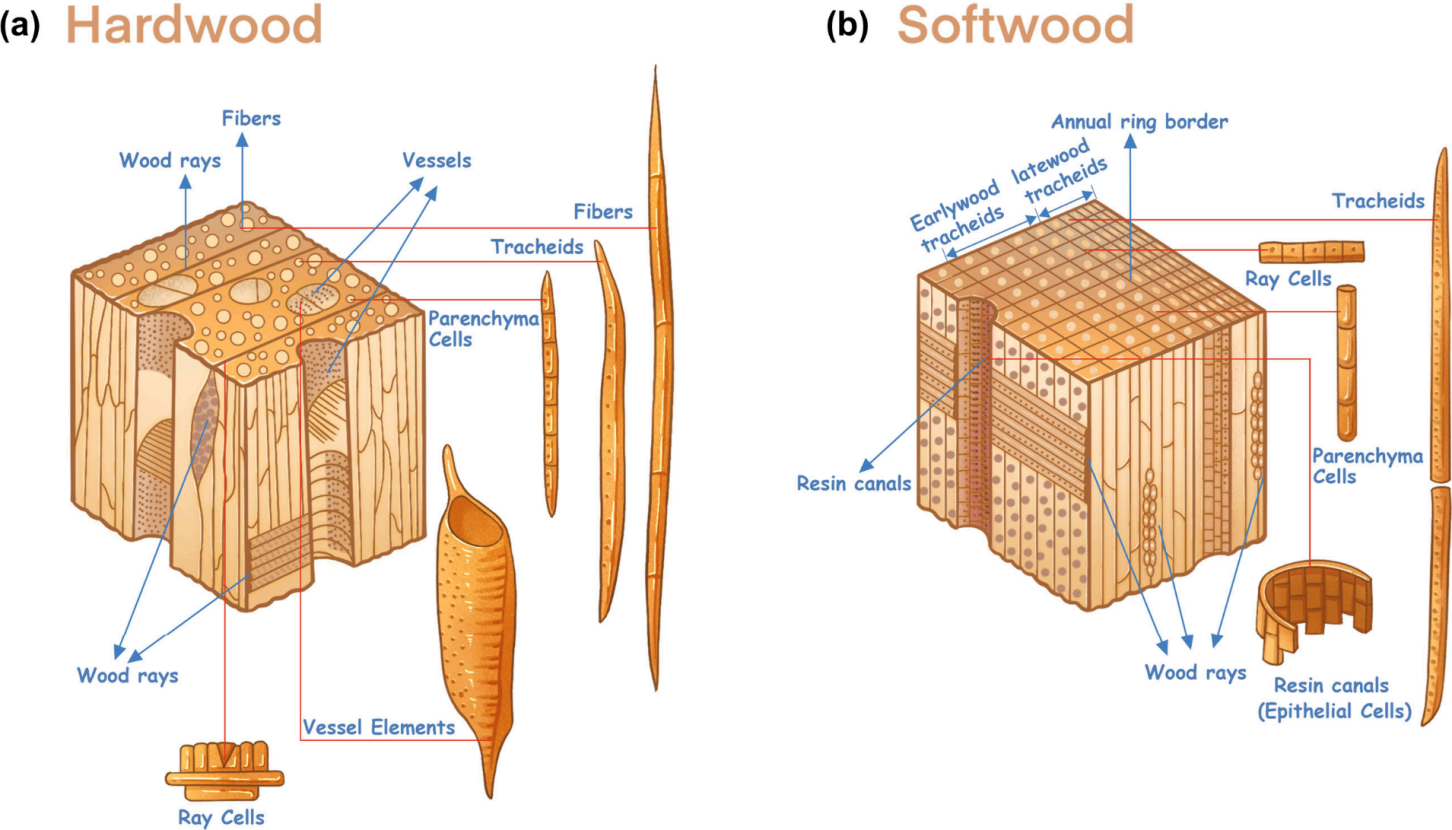
Cells that make up hardwood (a) and softwood (b), respectively.

Generally, cellulose fibers (pulps) for paper making can be produced through two different pulping methods: mechanical pulping and chemical pulping. In the mechanical pulping process, fibers are softened and separated from each other using mechanical grinding or refining at elevated temperatures.^72^ Since there is almost no loss of material in mechanical pulping, the mechanical pulp usually has a high yield of up to 90% based on the wood. However, the mechanical process is highly energy-intensive, and the resulting papers usually exhibit low strength due to the short fiber length, limited flexibility, and weak bonding capacity of the mechanical pulp.^73^ Chemical pulping, by contrast, achieves a lower yield (40–50%) but produces fibers with better mechanical strength. There are two main purposes in the chemical pulping process: (i) removing the lignin in the middle lamella to separate the individual fibers and (ii) removing lignin from the cell wall to make the fibers sufficiently flexible for higher paper strength.^74^ The sulfate (kraft) process and the sulfite process are the most important chemical pulping method, with kraft pulping being the most widely used and predominant method to date. Typically, kraft pulping removes about 90% of lignin.^73^ The remaining lignin can be further removed by the bleaching process, resulting in bleached pulp with less than 0.1% lignin.^75^

#### 2.2.2 Cell Wall Structure

As shown in Figure 6aii, the wood cell wall is comprised of multiple layers including the primary cell wall, secondary cell wall, and middle lamella. The secondary cell wall can be further divided into three layers, namely S1, S2, and S3.^76^ As shown in Figure 6b, the distribution of hemicellulose, cellulose, and lignin through the cell wall varies across different layers. In the middle lamella and primary wall, lignin is the dominant component, while cellulose and hemicellulose are present in smaller amounts. Moving inward, the S1 layer of the secondary wall shows a gradual increase in cellulose content, accompanied by significant hemicellulose and lignin levels. In the S2 and S3 layers of the secondary wall, cellulose becomes the most abundant component, with hemicellulose and lignin gradually decreasing. This structural composition highlights the varying roles of these biopolymers, with cellulose contributing to tensile strength, hemicellulose acting as a matrix, and lignin providing rigidity and hydrophobicity to the cell wall.^77^

**Figure 6:**
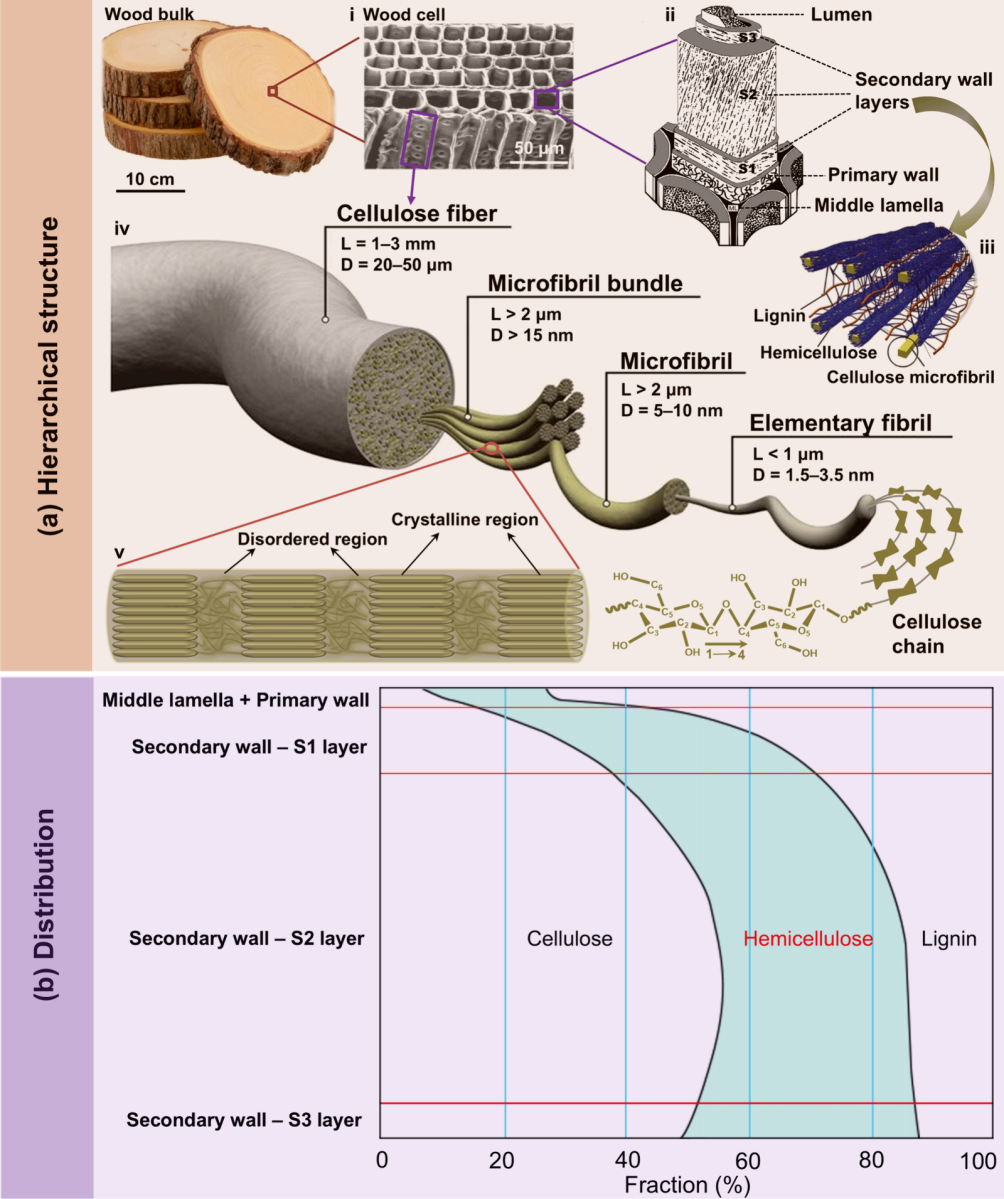
(a) Schematic illustration of the hierarchical structure of wood, from macroscopic to molecular scale. Wood cell image (i) is adapted with permission from ref 78. Copyright 2001 Elsevier. The cell wall comprised of multilayers (ii) is adapted with permission from ref 81. Copyright 2002 ACRIS under CC BY 4.0 (https://creativecommons.org/licenses/by/4.0/). Spatial arrangement of cellulose, hemicellulose and lignin in the cell wall of lignocellulosic biomass (iii) is adapted with permission from ref 82. Copyright 2011 Elsevier. The hierarchical structure of cellulose fiber (iv) is adapted with permission from ref 83. Copyright 2013 American Chemical Society. Schematic illustration showing the disordered and crystalline regions of cellulose (v). (b) Distribution of hemicellulose, cellulose, and lignin through the cell wall. Adapted with permission from ref 84. Copyright 2017 Springer Nature.

The primary cell wall is very thin and contains randomly oriented cellulose microfibrils surrounded by lignin, pectic substances, and hemicelluloses.^78^ Surrounding the primary cell wall is a thin lignin-rich layer (0.5–01.5 μm) called the middle lamella, which functions as the cellular glue to provide compressive strength to the plant tissue and stiffness to the cell wall.^79^ The secondary cell wall layers are the most important for the wood cell in terms of mechanical strength. Among these layers, the S1 layer is the thinnest layer, being only 0.1–0.35 μm thick, and is considered an intermediate between the primary cell wall and the S2 and S3 layers. The cellulose microfibrils angle relative to the cell axis in the S1 layer ranges from 60° to 80°. The S2 layer is the thickest layer (1–10 μm) in the secondary cell wall, contributing 75–85% of the total cell wall thickness and containing most of the total cellulose. The cellulose microfibrils angle in this layer is 5°–30° relative to the cell axis. The angle of cellulose microfibrils in the S2 layer can significantly influence the physical and mechanical properties of the cell, and even the stem wood as a whole. An increase in the cellulose microfibrils angle relative to the cell axis results in a decrease in the stiffness and longitudinal modulus of elasticity of the wood. The S3 layer is relatively thin, being 0.5–1.1 μm thick, with the cellulose microfibril angle ranging from 60° to 90° relative to the cell axis.^80^

#### 2.2.3 Spatial Arrangement and Functions of Cellulose, Hemicellulose, and Lignin in Cell Wall

Figure 6aiii illustrates the spatial arrangement of cellulose microfibrils, hemicellulose, and lignin in the cell wall. This arrangement allows cellulose microfibrils to be embedded in the hemicellulose-lignin network, much like steel bars embedded in concrete to form reinforced concrete.^43^ Among these components, cellulose microfibrils serve as the reinforcing steel bars, while the hemicellulose–lignin matrix acts as the concrete.^85^ Hemicellulose is thought to bind noncovalently to the cellulose microfibrils and covalently cross-link with lignin, forming a complex network of bonds that provides structural strength.^5^ It has been suggested that hemicellulose could also act as an amorphous matrix material, holding the stiff cellulose microfibrils in place.^86^ Lignin acts as a waterproof mechanical reinforcement element, which is covalently linked to hemicellulose. For decades, lignin has been considered the glue that binds cellulose microfibrils with hemicellulose,^87^ however, one recent study suggested that lignin and cellulose are spaced and joined by hemicellulose (e.g., xylan).^88^ It is reported that, in contrast to cellulose, the substitution of some hydrophobic groups such as acetyl and methyl groups could enhance the affinity of hemicellulose to lignin.^86^ Although several attempts to describe the specific locations of the different cell wall components and their mutual interactions have been made, the detailed structure of the lignin–carbohydrate complexes surrounding the cellulose microfibrils in the plant cell wall is still not well understood.^89^

### 2.3 Cellulose: Structure, Nanomaterials, and Derivatives

#### 2.3.1 Molecular Structure

Cellulose is a linear chain of ringed glucose with a flat ribbon-like conformation.^90^ The repeating anhydroglucose units are linked together through an oxygen covalently bonded to the C1 of one glucose ring and the C4 of the adjoining ring (1→4 linkage), namely β-1,4-glycosidic bond.^91^ The degree of polymerization (DP) of cellulose ranges from 10,000 to 15,000 depending on the cellulose sources.^92^ The three hydroxyl groups of the anhydroglucose unit are located at C2 (secondary), C3 (secondary), and C6 (primary). C2–OH and C3–OH are in equatorial position, while C6–OH is in axial position.^35^ These hydroxyl groups form strong intrachain (C2–OH···C6–OH, C5–O···C3–OH) and interchain (C3–OH···C6′–OH) hydrogen bonds, as discussed in detail in section 2.3.3, which contribute to the crystalline structure and physical properties of cellulose. Consequently, multiple cellulose chains form elementary fibrils of 1.5–3.5 nm in diameter. These elementary fibrils further aggregate into larger microfibrils with a diameter of 5–10 nm and several microns in length. Finally, the microfibrils and their bundles (typically 10–20 nm across),^93^ along with hemicellulose and lignin, form the secondary cell wall (Figure 6aiv).

#### 2.3.2 Crystalline Structure

As a result of the supramolecular structure of cellulose, cellulose microfibrils consist of both ordered (crystalline) and disordered (amorphous) regions, as illustrated in Figure 6av. Depending on the different crystal packings, there are four different polymorphs of cellulose: cellulose I, II, III, and IV, each with distinct structural and stability characteristics. Cellulose II, the most thermodynamically stable form, is commonly obtained through regeneration or mercerization of cellulose I and is widely used in commercial applications such as rayon and cellophane. Cellulose III, produced by treating cellulose I or II with liquid ammonia or amines, is primarily used in research due to its reversible structural transitions. Cellulose IV, formed by heating cellulose III under specific conditions, is a less common polymorph studied for its theoretical significance. Notably, cellulose I is naturally produced by plants and bacteria and is sometimes called “natural” cellulose.^90^ Cellulose I has two crystal forms, namely Iα and Iβ, which coexist in different proportions depending on the sources. Cellulose in higher plants (e.g., cotton, trees) cell walls and in tunicates are rich in Iβ, whereas some algae and bacteria are predominantly composed of Iα.^95^ As illustrated in Figure 7a, cellulose Iα displays a one-chain triclinic unit cell (P_1_ symmetry), while cellulose Iβ displays a two-chain monoclinic unit cell (P_21_ symmetry). For the cellulose Iα unit cell, the unit-cell parameters are *a* = 0.672 nm, *b* = 0.596 nm, *c* = 1.040 nm, α = 118.08°, β = 114.80°, γ = 80.375°. For the cellulose Iβ unit cell, the unit-cell parameters are *a* = 0.778 nm, *b* = 0.820 nm, *c* = 1.038 nm, α = 90°, β = 90°, γ = 96.5°.^90^ For both cellulose Iα and Iβ, the cellulose chains making up the sheets are parallel and aligned along the crystallographic *c*-axis, as displayed in Figure 7b. Parts c and d of Figure 7 illustrate a perpendicular view to the *c*-axis of a 36-chain elementary fibril of cellulose Iα and cellulose Iβ, respectively. At the molecular level, cellulose Iα and Iβ share similar conformation and chain packing in the a–b planes.^96^ The main difference between these two polymorphs is the relative displacement of hydrogen-bonded planes, i.e., (110) plane for cellulose Iα and (200) plane for cellulose Iβ, in the *c*-axis direction. More specifically, hydrogen-bonded planes in cellulose Iα always displace along the molecular axis by +*c*/4, whereas for cellulose Iβ the displacement between hydrogen-bonded planes alternates between +*c*/4 and −*c*/4.^90^

**Figure 7:**
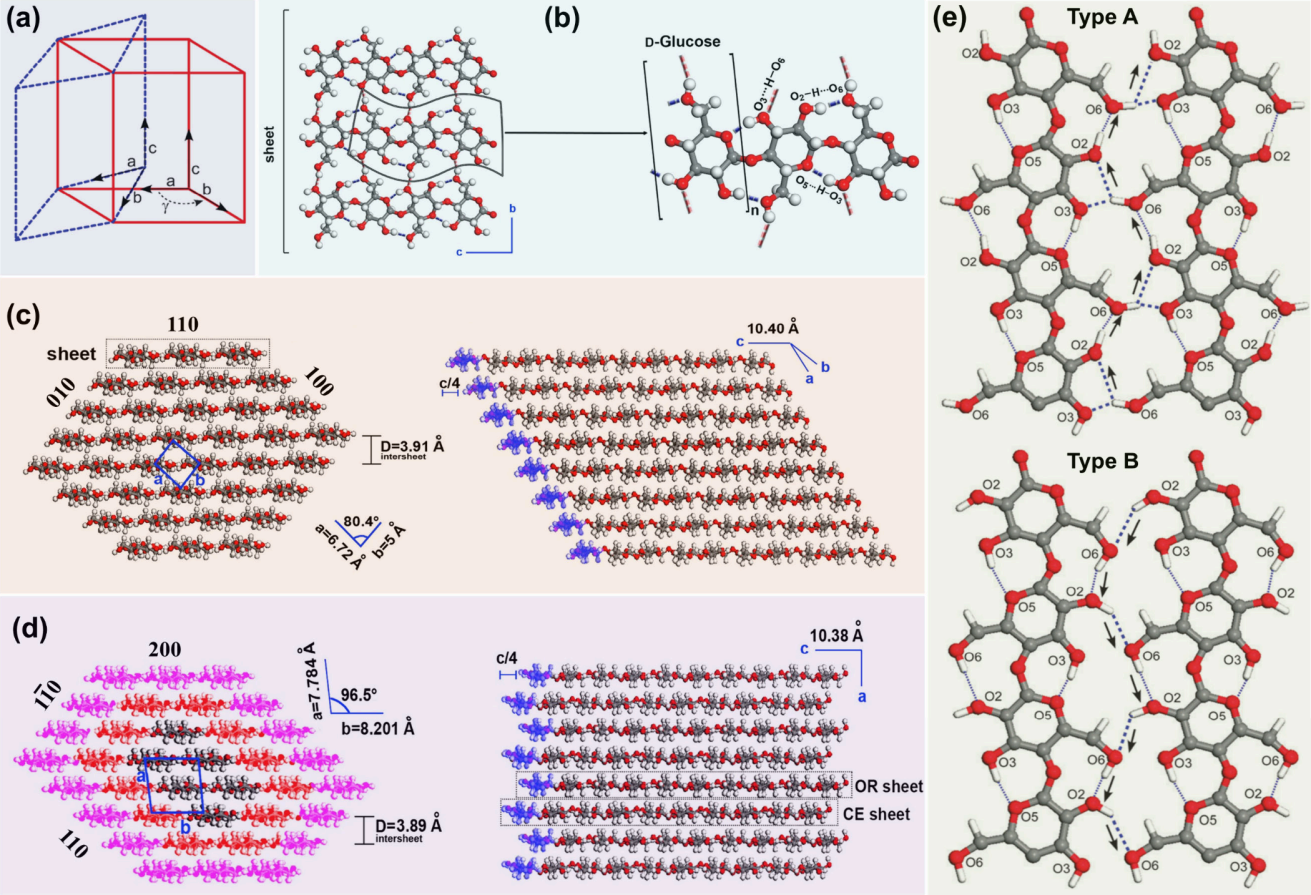
(a) Schematic configuration of the unit cells for cellulose Iα (dashed line) and Iβ (solid line). (b) Illustration of a cellulose elementary fibril sheet which contains an arrangement of cellulose chains interconnected by hydrogen bonds (left) and one cellulose chain contains three d-glucose repeated units with hydrogen bonds. (c) Illustrations of a perpendicular view to the *c*-axis of a 36-chain elementary fibril of cellulose Iα, where axes *a*, *b*, and *c* (in blue color) define the triclinic unit cell formed by one chain (left) and a longitudinal view of parallel sheets packing in cellulose Iα, where each sheet is shifted along the *c*-axis by +*c*/4 (right). (d) Illustrations of a perpendicular view (left) and a longitudinal view (right) of a 36-chain elementary fibril of cellulose Iβ. Axes *a*, *b*, and *c* define the monoclinic unit cell made up of two chains namely origin (OR) chain and center (CE) chain of cellulose Iβ. In the perpendicular view, the 36 cellulose chains are hexagonally arranged in the transverse section of the elementary fibril, where 18 chains (in pink color) represent the outer chains that sheath other subcrystalline 12 chains (in orange color), and inner 6 chains form the crystalline core. In the longitudinal view, there is an alternated displacement between OR and CE sheets by c/4 in the parallel sheets of cellulose Iβ. (e) Schematic illustration of two suggested hydrogen bond patterns within the hydrogen-bonded plane ((110) plane for cellulose Iα and (200) plane for cellulose Iβ). Note: thin dotted lines refer to intrachain hydrogen bonds, thick dotted lines refer to the interchain hydrogen bonds, arrows show the donor–acceptor–donor directions. (a,e) Adapted with permission from ref 90. Copyright 2011 Royal Society of Chemistry. (b–d) Adapted with permission from ref 94. Copyright 2019 Elsevier.

#### 2.3.3 Hydrogen Bonding

For both cellulose Iα and cellulose Iβ, a remarkable feature is that cellulose chains are well-stabilized within the sheets (hydrogen-bonded planes) by intrachain and interchain (O–H···O) hydrogen bond interactions, as illustrated in Figure 7b.^94^ As shown in Figure 7e, two coexisting hydrogen bond patterns have been reported, which are well described in previous work.^95^ It is reported that cellulose Iβ has a higher percentage (70–80%) of Type A hydrogen bond configuration in the hydrogen-bonded planes than cellulose Iα, resulting in a better bonding geometry. Moreover, within other planes, i.e., (100) and (010) planes for cellulose Iα and (110) and (1–10) planes for cellulose Iβ, the number of hydrogen bonds in cellulose Iβ is believed to be higher than that in cellulose Iα.^97^ Thus, cellulose Iβ shows higher stability than cellulose Iα. This inference has been supported by the fact that heating of the cellulose Iα causes irreversible conversion to cellulose Iβ.^96^ From the Type A hydrogen bond pattern (Figure 7e), it is clear to see that the intrachain hydrogen bonds include O3–H···O5 and O2–H···O6 bonds while the interchain hydrogen bonds are dominated by O6′–H···O3 bonds. As show in Figure 8a, the intrachain hydrogen bonds limit the rotation of glucose units in the linear chains around the glycosidic linkages and contribute to the linear configuration of cellulose chain, whereas the interchain hydrogen bonds are beneficial to the sheet stability.^98^ Further, the intersheet interactions involved both C–H···O pseudo hydrogen bonds and van der Waals interactions ensure the sheets stack on top of each other to form a 3D crystal structure.^94^ Gross and Chu^99^ investigated and compared the interaction network of cellulose Iα and cellulose Iβ using all-atom molecular dynamics (MD) simulations. As indicated in Figure 8b, while both cellulose Iα and Iβ possess almost the same amount of intrachain and interchain hydrogen bonds, cellulose Iβ has two more intersheet pseudo hydrogen bonds per glucose than cellulose Iα. This finding further supports the fact that cellulose Iβ is the more stable form. Additionally, the number of C–H···O pseudo hydrogen bonds per glucose is much higher than the sum of intrachain and interchain hydrogen bonds. Although individual C–H···O pseudo hydrogen bonds show a much lower interaction energy of 2 kcal/mol than O–H···O hydrogen bonds (6 kcal/mol), the large amount of these pseudo hydrogen bonds, combined with van der Waals interactions, make intersheet interactions stronger than interchain interactions.

**Figure 8:**
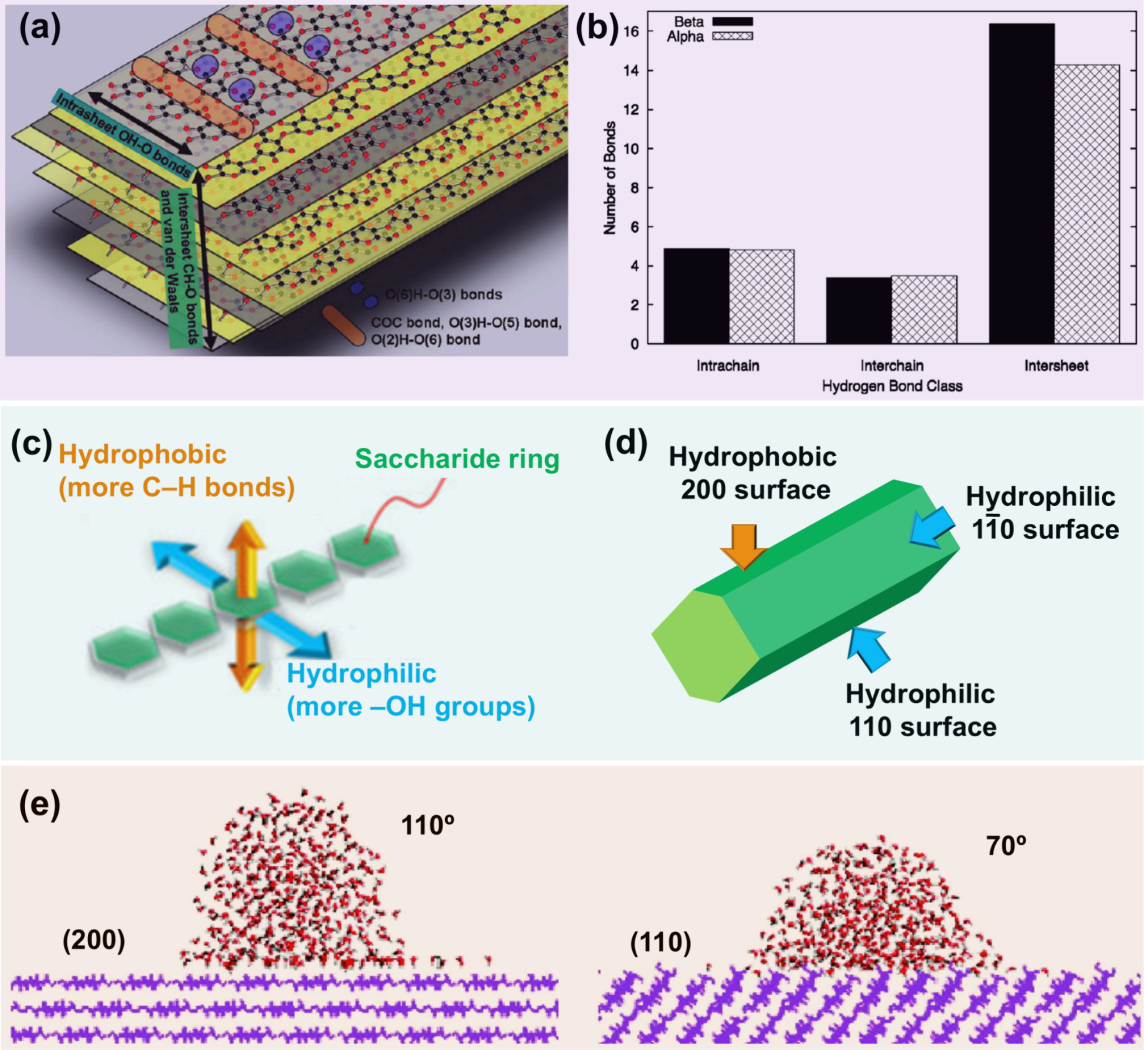
(a) Schematic diagram of hydrogen-bond network and layer structure for cellulose Iβ. Adapted with permission from ref 98. Copyright 2011 American Chemical Society. (b) Average number of hydrogen bonds per glucose of cellulose Iα and cellulose Iβ. Adapted with permission from ref 99. Copyright 2010 American Chemical Society. Schematic illustrations of cellulose molecular chain (c) and crystal planes (d) of cellulose Iβ showing amphiphilicity. (e) Water wetting of the (200) and (110) surfaces of cellulose Iβ. Adapted with permission from ref 100. Copyright 2008 American Chemical Society.

#### 2.3.4 Wetting Behavior and Amphiphilic Character

In general, natural cellulose is considered highly hydrophilic due to the existence of abundant hydroxyl groups. However, several studies have proven that cellulose also exhibits a hydrophobic character due to its anisotropic supramolecular structure. As illustrated in Figure 8c,d, the surfaces of cellulose Iβ are topographically and chemically heterogeneous, i.e., plenty of −OH groups appear on the (110) and (1–10) surfaces, while the (200) surface has the most −CH groups exposure with buried −OH groups, leading to the amphiphilicity of cellulose.^101,102^ Figure 8e displays the water wetting behavior of the (200) and (110) surfaces of cellulose Iβ using atomistic MD simulations.^100^ It can be clearly seen that the (200) surface exhibits a much higher contact angle than (110) surface, indicating higher hydrophobicity.

Based on the amphiphilic nature of cellulose, Zhao et al.^103^ proposed a theory of cellulose disintegration along the crystallographic planes induced by mechanical force and microenvironmental polarity. As illustrated in the cross-section of cellulose microfibrils (Figure 9a), elementary fibrils in the microfibrils are associated with each other through close contact. As described above, different crystallographic planes exhibit different supramolecular structures. Consequently, the interactions of these planes with different solvents vary significantly.^104^ Therefore, it is expected that both disintegration and reorganization of the cellulose microfibrils will be affected by changing the type of medium during mechanical shearing.^105^ For instance, when milling cellulose in polar solvents such as water, dimethylformamide (DMF) and dimethyl sulfoxide (DMSO), cellulose nanofibrils (CNFs) can be obtained, as illustrated in Figure 9a. This result is likely due to polar solvents having great affinity to hydrophilic (110) and (1–10) surfaces, allowing them to penetrate the interstitial space between these hydrophilic planes of the tightly bound elementary fibrils. During the ball milling process, the strong impact and frictional shear accompanying the solvent swelling effect cause the big microfibril bundles to be disintegrated into CNFs. Intriguingly, the fibrillation efficiency could be remarkably enhanced by simultaneously applying esterification during ball milling. More importantly, the functionality of the as-prepared CNFs could be tuned by changing esterifying agents.^106^ For example, by milling corncob cellulose in DMF with hexanoyl chloride as an esterifying agent, thin and hydrophobic CNFs with a diameter approximately 1.5–2.8 nm could be obtained, as shown in the AFM images (Figure 9c,d). In another case, hydrophilic CNFs could be prepared by milling cellulose in DMSO with succinic anhydride. It was found that the succinylated CNFs exhibited good dispersibility in aqueous media with a zeta potential around −34 mV.^107^

**Figure 9:**
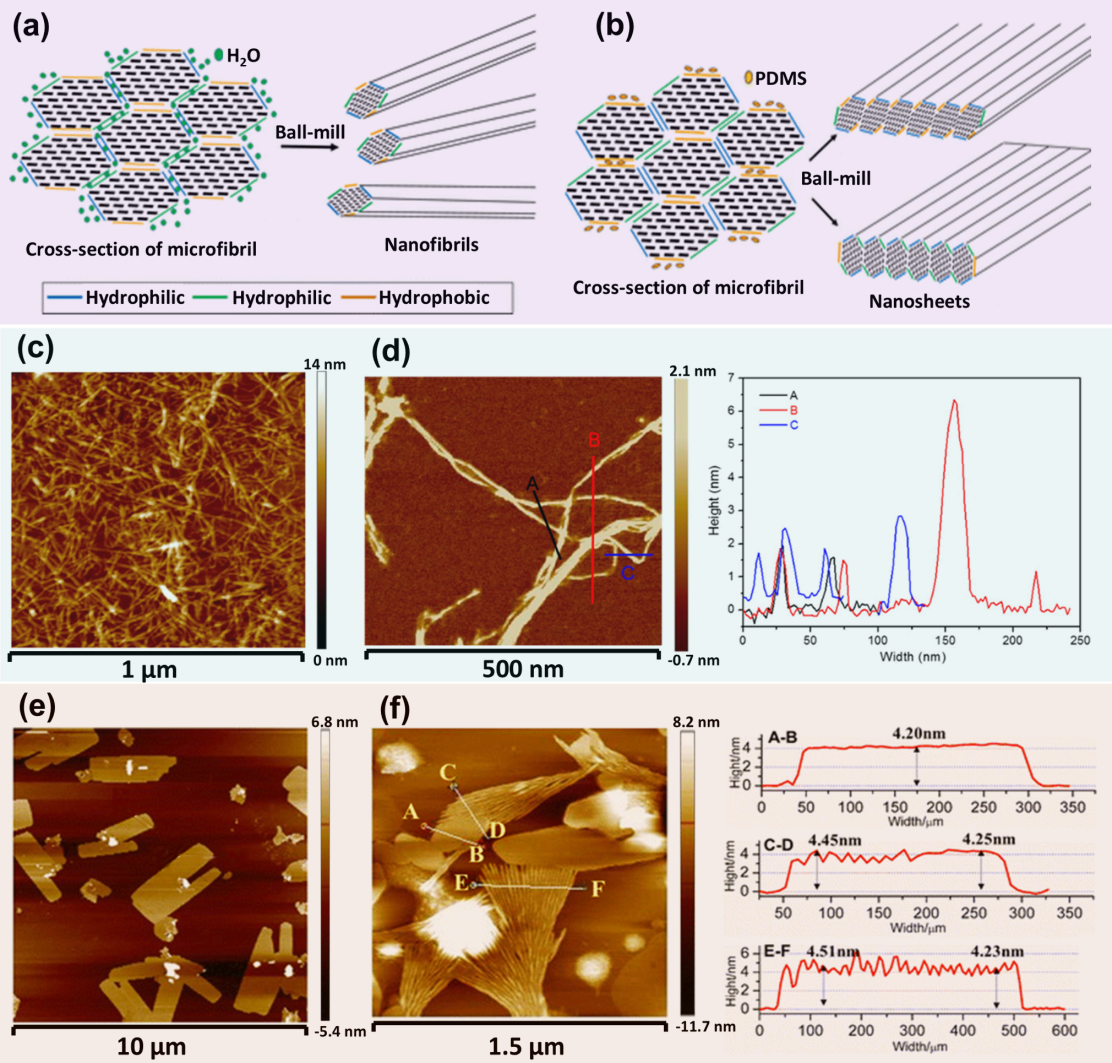
Schematic illustration of disintegration of cellulose in water (a) and polydimethylsiloxane (PDMS) (b). (c) AFM image of esterified CNFs produced through one-step mechanochemical esterification by ball milling in DMF. (d) Magnified AFM image of the CNFs and the corresponding height profile. (e) AFM image of cellulose nanosheets (CNS) prepared by ball milling in PDMS. (f) AFM image of the CNS after sonication in ethanol for 60 min and the corresponding height profile. (a,b,e,f) Adapted with permission from ref 103. Copyright 2016 Springer Nature. (c,d) Adapted with permission from ref 108. Copyright 2017 American Chemical Society.

As shown in Figure 9b, nonpolar solvents such as PDMS, toluene, and stearic acid exhibit strong affinity to hydrophobic (200) surfaces. When cellulose is milled in these nonpolar solvents, breaking interfaces mainly occur at the interstitial space between these hydrophobic planes while the hydrophilic (110) and (1–10) planes remain connected, resulting in the formation of CNS. As displayed in Figure 9e, thin CNS with a thickness around 4.2 nm (consistent with the thickness (3–5 nm) of elementary fibrils) and varied lateral size and shape were successfully produced by milling cellulose in PDMS for 12 h (with a 5 min pause after every 20 min). Additionally, Figure 9f shows the AFM image of the CNS after sonicating in ethanol for 60 min. Some CNS can be spread into 4.2–4.5 nm wide elementary fibrils with fan-like shapes, indicating that the CNS can be further disintegrated into nanofibrils. According to the above discussion, it can be concluded that the solvent polarity plays an important role on the hydrogen bonds or van der Waals force among cellulose molecular chains and crystallographic planes during mechanical disintegration.^109^ This fundamental knowledge provides evidence for the fact that most of the cellulose nanomaterials produced from lignocellulosic biomass exhibit fibrous morphology, as the processing media to produce cellulose nanomaterials is mainly aqueous solutions.

#### 2.3.5 Cellulose Nanomaterials

Cellulose nanomaterials, commonly referred to as nanocellulose, serve as the foundational building blocks for nanocellulose films, which represent one of the six major categories of LCFs discussed in this review. This section provides a brief overview of their categories, properties, and preparation methods to support the later discussion on nanocellulose-based LCFs. Nanocellulose has garnered significant attention from both academic and industrial researchers due to its unique nanostructure and impressive physicochemical properties such as high tensile strength (2–6 GPa) and elastic modulus (130–150 GPa), high specific surface area (up to several hundreds of m^2^/g), low density (1.6 g/cm^3^), and reactive surfaces, as well as biodegradability and renewability.^110–113^ Therefore, nanocellulose has been widely used in diverse fields such as reinforcing agents, coatings, rheology modifiers, optical materials, electroconductive materials, sensors, and biomedical materials.^114–116^ Based on the size, morphology, and preparation techniques, CNCs and CNFs are the two main categories of nanocellulose produced from lignocellulosic biomass through a top-down process (Figure 10a).^117,118^

**Figure 10:**
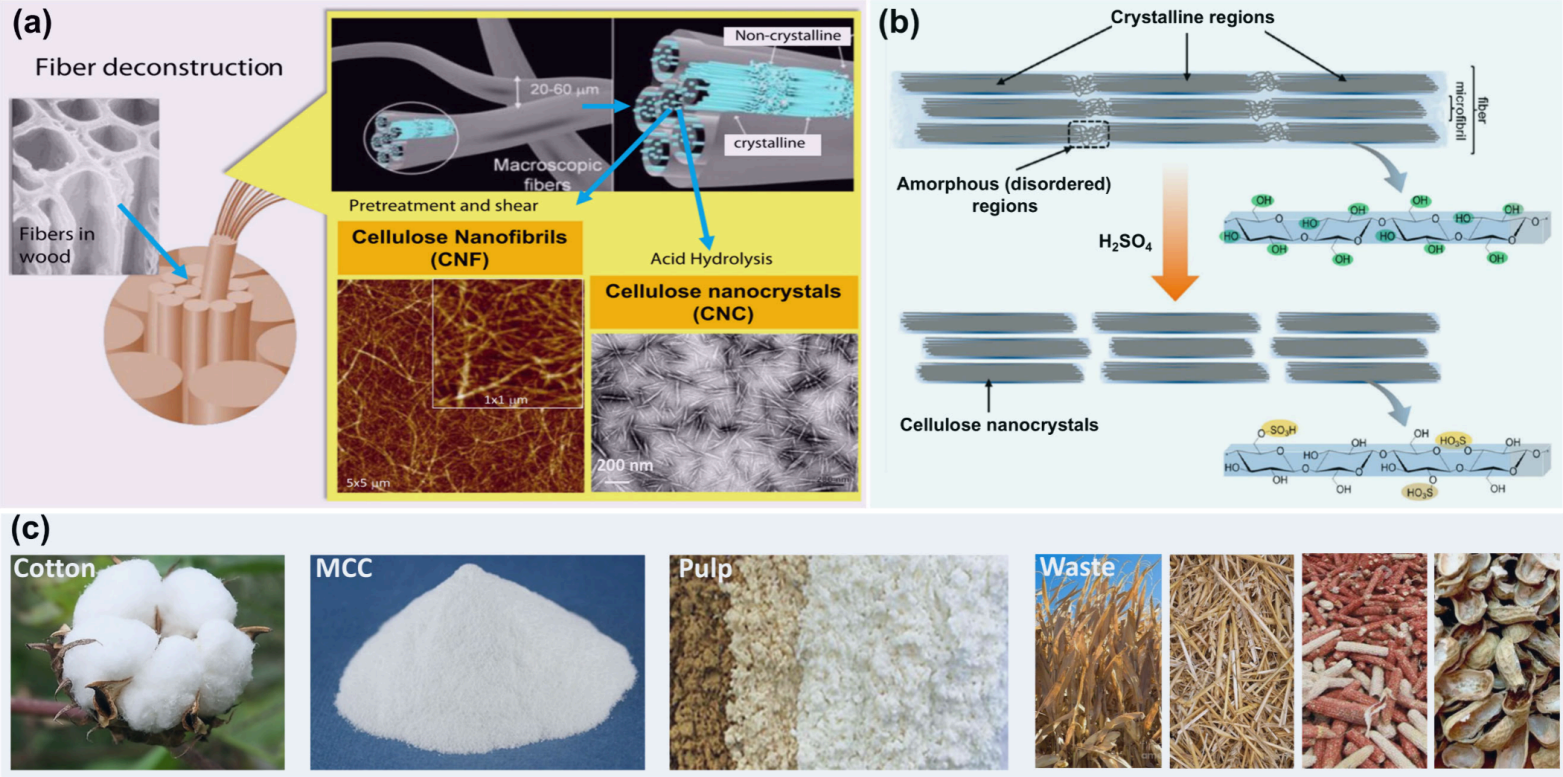
(a) Schematic illustration of CNFs and CNCs production from fiber cell walls by mechanical and chemical treatments, respectively. Adapted with permission from ref 120. Copyright 2014 Elsevier. (b) Schematics of idealized cellulose fibers showing one of the suggested configurations of the crystalline and amorphous regions, and CNCs after sulfuric acid hydrolysis of the amorphous regions, exhibiting the characteristic sulfate half-ester surface groups formed as a side reaction. Adapted with permission from ref 121 Copyright 2014 American Chemical Society. (c) Representatives of feedstocks used to produce CNCs and CNFs. Reproduced from Du, H. Ph.D. Theses, Auburn University, 2021.^122^

To date, a variety of lignocellulosic biomass such as cotton, microcrystalline cellulose (MCC), wood pulp, and different kinds of agricultural wastes have been used as the feedstocks to produce CNCs and CNFs (Figure 10c). Additionally, the commercialization of CNCs and CNFs has been realized by some companies (e.g., CelluForce, Borregaard, Nippon Paper Group) on the tons-per-day scale.^119^ According to a recent report by Zion Market Research, the global nanocellulose market was valued at approximately 410 million USD in 2023, and this number is anticipated to reach around 1886 million USD by the end of 2032. Notably, CNFs accounted for the major market share in 2023 and are expected to continue their dominance during the forecast period.

CNCs, sometimes termed nanocrystalline cellulose (NCC) or cellulose nanowhiskers (CNW), are rigid rod-like particles of 10–30 nm in diameter and several hundred nanometers in length, and they are mostly crystalline.^123^ The typical method for the preparation of CNCs is inorganic acid hydrolysis, with sulfuric acid being the most used acid for producing sulfonated CNCs with good dispersibility in water.^124^ Some other inorganic acids such as hydrochloric acid,^125,126^ phosphoric acid,^127,128^ hydrobromic acid^129^ and nitric acid^130,131^ have also been used for the preparation of CNCs. As shown in Figure 10b, during the sulfuric acid hydrolysis process, the disordered regions of cellulose are more easily degraded while the crystalline regions of cellulose remain as CNCs because of their inherent structure stability.^90,132^ Additionally, the introduction of sulfate half-ester surface groups endows the CNCs with good dispersibility in aqueous media.^121^ Strong acid hydrolysis is indeed a simple and time-saving method for the preparation of CNCs.^131^ However, certain issues such as harsh corrosion of equipment, severe environmental pollution, large water usage, and low production yield need to be addressed.^111^ Thus, some sustainable and environmentally friendly methods based on the use of recyclable chemicals have been developed to address these drawbacks, such as inorganic solid acid (e.g., phosphotungstic acid) hydrolysis,^133,134^ organic acid (e.g., formic acid, oxalic acid) hydrolysis,^123,135–137^ ionic liquid treatment,^138,139^ deep eutectic solvent (DES) treatment,^140,141^ among others.^142^

CNFs, sometimes termed nanofibrillated cellulose (NFC) or microfibrillated cellulose (MFC), are flexible fiber-like structures with a diameter of less than 100 nm and a length of 500 nm or even longer, which are composed of both disordered and crystalline domains.^143^ Different from CNCs, CNFs are mainly obtained by mechanical treatment of cellulosic fibers, such as high-pressure homogenization, microfluidization, grinding, ultrasonication, and ball milling.^144–146^ Figure 11a illustrates the corresponding instruments and their schematic diagrams. Among these methods, high-pressure homogenization and microfluidization are the most widely used for the preparation of high-quality CNFs.^147^ Both homogenization and microfluidization follow similar working mechanisms but have different configurations. In general, the homogenizer works at a pressure below 150 MPa, while the microfluidizer can provide a pressure over 250 MPa. During the mechanical fibrillation process, the homogenizer or microfluidizer generates various forces such as high pressure, high shear, high speed, and turbulence through the rapid change of pressure, which can break down the cellulose fibers and release aggregated nanofibrils.^148^ However, mechanical fibrillation by high-pressure homogenization or microfluidization for CNF production is very energy-intensive, with an estimated energy consumption in the range of 12–70 MWh/t.^149^ Another issue with homogenization and microfluidization is clogging when processing cellulose fibers, mainly attributed to the fact that long cellulose fibers tend to form entanglements.^147^

**Figure 11:**
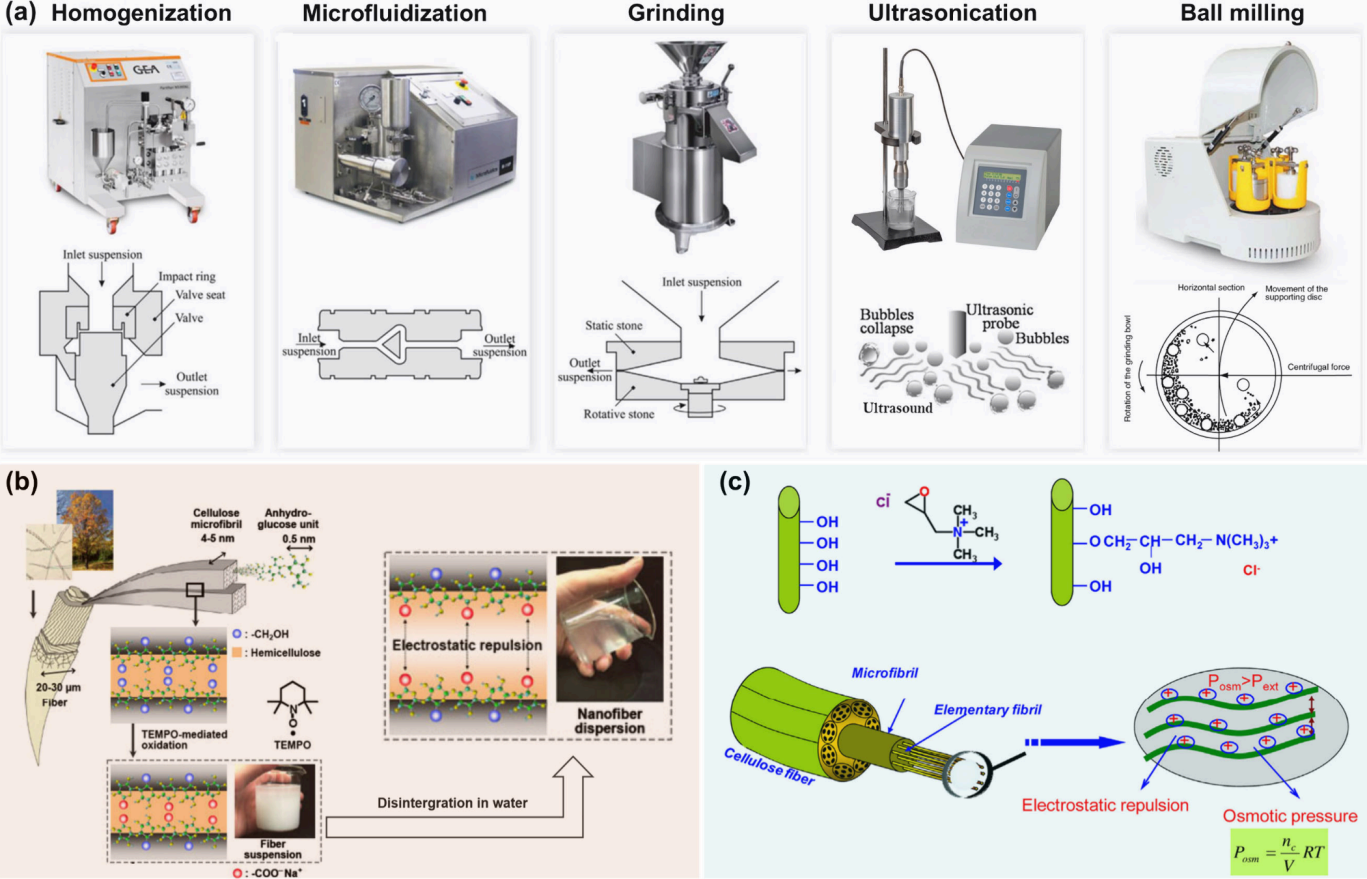
(a) Representatives of mechanical fibrillation approaches and the corresponding instruments and their schematic diagrams. Adapted with permission from refs 145,150,151 . Copyright 2016 Elsevier; Copyright 2021 Elsevier; Copyright 2001 Elsevier. (b) Systematic diagram of the preparation of CNFs by surface carboxylation using TEMPO oxidation. Adapted with permission from ref 114. Copyright 2019 Elsevier. (c) Schematic illustration of how the quaternization enhanced the fibrillation process. Adapted with permission from ref 152. Copyright 2015 Elsevier.

In order to reduce energy consumption and avoid clogging issues, some pretreatment methods have been proposed in recent years, including enzymatic hydrolysis,^153–156^ mineral acid hydrolysis,^157,158^ TEMPO-mediated oxidation,^159,160^ carboxymethylation,^161,162^ quaternization,^163,164^ among others. Among them, enzymatic or acid hydrolysis is intended to break down the big fibers into small pieces while swelling the cellulose fibers. The other pretreatment approaches are designed to introduce surface charges onto the cellulose fibers. The negative or positive charges on the surface of cellulose fibers could reduce the energy input needed for fibrillation by electrostatic repulsion and enhance the colloidal stability of the final CNFs.^145^ For example, negative carboxyl groups can be introduced on the cellulose pulp by TEMPO oxidation, which selectively oxidizes the cellulose C6 hydroxyls to carboxylates.^165–167^ The mechanism of the oxidation reaction is shown in Figure 11b. After mild mechanical disintegration, a transparent and gel-like CNF suspension can be obtained. Additionally, the mechanical energy consumption after TEMPO oxidation can be significantly reduced from 70 MWh/t (without pretreatment) to 1–10 MWh/t. Chaker and Boufi^153^ demonstrated another strategy by introducing positive quaternary trimethylammonium groups on the surface of cellulose fiber to increase the electrostatic repulsion and enhance fibrillation efficiency, as shown in Figure 11c. Saini et al.^164^ evaluated that the energy consumption at fibrillation stage could be reduced by five times using this cationic modification pretreatment. Interestingly, the introduced cationic groups endowed the final CNFs with antibacterial properties, which could be applied to food packaging and biomedical applications. Nevertheless, the above conventional pretreatment methods still face several deficiencies such as the high cost of enzymes and chemicals, time-consuming process, difficulty in recovering chemicals, and environmental issues.^147^ Thus, it is of critical importance to develop more cost-effective, sustainable, and environmentally benign pretreatment methodologies for the preparation of high-quality CNFs.

#### 2.3.6 Cellulose Derivatives

Due to its crystalline structure and strong intra- and intermolecular hydrogen bonding, cellulose is insoluble in water and most organic solvents and cannot be processed by melting as it decomposes before reaching its melting point.^168^ To enhance the value of cellulose and expand its multifunctionality, researchers have investigated a series of chemical reactions targeting the three reactive hydroxyl groups (located at C2, C3, and C6 positions) on the cellulose glucose units, thereby developing various derivatives that are soluble in water and various organic solvents.^169^ This effectively overcomes the original nonmelting and insolubility issues of cellulose, thereby improving its processability. Esterification and etherification are the most common methods used to enhance the processability and functionality of cellulose, resulting in products known respectively as cellulose esters and cellulose ethers.^170,171^ Figure 12 summarizes the typical strategies for chemical modification of cellulose and molecular structures of resulting cellulose derivatives, which serve as key precursors for fabricating cellulose derivative films, one of the six major categories of LCFs discussed in this review. Understanding their structures and properties provides important context for the development and performance of this specific LCF type.

**Figure 12:**
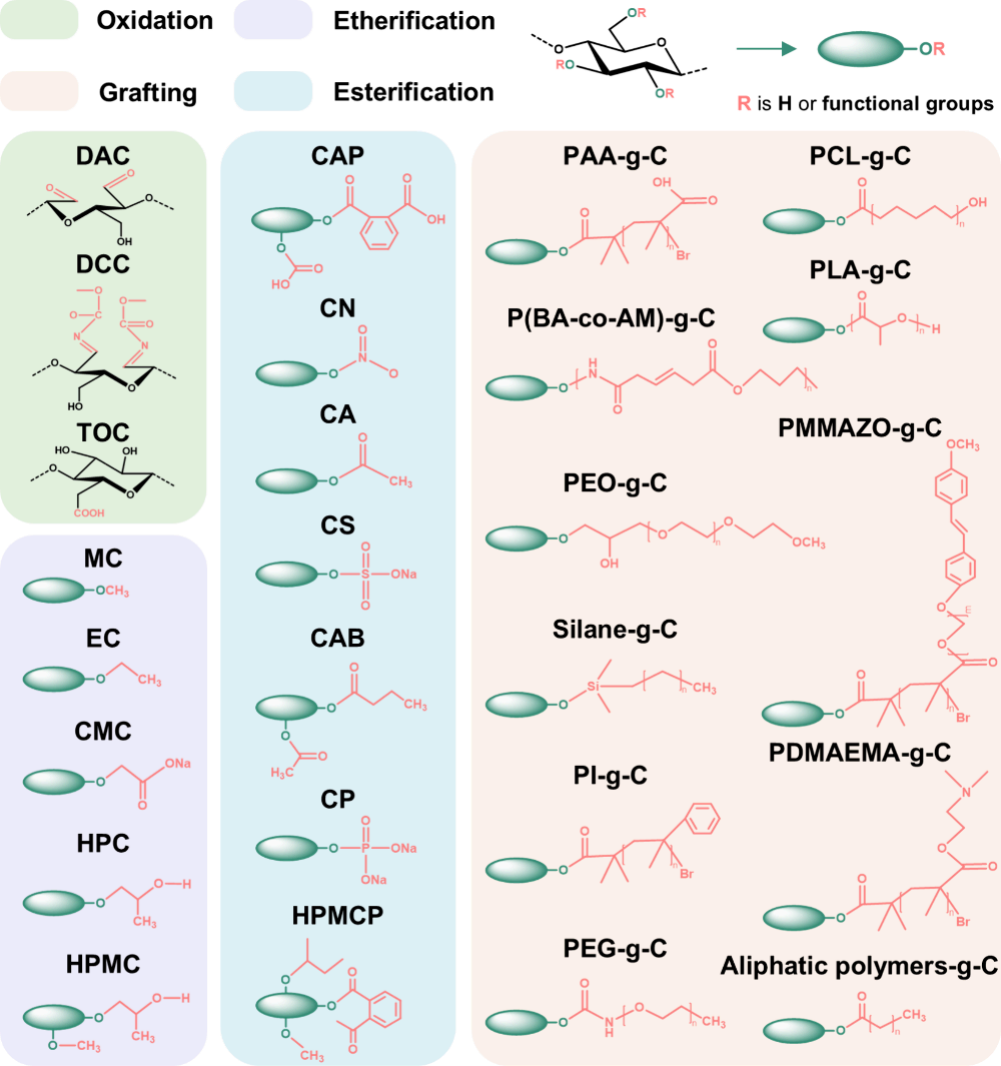
Most common modifications of cellulose and the chemical molecular formula of popular cellulose derivatives. The full names corresponding to the abbreviations in this figure are listed below: dialdehyde cellulose (DAC), dicarboxy cellulose (DCC), TEMPO oxidized cellulose (TOC), methyl cellulose (MC), ethyl cellulose (EC), carboxymethyl cellulose (CMC), hydroxypropyl cellulose (HPC), hydroxypropylmethyl cellulose (HPMC), cellulose acetate phthalate (CAP), cellulose nitrate (CN), CA, cellulose sulfate­(CS), cellulose acetate butyrate (CAB), cellulose phosphate (CP), hydroxypropylmethyl cellulose phthalate (HPMCP), grafted-cellulose (g-C), poly­(acrylic acid) (PAA), poly­(lactic acid) (PLA), poly­(*N*,*N*-dimethylaminoethyl methacrylate) (PDMAEMA), poly­(ethylene glycol) (PEG), poly­(ethylene oxide) (PEO), polyisoprene (PI), poly­(6-[4-(4-methoxyphenylazo) phenoxy]­hexyl methacrylate) (PMMAZO).

Cellulose esters are a category of thermoplastic biopolymers based on cellulose, with a long history of commercialization and widespread applications.^172^ The esterification of cellulose primarily involves the reaction of the hydroxyl groups on the cellulose molecular chains with acids, anhydrides, or acid chlorides. This process, catalyzed by acids, proceeds through dehydration condensation and can be conducted under homogeneous or heterogeneous conditions. Depending on the type of esterifying agent used, cellulose esters can be categorized into inorganic and organic cellulose esters.^173^ The former includes products of reactions between cellulose and inorganic acids such as sulfuric acid, nitric acid, and phosphoric acid, resulting in cellulose sulfate, CN, and CP, respectively. The latter encompasses products from reactions between cellulose and organic acids like formic acid, acetic acid, propionic acid, and higher fatty acids, resulting in cellulose formate, CA, cellulose propionate, and cellulose esters of higher fatty acids. Among these, CN is a typical example of an inorganic ester, while CA and its mixed esters (e.g., CA propionate and CA butyrate) are representative products of organic esters.^174^ Unlike cellulose, cellulose esters (e.g., CA) often possess relatively hydrophobic substituents, exhibit good solubility in organic solvents rather than water, and are thermoplastic, thus finding extensive use in everyday products such as plastics, coatings, separation membranes, and cigarette filters.^175^

Cellulose ethers are a series of derivatives formed by the partial or complete reaction of hydroxyl groups on cellulose molecular chains with etherifying agents (e.g., epoxides, halogenated alkanes, alkyl, silyl chlorides, bromides, or vinyl compounds) under alkaline conditions.^176^ These derivatives can be categorized into single ethers and mixed ethers based on the type of substituents. Single ethers include alkyl ethers (e.g., MC, EC, phenyl cellulose, and cyanoethyl cellulose), hydroxyalkyl ethers (e.g., hydroxymethyl cellulose and hydroxyethyl cellulose (HEC)), and carboxyalkyl ethers (e.g., CMC, carboxyethyl cellulose). Mixed ethers, such as hydroxypropyl methylcellulose, ethyl methylcellulose, and carboxymethyl hydroxyethylcellulose, are characterized by the substitution of the hydroxyl groups with two or more types of groups on the cellulose chain. Among those cellulose ether derivatives, products such as CMC, HEC, HPC, and HPMC are notable for their excellent thickening, emulsifying, suspending, film-forming, colloid-protective, moisture-retaining, and adhesive properties.^177^ These properties have led to their widespread application in fields such as food, pharmaceuticals, papermaking, coatings, building materials, oil field extraction, textiles, and electronic components.^178^

## 3 Preparation and Properties of Lignocellulosic Films

### 3.1 Categories of Lignocellulosic Films

Based on processing strategies and chemical compositions, LCFs can be categorized into cellulose derivative films, RC films, nanocellulose films, hemicellulose films, lignin films, and whole lignocellulosic biomass films. More details about each type of LCFs will be separately discussed in the following subsections.

#### 3.1.1 Cellulose Derivative Films

The preparation process for cellulose derivative films typically involves dissolution, film formation, and drying. First, cellulose derivatives are dissolved in suitable solvents to form a uniform solution. Common solvents include water, ethanol, acetone, and other organic solvents.^179^ The dissolution process requires careful control of the solution’s concentration and viscosity to ensure uniformity and quality in subsequent film formation. The dissolved cellulose derivative solution is then evenly coated onto a substrate using methods such as knife coating, spin coating, or just simply casting.^180–183^ The coated solution undergoes drying or curing to remove the solvent, forming a continuous film.

CA is one of the most common cellulose derivatives. Although it possesses inherent film-forming ability, film formation in practice often requires the use of plasticizers or elevated processing temperatures to achieve desirable flexibility and processability.^184^ CA films exhibit high chemical and mechanical stability, superior transport properties, low protein adsorption, excellent water affinity, excellent film-forming property, and ease of availability, making them ideal for gas separation, reverse osmosis (RO), nanofiltration, ultrafiltration, microfiltration, pervaporation, and ion exchange.^185,186^ For example, the incorporation of ZIF67 nanoparticles into CA films significantly improved their antibacterial, UV-shielding, and ammonia-responsive properties, demonstrating potential applications in extending food shelf life and monitoring meat freshness in real-time (Figure 13ai).^187^

**Figure 13:**
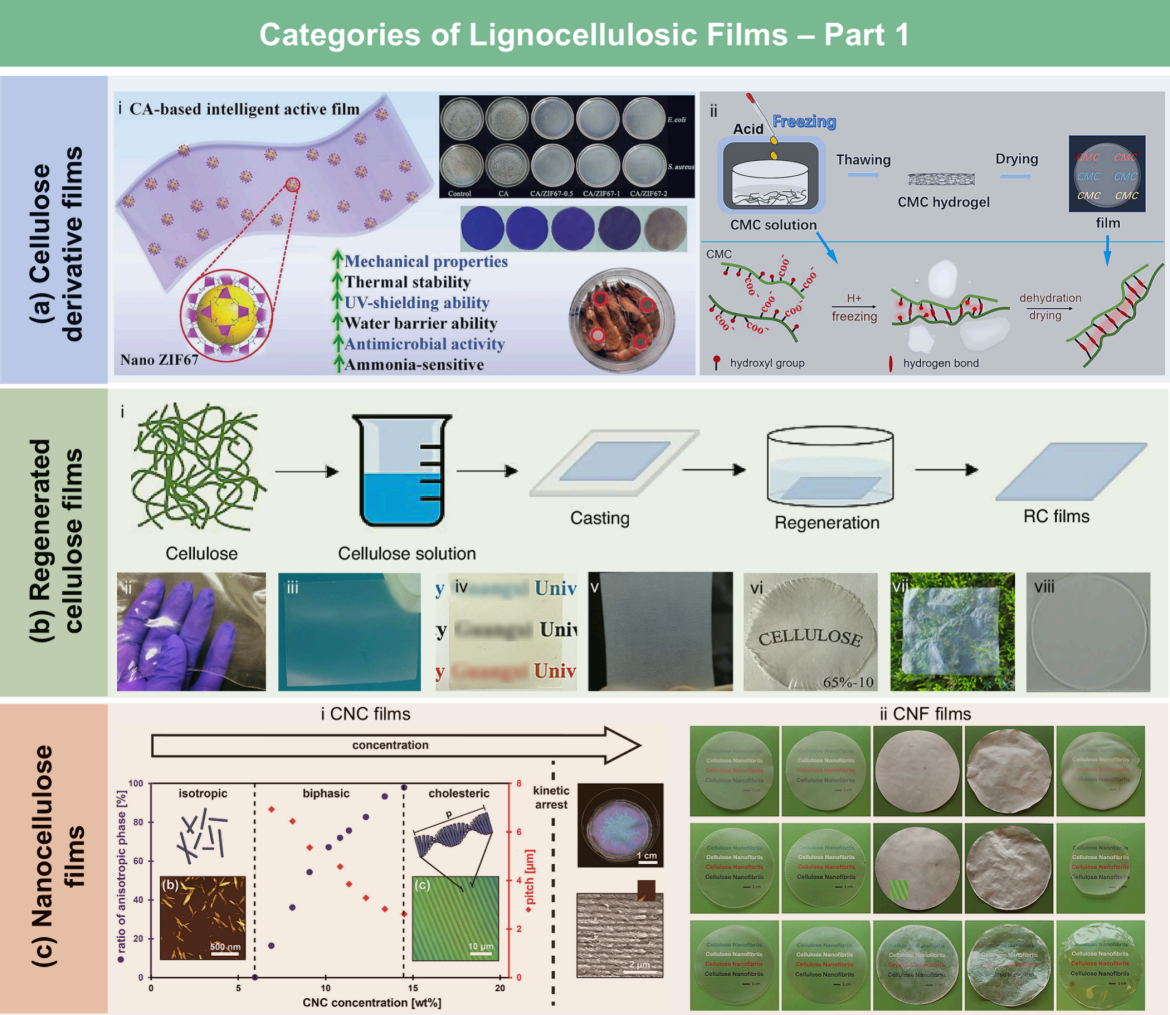
Categories of LCFs. (a) CA/ZIF67 intelligent active films for shrimp freshness monitoring (i). Adapted with permission from ref 221. Copyright 2022 Elsevier. Schematic showing the preparation of the CMC film (ii). Adapted with permission from ref 196. Copyright 2020 Elsevier. (b) Typical preparation methods for RC films (i). Adapted with permission from ref 222. Copyright 2021 Elsevier. Photos of films produced using various solvent systems (ii) LiCl/DMAc. Adapted with permission from ref 210. Copyright 2021 Springer Nature under CC BY 4.0 (https://creativecommons.org/licenses/by/4.0/). (iii) NaOH/urea. Adapted with permission from ref 223. Copyright 2020 Elsevier. (iv) AmimCl. Adapted with permission from ref 224. Copyright 2020 American Chemical Society. (v) BmimCl. Adapted with permission from ref 225. Copyright 2020 Elsevier. (vi) LiBr molten salt hydrate. Adapted with permission from ref 24. Copyright 2019 MDPI under CC BY 4.0 (https://creativecommons.org/licenses/by/4.0/). (vii) NMMO. Adapted with permission from ref 226. Copyright 2023 Elsevier. (viii) TBPH, adapted with permission from ref 227. Copyright 2020 Springer Nature under CC BY 4.0 (https://creativecommons.org/licenses/by/4.0/). (c) Schematic of the self-assembly of a CNC suspension upon evaporation to form a structurally colored film (i). Adapted with permission from ref 228. Copyright 2017 Wiley-VCH under CC BY 4.0 (https://creativecommons.org/licenses/by/4.0/). Photographs of different CNF films (ii). Adapted with permission from ref 229. Copyright 2015 Springer Nature.

Nitrocellulose is another cellulose derivative capable of independent film formation. Depending on its degree of substitution, nitrocellulose can be highly flammable and potentially hazardous, requiring careful handling and storage. For instance, it was originally used in the production of smokeless gunpowder and solid propellants.^188^ Currently, nitrocellulose is widely used in spray paint, artificial leather, ink, and other fields. Additionally, nitrocellulose membranes have good potential in products such as portable diagnostic biosensors and wearable sensors. Nitrocellulose membranes are generally prepared by phase inversion, spin coating, or electrospinning techniques.^189,190^ Compared with traditional filter paper and chromatography paper that require pretreatment, the nitrate groups on the surface of nitrocellulose have strong dipoles and can directly and efficiently immobilize biomolecules.^191^ Therefore, it can be used in many fields such as disease diagnosis, environmental testing, and food safety assessment.^192^

Cellulose acetate butyrate (CAB), due to the presence of acetyl and butyryl groups in its molecular structure, exhibits hydrophobic properties and demonstrates several advantageous characteristics for films, including good chlorine resistance, oxidation resistance, excellent chemical stability, biocompatibility, and toughness.^193^ CAB can form stable films without plasticizers and is widely used in coatings, plastics, and eyewear manufacturing, especially where enhanced appearance and durability are required. These films perform well in outdoor applications and maintain their properties and appearance over long-term use. However, it is worth noting that CAB may produce undesirable odors due to the hydrolysis or thermal decomposition of acetyl and butyryl groups, which can be a concern in consumer-facing and medical applications.

HPMC is another widely studied cellulose derivative capable of independently forming transparent, mechanically strong films. HPMC films are widely used in pharmaceutical applications, such as drug coatings and vegetarian capsules, due to their excellent biocompatibility and enzyme resistance.^194^ While HPMC has also been explored for food packaging, its practical use in this area is primarily as a component of composite materials rather than as a standalone film. In food preservation applications, it is often combined with antioxidants or antimicrobials to enhance functionality.^195^

Other cellulose derivatives, such as CMC, MC, and HEC, also possess film-forming abilities but they generally exhibit lower mechanical strength. For example, Wang et al.^196^ developed a green method to fabricate water-insoluble transparent films from CMC via an acid-assisted freeze–thaw process and drying, achieving films with high transparency, excellent mechanical properties, water resistance, and thermal stability (Figure 13aii). To improve the performance of these cellulose derivative films, chemical modifications or blending with other polymers can be employed. Introducing plasticizers, cross-linking agents, or blending with hydrophilic polymers (e.g., poly­(vinyl alcohol) (PVA), gelatin, and chitosan) can adjust the films’ mechanical properties, thermal stability, and barrier performance.^197–200^

#### 3.1.2 Regenerated Cellulose Films

RC films are produced by dissolving natural cellulose and reforming it into films (Figure 13bi). Selecting an appropriate solvent system is crucial in the preparation of RC films. Cellulose solvent systems can be classified into derivatizing and nonderivatizing systems, each with unique advantages and characteristics for the RC films produced. Figure 13b­(ii–viii) show RC films prepared in different solvent systems.

Derivatizing solvent systems dissolve cellulose by chemically converting it into its derivatives, often involving complex chemical processes and potential byproducts. One of the earliest derivatizing systems, commercialized in the early 20th century, is the sodium hydroxide and carbon disulfide system, where cellulose reacts with carbon disulfide to form cellulose xanthate.^201^ This viscose solution is mainly used to produce viscose fibers (rayon) and films (cellophane). Although the viscose process has historically raised concerns due to the use of carbon disulfide, a flammable and toxic compound, modern implementations in developed countries have improved its environmental profile through closed-loop recovery systems.^202^ At high temperatures, urea reacts with cellulose to form cellulose carbamate intermediates, which can be dissolved in sodium hydroxide solution and regenerated into cellulose films using sodium sulfate or sulfuric acid as coagulants.^203^ These films have similar properties to cellophane, but the process remains complex and is still largely at the research stage due to challenges in scalability and environmental impact. Proton acid systems like anhydrous phosphoric acid can rapidly dissolve cellulose to form uniform solutions. This method can produce high-performance RC films, but the recovery and handling of phosphoric acid remain difficult, and the process has not yet reached commercial maturity.

Nonderivatizing solvent systems dissolve cellulose through physical interactions without altering its chemical structure, making them more environmentally friendly and easier to handle. *N*-methylmorpholine-*N*-oxide (NMMO) is one such nonderivatizing solvent, where dissolution occurs entirely through physical means. The active N–O dipole and oxygen groups in NMMO form hydrogen bonds with the anhydroglucose units of cellulose, disrupting the intermolecular hydrogen bonds and enabling dissolution.^204^ The NMMO process has been successfully scaled up for industrial production of regenerated fibers and films, commonly known as Lyocell.^205^ RC films produced from NMMO solution exhibit good mechanical properties, high transparency, as well as good thermal stability and biodegradability. Additionally, these films show excellent gas barrier properties, making them suitable for food packaging and medical applications.^206^ However, it is important to note that anhydrous NMMO poses significant safety risks, including thermal instability and the potential for explosive decomposition. Strict temperature control and the use of stabilizers such as butylated hydroxytoluene are essential during processing. Although the recovery rate of NMMO solvent can be achieved as high as 99.5%, the recovery process is complicated, energy-consuming, and costly.^207^

The lithium chloride/*N*,*N*-dimethylacetamide (LiCl/DMAc) system is another nonderivatizing cellulose solvent. LiCl forms a complex with DMAc that disrupts the hydrogen bonding network between cellulose chains, enabling dissolution.^208^ RC films produced using the LiCl/DMAc system exhibit good transparency and flexibility, with reported tensile strengths reaching up to 120 MPa under optimized conditions.^209^ However, this value represents high-performance or “champion” data and may not reflect typical outcomes. Additionally, these films have demonstrated promising stability alkali and acidic solutions, with potential for future applications in electronic displays and optical devices.^210^ Nonetheless, the toxicity, cost, and safety concerns associated with the LiCl/DMAc system currently limit its use to research settings rather than commercial-scale production.

Ionic liquids (ILs) are a class of organic salts with low melting points, exhibiting excellent solubility for cellulose.^211^ Common ionic liquids include 1-allyl-3-methylimidazolium (AMIM) and 1-butyl-3-methylimidazolium (BMIM).^212^ These ionic liquids disrupt the hydrogen bonds between cellulose molecules through the polarity of their cations and anions, leading to the dissolution of cellulose.^213^ RC films prepared using ionic liquids demonstrate excellent mechanical properties and thermal stability, with a tensile strength of up to 170 MPa for the optimized sample.^214^ However, the corrosiveness of ionic liquids to equipment and challenges in solvent recovery need to be addressed.

The alkaline/urea aqueous solution system has been proven to be an effective cellulose solvent. In this system, alkali and urea form hydrogen bond complexes, which attract the hydroxyl groups of cellulose chains to form new hydrogen bonds at low temperatures, destroying the original intramolecular and intermolecular hydrogen bonds among cellulose chains.^215,216^ RC films produced using the alkaline/urea system exhibit good mechanical properties and biodegradability, with a tensile strength of up to 100 MPa.^17^ Compared to other solvents, the alkaline/urea system has the advantages of relatively low toxicity and cost, making it a “green” solvent. However, further improvement in solubility and solvent recovery efficiency is needed for industrial applications.

Molten salt hydrate (MSH) systems are an emerging cellulose solvent system known for their efficiency, cost-effectiveness, and environmental friendliness.^217^ For example, the LiBr·3H_2_O system effectively breaks the hydrogen bonds between cellulose molecules by forming strong ionic interactions and hydrogen bonds with LiBr and water.^218^ Specifically, Li^+^ ions bind with hydroxyl oxygen atoms in cellulose molecules, while Br^–^ ions bind with hydrogen atoms in hydroxyl groups, preventing hydrogen bond reformation.^219^ RC films prepared using the LiBr·3H_2_O system exhibit excellent transparency and mechanical properties, with a tensile strength of up to 90 MPa under the optimized reaction conditions.^220^ Additionally, this system has high solvent recovery efficiency and is environmentally friendly, showing great industrial application prospects. However, issues such as temperature control during dissolution and solvent recovery need to be resolved for practical applications.

In summary, RC films can be prepared using various solvent systems, each with its advantages. Derivatizing solvent systems effectively dissolve cellulose but often involve complex chemical reactions and environmental issues. Nonderivatizing solvent systems such as NMMO, LiCl/DMAc, ionic liquids, alkaline/urea, and molten salt hydrates offer significant advantages in terms of environmental friendliness and operational simplicity. Future research will further optimize these solvent systems to improve dissolution efficiency and solvent recovery rates, promoting the widespread industrial application of RC films.

#### 3.1.3 Nanocellulose Films

Nanocellulose film, also called cellulose nanopaper (CNP), is usually prepared from nanocellulose suspension by a self-assembly process in which the solvent is removed and the nanocellulose finally forms the CNP.^230,231^ Casting and filtration are two popular approaches for CNP production.^229^ Casting is a simple but time-consuming process, by which nanocellulose suspension is usually kept in an open Petri dish, and the solvent is allowed to evaporate, finally leaving the self-assembled CNP.^232^ Normally, a slow drying rate is necessary to reduce wrinkling problems and make flat, and uniform CNP.^233^ This process usually takes several days to obtain the final CNP. Filtration, which is similar to a papermaking process, is a relatively fast method to prepare CNP.^234^ Typically, the filtration process involves two separate operations: (a) filtration of nanocellulose suspension under vacuum or pressure and (b) drying (e.g., air-drying, oven-drying, hot-pressing) of thus obtained nanocellulose gels.^235,236^ The total time of the filtration process is reported to be a few hours instead of days, which is much faster than the casting method. Recent works have shown that CNP is a promising material for emerging applications in photovoltaic cells,^237^ as visual display substrate,^238^ energy storage electrode materials,^239^ etc., in light of its good thermal stability, low coefficient of thermal expansion (CTE) (<10 ppm/K), and tunable optical properties besides biodegradability and superior mechanical properties.^240,241^

When compared to CNFs, the self-assembled CNP of CNCs is generally more brittle due to the lack of an energy-dissipating amorphous phase and its inability to form entangled networks.^230^ To address this issue, CNC films are often combined with other polymeric materials such as poly­(ethylene glycol) and poly­(vinylpyrrolidone) for better flexibility and practical applications.^242^ It is worth noting that aqueous suspensions of surface functionalized CNCs (e.g., with sulfate half-ester groups) can arrange themselves into a left-handed chiral nematic lyotropic liquid crystalline phase.^243^ This structure can be preserved as an iridescent CNP (Figure 13ci) when the solvent evaporates, resulting in a vivid structural color induced by coherent scattering and interference of light, with the color varying with the length of the chiral nematic helical pitch.^244^ Unlike traditional chromophores in pigments or dyes, this structural color is easily adjustable and does not fade, making it advantageous for the development of innovative devices, optical displays, decorative applications, anticounterfeiting markers, and biosensors.^245,246^

Compared to CNC films, CNF films (Figure 13cii) are more flexible and can withstand greater deformation without breaking due to CNFs’ higher aspect ratio and semicrystalline structure.^247^ This makes CNF films advantageous in applications requiring high flexibility and strength, such as flexible electronic devices and high-performance packaging materials. Additionally, CNF films exhibit excellent thermal and chemical stability. Studies have shown that CNF films can maintain their structure and properties at high temperatures (150–200 °C) without significant thermal degradation.^248^ CNF films have an exceptionally low thermal expansion coefficient (less than 10 ppm/K), which is lower than that of glass (50 ppm/K) and significantly lower than that of most moldable plastics (approximately 200 ppm/K).^249^ Moreover, CNF films can resist various chemical reagents (e.g., methanol, acetone, and toluene), maintaining their physical and chemical properties,^250^ thus making them advantageous for applications requiring chemical resistance, such as protective coatings and chemical sensors.^14^

In brief, CNC and CNF films demonstrate outstanding properties and hold significant promise for a range of advanced applications. However, many of these uses remain in the research or early development stage. Moreover, it is important to note that the commercialization and industrial-scale application of nanocellulose films remain limited. Key challenges include high production costs, limited scalability of fabrication techniques, equipment requirements, and sensitivity to environmental factors such as humidity, which can affect storage and long-term performance. Continued efforts are needed to overcome technical and economic barriers before these materials can be widely adopted in commercial products.

#### 3.1.4 Hemicellulose Films

Since Smart and Whistler first reported hemicellulose acetate films in 1949,^251^ hemicellulose films have garnered significant attention in the fields of functional packaging films, edible films, and drug delivery films due to their renewable sources, environmental friendliness, biodegradability, and biocompatibility.^252^ Researchers have continually explored efficient extraction of hemicellulose from various biomasses, such as bagasse, Norway spruce, cotton straw, oil palm leaves, and barley bran.^253^ They have also studied the film-forming properties of different types of hemicellulose (e.g., arabinoxylan, glucomannan) and their impact on the mechanical properties of the films to achieve optimal hemicellulose materials. Mendes et al.^254^ developed biodegradable hemicellulose films from galactomannan (*C. pulcherrima*) and xyloglucan (*T. indica*), characterized by good transparency, thermal stability, and moisture barrier properties, suitable for food packaging applications (Figure 14ai). They showed that blending these hemicelluloses offers improved film properties compared to individual components. Studies have shown that the tensile strength and elongation of various hemicellulose films differ significantly, indicating that the biomass source greatly influences the mechanical properties of the hemicellulose films, even when the extraction methods are consistent.^255^ This variability is likely due to differences in the types and structures of hemicellulose present in different biomass, including variations in monomer composition, molecular weight, and degree of branching, which affect the density of molecular packing and hydrogen-bonding networks within the films. Additionally, differences in the polysaccharide components of hemicellulose from the same biomass source can also impact the mechanical and oxygen barrier properties of the films.^256^

**Figure 14:**
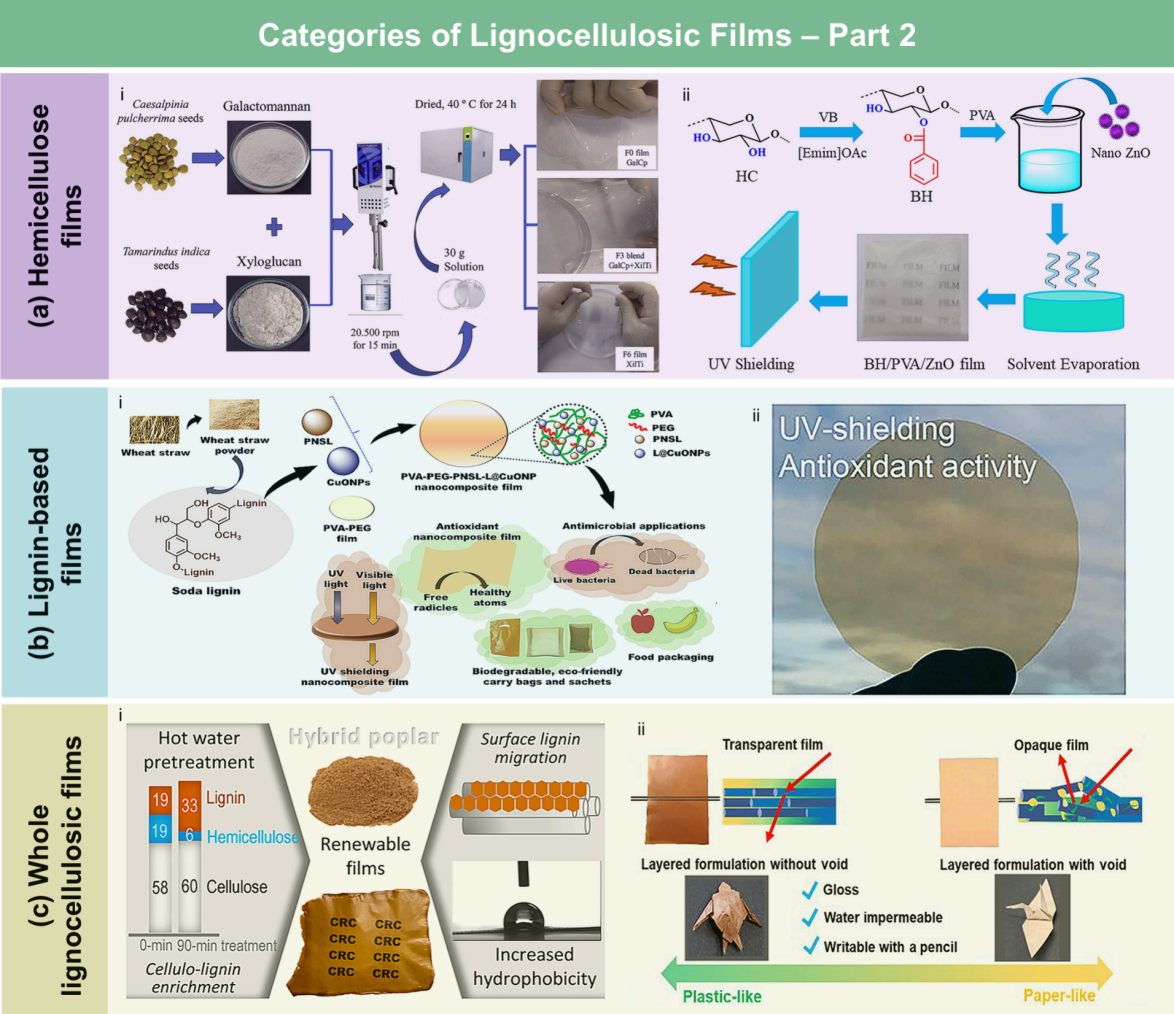
(a) Preparation process of galactomannan (GalCp) films, xyloglucan (XilTi) films, and their composite films (i). Adapted with permission from ref 273, Copyright 2017 Elsevier. Benzoated hemicellulose/PVA/ZnO films for UV-shielding (ii). Adapted with permission from ref 259. Copyright 2019 Springer Nature. (b) PVA-PEG-PNSL-L@CuONP nanocomposite films as advanced UV protective and antimicrobial sustainable packaging materials (i). Adapted with permission from ref 265 Copyright 2024 Elsevier. Lignin nanoparticles/PVA composite film with UV-shielding and antioxidant activity (ii). Adapted with permission from ref 274. Copyright 2017 Springer Nature under CC BY 4.0 (https://creativecommons.org/licenses/by/4.0/). (c) Ionic-liquid-assisted fabrication of lignocellulosic thin films with tunable hydrophobicity (i). Adapted with permission from ref 275. Copyright 2022 American Chemical Society. The transparent and opaque wood films with paper- and plastic-like properties were produced upon solvent removal (ii). Adapted with permission from ref 276. Copyright 2024 Royal Society of Chemistry under CC BY 3.0 (https://creativecommons.org/licenses/by/3.0/).

Despite their excellent properties in some areas, films prepared from isolated hemicellulose often exhibit poor flexibility due to the rigidity of the hemicellulose molecules, low chain flexibility, and strong intermolecular hydrogen bonding, making film formation challenging.^257^ Furthermore, such hemicellulose films show poor compatibility with traditional plastics and low thermal stability. To overcome these drawbacks, modification of hemicellulose films is necessary.^258^ Common modification methods include physical and chemical modifications. Physical modification often involves adding small molecule plasticizers, such as glycerol, sorbitol, and xylitol, to improve flexibility. Blending hemicellulose with various reinforcing agents, which interact with hemicellulose hydroxyl groups through hydrogen bonding, can create polymer systems with improved mechanical properties. Common reinforcing agents include inorganic materials like montmorillonite, bentonite, and nanosilica, and organic polymers like starch, CNCs, CMC, chitosan (CS), and PVA. For example, Zhang et al.^259^ synthesized hemicellulose-based functional films by chemically modifying hemicellulose with vinyl benzoate and reinforcing it with PVA and nano-ZnO. The films exhibited excellent flexibility, UV-shielding properties (UV-A blocking 99.34%, UV-B blocking 99.99%), moderate tensile strength, and low permeability, highlighting their potential for sustainable packaging applications (Figure 14aii).

In practical applications, hemicellulose films have been investigated for use in food packaging and medical dressings due to their eco-friendliness and biodegradability.^22,260^ Although their moisture sensitivity limits their use in certain applications, appropriate chemical modifications (e.g., esterification, etherification, and graft copolymerization) can alleviate these limitations.^261^ For instance, benzylated glucomannan films demonstrate excellent oxygen barrier properties in high-humidity environments, making them a potential choice for food packaging materials. In conclusion, hemicellulose films are a promising eco-friendly material with broad application prospects, despite some performance limitations. Reasonable modification treatments can significantly expand their application potential.

#### 3.1.5 Lignin-Based Films

Generally, long-chain molecules with highly branched or regular structures exhibit good film-forming properties.^262^ In contrast, lignin exhibits a highly irregular, heterogeneous, and polyaromatic structure. Depending on the source and extraction process (e.g., kraft, soda, or organosolv), lignin differs significantly in terms of functional group content, molecular weight, and polydispersity.^263^ Lignin’s complex and rigid molecular architecture, along with its limited chain entanglement, results in films with poor mechanical properties, such as high brittleness and insufficient strength and ductility.^264^ Additionally, lignin’s thermophysical characteristics, such as relatively high glass transition temperatures (*T*_g_) and lack of melting behavior, further complicate direct film processing. Consequently, most current research focuses on blending lignin with other materials, such as starch, cellulose, polypropylene (PP), PVA, and CS, to form lignin-based composite films. For instance, Kirar et al.^265^ synthesized eco-friendly nanocomposite films using soda-extracted lignin nanoparticles (PNSL) and lignin-derived copper oxide nanoparticles (L@CuONPs), blended with PVA and PEG (Figure 14bi). These films exhibited high UV protection, antimicrobial activity (99.999% reduction in pathogens), and flexibility, suggesting potential applications in sustainable food packaging. Tian et al.^266^ developed lignin nanoparticles (LNPs) from technical lignins via DES and ethanol organosolv methods (Figure 14bii). These LNPs, incorporated into PVA, formed nanocomposite films with excellent UV-shielding (∼100% at 400 nm with 4 wt % LNPs), antioxidant properties, and improved mechanical performance (tensile strength ∼60 MPa). The work highlights LNPs as a sustainable alternative to inorganic additives in advanced packaging. Due to the abundance of active functional groups in lignin molecules, it can readily interact with various polymers at the interface, enhancing compatibility and resulting in more uniform composite films.^267^ These film materials are not only cost-effective but also versatile, making them suitable for a wide range of industrial production processes.

Due to its natural UV-absorbing chromophores, lignin has shown promise as a functional additive in films intended for UV shielding, such as packaging for light-sensitive products and optical applications.^264^ Additionally, lignin-based films exhibit outstanding antioxidant properties, which can extend the shelf life of food in packaging applications while their biodegradability reduces plastic pollution, enhancing their environmental benefits (Figure 14b).^268^ Due to lignin’s excellent electrical insulation properties, lignin-based films have been used in electronic devices as insulating layers to protect circuits and prevent short circuits and other electrical failures.^269^ In the biomedical field, lignin-based films are being explored for wound dressings and drug delivery carriers, due to their potential biocompatibility and antibacterial activity. Moreover, through chemical modification and physical blending with other active materials, lignin-based films can be further endowed with various functionalities, such as conductivity and photosensitivity, making them promising candidates in high-value functional materials.^270,271^ In addition, the good adhesive properties of lignin make it suitable for coatings and adhesive materials, widely used in wood protection, paints, and glues.^272^

#### 3.1.6 Whole Lignocellulosic Biomass Films

Whole lignocellulosic biomass films, which integrate cellulose, hemicellulose, and lignin, exhibit notable comprehensive properties, making them superior to single-component films in various applications.^277^ Similar to the plant cell wall, within whole lignocellulosic biomass films cellulose provides strong structural support and mechanical strength, hemicellulose enhances flexibility, and lignin offers good water resistance, UV protection, and thermal stability.^278^ This synergistic effect results in a well-balanced and comprehensive performance, better meeting diverse application demands. In addition, extra costs and chemical utilization caused by delignification and cellulose modification could be significantly minimized by directly preparing films from whole lignocellulosic biomass. However, this is quite challenging because the low compatibility (poor interfacial adhesion) between lignin and cellulose often leads to phase separation between cellulose-rich and lignin-rich domains, resulting in structural discontinuities. These morphological irregularities can compromise mechanical integrity and optical uniformity.^279,280^

Currently, there are two main types of processing strategies for making whole lignocellulosic biomass films: direct dissolution and regeneration, and deconstruction and reassembly. For the first method, studies have shown some specific solvent systems that can directly dissolve lignocellulosic biomass to make whole lignocellulosic biomass films. For example, ILs like 1-ethyl-3-methylimidazolium chloride (EMIMCl) and 1-butyl-3-methylimidazolium chloride (BMIMCl) are highly effective in dissolving lignocellulose components.^281^ Rajan et al.^275^ demonstrated the preparation of whole biomass films from poplar wood using 1-ethyl-3-methylimidazolium acetate [EMIM]­[OAc] (Figure 14ci). The obtained films show a mechanical strength in the range of 32–40 MPa, but with a low elongation (2.3–5.8%). Chen et al.^282^ directly prepared lignocellulosic biomass films from bagasse using the DMSO/LiCl system. The mechanical strength of the film is 38.3, with a mechanical strain of only 4.4%. Kobayashi et al.^276^ introduced a low-energy method to dissolve wood sawdust in formic acid under mild conditions (40–50 °C), enabling the formylation of lignin, hemicellulose, and cellulose (Figure 14cii). This process resulted in reassembled films with plastic-like properties (e.g., tensile strength of 61 MPa for Eucalyptus films) or paper-like properties (e.g., Japanese cedar films), offering potential as sustainable alternatives to petroleum-derived plastics.

The second method, deconstruction and reassembly, involves deconstructing lignocellulosic biomass into cellulose, hemicellulose, and lignin, followed by reassembling these components into films.^283^ This strategy allows more precise control over component ratios, interfacial interactions, and microstructure, which can be tailored for specific performance targets. Component separation is often necessary to overcome intrinsic incompatibilities and to allow for chemical or physical modifications before recombination. During reassembly, molecular interactions, particularly hydrogen bonding and van der Waals forces, are re-engineered to promote network uniformity, mechanical cohesion, and multifunctionality. Deconstruction processes include mechanical, chemical, or enzymatic treatments, while reassembly methods vary, including solution casting, electrospinning, and coblending extrusion. For example, Sadeghifar et al.^284^ used click chemistry to prepare cellulose-lignin films with covalent bonding, achieving 100% UV-B and over 90% UV-A shielding. These transparent films also maintained good mechanical properties (∼90 MPa tensile strength) and stability under UV and thermal conditions. Ou et al.^285^ developed composite films by mixing phenolated lignin nanoparticles (LNPs) with CNFs. They demonstrated that the hydrogen bonding between phenolic hydroxyl groups on lignin and hydroxyl groups on cellulose significantly enhanced the films’ mechanical strength and toughness. The reported tensile strength reached up to 190 MPa, which represents an exceptional case among cellulose–lignin systems and highlights the potential of optimized interfacial design. The size, morphology, and chemical structure of cellulose, hemicellulose, and lignin significantly influence the final film’s performance. Cellulose size and crystallinity determine the film’s mechanical strength and thermal stability. Smaller nanocellulose enhances the film’s transparency and strength, while larger cellulose microfibers provide greater flexibility. The molecular weight and structure of hemicellulose affect the film’s flexibility and extensibility.^286^ Lignin content and structure impact the film’s hydrophobicity, UV resistance, and thermal stability.

In short, the preparation methods and solvent systems for whole lignocellulosic biomass films significantly affect their performance. By focusing on rational interface design, component compatibility, and processing scalability, it is possible to produce whole lignocellulosic biomass films with tailored properties to meet various application needs. Future research should aim to deepen the mechanistic understanding of film formation, while also developing more efficient, economical, and environmentally friendly preparation methods to enable broader commercial adoption.

### 3.2 Preparation Methods of Lignocellulosic Films

Various processes have been employed to prepare LCFs for different applications, ranging from packaging to sensors. This section will explore several key methods (summarized in Figures 15 and 16) used to produce LCFs, including casting, filtration, coating, regeneration, electrospinning, and other emerging techniques, highlighting their advantages, limitations, and suitability for specific applications.

**Figure 15:**
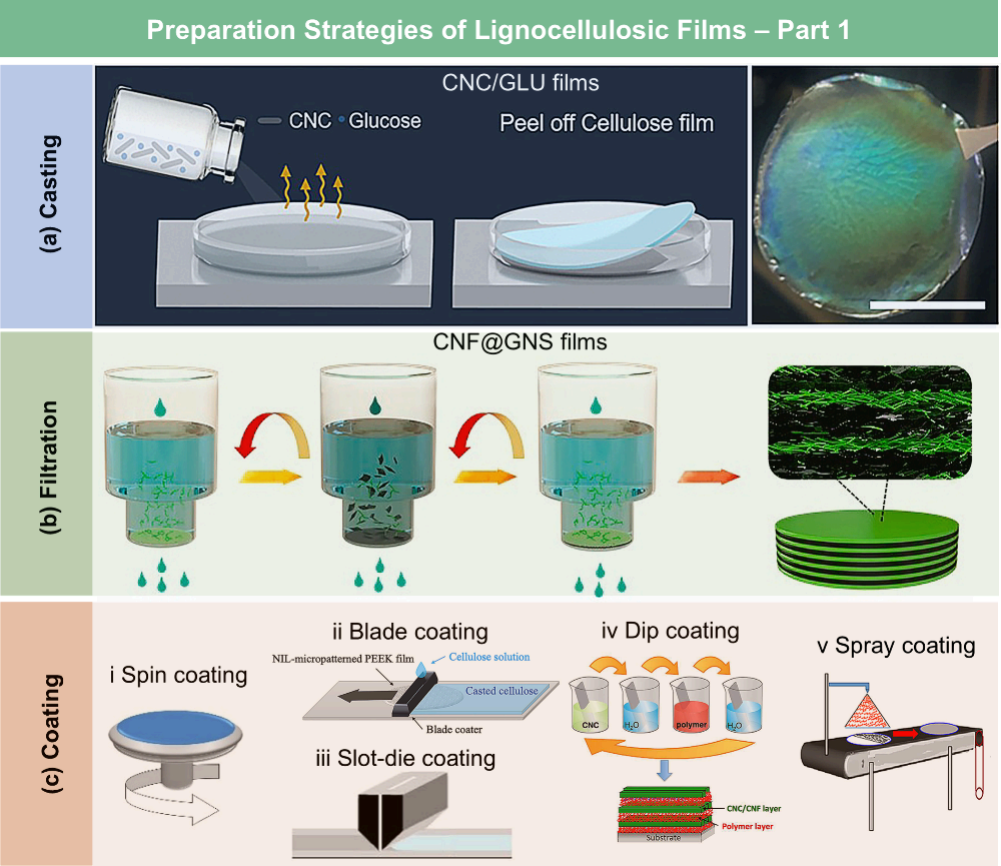
Preparation strategies for LCFs. (a) Schematic diagram of the production process of self-assembled CNC ColPRC film using the casting method and the prepared CNC/GLU film. Adapted with permission from ref 288. Copyright 2022 American Chemical Society. (b) Schematic diagram of the preparation of multilayer CNF@GNS films by the AVF process. Adapted with permission from ref 295. Copyright 2020 Elsevier. (c) Schematic diagram of CNC/PLA film formation in spin-coating method (i). Adapted with permission from ref 309. Copyright 2020 Elsevier. Casting one layer of cellulose solution using a blade coater on top of an NILmicro-patterned PEEK substrate (ii). Adapted with permission from ref 310, Copyright 2022 Elsevier under CC BY 4.0 (https://creativecommons.org/licenses/by/4.0/). Schematic diagram of slot-die preparation of CNC film (iii). Adapted with permission from ref 311. Copyright 2021 Springer Nature. Layer-by-layer assembly of (nanocellulose/polymer) thin films by dip-coating (iv). Adapted with permission from ref 312. Copyright 2025 Royal Society of Chemistry under a Creative Commons Attribution-NonCommercial-ShareAlike 3.0 Unported License. Schematic diagram of the experimental setup of the laboratory-scale spray system for the preparation of nanocellulose films (v). Adapted with permission from ref 313. Copyright 2017 Springer Nature.

**Figure 16:**
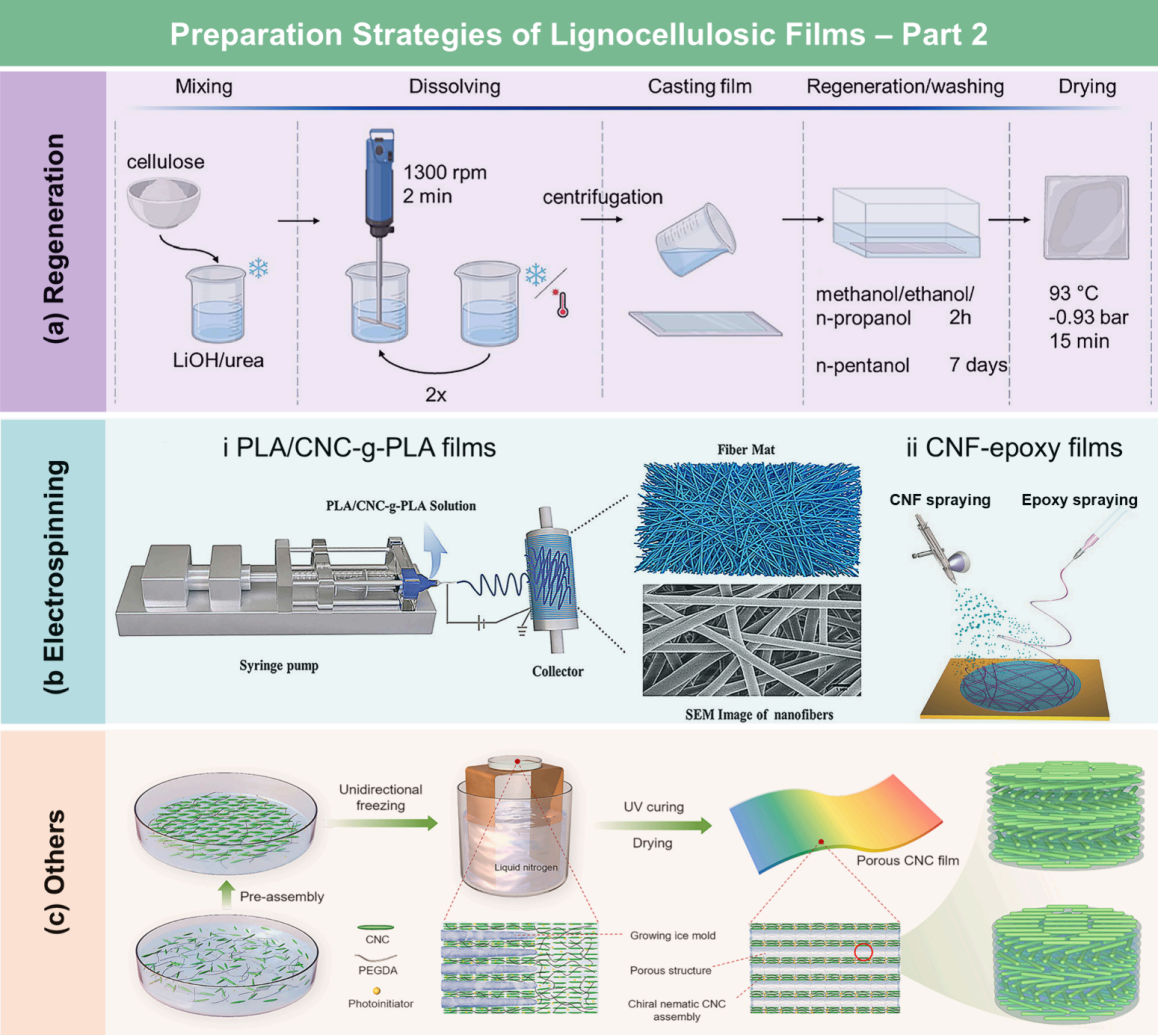
(a) Schematic illustration of the steps involved in the dissolution and regeneration of cellulose fibers to produce RC films. Adapted with permission from ref 329, Copyright 2024 Springer Nature under CC BY 4.0 (https://creativecommons.org/licenses/by/4.0/). (b) Schematic diagram of electrospinning to prepare PLA/CNC-*g*-PLA films (i). Adapted with permission from ref 339. Copyright 2023 Taylor and Francis. Schematic diagram of electrospinning to prepare CNF-epoxy hybrid film (ii). Adapted with permission from ref 340, Copyright 2016 Springer Nature under CC BY 4.0 (https://creativecommons.org/licenses/by/4.0/). (c) Fabrication scheme of porous CNC photonic film by unidirectional interlayer freezing-photo polymerization method. Adapted with permission from ref 341, Copyright 2024 Elsevier.

#### 3.2.1 Casting Method

The casting method is one of the most widely used and straightforward techniques for the preparation of LCFs. It involves dissolving or dispersing lignocellulosic materials in a solvent or aqueous medium to form a homogeneous solution or suspension.^260^ This solution is then poured into a mold or onto a flat substrate and left to dry, resulting in the formation of a thin, uniform film.^287^ The simplicity and scalability of the casting method make it a popular choice for producing films with a wide range of properties, depending on the material and drying conditions used. As shown in Figure 15a, casting enables the formation of diverse film morphologies and functionalities through simple solution-based processing.^288^ This versatility highlights the method’s potential for achieving structurally and functionally tailored LCFs under ambient or low-energy conditions.

A key factor in the casting method is the choice of solvent or dispersion medium. Nanocellulose, for instance, can form stable water suspensions without needing dissolution, while cellulose and lignin require specialized solvents like ionic liquids to break their hydrogen bonds and fully dissolve.^281,289^ Proper dispersion or dissolution is crucial to avoid issues like aggregation or incomplete mixing, which can affect the film’s mechanical properties and optical clarity.^290^ Thus, selecting the appropriate solvent or medium is essential for achieving strong, transparent, and uniform films. The drying process also has a significant impact on the final properties of LCFs. As the solvent evaporates, the material concentrates and forms a solid network. Drying conditions (e.g., temperature, humidity, and evaporation rate) determine film thickness, porosity, and surface smoothness.^291^ Slow drying under controlled conditions produces more homogeneous films with fewer defects, while rapid drying can lead to uneven surfaces or cracks.^292^ The casting method also offers easy control over film thickness by adjusting the solution concentration or volume, though uniformity can be challenging when scaling up for larger surfaces.

The casting method offers several advantages, including simplicity, cost-effectiveness, and scalability, making it suitable for both lab and industrial production. It allows for precise control over film properties by adjusting solution concentration, solvent composition, and drying parameters. However, limitations include long drying times, which can be impractical for high-throughput production, and the need to carefully control solvent evaporation to prevent defects like cracking or warping, especially in thicker films. Solvent residues may also affect the mechanical and thermal properties of the films, and the use of certain solvents, such as ionic liquids or DMAc/LiCl, poses environmental concerns due to toxicity or recycling difficulties.^293^ Despite these challenges, research continues to optimize the process by exploring water-based dispersions, biodegradable solvents, and hybrid techniques like freeze-drying to improve film properties. Functional additives, such as plasticizers, cross-linkers, or nanoparticles, can further enhance the mechanical strength, flexibility, and functionality of LCFs, broadening their application potential.

#### 3.2.2 Filtration

Filtration is a pressure-assisted technique widely applied in LCF fabrication, particularly effective for nanocellulose suspensions. It relies on the retention of solid components on a porous membrane during liquid removal, typically driven by vacuum or pressure. Key processing variables such as suspension concentration, membrane pore size, pressure, and filtration rate can significantly influence the resulting film’s thickness, uniformity, and microstructure. This process involves filtering a suspension of lignocellulosic material through a membrane, typically under vacuum or pressure.^294^ The solvent, usually water, is removed while the lignocellulosic material is retained on the membrane surface, forming a dense, uniform film. Due to the nanoscale dimensions and strong interfibrillar interactions of nanocellulose, this method is particularly effective for producing mechanically robust and compact films. Material-specific approaches and composite strategies are typically applied after mastering these fundamentals. For example, Li et al.^295^ fabricated flexible multilayered cellulose nanofiber/graphene films using an alternating vacuum filtration (AVF) method. This process involved alternately filtering CNF solutions and CNF/graphene nanosheet (GNS) suspensions to form alternating layers, with CNF layers providing mechanical strength and CNF/GNS layers enhancing thermal and electrical conductivity (Figure 15b). The filtration method is ideal for nanocellulose due to its high aspect ratio and strong hydrogen bonding, which creates compact films. By adjusting suspension concentration and filtration time, the packing density of the nanofibers can be controlled.^296^ The resulting films have excellent mechanical properties, such as high tensile strength and flexibility, making them suitable for applications like food packaging, flexible electronics, and water purification membranes.^297,298^

Once the filtration step is complete, the wet film is typically subjected to drying under controlled conditions. Drying method strongly affects thickness, porosity, and internal structure, all of which influence the film’s final performance. Air drying, vacuum drying, and freeze-drying are commonly employed methods.^299,300^ Air drying leads to dense, compact films, while freeze-drying can preserve the porous structure of the film, which is particularly useful for applications requiring high surface area, such as in catalytic or adsorptive membranes.^301,302^ In some cases, chemical cross-linking or heat treatments are applied to further enhance the mechanical or wet stability of the films.^303,304^ For example, lignin can be added to the nanocellulose suspension to introduce additional bonding interactions, improving the hydrophobicity of films.^305,306^ These postfiltration treatments allow the customization of films for specific applications by adjusting their mechanical properties, water resistance, and thermal stability.

The filtration method offers several advantages, especially when processing nanocellulose. It enables the production of films with well-defined thickness and uniformity, which are critical for high-performance applications. This method is scalable and can be adapted to produce films with varying properties by adjusting the suspension concentration, filtration time, and drying conditions. Filtration can also be combined with other techniques, such as casting or coating, to create hybrid materials with tailored properties.^307,308^ However, challenges remain, including the slow filtration process, particularly with highly concentrated suspensions or thick films. Solvent removal can be less efficient compared to methods like electrospinning, and defects like pinholes or cracks may form if filtration conditions are not properly controlled, compromising the film’s mechanical strength or barrier performance.

#### 3.2.3 Coating Method

The coating method is a versatile and efficient approach for the preparation of LCFs on various substrates. This technique involves depositing a thin layer of lignocellulosic material, such as cellulose, hemicellulose, lignin, or nanocellulose, onto a surface to form a functional film. The coating can be applied through different techniques, including spin-coating, blade coating, slot-die coating, dip-coating, and spray-coating (Figure 15c). Each of these techniques allows for control over film thickness, surface uniformity, and the mechanical and barrier properties of the resulting film.

Spin-coating is a widely used technique at the laboratory scale for producing uniform thin films in academic research. In this method, a small amount of lignocellulosic material in solution is placed on a substrate, which is then spun at high speed.^309,314^ The centrifugal force spreads the solution evenly across the surface, and the thickness of the film can be controlled by adjusting the spin speed, solution viscosity, and spin duration.^315,316^ Spin-coating is ideal for producing films with excellent surface uniformity, but it is typically limited to small-scale applications and requires precise control to avoid defects such as streaks or thickness variations.

Slot-die coating is a high-precision method commonly applied in industrial-scale production for continuous film fabrication. In this technique, a slot-die head deposits a solution of lignocellulosic material onto a moving substrate at a controlled flow rate, forming a uniform film.^317^ This method allows for excellent control over film thickness and is highly scalable for large-scale production of LCFs.^311,318^ Slot-die coating is often preferred for applications requiring consistent film thickness over large areas.^319^

Blade coating, also known as doctor blade coating, involves spreading a solution of lignocellulosic material onto a substrate using a blade to control the film’s thickness.^320^ This method offers good control over the thickness by adjusting the gap between the blade and the substrate, and it is compatible with large-area coatings.^321,322^ Blade coating is particularly useful for producing relatively thicker films and is scalable for industrial applications. However, achieving high uniformity can be challenging, especially for very thin films, and the method requires optimization of parameters such as solution viscosity and coating speed.

Dip-coating is one of the simplest and most explored methods in laboratory studies for film formation. In this process, a substrate is dipped into a solution or suspension containing lignocellulosic material, and then slowly withdrawn at a controlled speed.^323^ As the substrate is removed from the solution, a thin layer of the material adheres to its surface.^324^ The thickness of the film depends on several factors, including the concentration of the lignocellulosic solution, the withdrawal speed, and the viscosity of the solution.^325^ Dip-coating is ideal for producing uniform films, but it may require multiple coatings to achieve the desired thickness.

Spray-coating is increasingly adopted in research on flexible electronics, sensors, and barrier coatings. This method is particularly useful for covering large surface areas and creating thin, uniform films.^326^ The thickness of the resulting film can be controlled by adjusting the spray parameters, such as the nozzle size, solution concentration, spray pressure, and distance between the nozzle and substrate.^327,328^ Spray-coating is commonly used for flexible electronics, sensors, and barrier coatings.

The choice of substrate plays a crucial role in determining the final properties of LCFs. Substrates such as glass, plastic, metal, and paper offer varying levels of adhesion, flexibility, and surface smoothness. Flexible substrates like polyethylene (PE) and polyethylene terephthalate (PET) are commonly used for packaging and electronics, while glass and metal are preferred in optical or high-barrier applications. Surface modifications, including plasma and chemical treatments, are often employed to enhance surface energy and improve film adhesion and uniformity.

The coating method shows strong potential in packaging, electronics, and energy-related applications due to its precision and scalability. However, challenges remain, such as achieving uniformity over large areas, avoiding defects like cracks or bubbles, and ensuring sufficient film adhesion to prevent delamination. Current research trends include the use of lignocellulosic nanomaterials, formulation of biodegradable and water-based coatings, and hybrid strategies to enhance film functionality and environmental compatibility.

#### 3.2.4 Regeneration

The regeneration method is widely used for producing LCFs, especially from RC. It generally involves five major steps: dissolution, film casting, coagulation, drying, and optional functionalization. In this process, cellulose is dissolved in a suitable solvent and then precipitated in a coagulation bath, leading to the formation of solid cellulose films. A representative example is the work of Dahlström et al.,^329^ who used a LiOH/urea system and alcohol coagulation to prepare RC films with tunable structures (Figure 16a), demonstrating the method’s adaptability. RC films are known for their mechanical strength, transparency, and biocompatibility, making them suitable for packaging, biomedical devices, and flexible electronics.

Dissolving cellulose is the first and often most challenging step in the regeneration process.^330^ To overcome its strong hydrogen bonding network, specialized solvents such as ionic liquids (ILs), NaOH/urea solutions, or *N*-methylmorpholine N-oxide (NMMO) are commonly used.^331^ These solvents enable cellulose to form a homogeneous solution suitable for processing. Once dissolved, the solution is cast onto a substrate or extruded into a desired shape, initiating film formation. It is then submerged in a coagulation bath, typically containing an antisolvent like water or alcohol, to induce the precipitation of cellulose.^332^ The conditions of coagulation, such as temperature, nonsolvent concentration, and exchange rate, critically affect the structural and mechanical properties of the final film.^333,334^ Slower coagulation promotes molecular realignment and crystallinity, while faster coagulation may lead to greater porosity but reduced strength.^335^

After coagulation, the films undergo drying to remove residual solvent and water. Improper drying may cause cracking, shrinkage, or warping, compromising film uniformity and mechanical integrity.^336,337^ Controlled drying is therefore essential for maintaining film quality.

RC films are versatile, with properties like crystallinity, transparency, and mechanical strength being adjustable based on the processing conditions. These films can also be made porous, which is important for applications such as filtration or biomedical scaffolds. Additives or functional materials, such as nanoparticles or antimicrobial agents, can be incorporated into the cellulose matrix during the dissolution phase, allowing for multifunctional films with tailored properties.^338^ The regeneration method offers the advantage of producing films with excellent mechanical and customizable properties. However, challenges remain, particularly regarding the environmental impact and cost of the solvents used, as well as the difficulty in achieving uniform film quality during large-scale production.

#### 3.2.5 Electrospinning

Electrospinning is a highly versatile method for producing nanofibrous LCFs with properties like high surface area, porosity, and customizable mechanical characteristics.^342,343^ This process uses a high-voltage electric field to draw polymer solutions or melts into ultrafine fibers, which are collected on a substrate. The fibers, ranging from micro- to nanoscale, solidify as the solvent evaporates, forming a porous mat. While not strictly lignocellulosic films, PLA-based nanofiber films prepared by Chen et al.^339^ incorporated l-lactide functionalized CNC as a reinforcing agent (Figure 16bi), demonstrating the role of nanocellulose as a functional additive in synthetic polymer matrices. Similarly, Ji et al.^340^ demonstrated a hybrid approach by combining electrospinning of epoxy nanofibers with simultaneous spraying of CNFs, producing a three-dimensional nanoweb structure with improved thermal stability, optical transparency, and mechanical strength (Figure 16bii). This system also used a synthetic matrix, with lignocellulosic components serving as structural enhancers. These examples illustrate the broader utility of lignocellulosic materials as functional additives, but for this review, we primarily focus on electrospun films where lignocellulose forms the continuous matrix.

In electrospinning, key parameters such as applied voltage, flow rate, and the distance between the nozzle and collector are critical for controlling fiber formation.^344^ Higher voltage stretches the fibers more, producing thinner fibers, while solution properties like viscosity and concentration influence fiber thickness and morphology.^345^ For instance, higher-viscosity solutions tend to produce thicker fibers, whereas lower-viscosity solutions may result in thinner or beaded fibers.^346^ Solvent evaporation rates also play an important role in fiber quality, with more volatile solvents causing faster drying and possibly irregular fiber shapes, while slower evaporation allows for smoother fiber formation.^347^ Environmental factors, such as humidity and temperature, also influence the morphology of electrospun fibers.^348^ Higher humidity levels can lead to porous fibers due to slower solvent evaporation, while lower humidity tends to produce smoother fibers. Temperature affects solvent evaporation rates and solution fluidity, further impacting fiber formation.

Electrospinning lignocellulosic materials, such as nanocellulose, cellulose derivatives, and lignin, have attracted significant interest due to their superior mechanical strength, biodegradability, and biocompatibility.^349–351^ CA is commonly electrospun because it dissolves more easily than cellulose itself, and after electrospinning, the fibers can be deacetylated to produce pure cellulose nanofibers. This two-step approach allows the fabrication of cellulose films, which would otherwise be difficult due to the solubility challenges of native cellulose.^352^

Lignin, another important lignocellulosic component, can also be electrospun, resulting in fibers with unique properties like UV absorbance and antimicrobial activity. By blending lignin with other polymers or nanocellulose, the processability and functional properties of the resulting nanofibrous films can be enhanced, making them suitable for applications in packaging, biomedical devices, and energy storage.^353,354^

Despite its advantages, electrospinning faces challenges, particularly in processing native cellulose due to its poor solubility in most solvents. The need for expensive or toxic solvents also limits scalability and environmental sustainability. Furthermore, achieving uniform fiber diameter and consistent film thickness requires fine-tuning the process parameters, which can be time-consuming. Low throughput in conventional electrospinning systems is another barrier to large-scale production. Future research aims to address these challenges by developing greener solvent systems and improving process scalability through high-throughput techniques like needleless or multinozzle electrospinning.

#### 3.2.6 Other Emerging Methods

In recent years, novel methods for preparing LCFs have emerged, complementing the traditional techniques mentioned above. These methods, ranging from precision-driven assembly to bioinspired structural retention, offer diverse pathways to tailor film morphology, functionality, and sustainability. They can be broadly classified as follows: precision-driven (e.g., LbL), shape-adaptable (e.g., 3D printing), microstructure-controlled (e.g., freeze-casting), and bioinspired (e.g., top-down wood structuring).^355–358^

LbL assembly is a versatile and precise technique for creating multilayered films by sequentially depositing oppositely charged materials such as polymers, nanoparticles, or biomolecules. This method enables nanoscale control over layer thickness and composition, making it suitable for applications like gas barriers, drug delivery systems, and electronic devices. In LCFs, it enhances mechanical strength, conductivity, and multifunctionality such as flame resistance or antibacterial performance. Examples include nanocellulose-based flame-retardant films, hydrophobic microcapsules from CNC for drug delivery, and graphene oxide-reinforced multilayers for mechanical enhancement.^359–361^

3D printing builds materials layer by layer using customized inks, enabling precise geometric control and integration of functionality. It is particularly suited for producing LCFs with complex shapes, graded porosity, or tailored mechanical zones, useful for wearable devices, flexible sensors, and biomedical scaffolds. Representative works include 3D-printed cellulose/BN composite films for energy harvesting, shear-aligned CNF films with enhanced toughness, and lignin/PLA inks with UV-shielding or antioxidant properties.^358,362,363^ Yet, print resolution, ink rheology, and mechanical anisotropy still limit broader adoption.

Freeze-casting creates anisotropic, porous structures through directional freezing and sublimation. This allows precise control over pore size, orientation, and gradient structures, relevant to filtration, insulation, and tissue engineering. For example, Liu et al.^341^ developed porous cellulose photonic films via controlled unidirectional interlayer freezing (Figure 16c), enabling rapid visual sensing with precise control over pore structure.

Top-down approaches retain the native cellulose alignment by selectively removing lignin from natural wood, creating transparent films with preserved mechanical integrity. Such structures combine optical clarity, toughness, and sustainability, making them ideal for smart windows, solar devices, and optical sensors.^364^ Recent advances include UV-blocking, thermochromic, and luminescent wood films that retain natural texture while introducing functionality.^365–367^ Remaining issues involve uniform lignin removal and large-area consistency.

In summary, these emerging methods expand the design space for LCFs beyond traditional techniques. Their integration with casting, filtration, or regeneration holds promise for multifunctional, application-tailored lignocellulosic films. Future research should focus on improving scalability, structural precision, and green processing compatibility.

### 3.3 Properties of Lignocellulosic Films

#### 3.3.1 Mechanical Properties

LCFs exhibit a wide spectrum of mechanical properties depending on their chemical composition, microstructure, and processing method. Their performance can range from flexible and ductile to stiff and strong, enabling application in diverse functional contexts.^368–373^ The mechanical behavior of LCFs is influenced by factors such as the type and ratio of lignocellulosic components (cellulose, hemicellulose, lignin), fiber dimensions and orientation, degree of polymerization, and interfacial interactions. Representative values for tensile strength, stiffness, and elongation at failure across different categories of LCFs are summarized in Table 2. These structure–property relationships vary considerably across different categories of LCFs, and are discussed in more detail in the following sections.

**Table 2:** Mechanical Properties of Various Types of LCFs^a^

categories of LCF	tensile strength (MPa)	stiffness (MPa)	elongation at failure (%)	ref
cellulose derivative films	3.3–99 (20–60)	30–1,200 (100–500)	4–148.8 (10–40)	^179,180,183,187,195–200^
RC films	4.19–400 (50–150)	343–33,000 (1,000–10,000)	0.5–50 (5–20)	^17,24,209,210,212–214,217,220–223,225,374–376^
nanocellulose films	8.4–575 (80–200)	300–32,000 (1,000–10,000)	2.4–34 (5–15)	^235,240,247,377–381^
hemicellulose films	3.17–143 (20–80)	10–11,600 (100–3,000)	1.86–97.5 (5–30)	^22,253,256,257,260,261^
lignin based films	0.26–61 (5–30)	10–3,097 (100–1,000)	1.1–132 (5–30)	^265,269,270,275,382,383^
whole lignocellulosic biomass films	3.5–190 (20–80)	167–9,440 (500–5,000)	1–10 (2–8)	^277,280,283–286,384,385^

a Note: Reported values reflect both the full range and typical performance (shown in parentheses). Differences in processing strategies, lignocellulosic biomass sources, post-treatment, and testing protocols contribute to variability across studies.

Cellulose derivative films, such as those made from CA, CMC, and HPC, typically exhibit moderate mechanical performance, with tensile strengths ranging from ∼10 to 100 MPa and elongation at break values varying from a few percent to over 100%. These properties are highly tunable and depend on the degree of substitution, molecular weight, and presence of plasticizers or blending agents. The substitution of cellulose hydroxyl groups reduces interchain hydrogen bonding, often lowering tensile strength but improving flexibility and processability.^386^ As a result, cellulose derivatives are commonly used in applications where mechanical strength is less critical than film-forming ability, flexibility, and compatibility with additives.

The mechanical performance of RC films is generally superior to that of cellulose derivatives, with typical tensile strengths ranging from 50 to 150 MPa and Young’s modulus reaching up to tens of GPa.^387^ These properties stem from the preservation or reformation of hydrogen bonding and crystallinity during the regeneration process. Postprocessing treatments such as uniaxial or biaxial stretching can further improve chain orientation and enhance strength and stiffness. The balance of strength, transparency, and processability makes RC films promising candidates for sustainable packaging and optical substrates.^222^

Cellulose possesses outstanding mechanical properties, with specific modulus and strength values exceeding those of most metals.^388^ Yet, a key challenge is effectively translating these remarkable nanoscale properties into macroscale films. For instance, CNCs derived from biomass can reach tensile strengths of about 6–10 GPa and stiffness values near 150 GPa.^389–392^ In comparison, natural lignocellulosic biomass (e.g., wood) shows much lower tensile strength (<100 MPa) and stiffness of only a few GPa, while conventional cellulose paper sheets typically exhibit even weaker mechanical performance than natural wood.^393^

Extensive research has focused on improving the mechanical performance of nanocellulose films, with particular attention to the inherent trade-off between strength and toughness. These two properties are often considered mutually exclusive: toughness depends on a material’s ability to dissipate localized stress through deformation, whereas materials with higher strength typically exhibit greater brittleness.^394,395^ Conversely, materials with lower strength but higher deformability are usually tougher. Interestingly, recent studies show that cellulose nanopaper (CNP), with a thickness of 30–50 μm and primarily made from CNFs, can achieve both superior strength and toughness compared to conventional paper made from larger cellulose fibers.^396^ For example, reducing fiber diameter from 27 μm to 11 nm yields CNP with strength and toughness enhanced by factors of 40 and 130, respectively, relative to standard cellulose microfiber paper. This observation highlights a remarkable and highly desirable scaling law: as fiber diameter decreases, both strength and toughness increase.

Elucidating the mechanisms behind the superior strength and toughness of CNP offers important guidance for broader material design strategies.^372,397^ The exceptional strength of CNP arises from the markedly reduced defect size associated with nanoscale cellulose fibers, while its toughness is attributed to the dynamic hydrogen bonding among hydroxyl groups along the cellulose chains. These reversible bonds continuously form, break, and reform, enabling efficient energy dissipation and thereby enhancing fracture toughness. This concurrent improvement in strength and toughness achieved by reducing fiber dimensions exemplifies a bottom-up design principle that may help overcome the long-standing strength–toughness trade-off in other material systems.^397,398^ Furthermore, recent studies have highlighted several key factors that govern the mechanical performance of CNP:

##### Alignment

CNP is typically composed of randomly entangled nanofibers, but aligning these fibers can markedly improve its mechanical properties.^399,400^ For instance, Zhu et al.^23^ demonstrated that hot-pressing could produce transparent CNP with highly aligned cellulose nanofibers. By adjusting the cutting angle of the wood, they controlled the degree of alignment and achieved tensile strengths of ∼350 MPa, about three times higher than that of isotropic CNP. Lower cutting angles enhanced alignment and strength, whereas higher angles reduced strength to ∼190 MPa. Other approaches, such as wet-drawing, can also increase anisotropy. For instance, Wang et al.^401^ reported that wet-drawn bacterial cellulose films exhibit well-aligned structures with tensile strengths approaching 1 GPa.

##### Degree of Polymerization

The degree of polymerization (DP) of cellulose plays a critical role in determining the mechanical performance of CNP, with higher DP values generally leading to superior mechanical properties.^402^ Fang et al.^403^ employed an alkaline sulfite-anthraquinone-methanol pulping method to effectively remove lignin and hemicellulose while minimizing cellulose degradation. This treatment promoted hydrogen bonding among adjacent fibers during mechanical pressing, resulting in CNP with outstanding mechanical performance, including tensile strength exceeding 1 GPa, a modulus of ∼60.2 GPa, and a fracture energy of 15.2 MJ·m^–3^.

##### Aspect Ratios of Constituent Nanofibers

The mechanical performance of CNP is strongly governed by the diameter, length, and length-to-diameter ratio of its nanofiber building blocks.^404–406^ Material strength is largely dictated by defect size, with smaller defects yielding higher strength. Reducing fiber diameter, as in cellulose nanofibers, produces densely packed networks with minimized defect sizes, thereby enhancing strength. Zhu et al.^396^ demonstrated that the tensile strength of CNP scales inversely with the square root of fiber diameter, a relationship validated experimentally over 3 orders of magnitude. Mechanical failure in CNP typically involves relative nanofiber sliding; longer fibers promote more successive hydrogen bond breaking and reformation events during fracture, dissipating greater energy and thus improving toughness. Flaw sensitivity, characterized by the fractocohesive length,^407,408^ is another key factor influenced by nanofiber aspect ratio. Chen et al.^395^ showed that higher aspect ratios increase the fractocohesive length, thereby improving flaw tolerance. Such insights into flaw sensitivity provide valuable guidance for designing mechanically robust CNP.

Compared to cellulose, the lower molecular weight, highly branched structure, and limited interchain hydrogen bonding capacity of hemicellulose hinder efficient load transfer, resulting in weaker films. As summarized in Table 2, hemicellulose films typically exhibit tensile strengths of 20–80 MPa and elongation at break ranging from 10% to 50%. Despite their relatively modest strength, hemicellulose films are often valued for their good flexibility and ease of processing. Their mechanical performance can be substantially improved through cross-linking or blending with reinforcing agents such as nanocellulose and other nanomaterials.^22,409^ While hemicellulose films may not achieve the mechanical robustness of CNP or RC films, their biodegradability, oxygen barrier properties, and tunable flexibility make them well suited for packaging and coating applications where high mechanical strength is not a primary requirement.

Lignin-based films typically show low to moderate mechanical strength, reflecting the complex and irregular structure of lignin macromolecules. As summarized in Table 2, lignin-based films display tensile strengths from 0.26 to 61 MPa, stiffness values between 10 and 3097 MPa, and elongation at break up to ∼130%. This broad variability arises from differences in lignin type (e.g., kraft, organosolv, soda, enzymatic), purity, and film formulation strategies. Lignin’s highly heterogeneous and amorphous structure results in films that are often brittle and prone to cracking, especially when lignin is used alone.^410^ However, lignin-based films can be toughened by chemical modification (e.g., esterification, etherification) or blending with flexible biopolymers or plasticizers (e.g., PEG, glycerol).^27^ Such approaches reduce internal stresses, enhance compatibility, and improve elongation and toughness. Additionally, fractionation and molecular weight tailoring of lignin have shown promise in improving its film-forming ability and mechanical behavior.^411^

The mechanical properties of whole lignocellulosic biomass films are governed by the synergistic and competing interactions among cellulose, hemicellulose, and lignin, which are retained in their native or partially modified forms during film fabrication.^370^ Unlike purified or reconstituted cellulose-based films, these materials maintain the heterogeneous architecture of plant cell walls, making them attractive for low-cost, sustainable film production with minimal chemical processing. As summarized in Table 2, whole biomass films typically exhibit tensile strengths in the range of 20 to 80 MPa, and elongation at break between 2% and 8%. These values reflect moderate mechanical performance overall, normally below that of cellulose films, but generally superior to hemicellulose- or lignin-rich films due to the presence of intact cellulose nanofibers. While whole lignocellulosic biomass films may not match the mechanical strength of highly engineered cellulose films, they offer a compelling balance between performance, sustainability, and processing simplicity. Their utility is particularly promising in applications that require moderate strength, biodegradability, and cost efficiency, such as disposable packaging and agricultural mulch films.

#### 3.3.2 Barrier Properties

LCFs possess inherent barrier properties due to the complex molecular structure and unique physicochemical characteristics of their constituents. The high crystallinity of cellulose imparts resistance to molecular diffusion, while lignin, with its hydrophobic and aromatic structure, significantly limits permeability to water and oxygen.^412–414^ Together, these features make LCFs promising candidates for sustainable packaging applications. Two of the most critical parameters for evaluating LCFs as barrier materials are water vapor barrier and oxygen barrier performance. These are commonly quantified using the following definitions and equations:

Water vapor transmission rate (WVTR)

$$\mathrm{WVTR}=\frac{\mathrm{weight of vapor transmitted}}{\mathrm{area}\times \mathrm{time}}g/\left(m^{2}\cdot \mathrm{day}\right)$$

Water vapor permeability (WVP)

$$\mathrm{WVP}=\frac{\mathrm{WVTR}\times L}{P_{\mathrm{sat}}\times \Delta \mathrm{RH}}g\cdot \mu m/\left(m^{2}\cdot \mathrm{day}\cdot \mathrm{kPa}\right)$$

where *L* is the film thickness (μm), *P*_sat_ is the saturated water vapor pressure at the testing temperature (e.g., ∼3.17 kPa at 25 °C), and ΔRH is the relative humidity difference across the film, expressed as a decimal (e.g., 75% – 0% = 0.75).

Oxygen transmission rate (OTR)

$$\mathrm{OTR}=\frac{\mathrm{volume of oxygen transmitted}}{\mathrm{area}\times \mathrm{time}}{\mathrm{cm}}^{3}/\left(m^{2}\cdot d\right)$$

Oxygen permeability (OP)

$$\mathrm{OP}=\frac{\mathrm{OTR}\times L}{\Delta P}{\mathrm{cm}}^{3}\cdot \mu m/\left(m^{2}\cdot \mathrm{day}\cdot \mathrm{atm}\right)$$

where Δ*P* is the oxygen partial pressure difference across the film (typically 1 atm under 100% O_2_ vs 0% O_2_ testing conditions).

Among these, WVP and OP are intrinsic properties of the material, independent of film thickness, and are widely used to compare the true barrier performance of different films. However, a major challenge in the literature is the inconsistent use of units, such as g·mm/(m^2^·h·kPa), cm^3^·cm/(cm^2^·s·Pa), or mL·μm/(m^2^·day·mmHg), which complicates data interpretation and cross-study comparisons. In this review, we adopt the units g·μm/(m^2^·day·kPa) for WVP and cm^3^·μm/(m^2^·day·atm) for OP, which are now widely accepted for their clarity, consistency, and direct correlation with physical meaning.^415^

As summarized in Table 3, LCFs generally exhibit excellent oxygen barrier properties, particularly under dry or low humidity conditions, due to the dense hydrogen-bonding network in cellulose-based matrices. However, their water vapor barrier performance is often limited by the hydrophilic nature of cellulose and hemicellulose, which allows moisture penetration and plasticization at high relative humidity.^416^ Lignin, in contrast, can improve moisture resistance due to its hydrophobicity, but often at the cost of film transparency or flexibility. The barrier properties vary significantly depending on the composition, nanostructure, and processing methods used for different types of LCFs. In the following sections, we discuss in detail the water vapor and oxygen barrier properties of each category of LCF.

**Table 3:** Oxygen and Water Vapor Permeability of Different Types of LCFs

types of LCFs	water vapor permeability (g·μm/(m^2^·day·kPa))	test conditions	oxygen permeability (cm^3^·μm/(m^2^·day·atm))	test conditions	ref
CA films	1,585	38 °C, 90/0%	7,997	23 °C, 50%	^417^
CMC films	29,376	37.8 °C, 40/10%	2,240	23 °C, 30%	^418^
HPC films	12,960	25 °C, 75/0%	28,020	23 °C, 50%	^419^
RC films	86.4–25,920	25 or 38 °C, 0–100%	0.5–50,000	23 °C, 0–100%	^201,415,420^
TEMPO oxidized CNF films	2,882–4,220	23 °C, 50/0%	63–152	23 °C, 50%	^421^
MFC films	916	23 °C, 50/0%	35–43	23 °C, 50%	^421^
carboxymethylated CNF films	285–5,070	23 °C, 50% or 80%	53–2,584	23 °C, 50% or 80%	^422^
CNF films	3,129–11,842	21.1 or 26.7 °C, 50–90%	28–2,610	23 °C, 50–90%	^423^
partially esterified CNF films	3,982	23 °C, 50%	60.8	23 °C, 50%	^424^
modified CNC films	2,800–3,300	22 °C, 52/0%	79–1,419	23 °C, 50–80%	^425^
hemicellulose films	17,300–60,000	25 or 38 °C, 50–100%	21–55,323	23 °C, 50–83%	^291,426,427^
lignin-containing CNF films	2,570–3,280	23 °C, 50/0%	0.76–1.84	23 °C, 50%	^240^

Cellulose derivative films, particularly cellulose esters such as CA films, exhibit moderate to good water vapor barrier properties due to their relatively hydrophobic ester groups. For example, CA films show a WVP of 1,585 g·μm/(m^2^·day·kPa) under high humidity conditions (38 °C, 90/0% RH), which is superior to more hydrophilic cellulose derivatives.^417^ However, their oxygen barrier performance is relatively modest, with an OP of ∼7,997 cm^3^·μm/(m^2^·day·atm). In contrast, hydrophilic cellulose ethers like CMC and HPC films demonstrate inferior water vapor barrier properties. For example, the WVP value of CMC films was reported as high as 29,376 g·μm/(m^2^·day·kPa) under moderate humidity conditions (37.8 °C, 40/10% RH).^418^ Nonetheless, their oxygen barrier is better than CA films, especially under lower humidity (e.g., 23 °C, 30% RH), mainly due to the tighter polymer packing in the dry state.

RC films show highly variable barrier properties, with WVP values ranging from 86.4 to 25,920 g·μm/(m^2^·day·kPa) and OP values from 0.5 to 50,000 cm^3^·μm/(m^2^·day·atm), depending on cellulose sources, processing methods, and post-treatment. At low RH, RC films can form dense hydrogen-bonded networks that provide excellent barrier performance to both water vapor and oxygen. However, under high humidity, their performance degrades rapidly due to the inherent hydrophilicity of cellulose, which leads to moisture uptake, matrix swelling, and plasticization.^428^ Bedane et al.^420^ systematically examined the effect of RH (ranging from 0% to 100%) on the WVP of RC films and reported values spanning from 86 to 25,920 g·μm/(m^2^·day·kPa). The wide variation reflects the cellulose matrix’s strong affinity for water. As RH increases, RC films absorb moisture and swell, resulting in a significant increase in WVP. In another study, Okugawa et al.^201^ investigated the influence of water regain on the OP of RC films. In the dry state, these films exhibited exceptionally low OP, qualifying them as “very high”-performance gas barriers. However, as water regain increased, the OP rose markedly, eventually reaching levels comparable to low-density polyethylene (LDPE), a material considered a “poor” gas barrier. This dramatic decline in barrier performance is attributed to moisture-induced swelling, which increases intermolecular spacing and enhances micro-Brownian motion of cellulose chains, thereby facilitating oxygen diffusion through the film matrix.

Nanocellulose films, including those made from CNFs, MFCs, CNCs, and their modified forms, also exhibit a broad range of barrier properties, with WVP values ranging from 285 to 11,842 g·μm/(m^2^·day·kPa) and OP values from 28 to 2,610 cm^3^·μm/(m^2^·day·atm) (Table 3). These values are influenced by nanofiber morphology, film density, surface chemistry, and processing methods. For example, Wei et al.^423^ investigated the impact of mechanical fibrillation on CNF films and found that increased fibrillation improved film density and reduced surface roughness, leading to enhanced water vapor barrier performance at moderate humidity. Films made from more intensively fibrillated CNFs showed up to 40% lower WVP at 50% RH, while OP remained largely unchanged. However, under high humidity (90% RH), both water vapor and oxygen barrier performance declined due to the intrinsic hydrophilicity of cellulose. To improve the barrier properties of nanocellulose film under high humidity, a variety of physical and chemical modification strategies have been developed, which will be discussed in detail in sections 4.1 and 4.2.

Hemicellulose films generally have inferior barrier properties compared to cellulose-based films. As summarized in Table 3, their WVP ranges from 17,300 to 60,000 g·μm/(m^2^·day·kPa), and OP ranges from 21 to over 55,323 cm^3^·μm/(m^2^·day·atm). This is primarily due to the branched, amorphous molecular structure of hemicelluloses, which prevents the formation of tightly packed crystalline domains. The loosely organized network allows for easier diffusion of small gas and water molecules, resulting in low resistance to moisture and oxygen. Cross-linking and blending with nanomaterials (e.g., nanocellulose, nanoclays) or hydrophobic polymers can partially mitigate these limitations.^429,430^

There is limited data on the barrier performance of pure lignin films for packaging applications. However, lignin is commonly used as an additive or surface coating to improve moisture resistance in cellulose-based films. For instance, lignin-containing CNF films demonstrate significantly improved oxygen barrier properties, with OP values as low as 0.76–1.84 cm^3^·μm/(m^2^·day·atm), and WVP in the range of 2,570–3,280 g·μm/(m^2^·day·kPa).^240^ These improvements are attributed to lignin’s hydrophobic, aromatic-rich structure, which reduces water uptake and limits gas diffusion. Nevertheless, excessive lignin content may compromise film flexibility and transparency.

Whole lignocellulosic biomass films, which retain cellulose, hemicellulose, and lignin without fractionation, are gaining attention as a low-cost and sustainable alternative. However, research on their barrier performance is still at an early stage, and quantitative data remain scarce. More systematic investigations are needed to evaluate their potential as packaging materials and to understand how their inherent heterogeneity affects moisture and gas barrier properties.

#### 3.3.3 Optical Properties

LCFs have emerged as promising sustainable and biodegradable alternatives to petroleum-derived optical materials. Their optical properties, particularly transmittance and haze, are critical for performance in applications such as packaging, displays, solar cells, smart windows, and optical sensors. Transmittance refers to the proportion of incident light that passes directly through a film and is typically measured at 550 or 600 nm in the visible spectrum. Haze, on the other hand, quantifies the extent of light scattering, specifically the fraction of transmitted light that deviates at wide angles, affecting the clarity and diffusion characteristics of the film. Films with high transmittance and low haze are desirable for transparent substrates and display panels, whereas high-haze films with sufficient transmittance are favored in light-diffusing layers, such as those used in photovoltaics and antiglare applications. These optical characteristics can be tuned by controlling film parameters such as chemical composition, nanofiber morphology, surface roughness, refractive index mismatches, and the incorporation of light-scattering or UV-absorbing structures. Table 4 summarizes the representative optical performance of six major types of LCFs, including typical transmittance and haze values along with their key features. The following subsections will discuss each film type in detail.

**Table 4:** Optical Properties of Various Types of LCFs

categories of LCFs	transmittance (%)	haze (%)	key features	ref
cellulose derivative films	>80%	<10%	good clarity, tunable with additives	^431–433^
RC films	>80%	<10% to >80%	highly transparent, smooth surface, tunable haze	^434–437^
nanocellulose films	<30% to >90%	<10% to >90%	highly tunable; CNCs allows structural color, anisotropy	^438–441^
hemicellulose films	50–90%	<30%	moderate to high transparency, low haze	^286,442,443^
lignin based films	<80%	>10%	excellent UV-blocking, moderate visible transparency	^444–446^
whole lignocellulosic biomass films	<80%	>30%	UV-blocking, low to moderate visible transparency	^447–449^

Cellulose derivatives such as CA, HPC, and CMC can form transparent and flexible films with moderate to high optical clarity, depending on the degree of substitution and processing.^431–433^ These films typically show transmittance above 80% at visible wavelengths, making them suitable for packaging, coatings, and optical substrates. Plasticizers and blending agents can be used to reduce surface roughness and improve optical homogeneity. However, their haze is usually low (<10%), and they lack tunability in optical properties like structural color or UV-blocking unless functional fillers are added.^450^

RC films exhibit excellent optical transparency and smooth surface morphology. When properly processed, these films can achieve transmittance exceeding 90% at 550 nm and maintain low haze levels (<10%), making them comparable to synthetic optical films such PE and PET. Their transparency benefits from a densely reconstructed cellulose network with minimal light-scattering centers, particularly when the films are dried under tension or cast with directional alignment.^436^ However, the absence of inherent light-scattering structures limits haze, which can be advantageous or disadvantageous depending on the target application. For instance, low haze is ideal for display panels, while higher haze is preferred in solar light diffusion layers. To address this limitation, Hu et al.^437^ developed a one-step method to fabricate RC films with high transparency and adjustable haze by partially dissolving bamboo and softwood pulp in BzMe_3_NOH aqueous solution. The controlled dissolution preserved undissolved cellulose, which increased surface roughness and introduced light-scattering domains. The resulting films exhibited high transmittance (∼90%) with haze tunable from 14.3% to 83.7%.

Nanocellulose films, particularly those derived from CNFs and CNCs, demonstrate the highest degree of tunability in optical performance. By adjusting nanofiber diameter, surface chemistry, alignment, or assembly structure, these films can span a wide range of transmittance (up to >90% at 550 nm) and haze values (from <10% to >90%). For instance, Li et al.^451^ developed a strategy to regulate the optical haze of CNP by tailoring the morphology of cellulose nanoparticles and engineering CNF/CNC nanohybrids. By varying the particle shape and size from spherical CNCs to fibrous CNFs, they achieved continuous haze modulation (5–77%) while maintaining high transparency (∼90%). CNCs are particularly notable for their ability to self-assemble into chiral nematic structures, producing structural color that is responsive to environmental stimuli such as humidity, temperature, and gas exposure.^452^ These optical features enable applications in colorimetric sensors, smart coatings, and decorative or anticounterfeit materials.^453,454^

Hemicellulose films exhibit moderate to high optical transparency, with transmittance typically ranging from 50% to 90% and haze values below 30%, depending on their source, degree of purification, and processing conditions. While certain native hemicelluloses with low molecular weight and branched structures may show limited film-forming ability, xylan and glucomannan derivatives have demonstrated excellent potential to form uniform, transparent, and flexible films, especially when plasticized or chemically modified.^286,455^ Recent studies have shown that well-processed hemicellulose films can achieve the transmittance above 80% and haze below 10%, making them suitable for transparent packaging, coatings, and UV-protective applications when combined with functional additives such as lignin or nanoparticles.^442^

Lignin offers a unique advantage in UV shielding due to its aromatic-rich backbone and the presence of chromophoric functional groups such as phenolic hydroxyls, carbonyls, and conjugated double bonds. These groups strongly absorb ultraviolet light, particularly below 380 nm, making lignin an effective natural UV barrier.^456^ Lignin-based films typically exhibit excellent UV-blocking performance but reduced visible light transmittance, often below 80% and sometimes <50%, depending on the lignin content and structure.^444–446^ The inherent color of lignin, ranging from yellowish to dark brown, results from its conjugated aromatic structures and contributes to visible light absorption. Nevertheless, lignin can be incorporated into transparent matrices such as regenerated cellulose or cellulose derivatives to achieve a balance between UV shielding and visible transparency.^457,458^ This approach offers a sustainable alternative to synthetic UV absorbers for applications in packaging, electronics, and solar protection, where photodegradation resistance is critical.^459^ These molecular characteristics also guide film design strategies. Optimizing lignin dispersion at the nanoscale can minimize light scattering and reduce color impact, while enhancing compatibility with polymer matrices improves film uniformity and stability. This can be achieved through approaches such as chemical modification or copolymer blending. Such strategies are essential for tailoring optical performance and achieving a balance between effective UV protection and desirable visible transparency.

Due to the presence of unseparated cellulose, hemicellulose, and lignin, whole biomass films typically exhibit limited optical transparency and relatively high haze. Their turbid appearance is primarily caused by phase separation, fiber aggregation, and light absorption by lignin. However, optical properties can be improved through strategies such as controlled mechanical pressing, partial delignification, and surface smoothing. While these films are not suitable for high-end optoelectronic applications, they hold potential for UV-blocking wraps, translucent packaging, and biodegradable agricultural films where visual clarity is less critical. For instance, Li et al.^447^ developed a green and efficient approach to fabricate whole biomass films by directly dissolving wheat straw in ionic liquid (AmimCl). The resulting films showed excellent UV shielding (>97% UV blockage), moderate visible light transmittance (∼50% at 550 nm), and high haze (97%), highlighting their promise as sustainable packaging materials.

#### 3.3.4 Antimicrobial Properties

LCFs have attracted attention for antimicrobial applications, particularly in food packaging, due to their potential to inhibit bacterial growth and enhance product safety. However, the intrinsic antimicrobial properties of LCFs vary significantly depending on their composition. Cellulose derivative films, RC films, nanocellulose films, and hemicellulose films are primarily composed of polysaccharides, which inherently lack antimicrobial activity. Their neutral and hydrophilic nature tends to support, rather than resist, microbial colonization.^460^ Therefore, strategies to impart antimicrobial function to these films typically involve the incorporation of antimicrobial agents such as metal nanoparticles (e.g., Ag, ZnO), essential oils, natural plant extracts, or cationic polymers.^461^ These modifications can effectively inhibit the growth of both Gram-positive and Gram-negative bacteria, but their performance largely depends on the compatibility between the additive and the LCF matrix. More details will be discussed in section 5.1.1.

In contrast, lignin-based and lignin-containing films exhibit antimicrobial activity attributed to lignin’s unique aromatic structure and functional groups. Phenolic hydroxyl and methoxy groups in lignin can disrupt microbial cell membranes, resulting in the leakage of intracellular contents.^462^ In addition, lignin can promote the generation of reactive oxygen species on bacterial surfaces, interfere with microbial metabolism by binding to DNA and proteins, and inhibit enzymatic activity.^463–465^ Examples of lignin-containing cellulose films for active food packaging will be discussed in detail in Section 5.1.2. Whole lignocellulosic biomass films, which retain the natural mixture of cellulose, hemicellulose, and lignin, may also exhibit mild antimicrobial properties due to the presence of lignin. These films offer a sustainable and low-cost alternative with passive antimicrobial potential, especially suitable for applications where strong antimicrobial performance is not essential.

#### 3.3.5 Thermal Properties

LCFs exhibit complex thermal properties spanning multiple scales, including molecular-level thermal stability, macro-scale insulation, and processability under heat. These properties are not uniform across all LCFs but vary depending on their chemical composition, particularly the ratio and structure of cellulose, hemicellulose, and lignin, as well as film-forming methods and modification strategies. Therefore, this section categorizes thermal behaviors according to material type (cellulose-based, hemicellulose-based, lignin-based, and all-lignocellulose composite films) and thermal property type (stability, insulation, and thermoformability).

##### 3.3.5.1 Molecular-Scale Thermal Stability

The thermal stability of LCFs at the molecular scale is often evaluated through the decomposition behavior of the polymeric backbone, typically using thermogravimetric analysis (TGA) and differential scanning calorimetry (DSC). The initial decomposition temperature (*T*_onset_), peak degradation temperature (*T*_max_), and residual char yield are widely used parameters to describe their thermal stability.

Cellulose derivative films, such as CA, HPC, and CMC, present diverse thermal behaviors based on substitution degree and plasticizer use. CA films typically degrade in two steps, with *T*_onset_ around 270–290 °C and significant weight loss near 330 °C, where the first stage corresponds to the loss of acetyl groups and the second to the breakdown of the cellulose backbone.^466^ Plasticizers like triacetin or natural extracts often reduce the thermal stability but enhance processability. CMC-based films, especially when blended with additives like chickpea extract or silver nanoparticles, exhibit *T*_onset_ around 260 °C, but the incorporation of antioxidants can slightly delay degradation due to radical scavenging effects.^467^ HPC films reinforced with CNCs or surfactants show improved *T*_max_ (up to 325 °C), attributed to stronger hydrogen bonding and denser packing structures.^468^ These findings highlight the structure–property relationship between functional groups and the cellulose backbone, which governs their degradation pathways.

For regenerated cellulose films, the thermal stability strongly depends on the cellulose source and regeneration process. In general, regenerated cellulose films exhibit *T*_onset_ values ranging from 250–300 °C. For example, films prepared via low-temperature DMAc/LiCl dissolution using parenchyma cells exhibit *T*_onset_ around 255 °C, with a major weight loss between 320–350 °C, indicating moderate thermal stability due to limited crystallinity and residual solvent effects.^435,469,470^ In contrast, films regenerated from different cellulose types (e.g., Avicel, microcrystalline cellulose, cotton linter) in NaOH/urea systems show improved *T*_onset_ values (up to 295 °C) when higher crystallinity and fiber alignment are present.^471,472^ Moreover, cross-linking or incorporating thermally stable additives (e.g., citric acid, boric acid) can further improve thermal performance by forming a more rigid hydrogen-bonded network.

For nanocellulose films, the decomposition behavior is dictated by particle type, surface modification, and film architecture. CNF-based films generally degrade at 250–280 °C, while CNC-based films may begin degrading as early as 200 °C due to their higher sulfate group content and lower DP.^473^ However, combining CNC and CNF can create hybrid films with enhanced thermal resistance and mechanical strength, as seen in hybrid CNF-CNC films showing *T*_onset_ values near 280 °C and *T*_max_ over 330 °C.^474^ Further, alignment of nanocellulose improves heat resistance via better stress transfer and compact packing. For instance, films with aligned CNCs and hydrophobic coatings show higher onset degradation temperatures (∼300 °C) and reduced heat release rate, revealing synergistic enhancement of thermal and barrier properties. Additionally, surface functionalization (e.g., TEMPO oxidation, acetylation, or silylation) plays a dual role by improving or reducing thermal stability depending on the nature of grafted groups and their interaction with cellulose chains.

For hemicellulose-based films, their thermal resistance is generally lower than cellulose due to their branched, amorphous structures. *T*_onset_ values typically range from 190–240 °C. Sugar cane bagasse hemicellulose films, for example, showed a *T*_onset_ of 215 °C with a major mass loss around 260 °C, related to xylan depolymerization. Chemical modifications such as cationization, esterification, or cross-linking with glutaraldehyde can enhance thermal behavior.^475^ Films with quaternized hemicellulose and CNCs display improved *T*_max_ (∼270 °C), demonstrating the role of nanofillers and electrostatic interactions in stabilizing the matrix.^476^ Similarly, cross-linked hemicellulose films achieved using epichlorohydrin exhibit improved thermal resistance due to the formation of a rigid 3D network.^477^

Lignin-based films show variable thermal properties due to the heterogeneity of lignin structures. Their decomposition typically begins at 220–250 °C, with a wide degradation range extending up to 500 °C, reflecting the complexity of C–O–C and C–C linkages in the polyphenolic matrix. For instance, lignin-polyethylene films for agricultural use showed *T*_onset_ around 260 °C and enhanced thermal durability after compatibilization with maleic anhydride, likely due to better dispersion and matrix–filler interfacial bonding.^478^ Lignin-PVA composite films prepared via solution casting exhibited *T*_onset_ of ∼240 °C and a more gradual degradation curve, attributed to hydrogen bonding and partial aromatic stabilization.^478^ In addition, high-solid-content lignin films derived from kraft lignin showed significant residual char yields (∼35 wt %) at 600 °C, revealing lignin’s char-forming capability that contributes to flame retardancy.^27^

##### 3.3.5.2 Macro-Scale Thermal Insulation

At the macroscopic scale, the thermal insulation performance of LCFs primarily depends on factors such as structural density, pore architecture, component distribution, and interfacial thermal resistance. LCFs inherently possess low thermal conductivity, endowing them with excellent heat-blocking capabilities. This property can be further enhanced through the regulation of multilayer configurations, microporous structures, and anisotropic alignment.

Cellulose derivative films (e.g., CA, CMC) generally show average macroscopic thermal insulation performance. Pure CA films possess decent density but are typically used in applications requiring heat-sealing properties rather than thermal insulation due to their thermoplasticity and high optical transparency. Tedeschi et al.^479^ demonstrated that CAO films esterified with aliphatic acid esters introduced uniformly dispersed hydrophobic segments into the microstructure, improving structural stability under high humidity. However, their overall thermal conductivity remained relatively unchanged. Composite strategies involving inorganic nanoparticles such as SiO_2_ or TiO_2_ offer potential for enhancing interfacial thermal resistance and achieving moderate insulation performance.

Regenerated cellulose films typically exhibit moderate thermal conductivity, which limits their ability to meet stringent insulation requirements. However, their thermal insulation can be significantly improved through microstructural engineering or multicomponent integration. For instance, regenerated cellulose films prepared via a low-temperature DMAc solvent method achieve both high transparency and compact morphology, thereby reducing heat transfer efficiency.^469^ Other studies have reported the incorporation of hollow microspheres, micropores, or multilayered structures into regenerated cellulose matrices to enhance their insulating capability for applications such as flexible thermal pads.^15^

Nanocellulose films, owing to their nanoscale fiber networks, high crystallinity, and dense layered structures, demonstrate superior thermal insulation properties. Apostolopoulou-Kalkavoura et al.^480^ reported a CNF-based aerogel film fabricated via freeze-drying, exhibiting an ultralow through-plane thermal conductivity of 0.03 W·m^–1^·K^–1^, outperforming many commercial polymer foams. This exceptional performance is attributed to abundant nano- and microscale air pockets formed between nanofibers and substantial interfacial thermal resistance. Moreover, the alignment of layered structures hinders directional heat flow, enabling synergistic control over in-plane insulation and out-of-plane thermal dissipation.

Hemicellulose films, characterized by low crystallinity and loose structure, inherently exhibit low thermal conductivity, providing potential for thermal insulation. Braga and Poletto^475^ reported hemicellulose films derived from sugar cane bagasse, whose high amorphous content and hydrophilic structure facilitated the formation of hydrogen-bonded networks and moisture-retaining layers, thereby inhibiting efficient heat conduction. Nevertheless, the poor thermal and moisture stability of pure hemicellulose films under hot and humid conditions limits their broader thermal insulation applications, often necessitating cross-linking or hybridization to enhance stability and structural integrity.

Lignin-based films, benefiting from their aromatic structure and low polarity, demonstrate unique advantages in thermal blocking. Zadeh et al.^481^ summarized lignin’s role in polymeric membranes, noting that its high carbon content and nonpolar aromatic rings effectively suppress phonon and electron transport pathways, leading to reduced thermal conductivity. Furthermore, when blended with polyethylene or polylactic acid, lignin can form “core–shell” microstructures that enhance thermal resistance. For example, lignin-PE composite films developed by Chiappero et al.^482^ showed excellent heat shielding and weather resistance in agricultural mulch applications. However, the intrinsic brittleness and processing difficulty of lignin remain major challenges for its large-scale application in thermal insulation.

##### 3.3.5.3 Thermoformability and Thermoplasticity

Thermoplastic processability refers to a material’s ability to transition from a glassy state to a molten or softened state upon heating, enabling shaping through extrusion, hot pressing, or injection molding. For natural polymers, particularly those with polyhydroxy structures dominated by hydrogen bonding, their thermoplasticity is typically poor, and chemical modification or plasticization is required to improve their processability. The thermoplastic behavior of cellulose-based films varies significantly, depending largely on structural regularity, polarity, intermolecular interactions, and the modification strategies employed.

Regenerated cellulose films are typically fabricated via solution-based processes and are difficult to thermally mold directly. Their high molecular orientation and extensive hydrogen bonding network result in extremely high melting points and narrow thermal processing windows. Without the addition of plasticizers such as glycerol or PEG to lower the *T*_g_, their thermoplasticity is extremely limited. Moreover, conventional regeneration methods, such as the viscose process, NMMO method, or NaOH/urea system, primarily produce films via casting or immersion rather than hot pressing or melt processing. Therefore, regenerated cellulose materials are mostly suitable for ambient or low-temperature forming techniques, and thermoplastic modification remains a research focus.

Nanocellulose films also face limitations in thermoplasticity. Due to their small size, high crystallinity, and dense hydrogen bonding, CNFs and CNCs exhibit virtually no typical melting behavior. Upon heating, nanocellulose tends to undergo color changes or mass loss prior to melting. Although efforts have been made to enhance melt flow through surface esterification or blending with thermoplastics, pure CNF films are still not suitable for thermal molding. Some studies suggest using CNF as a reinforcing phase and blending it with thermoplastic matrices like PVA or polycaprolactone (PCL), followed by hot pressing to significantly improve overall formability.

Cellulose derivatives, by contrast, show better thermoplastic behavior, especially CA, which exhibits excellent thermal processability. CA can be stably melted between 160–200 °C and is widely used in hot pressing and extrusion film processing. Lucena et al.^483^ demonstrated that the addition of various plasticizers such as triacetin and phthalates further enhances the processing stability and flexibility of CA. Tedeschi et al.^479^ developed oleoyl-modified cellulose acetate (CAO) films with both flexibility and heat-sealing capability, which also maintain good structural integrity under high humidity. In addition, CMC, when properly cross-linked or plasticized, exhibits moderate suitability for heat sealing in water-soluble packaging films.

Hemicellulose films generally exhibit inferior thermoplastic processability. Natural hemicellulose, being a branched polysaccharide with high polarity and low crystallinity, tends to decompose during thermal processing. However, esterification, etherification, or blending with polymers can significantly enhance its thermal processability. For instance, Shao et al.^477^ used melamine and glutaraldehyde as dual cross-linkers to modify xylan films, improving their hydrophobicity, thermal stability, and heat-press formability. Huang et al.^476^ further suggested that the addition of cationic-modified nanocellulose could act as a thermally stable filler to improve film formation and reduce processing temperatures.

Lignin-based films exhibit considerable potential for thermoplastic processing. Despite the broad molecular weight distribution and relatively low thermal stability of native lignin, its aromatic backbone and high carbon content make it a promising compatibilizer and reinforcing agent in melt blending. Zadeh et al.^481^ comprehensively reviewed the use of technical lignins (e.g., kraft lignin, organosolv lignin) in thermoplastic systems, noting their effectiveness as performance-enhancing additives for thermoplastics such as PLA, PBS, and PE. Chiappero et al.^482^ successfully blended lignin with PE via melt extrusion to produce agricultural films with excellent processability, aging resistance, and environmental friendliness during degradation. Furthermore, lignin can be polymerized or grafted to form thermoresponsive chains, expanding its potential in thermoplastic composite fabrication.

#### 3.3.6 Surface Wettability

Surface wettability plays a pivotal role in determining the performance of LCFs in applications such as packaging, coatings, flexible electronics, and fluid separation. However, wettability is not an intrinsic property of all LCFs but rather a function of their surface chemistry, morphology, and processing methods.

Cellulose-based films, including certain cellulose derivative films, RC films, and nanocellulose films, typically exhibit hydrophilic surfaces due to abundant hydroxyl groups. Water contact angles (WCAs) are often in the range of 40–65° for CNF and RC films,^484^ although surface roughness and drying-induced porosity can result in lower apparent contact angles or rapid droplet absorption. In these fiber-rich films, the apparent WCA is influenced by capillarity and wicking, complicating measurement. In contrast, dense and smooth films, such as those derived from CA or trimethylsilyl cellulose, allow reliable WCA measurements using standard goniometry, with reported values ranging from 70° to 90°, depending on substitution and surface reconstruction.^485^ To increase hydrophobicity, surface modification techniques are widely used. Coating CNF films with natural waxes (e.g., halimium wax) or grafting with silanes or fluoroalkyl groups raises water contact angles to 100–120°, significantly improving water resistance.^486^ For instance, nanocellulose-PDMS composites and fluoroalkylated cellulose films have reached superhydrophobic regimes (>140°), depending on the surface topology and grafting density.^487^

Hemicellulose-based films are generally more hydrophilic, with WCAs typically around 40°, attributed to their branched structure and high content of hydroxyl and carboxyl groups.^477^ As with cellulose, surface treatments such as esterification, cross-linking with citric or fatty acids, and blending with hydrophobic polymers can significantly enhance water resistance.^488^

Lignin-rich films, due to lignin’s aromatic and low-oxygen-content structure (O/C ∼0.4), are inherently more hydrophobic. Incorporating technical lignin into CNF or hemicellulose matrices increases WCA and reduces water absorption.^489^ Blends of lignin with cellulose or surface-coating with alkali or kraft lignin also promote water repellency.^490^ However, dispersion uniformity and interfacial compatibility critically influence the manifestation of hydrophobicity.

Topography also synergistically affects wettability, as described by the Wenzel and Cassie–Baxter models. Rough hydrophilic surfaces increase droplet spreading, while rough hydrophobic surfaces become more water-repellent. For instance, rough hot-pressed CNF-lignin composite straws showed high WCA (∼120°), which remained ∼70° after 30 min in water, maintaining high tensile strength (∼30 MPa) and structural integrity.^491^

Overall, surface wettability of LCFs is governed by the interplay of surface chemistry (−OH, −COOH, aromatic rings), roughness, and processing route. Understanding and manipulating these variables enable the design of hydrophobic yet biodegradable LCFs suited for specific functional environments.

#### 3.3.7 Biodegradability

Biodegradability is a key sustainability attribute of lignocellulosic films, offering an environmentally friendly alternative to synthetic polymers. However, biodegradation behavior varies significantly across cellulose, hemicellulose, lignin, and their composites, depending on chemical structure, crystallinity, processing, and environmental exposure.

Cellulose-based films, especially those derived from regenerated cellulose or nanocellulose, are generally readily biodegradable in aerobic and anaerobic environments. Microorganisms such as fungi and bacteria secrete cellulases, including endoglucanase, exoglucanase, and β-glucosidase, can hydrolyze the β-1,4-glycosidic bonds in cellulose, resulting in glucose monomers.^492^ For example, CNF/alginate films showed substantial weight loss within weeks under composting,^493^ although high crystallinity can reduce enzyme accessibility and slow degradation.

Cellulose derivatives, notably CA, show slower degradation, especially when the DS exceeds 2.5. Films with lower DS (≤2.0) degrade more readily under composting and soil burial.^494^ High-DS CA may require enzymatic additives or oxidative pretreatment for effective breakdown. This distinction is important when comparing cellulose-based films: cellophane is biodegradable, but high-DS CA is recalcitrant under natural conditions.^495^

Hemicellulose-based films exhibit faster biodegradation due to their amorphous structure and branched heteropolysaccharide chains. Films from xylan, arabinoxylan, or hemicellulose-cellulose blends disintegrate rapidly in moist soil or compost.^496^ Cross-linking does not preclude biodegradability if degradable linkers like citric acid are used.^497^

Lignin, due to its aromatic and cross-linked structure, is the most biodegradation-resistant component in LCFs. Nevertheless, white-rot fungi and certain bacteria can degrade lignin via oxidative enzymes such as lignin peroxidase, manganese peroxidase, and laccase.^411^ Blending lignin with more labile polysaccharides like CNFs improves film degradability and compostability. Moreover, oxidized or depolymerized lignin show enhanced microbial susceptibility.^490^

Environmental conditions, including moisture, temperature, microbial load, and oxygen, also influence degradation, as do film thickness, surface area, and processing additives. Analytical techniques such as weight loss tracking, CO_2_ evolution, SEM, and XRD are commonly used to assess biodegradation. Emerging research has highlighted the potential of LCFs for transient electronics and marine-degradable materials. For instance, photoluminescent cellulose films degraded fully within 20 days in soil,^498^ and Meza et al. demonstrated a complete breakdown of cellulose-based sensors in compost and water without microplastic release.^499^

In conclusion, the biodegradability of LCFs is highly tunable via material choice and structural design. A clear understanding of enzymatic degradation pathways, environmental influences, and compositional differences is essential to developing high-performance yet eco-benign materials.

## 4 Functionalization of Lignocellulosic Films

### 4.1 Additives to Improve Physical Properties

Generally, the microcomposition and structure of LCFs can be regulated by nonchemical bonding through the addition of various additives.^500^ To improve moisture barrier performance, hydrophobic additives such as PDMS and wax have been incorporated into LCFs. Plasticizers, including glycerol and other similar compounds, not only enhance the film-forming properties of LCFs but also improve their flexibility and elongation. By introducing different reinforcing additives, the molecular distribution and structure can be modified through hydrogen bonding with the hydroxyl groups in lignocellulose, resulting in LCFs with enhanced mechanical properties. The following discussion will focus on hydrophobic modification additives, plasticizers, and strength-enhancing additives.

#### 4.1.1 Hydrophobic Additives

A droplet-surface contact angle below 90° is considered hydrophilic, while an angle above 90° indicates a hydrophobic surface. The contact angle is influenced by surface structure and chemistry. Typically, surfaces with high micro- and nanoscale roughness tend to be more hydrophobic due to a reduction in the droplet/surface contact area.^501^ Moreover, the chemical composition of lignocellulosic materials significantly impacts the contact angle. For instance, cellulose films exhibit limited moisture barrier performance because of the inherently hydrophilic nature of cellulose,^502^ which hinders their broad applications. To address this issue, incorporating suitable hydrophobic additives has proven to be an effective approach. As illustrated in Figure 17, commonly used hydrophobic modification additives for LCFs include wax, long-chain fatty acids, low surface energy organic compounds (e.g., PDMS), inorganic nanoparticles (e.g., SiO_2_, ZnO), lignin, among others.^503^ Additionally, various fabrication methods have been developed to manipulate the surface roughness of LCFs. Beyond conventional blending and coating techniques, approaches such as electrostatic layer-by-layer deposition, electrospinning, and chemical vapor deposition are also widely employed for hydrophobic modification in nanocomposite systems and interfacial engineering of LCFs.^342,457,504^

**Figure 17:**
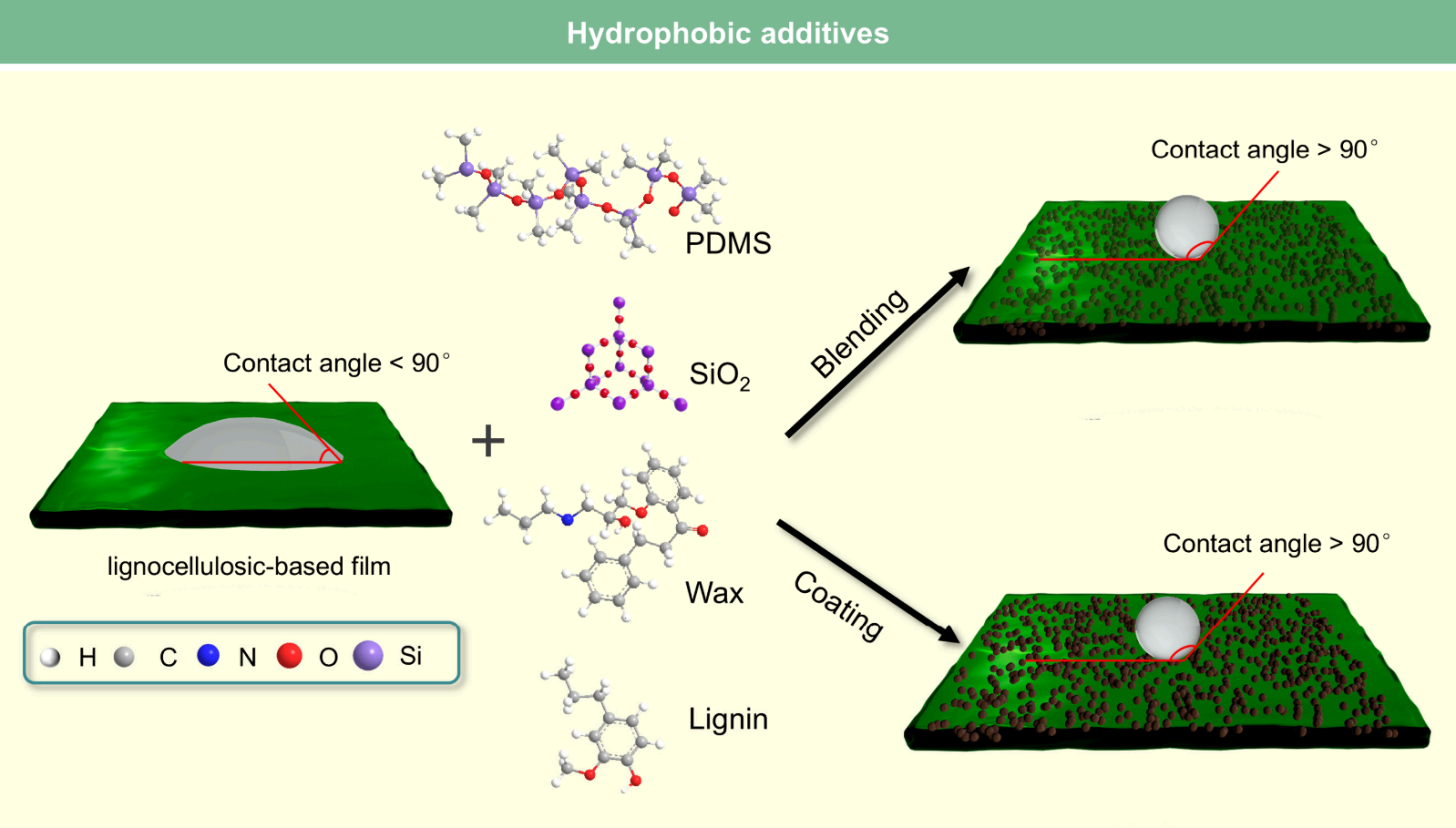
Hydrophobic additives for LCFs and their integration via blending and coating strategies.

According to the hydrophobic mechanism, wax and long-chain fatty acids typically interact with the hydroxyl groups of lignocellulose through physical adsorption, forming a hydrophobic layer composed of carbon chain. The resulting LCFs exhibit water contact angles (WCA) ranging from 90° to 120°. For example, Österberg et al.^381^ reported a fast method to improve the hydrophobicity of CNF films through surface coating with wax. These films exhibited resistance to various solvents, including water, methanol, toluene, and dimethylacetamide. Compared to untreated CNF films, the contact angle of the wax-coated CNF films significantly increased from 40° to 110°, indicating greatly enhanced hydrophobicity. Furthermore, the oxygen and water vapor barrier properties of the films were improved after the wax coating. Beeswax (BW), a food-grade wax, is of particular interest for food packaging. Indriyati et al.^505^ developed bacterial nanocellulose (BNC) films containing up to 40% BW. The addition of CMC and Tween 80 further improved the properties of the composite films. The contact angle of the BNC films was 45°, which increased to 53° with the addition of CMC, and further to 124° with 40 wt % BW. Stearic acid, known for its good biocompatibility, has been employed to enhance the moisture barrier properties of LCFs. Sebti et al.^506^ prepared HPMC/stearic acid films by incorporating stearic acid into an HPMC solution. The results showed that the addition of stearic acid increased the film’s contact angle by 40% (from 49° to 82°). Costa et al.^507^ investigated the application of a hydrophobic layer composed of calcium carbonate modified with stearic acid to prepare hydrophobic CNF films. The neat CNF films were hydrophilic, with a contact angle of approximately 45°. The application of a calcium carbonate layer modified with stearic acid, even without heat treatment, was sufficient to impart hydrophobicity to the CNF films, achieving a contact angle of 122°. Furthermore, to realize high hydrophobicity, He et al.^508^ fabricated RC/stearic acid films with rough surface structure by simple gelation and hot-pressing process. The crystallization of stearic acid was controlled within the micropores of the RC film surface and interface, leading to high WCA of 145°.

The hydrophobicity of AKD also arises from its long-chain fatty alkyl groups. Additionally, the enone dimer moiety reacts with hydroxyl groups in lignocellulose, forming stable covalent bonds. Lei et al.^509^ fabricated hydrophobic hemicellulose/montmorillonite films using AKD as a hydrophobic agent. The incorporation of AKD increased the contact angle to 121°. The hemicellulose/montmorillonite films demonstrated excellent barrier properties, highlighting their significant potential for food preservation under high-humidity conditions. Yang et al.^510^ developed water-repellent and transparent alkali/urea-RC films by immersing them in a cationic AKD dispersion followed by heating and drying. The treatment caused hydrophilic molecules on the film surface to be fully or partially covered with hydrophobic AKD components, increasing the contact angle from 50° to over 100°. Imani et al.^511^ prepared novel superhydrophobic cellulose-calcium carbonate composite films by incorporating AKD and starch. The hydrophobic functional groups and surface microstructure altered the surface chemistry of the internally sized films from hydrophilic to superhydrophobic through AKD coating and oven drying. Similarly, Nadeem et al.^512^ reported highly hydrophobic nanocellulose-based films achieved by spray-depositing AKD. The hydrophobicity of AKD-treated films was attributed to the ketoester linkages formed by the long alkyl chains of AKD on the nanocellulose surface.

Another type of hydrophobic additive includes low surface energy organic compounds, such as PDMS and methyltrimethoxysilane (MTMS). These additives typically rely on Si–O and –CH_3_ functional groups to lower the surface energy of the material. After modification with PDMS, the WCA of the LCFs can reach 110° to 130°. Chen et al.^513^ fabricated hydrophobic and transparent biodegradable cellulose-based films by combining PDMS, copper nanowires (CuNWs), and lignocellulosic nanofibers (LCNF)]. The hydrophobicity (WCA > 90°) of the cellulose-based films was due to the presence of methyl groups in the PDMS layer. These films exhibited excellent mechanical properties, transparency, and thermal insulation, making them a promising alternative to traditional transparent plastic films with significant potential for agricultural applications. Vyas et al.^514^ prepared a superhydrophobic CMC/ZnO nanoparticles (NPs) composite film by spray coating with PDMS/starch. The hydrophobic silicon groups in PDMS, combined with the increased surface roughness imparted by starch, significantly enhanced the WCAs.

Inorganic nanoparticles, such as SiO_2_, ZnO, and graphene nanosheets, can be employed to construct a rough surface on LCFs. When combined with low-surface-energy organic compounds, they can significantly enhance the hydrophobicity of LCFs. Shi et al.^515^ developed CNF-based films by incorporating SiO_2_ NPs and poly­(butylene adipate-*co*-terephthalate) (PBAT). Compared to pure CNF films, the CNF/PBAT/SiO_2_ composite films exhibited significantly enhanced hydrophobicity, with a WCA exceeding 80°. This improvement was primarily attributed to the melting of PBAT at high temperatures, which facilitated the formation of CNF/PBAT composite films with a consistent chemical composition (PBAT). Additionally, the SiO_2_ NPs enhanced the infiltration of melted PBAT into the CNF network structure. Zhu et al.^516^ fabricated hydrophobic RC films with nano-SiO_2_ and surface silanization modification. The films exhibited strong water resistance and high mechanical strength. When the nano-SiO_2_ content was 6%, the tensile stress and WCA of the composite film was 73.91 MPa and 143.8°. The good hydrophobicity was attributed to the fact that both the regenerated cellulose matrix and the nano-SiO_2_ surface were silanized during the graft modification, which reduced the surface energy and increased the hydrophobicity of the composite film.

In addition, the aromatic ring structure of lignin endows it with good hydrophobic properties. Tian et al.^517^ prepared hydrophobic cellulose/lignin composite films using a simple blending strategy. As the percentage of lignin nanoparticles and esterified lignin nanoparticles increased, the WCA also increased, reaching a maximum of 103.6° at 10% loading. In addition to their hydrophobicity, the cellulose/lignin composite films exhibited good antioxidant, UV absorption, and biodegradability, showing potential for use in both food and nonfood packaging applications.

#### 4.1.2 Plasticizers

Plasticizers are low molecular-weight compounds widely used as additives in the polymer industry. The mechanical properties, particularly the flexibility, of LCFs can be enhanced through plasticization, which involves nonchemical bonding.^518^ The addition of a plasticizer typically reduces the strong intermolecular interactions between lignocellulosic molecules, leading to decreased viscosity, deformation stress, and hardness of the LCFs.^519^ The plasticization mechanism can be explained by several theories, including the lubricity theory, gel theory, and free volume theory.^520^ Among these, the free volume theory is the most widely accepted and attempts to explain the reduction in glass transition temperature (*T*_g_) and other material properties, such as specific volume, thermal expansion coefficients, and viscosity, as plasticizer content increases.^521^ It remains the primary theory for understanding the plasticization mechanism.

Free volume refers to the internal space between polymer chains. In rigid polymers, free volume is minimal, and the molecules are densely packed between the chains. The primary function of plasticizers is to increase the free volume within the polymer by reducing intermolecular forces between chains, thereby enhancing chain mobility and rendering the polymer rubbery and flexible.^522^ According to the Williams–Landel–Ferry (WLF) approach, there is a linear correlation between fractional free volume and temperature.^523^

1 $$f=f_{0}+\beta_{f}\left(T-T_{0}\right)$$

*f* refers to the fractional free volume of a liquid, defined as *f* = *v*_1_/*v*_0_, where *v*_1_ is the free volume and *v*_0_ is the occupied volume. β is the cubic expansion coefficient. Typically, free volume originates from three main types of motion within the polymer structure: the motion of the main chain, side chains, and chain ends. Both external plasticization (the incorporation of a lower molecular weight material acting as a plasticizer) and internal plasticization (increasing the number or length of side chains) can enhance the free volume between chains.

Commonly used plasticizers for LCFs include small polar molecules (e.g., glycerol, sorbitol, urea), polymeric plasticizers (e.g., polyethylene glycol, PVA), and ionic liquids. In the case of polyol-based plasticizers, the hydroxyl groups of polyols form weak hydrogen bonds with the hydroxyl groups of lignocellulose, replacing strong intermolecular hydrogen bonds. This interaction increases the spacing between polymer chains and lowers the *T*_g_. Additionally, the polymer chains of plasticizers can form interpenetrating networks with lignocellulose through physical entanglement, further reducing intermolecular forces. Gupta et al.^524^ prepared coconut coir and groundnut shell-derived CMC film. By employing glycerol as the plasticizer, the obtained CMC film exhibited improved flexibility and extension. Benitez et al.^518^ fabricated cellulose-glycerol free-standing films using a drop-casting strategy. The films exhibited a reinforced hydrogen bond network and an amorphous molecular structure. The plasticizing effect resulted in a more flexible matrix, enhancing elongation at break and toughness, albeit at the expense of a reduced ultimate Young’s modulus and tensile strength. Paudel et al.^525^ used glycerol and sorbitol as plasticizers to overcome the brittleness of cellulose films and significantly enhance their flexibility. MCC was selected as the starting material, solubilized in ZnCl_2_, and films were formed by cross-linking the cellulose chains with calcium ions in the presence of glycerol and sorbitol. The results showed that glycerol-added films exhibited higher solubility, water absorption, and water vapor permeability (WVP) compared to sorbitol films. In contrast, sorbitol films demonstrated higher tensile strength, whereas glycerol films were more flexible. Fernández-Santos et al.^526^ investigated the effects of different additives, including sorbitol, glycerol, maltitol, xylitol, mannitol, gellan gum, and ethylene glycol, on CNC films. All additives, except ethylene glycol and mannitol, improved film elongation while also increasing tensile strength and reducing air and water permeance. Films containing sorbitol, xylitol, and maltitol exhibited the highest barrier properties, providing complete resistance to oxygen under 60% RH conditions. Interestingly, films containing additives degraded more easily than the control film. For urea, the amide groups on urea can be bonded to lignocellulose through hydrogen bonds, disrupting the original hydrogen bond network, therefore enhancing the ductility of the LCFs. Wang et al.^527^ used choline chloride/urea as a plasticizer to prepare RC films (RCF). Choline chloride/urea exhibited good compatibility with cellulose, with no chemical reaction or crystallization occurring between the plasticizer and cellulose. The incorporation of choline chloride/urea significantly improved the film-forming performance of cellulose by disrupting strong inter- and intramolecular hydrogen bonds, increasing elongation (34.9%), and reducing the tensile strength of RCF. For ionic liquids, the anions and cations of ionic liquids can form strong interactions with the hydroxyl groups of lignocellulose, disrupting the hydrogen bond network. Qiao et al.^528^ used the plasticizing-rolling method to prepare all-cellulose composite films by employing BmimCl and lithium chloride (LiCl) as plasticizers. Both the plasticization effect and the shear effect from rolling disrupted the crystalline regions of cellulose, increasing the mobility of cellulosic molecular chains. As a result, the plasticized cellulose composite films exhibited a significantly higher elongation (3.7%) compared to the untreated cellulose composite films (1.0%).

#### 4.1.3 Wet and Dry Strength Additives

##### 4.1.3.1 Wet Strength Additives

The mechanical performance of LCFs is highly sensitive to moisture due to the inherent hydrophilicity of cellulose and the possible introduction of hydrophilic groups during the preparation process. This moisture sensitivity significantly limits the applicability of LCFs in high-humidity environments.^529^ Wet strength additives are chemical agents capable of significantly improving the mechanical strength of paper or film materials under wet conditions. The primary strengthening mechanisms include: (i) Enhanced hydrogen bonding: Wet strength resins interact with hydroxyl groups on the fiber surface, promoting the formation of additional hydrogen bonds and thereby enhancing the intermolecular cohesion between fibers. (ii) Formation of water-resistant chemical bonds: Functional groups within wet strength agents chemically react with fiber surfaces, generating new water-resistant covalent bonds that improve wet strength. (iii) Creation of a waterproof protective layer: Wet-strength resins can self-cross-link to form a hydrophobic coating over the fibrous network, protecting the fibers from moisture-induced degradation. For LCFs, CS, polyamide-epichlorohydrin resin (PAE), and lignin among the most commonly used wet strength additives.^424^

Wu et al.^530^ used the synergistic hydration of CS and CNFs network to prepare shape memory CNP (Figure 18ai). The strong interactions between CS and CNFs endowed the CNP with good mechanical performance (wet tensile strength of 65 MPa) and high transparency, as well as outstanding antibacterial properties and barrier properties. Du et al.^424^ reported a facile approach to functionalize CNP by impregnating it with CS. The functionalized CNP exhibited enhanced mechanical strength in both dry and wet states. Notably, the wet strength of CNP/CS significantly increased from 6.98 to 42.76 MPa. In their subsequent study, polypyrrole (PPy) was further incorporated into CNP/CS, resulting in CNP/CS/PPy with a high wet tensile strength of 80.13 MPa (Figure 18aii).^531^ The enhanced wet strength primarily stems from the compact structure and surface hydrophobicity generated by the synergy between CS and PPy, which effectively reduces water penetration into the structure and protects most chemical bonds from disruption by water molecules. Sharma et al.^532^ investigated the physical and chemical properties of CNF films by incorporating PAE (Figure 18bi). After the cross-linking process, the surface of the CNF film transitioned from hydrophilic (contact angle of 50°) to hydrophobic (contact angle of 110°). As expected, the wet strength of the cross-linked CNF films significantly improved, increasing from 12 to 32 MPa. Yang et al.^533^ reported the fabrication of 2,2,6,6-tetramethylpiperidine-1-oxyl (TEMPO)-oxidized cellulose nanofibrils (TOCNF) films cross-linked with PAE (Figure 18bii). The resulting films exhibited high transparency (79.5% at a wavelength of 600 nm), along with excellent mechanical properties, including a tensile strength of approximately 135 MPa and a Young’s modulus of 11 GPa. Additionally, a high wet strength of 95 MPa was achieved due to the formation of a compact cross-linking network. FTIR spectra indicated that the surface carboxyl groups of TOCNF formed ester bonds with the active groups of PAE, effectively enhancing the thermal stability of the TOCNF/PAE hybrid films. In the study by Wang et al.,^240^ lignin was identified as a hydrophobic component in CNP, significantly enhancing wet tensile strength by minimizing water absorption and maintaining CNP’s structural integrity. As shown in Figure 18ci, the wet tensile strength of CNP strongly correlates with its residual lignin content. For instance, 14-CNP, which contained approximately 14% lignin, achieved a wet tensile strength of 83.5 MPa and could lift a 500 g weight in water. Wang et al.^534^ utilized activated residual lignin as a reinforcement to prepare CNP with improved wet strength and water stability (Figure 18cii). Residual lignin, a hydrophobic polymer, acted as a hydroxyl donor for hydrogen bonding, enhancing the water resistance of CNP. The wet strength of CNP was positively correlated with the residual lignin content. The results suggested that the bonding strength between the residual lignin and nanofibrils in CNP was water-stable, highlighting the strengthening role of residual lignin. Additionally, Li et al.^535^ used citric acid as a nontoxic cross-linking agent to enhance the wet strength of hemicellulose-based composite films. The free hydroxyl and carboxyl groups of citric acid provided additional binding sites, improving the wet strength of the hemicellulose films. The results clearly showed that the tensile strength and elongation at break of cross-linked hemicellulose films increased from 5.8 to 12.2 MPa. A consistently higher strength was also observed in cross-linked hemicellulose films under varying RH conditions.

**Figure 18:**
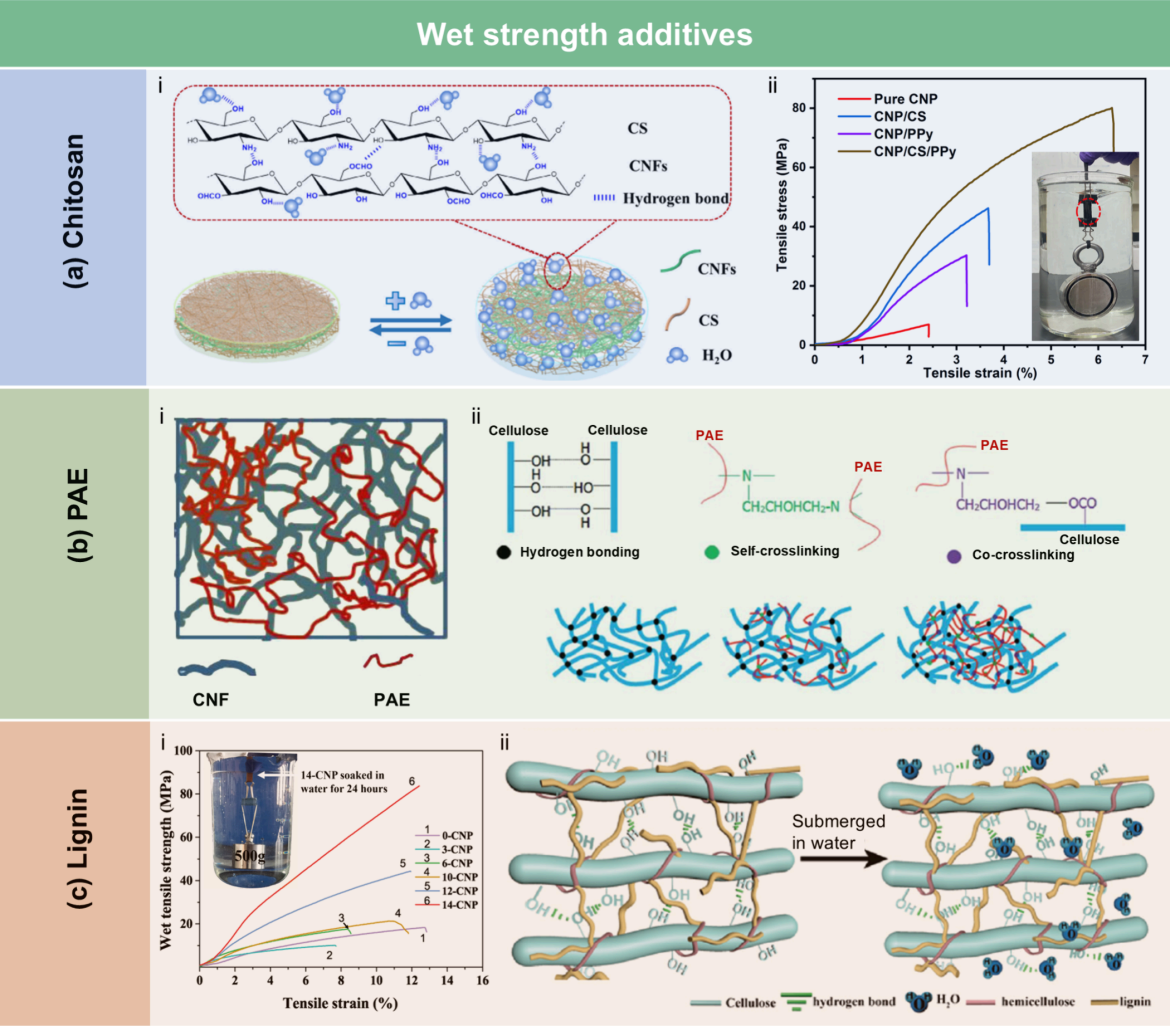
(a) Schematic diagram of the synergistic hydration of CS and the CNF network (i). Adapted with permission from ref 530. Copyright 2019 Elsevier. Stress–strain curves of pure CNP and functionalized CNP samples in the wet state (ii), with the inset illustrating that a CNP/CS/PPy strip (63 μm thick and 1 cm wide) can support a 500 g weight while submerged in water. Adapted with permission from ref 531. Copyright 2021 American Chemical Society. (b) Schematic illustration of the CNF film cross-linked by PAE (i). Adapted with permission from ref 532. Copyright 2016 American Chemical Society. Illustrating diagram of the interfibrillar hydrogen bonding in TOCNF films and the cross-linking structure of the TOCNF/PAE films (ii). Adapted with permission from ref 533. Copyright 2017 Royal Society of Chemistry under CC BY 3.0 (https://creativecommons.org/licenses/by/3.0/). (c) Stress–strain curves of CNP samples with varying lignin content in the wet state, with the inset showing that the CNP sample containing 14% lignin can lift a 500 g weight in water (i). Adapted with permission from ref 240. Copyright 2017 Royal Society of Chemistry. Schematic diagram illustrating CNP with activated residual lignin interacting with water (ii). Adapted with permission from ref 534. Copyright 2020 Royal Society of Chemistry.

##### 4.1.3.2 Dry Strength Additives

Dry strength additives mainly improve the mechanical properties of LCFs, such as tensile strength and burst strength, by increasing the effective contact area between fibers, forming chemical bonds or establishing physical entanglements. Common dry-strength additives for LCFs include carbon nanomaterials (e.g., carbon nanotubes (CNTs) and graphene),^536,537^ synthetic polymers (e.g., PVA, PAM),^538^ metal nanoparticles,^539^ among others. The main strengthening mechanisms of dry strength additives can be summarized as follows: (i) Hydrogen and ionic bonding: Polar functional groups (e.g., hydroxyl, amino) in the additives can form hydrogen bonds with lignocellulose or enhance interfiber bonding through electrostatic interactions. (ii) Formation of network structure: Carbon nanomaterials and synthetic polymers can form three-dimensional networks through physical entanglement or chemical cross-linking, which help to distribute stress and inhibit crack propagation. (iii) Interface synergistic effects: Additives such as nanoparticles can fill the voids between cellulose fibers, reducing relative fiber movement. They also serve as a protective buffer, mitigating the direct impact of external forces and thereby enhancing structural integrity.

Carbon nanomaterials not only enhance the mechanical properties of LCFs but also impart various additional functionalities (which will be discussed in detail in section 4.3.1).^544^ Huang et al.^540^ reported the preparation of cellulose/CNT nanocomposite films using solution dispersion, slow gelation, and a subsequent hot-press drying process. As shown in Figure 19ai, with a 5 wt % CNT loading, Young’s modulus and tensile strength of the cellulose nanocomposite films increased by 21% and 55%, respectively, compared to pure cellulose films. The hot-press drying and slow gelation processes effectively brought the CNTs and cellulose chains into close contact and reduced the free volume, facilitating the formation of strong interfacial hydrogen bonds between the hydroxyl groups in the cellulose chains and the residual oxygen-containing functional groups on the CNT surfaces. This interaction significantly enhanced the mechanical strength of the cellulose nanocomposite films. In another study, Zhang et al.^541^ synthesized holocellulose nanofibrils (HCNFs) with a core–shell structure to develop high-performance HCNF/CNT nanocomposite films (Figure 19aii). At a high RH of 83%, the HCNF/CNT composite film containing 20% CNT exhibited a tensile strength of 107 MPa, which was 1.8 times higher than that of the pure CNF/CNT composite film (61 MPa). The enhanced mechanical performance under high humidity conditions was attributed to hemicellulose-improved bonding in the HCNF/CNT composite films, their well-ordered structure, and the high intrinsic strength of the components. To achieve excellent optical transparency, electrical conductivity, and mechanical performance, Xiong et al.^542^ fabricated CNC-GO laminated nanocomposite films, consisting of rigid CNC networks conformally wrapped by flexible GO sheets (Figure 19bi). The combination of compliance and synergistic mechanisms between CNC and GO enhanced the mechanical performance of the films. Ampaiwong et al.^545^ prepared carboxymethyl cellulose/reduced graphene oxide (CMC/rGO) nanocomposite films with improved mechanical and electrical properties. The incorporation of GO and rGO significantly increased the tensile strength and elastic modulus of the CMC film. Notably, rGO incorporation led to greater improvements in tensile strength and elastic modulus compared to GO (Figure 19bii). This was primarily due to the stronger van der Waals interactions between rGO layers, resulting in a more closely packed structure within the films and, consequently, higher strength.

**Figure 19:**
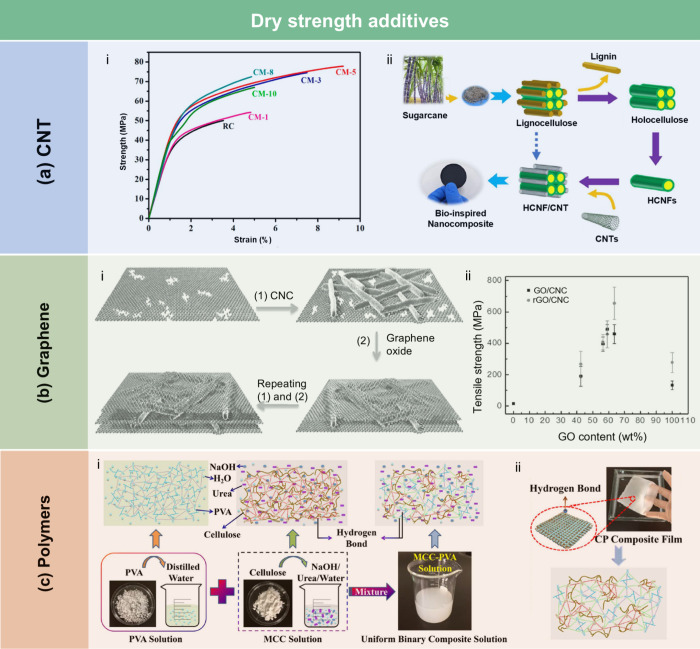
(a) Stress–strain curves of cellulose nanocomposite films and cellulose nanocomposite films/CNT as a function of CNT loadings (i). Adapted with permission from ref 540. Copyright 2015 American Chemical Society. Schematic illustration of the preparation of HCNF/CNT composite film (ii). Adapted with permission from ref 541. Copyright 2020 American Chemical Society. (b) Fabrication of the laminated CNC/GO films (i); Stress–strain curves of the CNC/GO and CNC/rGO films with different GO contents (ii). Adapted with permission from ref 542. Copyright 2015 Wiley-VCH. (c) The preparation process of binary MCC-PVA composite solution (i) and cellulose/PVA composite film (ii). Adapted with permission from ref 543. Copyright 2020 IOP Publishing Ltd.

Synthetic polymers, such as PVA and PAM have been incorporated into LCFs to enhance mechanical strength. For instance, Zhao et al.^546^ fabricated RC/PVA composite films through a controlled dissolution and regeneration process. The addition of PVA significantly improved the mechanical properties of pure cellulose films. The film containing 1% PVA exhibited the highest tensile strength of about 100 MPa, primarily due to the dense structure maintained with the appropriate PVA content, which preserved the self-assembly regularity of molecular chains. Heng et al.^544^ reported cellulose/PVA blend films prepared by phase inversion for use as supercapacitor separators (Figure 19ci). The tensile strength increased from 21 to 29 MPa, and Young’s modulus increased from 0.8 to 3 GPa as the PVA mass ratio rose from 10% to 20%. This enhancement was attributed to the formation of intra- and intermolecular hydrogen bonds between the hydroxyl groups of PVA and cellulose molecules (Figure 19cii). Kurihara et al.^547^ fabricated a TOCNF/PAM composite film by casting and drying of homogeneous mixtures of aqueous TOCNF dispersion and PAM solution. Both the Young’s modulus and tensile strength of the TOCNF film were improved by compositing with 25% PAM. The enhancement mechanism was systematically analyzed.^548^ On the one hand, formation of hydrogen bonds between TOCNF and PAM contributed to the improvement of mechanical properties. On the other hand, the formation of nematic-ordered domain structures of TOCNF in aqueous dispersions therefore probably played a significant role in the reinforcing effects.

### 4.2 Chemical Modification

Beyond the use of wet-strength agents, chemical modification presents a powerful strategy for enhancing the water resistance of LCFs. The underlying chemistry of cellulose derivatization is outlined in Figure 12 in section 2.3.6. This section focuses on the practical application of various chemical modification techniques, such as esterification, silylation, carbamylation, and graft polymerization, to improve the hydrophobicity and water resistance of LCFs, as illustrated in Figure 20.

**Figure 20:**
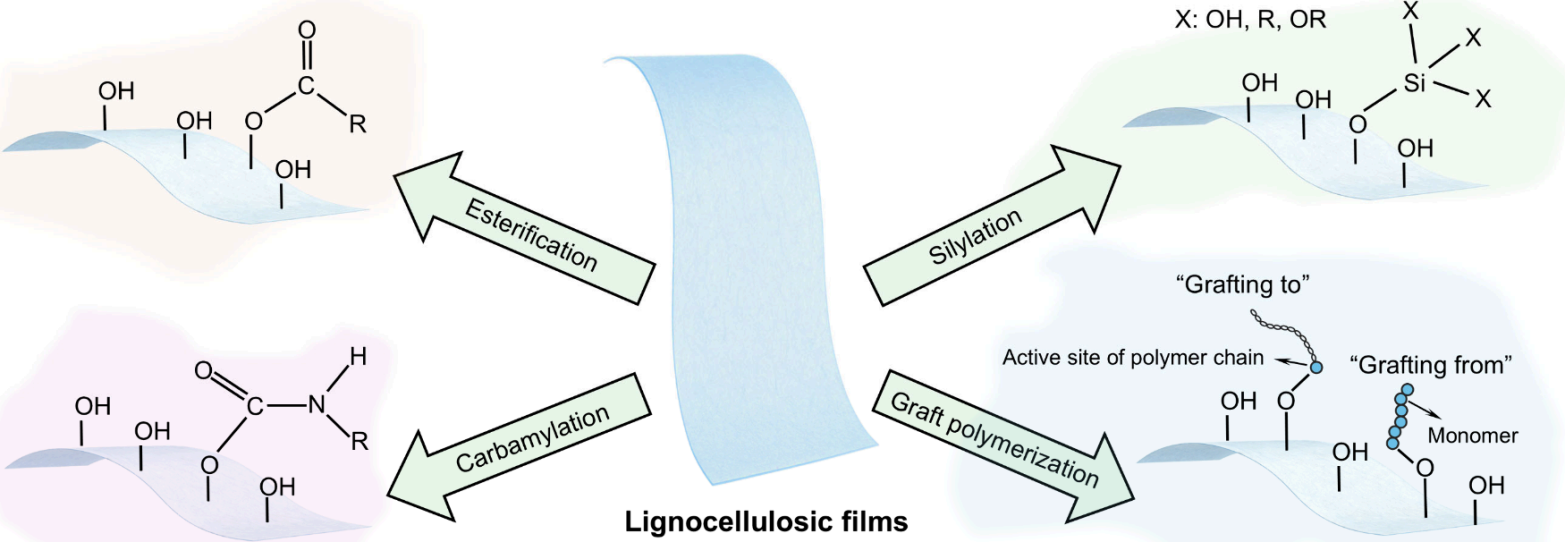
Schematic illustration of chemical modification of LCFs, including esterification, silylation, carbamylation, and graft polymerization.

#### 4.2.1 Esterification

Esterification substitutes cellulose hydroxyl groups with ester moieties, thereby enhancing the hydrophobicity of LCFs in direct relation to the substituents’ chain length and nonpolarity.^549^ Short-chain esters, such as acetate, have been widely employed to improve water resistance.^550^ For example, Salem et al.^551^ modified cellulose micro- and nanofibril (CMNF) films through acetylation, demonstrating that precise control over both fibrillation and acetylation levels significantly enhanced hydrophobicity and resistance to water vapor, air, oil, and grease. The incorporation of longer alkyl chains (C8–C18) using appropriate acylating agents further amplifies hydrophobicity and barrier performance by introducing extended nonpolar segments along the cellulose backbone.^552–555^ For instance, developed hydrophobic CNP through a mild esterification process, modifying CNF with anhydrides of varying alkyl chain lengths to reduce hygroscopicity. The modified nanopaper exhibited a significant increase in WCA from 24° (unmodified) to 118°, a remarkable reduction in water absorption, and improvements in wet tensile strength (7-fold increase) and stiffness (24-fold increase), outperforming unmodified CNF films. In another study, Deng et al.^555^ employed ball milling-assisted acylation modification of MFC to prepare highly hydrophobic and mechanically superior cellulose films. Through acylation with hexanoyl chloride (HC), simultaneous delamination and surface modification of MFC were achieved, significantly improving the tensile strength (140 MPa) and elongation at break (21.3%) of the films while increasing the WCA from 50° to ∼105°. The mechanochemical modification mechanism induced by ball milling generated shear forces that not only disrupted MFC aggregates but also facilitated the esterification reaction. The resulting films exhibited excellent water resistance, with a reduction in WVP to 0.65 × 10^3^ g·mm/(m^2^·day·atm). Fluorinated and aromatic esters have also been explored for hydrophobic modification of LCFs. For example, Rodionova et al.^556^ significantly enhanced the hydrophobicity and barrier properties of MFC films through gas-phase esterification using trifluoroacetic anhydride (TFAA), acetic acid (AcOH), and acetic anhydride (Ac_2_O). This process selectively esterified accessible hydroxyl groups while preserving the crystalline structure of cellulose. Following the modification, the WCA increased from 41.2° (untreated MFC) to 96.9° (TFAA/Ac_2_O-treated MFC), indicating a substantial enhancement in water resistance.

The efficiency of cellulose esterification depends largely on the source and structure of the cellulosic substrate as well as the choice of reaction medium and catalysts.^557–559^ The degree of substitution (DS) is the most widely used metric for evaluating the extent of chemical modification in lignocellulosic biomass.^560^ It refers to the average number of −OH groups substituted per anhydroglucose unit, with a theoretical maximum of 3.^549^ A higher DS generally corresponds to increased hydrophobicity. However, the actual DS may be limited by the steric hindrance of bulky substituents or restricted substrate accessibility.^561–563^ Wang et al.^564^ utilized lipase-catalyzed esterification to modify NFC films with dimethyl adipate (DA) and subsequently coated them with silver nanowires (AgNWs) to produce highly hydrophobic NFC films, as shown in Figure 21ai. Enzymatic catalysis enhanced the DS (NMR = 0.18, three times higher than NFC-DA samples), increasing the WCA from 50° to 105°, significantly enhancing hydrophobicity. Lakovaara et al.^552^ employed a deep eutectic solvent (DES) system composed of triethylmethylammonium chloride (TEMACl) and imidazole as a reaction medium for esterification with *n*-octenyl succinic anhydride (OSA), successfully improving the performance of CNF and all-cellulose composite (ACC) films. After modification, CNF-DES/OSA films exhibited higher mechanical strength in dry, humid, and wet conditions, with wet tensile strength increasing to 31 MPa and elongation at break reaching 6.1%, significantly higher than unmodified CNF (18 MPa, 2.2%). Moreover, the WCA increased from 37° (CNF) to 51° (CNF-DES/OSA), indicating improved hydrophobicity. Similarly, Chen et al.^553^ used the same DES system for esterification of cellulose with dodecenyl succinic anhydride (DDSA) to obtain hydrophobic CNF, as shown in Figure 21aii. A high-strength water-plasticized film was subsequently fabricated through vacuum-assisted filtration, achieving a dry tensile strength of 150.8 MPa and a wet tensile strength of 94.8 MPa, with excellent water resistance and a maximum contact angle of 153°. These films demonstrate water plasticity and could be shape-programmed using kirigami and origami techniques. Furthermore, both the films and the solvent system were recyclable, enhancing their sustainability. In addition, other parameters such as reaction time, temperature, catalyst concentration, and structural characteristics of lignocellulose also strongly influence esterification efficiency.^565–570^

**Figure 21:**
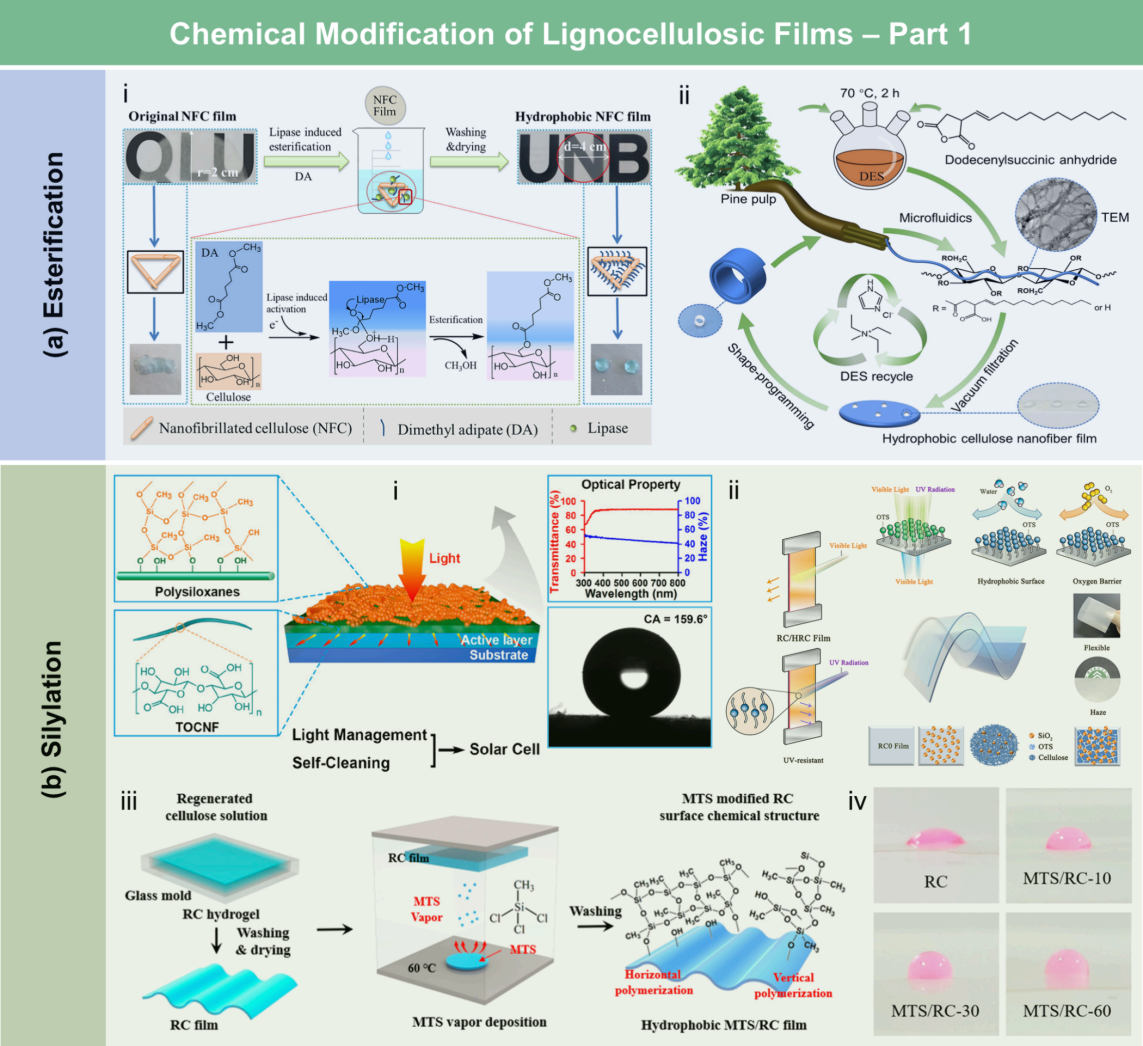
(a) Schematic illustration depicting the formation of a highly hydrophobic NFC film catalyzed by lipase (i). Adapted with permission from ref 564. Copyright 2022 Elsevier. Illustrative schematic outlining the process used to produce hydrophobic cellulose nanofibers and hydroplastic cellulose-based films (ii). Adapted with permission from ref 553 Copyright 2023 Elsevier. (b) Schematic of hazy and highly transparent cellulose nanopaper depicting a superhydrophobic self-cleaning surface (i). Adapted with permission from ref 571. Copyright 2018 American Chemical Society. Surface modification strategy for RC nanocomposite films to achieve enhanced mechanical strength, UV resistance, and hydrophobicity (ii). Adapted with permission from ref 516. Copyright 2023 MDPI under CC BY 4.0 (https://creativecommons.org/licenses/by/4.0/). Fabrication of the hydrophobic MTS/RC films through chemical vapor deposition (CVD) (iii) and the hydrophobic characteristics of the neat RC film and the RC/MTS films (iv). Adapted with permission from ref 572. Copyright 2023 MDPI under CC BY 4.0 (https://creativecommons.org/licenses/by/4.0/).

#### 4.2.2 Silylation

Silylation of lignocellulosic biomass proceeds in aqueous conditions, wherein hydroxyl and carboxyl groups react with silyl groups (R_3_Si−) to form covalent bonds.^573^ The structure of grafted silanes depends on the number of hydrolyzable substituents (−OR, −Cl, −N­(CH_3_)_2_) and the specific reaction conditions.^574,575^ Silylation has been demonstrated to effectively enhance the hydrophobicity of LCFs. For example, Chen et al.^571^ combined TOCNF with polysiloxane through vacuum filtration and silanization, resulting in a highly transparent (90.2%), highly hazy (46.5%), and superhydrophobic (contact angle of 159.6°) nanocellulose paper, as shown in Figure 21bi. Polysiloxane modification significantly enhanced the water repellency and toughness of the nanopaper (118.7% increase). This nanopaper exhibited dual functionality: it improved solar cell efficiency by 10.43% through light management while possessing self-cleaning properties that removed surface dust and restored most of the photovoltaic performance loss. Using roll-to-roll plasma deposition, researchers modified CNF films with hexamethyldisiloxane (HMDSO).^576^ After modification, the WCA of CNF films increased from 23° to 103°, and their relative polarity decreased to 0.6% (compared to 46.8% for unmodified films). Additionally, the modified CNF films demonstrated excellent resistance to penetration by olive oil, *n*-heptane, castor oil, toluene, and their mixtures. Zhu et al.^516^ fabricated high-strength, UV-resistant, and hydrophobic RC (HRC) nanocomposite films through nanostructured SiO_2_ reinforcement and octadecyltrichlorosilane (OTS) surface silanization, as shown in Figure 21bii. The optimized film with 6% nano-SiO2 content exhibited a 41.2% growth in tensile strength (77.22 MPa) and an elongation at break of 14%. After OTS modification, the HRC film achieved a WCA of 143.8°, with a significant reduction in water absorption (from 104.3% for unmodified RC to 2.4% for surface-silanized RC), a UV-blocking efficiency exceeding 95%, and an oxygen barrier performance of 5.41 × 10^–11^ mL·cm/m^2^·s·Pa, and a tensile strength of 73.91 MPa. The researchers emphasized that the properties of modified RC films were strongly influenced by the OTS-to-*n*-hexane ratio, with the best performance observed at a ratio of 6 for OTS:*n*-hexane. Moreover, the film was fully biodegradable in soil, demonstrating its potential for sustainable packaging applications. Kwon et al.^572^ employed a simple one-step CVD process to modify RC films with methyltrichlorosilane (MTS), achieving excellent hydrophobicity and barrier properties (Figure 21biii). The MTS-modified RC (MTS/RC) film exhibited a high tensile strength of 146 MPa, an optical transmittance of 87% at 550 nm, and significantly enhanced hydrophobicity, with a WCA of 83.2° (Figure 21biv). Furthermore, its OTR and WVTR were reduced by 80% and 63%, respectively, reaching 3 cm^3^/m^2^·day and 41 g/m^2^·day. In another study, researchers used plasma-enhanced CVD (PECVD) and ascorbic acid as a coagulation bath to fabricate RC films with hydrophobic barrier functionality.^577^ By varying the deposition time (300, 600, 900, 1200 s) and HMDSO:O_2_ ratio (1:1, 1:2, 2:1), they achieved OTR of 31.21 cm^3^/(m^2^·day) and WVTR of 27.24 g/(m^2^·day), which were improved by 17-fold and 20-fold, respectively. The WCA increased from 84.5° (unmodified film) to 116.7°. A one-step method integrating fibrillation and hydrophobization was employed to prepare MFC by combining high mechanical ball milling with 3-aminopropyltriethoxysilane (APTES) modification.^578^ This method produced a synergistic effect on MFC fibrillation. The modified transparent MFC film exhibited a significantly increased WCA: for films with 2.4 mL APTES and 24-h ball milling, the WCA reached 133.2 ± 3.4°. Compared to unmodified MFC films, those modified with 4.7 mL APTES and 8-h ball milling showed substantial improvements in mechanical properties, with Young’s modulus increasing from 64.1 ± 4.6 MPa to 1,194.5 ± 130.6 MPa and tensile strength rising from 0.3 ± 0.1 MPa to 35.6 ± 2.1 MPa. Additionally, studies demonstrated that carboxymethyl hemicellulose modified with aminopropyl polyhedral oligomeric silsesquioxane (POSS-NH_2_) could form optically transparent composite films with improved thermal stability (*T*_onset_ > 260 °C) and glass transition temperature (*T*_g_ > 130 °C).^579^ For composite films containing 20 wt % POSS-NH_2_, tensile strength increased by 18.8% compared to carboxymethyl hemicellulose films, while water vapor barrier performance improved 2-fold (18.82 g·m^–2^·h^–1^).

#### 4.2.3 Carbamylation

Carbamylation involves the reaction of cellulose hydroxyl groups with isocyanates to form urethane (Figure 20).^549^ This chemical modification significantly enhances the hydrophobicity and water resistance of LCFs. Missoum et al.^584^ grafted NFC with octadecyl isocyanate at molar ratios of 1, 10, and 30 equiv, followed by film formation via casting. At low and high reagent levels, the modification was predominantly confined to the surface, whereas 10 equiv enabled penetration into the bulk. The resulting films exhibited strong hydrophobicity, with contact angles of ∼90° for 1 and 30 equiv, and a slightly lower angle (∼80°) for 10 equiv, reflecting variationss in chain organization. In another study, de Souza et al.^585^ modified NFC films with blocked isocyanates by casting and thermal deblocking. The treatment preserved nanofibril morphology while facilitating urethane formation. After modification, the films showed a substantial increase in hydrophobicity, with contact angle rising from ∼28° for neat NFC to ∼97° after modification. Chen et al.^580^ developed superhydrophobic cellulose nanofiber (CNF)-SiO_2_ composite films and coatings through a one-step mechanochemical approach, incorporating 3,4-dichlorophenyl isocyanate (DCPI) with SiO_2_ nanoparticles via ball milling. The resulting hydrophobic films achieved a WCA of 158°, demonstrating remarkable water resistance. As illustrated in Figure 22ai, the ball milling process facilitated both CNF fiber dissociation and uniform dispersion of SiO_2_ nanoparticles, ensuring the formation of a stable composite material. Authors coated this nanocomposite on the paper surface, which exhibited a WCA of 155.8°, significantly enhancing its water-repellent properties (Figure 22aii). To improve the application value of lignin-rich agricultural waste, Chen et al.^581^ performed a low-concentration alkaline pretreatment on bamboo powder, followed by carbamylation modification with 3,4-dichlorophenyl isocyanate (DCPI), successfully producing carbamylated lignin-containing cellulose nanofibers (CLCN) and fabricating CLCN films, as shown in Figure 22aiii. The resulting CLCN films exhibited high optical transparency (86% transmittance at 550 nm), high haze (>80%), and excellent UV-blocking capability (>90%). Additionally, these films demonstrated enhanced hydrophobicity (WCA = 86.4°) and outstanding mechanical characteristics, with a tensile strength of 83.26 MPa. Josey et al.^582^ successfully prepared hydrophobic films and moisture-resistant coatings by functionalizing xylan extracted from dissolving pulp waste liquor using octyl isocyanate in DMSO, achieving a hydroxyl conversion rate of 79%. The resulting xylan-based carbamate exhibited good solubility in chloroform and thermoplastic properties, making it suitable for coating applications. The fabricated hydrophobic film that was coated on the cellulose sheet, demonstrated excellent water resistance, with a WCA of 104°, and significantly reduced the WVTR to 31.5 g/m^2^·day. As shown in Figure 22aiv, contact angle analysis confirmed the enhanced hydrophobicity of cellulose substrates coated with the modified xylan material, demonstrating significantly improved moisture resistance compared to uncoated control samples.

**Figure 22:**
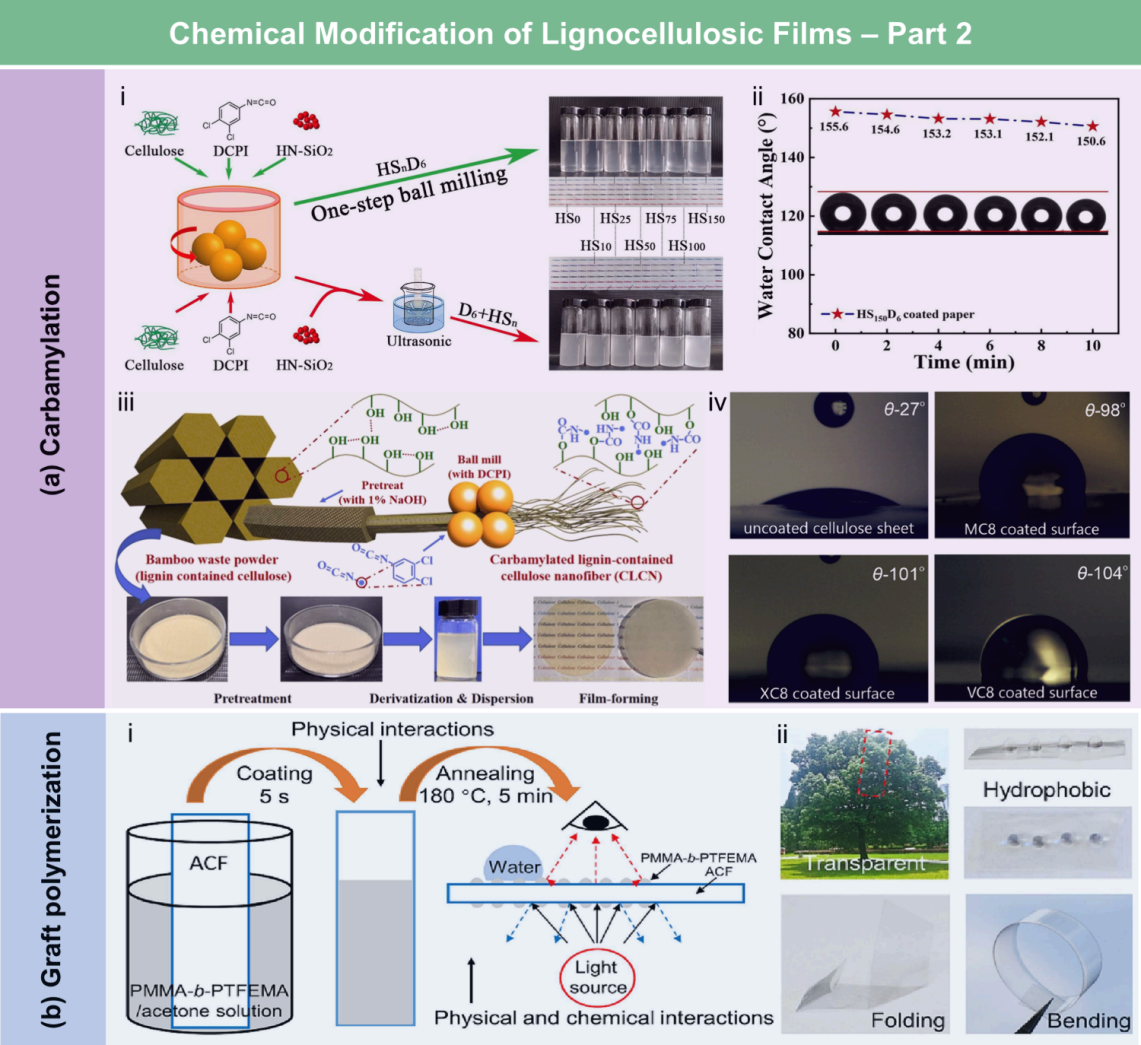
(a) Diagram illustrating the ball milling-based fabrication of hydrophobic CNF/HN-SiO_2_ composites (i), along with the variation in water contact angle (WCA) of paper coated with HS_150_D_6_ (ii). Adapted with permission from ref 580. Copyright 2022 MDPI under CC BY 4.0 (https://creativecommons.org/licenses/by/4.0/). Process of preparing hydrophobic carbamylated lignin-containing cellulose nanofiber containing lignin (CLCN) by ball milling and its films (iii). Adapted with permission from ref 581. Copyright 2024 Elsevier. Analysis of contact angle by a DI water droplet on cellulose coated with XC8, MC8, VC8 (MC8, mannose-C8; XC8, xylose-C8; VC8, viscose-C8; xylan obtained from viscose waste) (iv). Adapted with permission from ref 582. Copyright 2024 Springer Nature under CC BY 4.0 (https://creativecommons.org/licenses/by/4.0/). (b) Diagram depicting the fabrication pathway of a hydrophobic and transparent film (i); Photographs of the films (ii). Adapted with permission from ref 583. Copyright 2023 Elsevier.

#### 4.2.4 Graft Polymerization

Polymer grafting onto lignocellulosic surfaces can be achieved by either “grafting-to” or “grafting-from” approaches (Figure 20).^586–589^ In the “grafting-to”, preformed polymers are covalently attached through reactive end groups, although this approach typically results in low grafting density. In contrast, the “grafting-from” strategy initiates polymerization directly from hydroxyl groups, enabling higher density.^590–592^ Several studies have demonstrated that graft polymerization effectively improves the hydrophobicity and water resistance of LCFs. Salmieri et al.^593^ modified methylcellulose (MC) films by grafting trimethylolpropane trimethacrylate (TMPTMA) using γ-radiation. The optimal formulation (0.1% TMPTMA, 5 kGy) yields notable improvements in tensile strength (47.9 MPa) and modulus (1,792 MPa), while lowering water vapor permeability by approximately 12%. Further reinforcement with nanocrystalline cellulose (up to 1%) led to additional benefits, reducing WVP by 24% and significantly enhancing mechanical performance. By using a coating-annealing approach, Chen et al.^583^ fabricated anisotropic cellulose films that were hydrophobic, highly transparent, and mechanically durable. In this process, poly­(methyl methacrylate)-*block*-poly­(trifluoroethyl methacrylate) (PMMA-*b*-PTFEMA) was grafted onto RC films through hydrogen bonding and transesterification. The resulting films exhibited excellent optical transparency (92.3% transmittance at 550 nm), good hydrophobicity (contact angle ∼96°), and outstanding mechanical characteristics, with a tensile strength of 198.7 MPa in the dry state and 124 MPa in the wet state. As illustrated in Figure 22bi, the schematic diagram demonstrates how the coating-annealing strategy optimizes film performance, while Figure 22bii showcases the film’s flexibility, allowing it to be folded and bent while maintaining transparency and durability. Yao et al.^594^ modified cellulose films by oxygen plasma activation followed by condensation grafting with trisilanolisobutyl-polyhedral oligomeric silsesquioxane (TS-POSS). This treatment activated surface hydroxyl groups, lowered surface energy, and increased nanoscale roughness, resulting in a superhydrophobic film with a static water contact angle of 152.9°. Huh et al.^595^ developed superhydrophobic cellulose membranes by covalently grafting PVDF via RAFT/MADIX polymerization. The grafting process generated uniform PVDF nanostructures, significantly roughening the membrane surface and yielding a stable water contact angle of approximately 155°. Compared with PVDF-coated membranes, the grafted membranes retained superhydrophobicity and exhibited high oil/water separation efficiency (>97%) even after extended cycling and immersion tests, demonstrating their superior durability.

### 4.3 Functionalization with Nanomaterials

The integration of nanomaterials with lignocellulose represents a promising strategy for creating advanced composite films with enhanced functionalities.^596,597^ Functionalization of LCFs using some nanomaterials, including carbon nanomaterials, metal nanoparticles, metal-oxide nanoparticles, and two-dimensional (2D) nanomaterials, enables significant enhancements in properties such as mechanical strength, UV shielding, antibacterial activity, and electrical conductivity. These functionalized composites are tailored for a wide range of applications, including flexible electronics, food packaging, water treatment, biomedicine, energy storage, and plant growth.^598^ This section provides an overview of the functionalization strategies for lignocellulose films and highlights their applications across different fields.

#### 4.3.1 Carbon Nanomaterials

Carbon nanomaterials, characterized by their nanoscale structures, have garnered significant interest in materials science, nanotechnology, and biomedicine due to their distinctive properties.^599^ These materials can be categorized based on their spatial structure: zero-dimensional carbon nanomaterials (such as carbon quantum dots (CQDs) and fullerenes), one-dimensional nanomaterials (including CNTs and carbon nanofibers), two-dimensional materials (such as graphene and its derivatives, including GO and reduced graphene oxide (rGO)), and three-dimensional materials (like nanodiamonds).^600^ Recent advancements in the study of lignocellulosic materials, carbon nanomaterials, and supramolecular chemistry engineering have led to the development of hybrid composites.^112,601^ These composites harness the advantageous properties of both materials above, resulting in enhanced physical and chemical characteristics and broadening their potential applications.^600,602^

Carbon-based nanofillers dramatically alter LCF properties through their inherent high aspect ratios and carbon networks. In general, one- and two-dimensional allotropes (CNTs, graphene) provide exceptional electrical and thermal conductivities, while zero-dimensional forms (CQDs, fullerenes) are better known for optical functionality. While numerous studies have demonstrated the successful fabrication of LCF-carbon nanomaterial composites,^603^ a systematic comparison is crucial for guiding material selection toward specific functional targets. Table 5 compares key metrics of these nanomaterials in LCFs, including electrical/thermal conductivity, dispersion behavior, mechanical reinforcement, and matrix compatibility. Electrical and thermal conductivities are highest in CNTs and graphene due to continuous π-networks. However, CNTs/graphene strongly agglomerate without surface treatment or surfactants. 0D CQDs and fullerenes have lower conductivity and provide minimal tensile reinforcement, but they disperse readily and contribute useful optical/antioxidant functionality. Nanodiamonds are insulating electrically but exceptionally hard and thermally conductive. In practice, chemical modification of these materials is key for their functionalization as hybrid films, bioplastics, and gels.^596,604^ For example, GO with −COOH/–OH group mixes into cellulose but must be reduced to restore conductivity, and CNTs often require carboxylation or dispersion by nanocellulose to form percolating networks. Functional suitability thus depends on the application: CNTs/graphene for conductive or EMI-shielding films, CQDs/fullerenes for UV/photoluminescence or antioxidant roles, and nanodiamonds for thermal management or stiff, wear-resistant composites. Below are some research advances of these carbon nanomaterials for realizing LCF functionalization.

**Table 5:** Comparative Properties of Carbon Nanomaterials in Lignocellulosic Films

nanomaterial	electrical conductivity	thermal conductivity	dispersion behavior	mechanical reinforcement	matrix compatibility	ref
fullerenes	insulating/low	low	good	none	poor	^605^
CQDs	low–moderate	low	excellent	poor	good	^606^
CNTs	very high (metal-like)	very high	poor	excellent	moderate	^607,608^
graphene	extremely high	extremely high	poor	excellent	moderate	^609^
nanodiamonds	insulating/low	very high	moderate	excellent	good	^610^

Fullerene, as a zero-dimensional carbon nanomaterial, boasts a highly symmetrical structure where carbon atoms are tightly bound by covalent bonds, forming a resilient cage structure.^617^ This configuration imparts exceptional structural, chemical, and thermal stability. Moreover, the surface of fullerene molecules can be functionalized with a variety of groups, making them highly suitable for applications in drug delivery, diagnostics, imaging, and biosensing (Figure 23ai).^605,618^ Recent research has focused on combining lignocellulosic materials with fullerenes to create biobased films, leveraging their electrochemical and photoelectrocatalytic properties. For example, Milano et al. explored the assembly of Langmuir hybrid films using cationic fullerene derivatives and anionic CNCs through electrostatic interactions (Figure 23aii).^611^ The resulting film-modified ITO electrode exhibited an anodic photocurrent intensity of 230 nA/cm^2^, which is four times higher than that of a single fullerene derivative, demonstrating the potential of nanocellulose/fullerene hybrid films as photoelectrocatalytic materials. Similarly, Sawalha et al. prepared Langmuir hybrid membranes using anionic CNCs and cationic fulleridine, enhancing electrochemical performance and demonstrating that air/water interface interactions facilitate the assembly of C60/CNC membranes.^605^ CQDs, another zero-dimensional carbon nanomaterial, possess excellent optical properties and biocompatibility. These quasi-spherical carbon nanoparticles exhibit high surface area, low toxicity, and good water solubility.^619,620^ These attributes make CQDs ideal for integration with lignocellulosic materials to fabricate biobased films for applications such as food packaging and spoilage detection. For instance, Ezati et al. synthesized nitrogen-doped CQDs from glucose via a hydrothermal method and combined them with cellulose nanofibers to create composite films with high UV shielding and antioxidant activity, extending the shelf life of citrus fruits by more than 10 days.^621^ Additionally, Si et al. developed a biobased film using CQDs derived from lignin and CNF for detecting biogenic amines produced during food spoilage. This film exhibited a detection limit as low as 0.045 μM, enabling real-time visual monitoring of food spoilage.^622^ These studies highlight the significant potential of hybrid composites of lignocellulosic materials and carbon nanomaterials in various advanced applications, particularly in enhancing the functionality and performance of biobased films.

**Figure 23:**
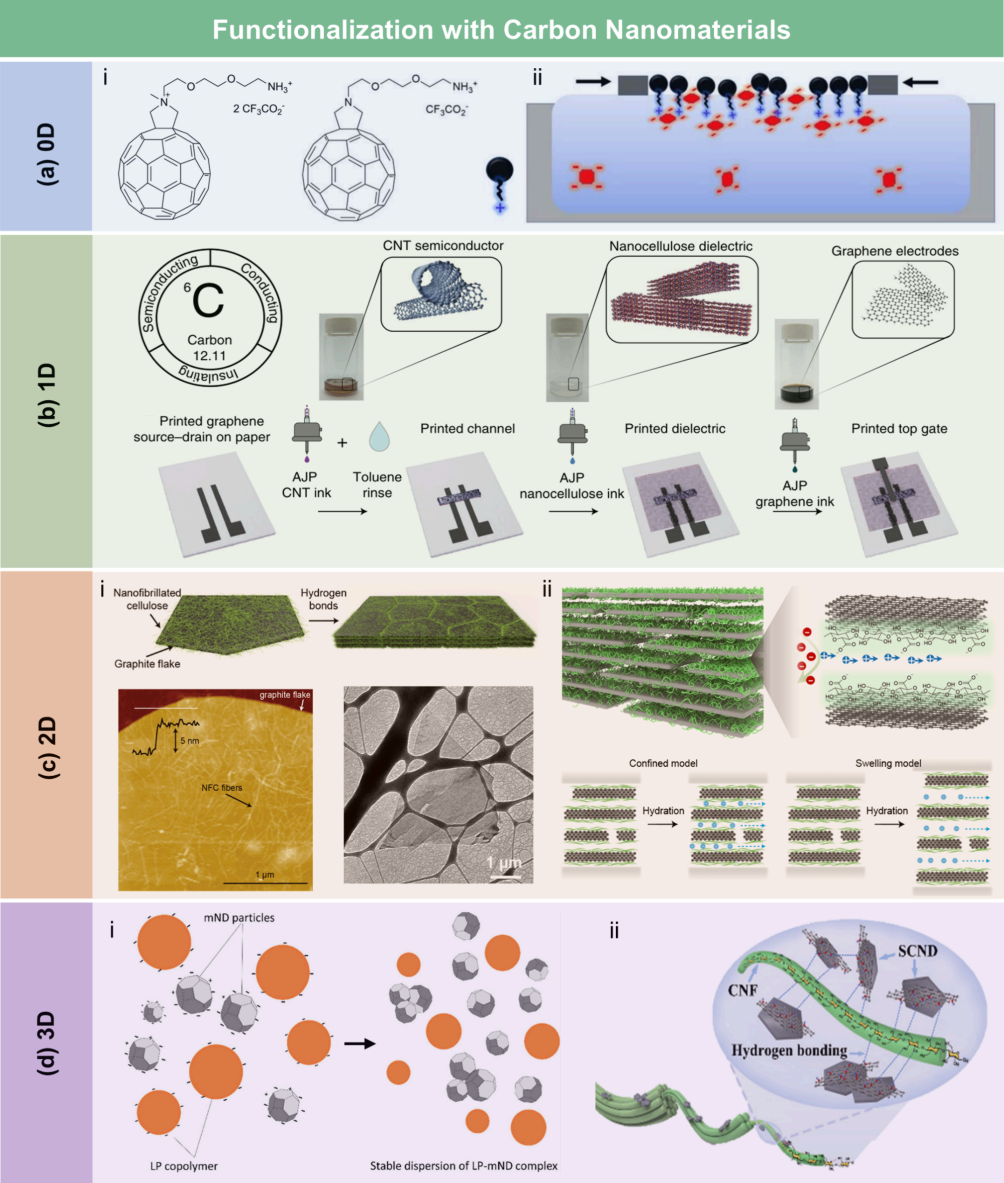
Functionalization of LCFs with carbon nanomaterials. (a) Chemical structures of the fullerene derivatives (i). Adapted with permission from ref 605. Copyright 2020 Elsevier. Schematic of the cationic fulleropyrrolidine floating layer compressed in the Langmuir trough to the same target surface pressure using CNCs/meso-tetraphenylporphyrine-4,4′,4′′,4′′′-tetrasulfonate blend as subphases (ii). Adapted with permission from ref 611. Copyright 2021 MDPI under CC BY 4.0 (https://creativecommons.org/licenses/by/4.0/). (b) Printing fabrication schematic with all-carbon inks. Adapted with permission from ref 612. Copyright 2021 Springer Nature. (c) Illustration depicting the interaction between CNF nanofibers and graphite flakes through the bonding of their hydrophobic domains and the microscopic morphology of graphite/CNF composite (i). Adapted with permission from ref 613. Copyright 2019 Elsevier. Schematic of the proposed microstructure for the printed graphite-CNF composite and schematics of the nanofluidic channels of the graphite-CNF membrane in the confined hydrated state and the swelling state (ii). Adapted with permission from ref 614. Copyright 2019 American Chemical Society. (d) Lignin-poly­(ethylene glycol) (LP)-furnace nanodiamond (mND) as both LP and mND particles repel each other to establish a well-dispersed complex (i). Adapted with permission from ref 615. Copyright 2021 Elsevier. Schematic of the hydrogen bonding interaction between CNF and single-crystal nanodiamond (ii). Adapted with permission from ref 616. Copyright 2021 Royal Society of Chemistry.

CNTs are one-dimensional carbon-based nanomaterials characterized by their tubular nanostructure. They can be classified into two primary categories: single-walled carbon nanotubes (SWCNTs) and multiwalled carbon nanotubes (MWCNTs).^623,624^ Their exceptional electrical conductivity, thermal conductivity, mechanical strength, and permeability have led to extensive research and development in various fields such as energy storage and conversion, water decomposition, nanodevices, environmental chemistry, catalysis, biosensors, and biotherapy.^599^ The incorporation of CNTs into LCFs imparts new functionalities and properties, greatly expanding their potential applications. For instance, Williams et al. employed CNF as a dielectric material, CNTs as a semiconductor, graphene as a conductor, and paper as a substrate to fabricate a printable gate dielectric film (Figure 23b).^612^ This film exhibited frequency-independent dielectric behavior and could be produced at room temperature. In another study, Zhang et al. developed a highly conductive and flexible film by creating a semi-interpenetrating network structure using multiwalled CNTs and CNFs. The resulting film demonstrated exceptional electrical conductivity of up to 37.6 S/m and maintained its performance stability even under various bending conditions.^625^

Graphene, a two-dimensional material consisting of a single layer of carbon atoms, exhibits exceptional electrical conductivity, thermal conductivity, and mechanical strength.^626,627^ GO, derived from graphene, features abundant oxygen functional groups such as hydroxyl, carboxyl, and epoxy groups, granting it favorable water solubility, biocompatibility, and surface modification capabilities.^628^ rGO, obtained through chemical or thermal reduction methods, exhibits intermediate electrical conductivity and mechanical properties, bridging the gap between graphene and GO.^629^ These two-dimensional carbon nanomaterials hold significant promise for functional applications in LCFs. For example, Zhou et al. dispersed graphite flakes and CNF in water at room temperature to create a stable and uniform solution with a high solid concentration (20 wt %) (Figure 23ci).^613^ They showcased that this slurry can be utilized on a large scale for printing, enabling the production of graphite-CNF films with excellent tensile strength (∼1.0 GPa) and toughness (∼30.0 MJ/m^3^). In another study from the same team, the researchers devised a nanofluid structure resembling nanographite, where nanometer-thick graphite sheets are encased by negatively charged CNF fibers, forming multiple two-dimensional confined spaces serving as nanochannels for rapid cation transport (Figure 23cii).^614^ This graphite-NFC configuration showcases an extremely low electrical conductivity (σ_e_ ≤ 10^–9^ S/cm). The developed charge-selective conductor exhibits notable features of high ionic conductivity and low electrical conductivity.

Nanodiamonds are essentially three-dimensional materials that combine the exceptional properties of diamonds, such as hardness, wear resistance, and chemical stability.^630^ These attributes make nanodiamonds highly promising for applications in lignocellulosic film materials.^631^ Yew et al. developed a tunable lignin PE glycol/nanodiamond composite that exhibited superior UV filtering capability, significantly increasing the sun protection factor from 29 to 89 (Figure 22di).^615^ Gong et al. utilized vacuum-assisted filtration to create a highly thermally conductive, flexible CNF/nanodiamond composite film (Figure 23dii).^616^ This composite demonstrated a thermal conductivity approximately 145.6 times higher than that of pure CNF film (76.23 W·m^–1^·K^–1^), while also maintaining excellent flexibility and mechanical strength. Additionally, Morimune-Moriya et al. prepared a composite film using cationic CNF and anionic nanodiamond, resulting in enhanced Young’s modulus and tensile strength, which increased from 9.8 GPa and 209.5 MPa to 16.6 GPa and 277.5 MPa, respectively.^632^

In summary, carbon nanomaterials offer substantial potential for enhancing the functionality of LCFs through strategic composite design and selection. These materials can significantly improve film properties such as electrical conductivity, mechanical strength, thermal conductivity, and functional versatility, thereby expanding their applications in electronics, energy storage, environmental remediation, and biomedicine. However, a critical challenge across all carbon nanomaterials is simultaneously achieving uniform dispersion and strong interfacial adhesion within the lignocellulosic matrix, which is similar to polymer materials.^633^ Hydrophobic materials like pristine graphene and CNTs require surface functionalization or the use of dispersing agents, whereas hydrophilic materials like GO and CQDs integrate more readily. Future research should focus on optimizing performance, developing novel functionalities, and ensuring sustainability in practical applications. These approaches would unlock the full potential of carbon nanomaterial-lignocellulose composites, offering innovative solutions for advancing technology and promoting sustainable development.

#### 4.3.2 Metal Nanomaterials

Metal nanoparticles possess unique properties stemming from their size and surface effects, such as high specific surface area, strong surface plasmon resonance, and distinctive optical, electrical, and magnetic characteristics. These attributes make them highly valuable in diverse fields including catalysis, electronics, biomedicine, environmental protection, and sensing applications.^634^ In recent years, to enhance the mechanical properties, UV shielding, thermal stability, and antibacterial functionality of pure LCFs, researchers have integrated various metal nanoparticles into the fabrication of lignocellulosic composite films.^635^ These nanoparticles include pure metals like silver (Ag), gold (Au), copper (Cu), zinc (Zn), platinum (Pt), nickel (Ni), and palladium (Pd), as well as metal compound nanoparticles such as aluminum nitride (AlN), lithium chloride (LiCl), and neodymium iron boron magnets (Nd_2_Fe_14_B).^635,636^ This approach broadens the potential applications of lignocellulosic-based materials, offering solutions for advanced functionalization and improved performance in various technological and environmental contexts.

Metallic NPs impart diverse functions depending on their composition. Key performance metrics, summarized in Table 6, highlight the trade-offs between nanoparticle type, their functions, typical loading, dispersion methods, and toxicity concerns, offering a framework for designing functional LCFs. Antimicrobial agents are commonly Ag or Cu nanoparticles embedded in cellulose. Ag NPs release Ag^+^ ions that damage bacterial cell walls at low concentrations,^637^ but their cytotoxicity demands minimal loading and strong binding in the film. Cu NPs similarly exert antimicrobial activity via Cu^2+^ release and Fenton-like reactive oxygen species generation, often effective at loadings of 0.1–5 wt % in LCFs,^638^ though fewer studies focus on Cu in lignocellulose films compared to Ag. For electrical and thermal conductivity, noble metals such as Ag and Au NPs can form percolating networks within LCFs, enabling conductive paths at relatively low volume fractions. Percolation thresholds typically depend on NP shape, aspect ratio, and dispersion quality: Ag nanowires can achieve conductivity at loadings of a few weight percentages by forming interconnected networks, whereas spherical NPs often require higher loadings or postdeposition treatments to establish conductive necks.^639^ Thermal conductivity enhancement likewise benefits from metal NP networks, though the inherently low thermal conductivity of cellulose limits overall gains; hybrid strategies may combine metal NPs with thermally conductive fillers. UV-blocking also leverages Ag and Au NPs: their localized surface plasmon resonance can be tuned by NP size and surrounding matrix, allowing LCF-based sensors or protective films. Effective NP concentrations are typically <1 wt % for potent biocides (Ag, Cu) and higher values for conductive/optical effects. Below are some specific research examples detailing the utilization of metal NPs to achieve the functionalization of LCFs.

**Table 6:** Key Metrics for Metal and Metal-Oxide NPs in Lignocellulosic Films

nanoparticles	primary function	typical loading	dispersion method	cytotoxicity	ref
Ag	UV blocking, antimicrobial, conductive	0.1–2 wt %	in situ reduction or coating	low	^640^
Au	photothermal conversion, conductive	0.04–1 mM	CNC embedding	low	^641^
CuO	antimicrobial, conductive	0.1–5 wt %	in situ reduction	moderate	^638^
ZnO	UV shielding, antimicrobial	∼20 mM	coprecipitation on cellulose	moderate (Zn^2+^ release at high doses)	^642^
TiO_2_	UV shielding, photocatalysis	1–10 wt %	sol–gel or mixing	low	^643^

Ag nanoparticles are extensively studied due to their broad-spectrum antibacterial activity against fungi, bacteria, algae, and certain viruses, offering advantages over conventional antimicrobial agents with enhanced durability.^649,650^ Utilizing lignocellulose as a natural biomass can mitigate the cytotoxicity of Ag and enhance biocompatibility. In antibacterial applications, Lu et al. demonstrated the reduction of Ag^+^ by lignin to elemental Ag in situ. They further self-assembled lignin molecules into nanotubes using an aqueous AgNO_3_ electrolyte solution to fabricate composite films (Figure 24ai).^644^ These films exhibited impressive mechanical properties with tensile strength and elongation at break of 68.7 MPa and 11.3%, respectively. The antibacterial efficacy against *Escherichia coli* and *Staphylococcus aureus* reached 99.9% and 97.2%, respectively, alongside notable hydrophobic and UV-resistant properties. In water treatment, He et al. immobilized Ag nanoparticles in situ on sulfuric acid-acidified CNC during interfacial polymerization (Figure 24aii).^645^ The resulting nanofiltration membrane demonstrated high retention rates of 97.11% for Na_2_SO_4_ and 32.55% for NaCl, highlighting its effective filtration capabilities.

**Figure 24:**
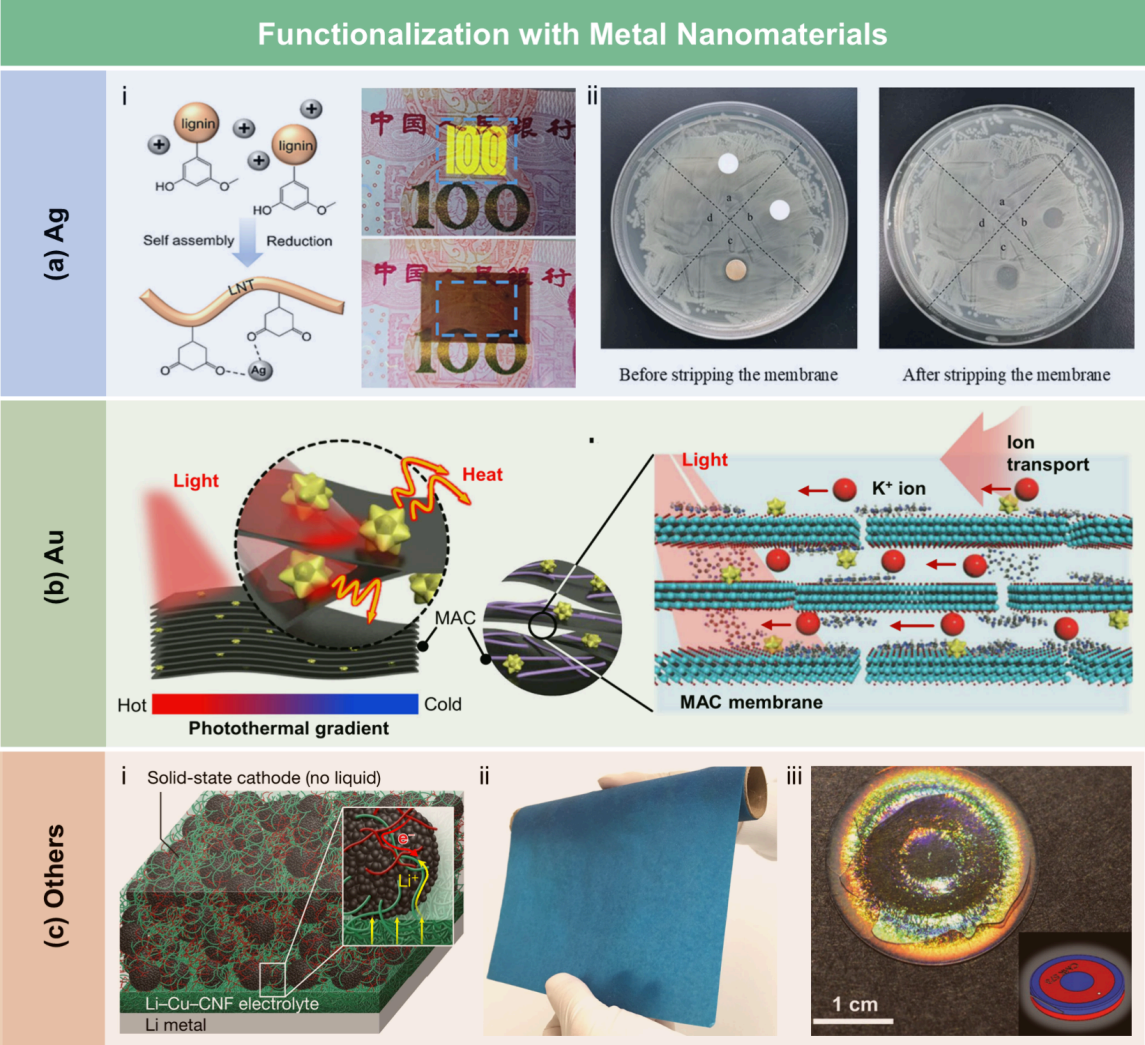
Functionalization of LCFs with metal nanomaterials. (a) Schematic diagram of lignin nanolization/AgNPs generation and the UV blocking at 365 nm wavelength (i). Adapted with permission from ref 644. Copyright 2023 Elsevier. Bacteriostatic zone experiment of Ag nanoparticles/CNF film (ii). Adapted with permission from ref 645. Copyright 2022 Springer Nature under CC BY 4.0 (https://creativecommons.org/licenses/by/4.0/). (b) MXene/AuNS/CNF membrane under exposure to localized NIR light and directional cation transport through the MXene/AuNS/CNF membrane under photothermal gradient. Adapted with permission from ref 646. Copyright 2023 Springer Nature under CC BY 4.0 (https://creativecommons.org/licenses/by/4.0/). (c) Diagram of a solid-state full battery consisting of a Li-metal anode (i); Digital photo of a 1-m-long roll of Li-Cu-CNF membrane (ii). Adapted with permission from ref 647. Copyright 2021 Springer Nature. Exotically patterned films cast on oppositely magnetized polydomain magnets (iii). Adapted with permission from ref 648. Copyright Wiley-VCH under CC BY 4.0 (https://creativecommons.org/licenses/by/4.0/).

Au nanoparticles (AuNPs) are renowned for their exceptional biocompatibility, chemical stability, conductivity, and distinctive optical properties, making them highly versatile in biosensing, catalysis, and drug delivery applications.^651^ Yeom et al. developed a photosensitive ion channel using plasmonic AuNPs embedded in CNF within layered MXene nanosheets (Figure 24b).^646^ The optimized composite film demonstrated a photothermal ion current of approximately 40 nA, which significantly exceeded the original MXene nanochannel’s performance by nearly 7-fold. In catalysis, Cabreira et al. spin-coated MCC dissolved in 1-butyl-3-methylimidazole chloride solution, followed by immersion in an aqueous dispersion of AuNPs.^652^ The resulting cellulose/AuNPs composite film, featuring nanoparticles approximately 10 nm in diameter, exhibited robust catalytic activity in the reduction of 4-nitrophenol to 4-aminophenol using NaBH_4_, maintaining effectiveness over five cycles.

Other metals, such as Cu and Ni, have also been incorporated into lignocellulose to create composite films for applications in solid-state batteries, wood antibacterial treatments, UV/electromagnetic shielding, and catalytic reduction (Figure 24c).^647,653–655^ Recent advancements have explored composite metal nanoparticles in LCFs. For instance, Zong et al. developed a laminated double-layer structure comprising Metglas and cellulose films, exhibiting a significant magnetoelectric coefficient of approximately 1.5 V·cm^–1^·Oe^–1^, including ubiquitous Fano resonance phenomena in physics.^656^ In another work, Frka-Petesic et al. utilized CNC and Nd_2_Fe_14_B magnets to create composite films with adjustable iridescent visual properties (Figure 24ciii).^648^

The composite integration of metal nanoparticles with LCFs enables versatile functional enhancements, encompassing improved electrical and thermal conductivity, enhanced optical properties, antibacterial and antioxidant capabilities, and heightened catalytic efficiency. These advancements find applications across diverse sectors including electronics, food packaging, biomedicine, and catalytic conversions. However, challenges such as high production costs and potential environmental impacts necessitate further research and development to facilitate widespread industrial adoption of this approach.

#### 4.3.3 Metal-Oxide Nanomaterials

Metal oxide nanoparticles share similarities with metal nanoparticles, including high specific surface area and excellent catalytic, magnetic, optical, and electrical properties.^657^ Reorganized by function, LCFs functionalized with metal-oxide NPs such as ZnO, TiO_2_, CeO_2_, and CuO are widely incorporated into LCFs to impart UV-blocking, electrical conductivity, photocatalytic self-cleaning, and antimicrobial functionalities. Incorporating metal oxide nanoparticles into LCFs can substantially improve their mechanical strength, UV shielding efficacy, antibacterial properties, self-cleaning capabilities, and ability to degrade organic pollutants.^635^ Table 6 summarizes the typical NP loadings, dispersion techniques, and associated toxicity concerns for various functional applications. Photoactive metal oxides such as ZnO and TiO_2_ are commonly employed in antimicrobial layers, where their ability to generate reactive oxygen species under light irradiation disrupts bacterial membranes and suppresses microbial proliferation. For UV protection, TiO_2_ and ZnO NPs are typically used at loadings of 5–10 wt %,^658^ often in combination with biobased UV-absorbing components such as lignin to extend coverage across the UV-A, UV-B, and UV-C ranges while maintaining high visible light transmittance. Importantly, the performance of LCFs incorporating metal oxides can rival or even surpass that of conventional synthetic composites, but successful design requires careful optimization to balance functionality with environmental and biological safety. As a case in point, ZnO-nanocellulose composites have demonstrated the ability to downregulate bacterial virulence genes while maintaining low cytotoxicity toward human cells,^659^ illustrating the nuanced trade-offs between efficacy and biocompatibility that must be navigated when engineering multifunctional, sustainable biobased materials. Below are some specific research examples detailing the utilization of metal oxide NPs to achieve the functionalization of LCFs.

Among various metal oxide nanoparticles, TiO_2_, zinc oxide (ZnO), copper oxide (CuO), and iron oxide (Fe*_n_* O*_m_*) have been extensively investigated for their applications in cellulose-metal oxide composites due to their abundance and superior performance.^636^ TiO_2_ nanoparticles are nontoxic materials known for their excellent photocatalytic activity. They generate electrons and holes when exposed to ultraviolet light, enabling the oxidation and decomposition of organic pollutants while also possessing antibacterial and antioxidant properties.^666^ Incorporating TiO_2_ nanoparticles into LCFs enhances their capabilities for photocatalytic pollutant degradation, antibacterial action, antioxidant protection, self-cleaning, and electrochemical functions (Figure 25ai).^539,660^ For instance, Yu et al. synthesized CNF/TiO_2_ nanoparticle composites and investigated their potential application in PVA-based membranes (Figure 25aii).^661^ The results demonstrate that these nanocomposites significantly enhance the mechanical and light-blocking properties of the PVA film, displaying no evident toxicity toward intestinal bacteria and human colon cells and exhibiting favorable biocompatibility. Furthermore, TiO_2_/lignocellulosic composite films have found applications in flexible electrodes, drug delivery systems, food packaging, and other fields.^667,668^

**Figure 25:**
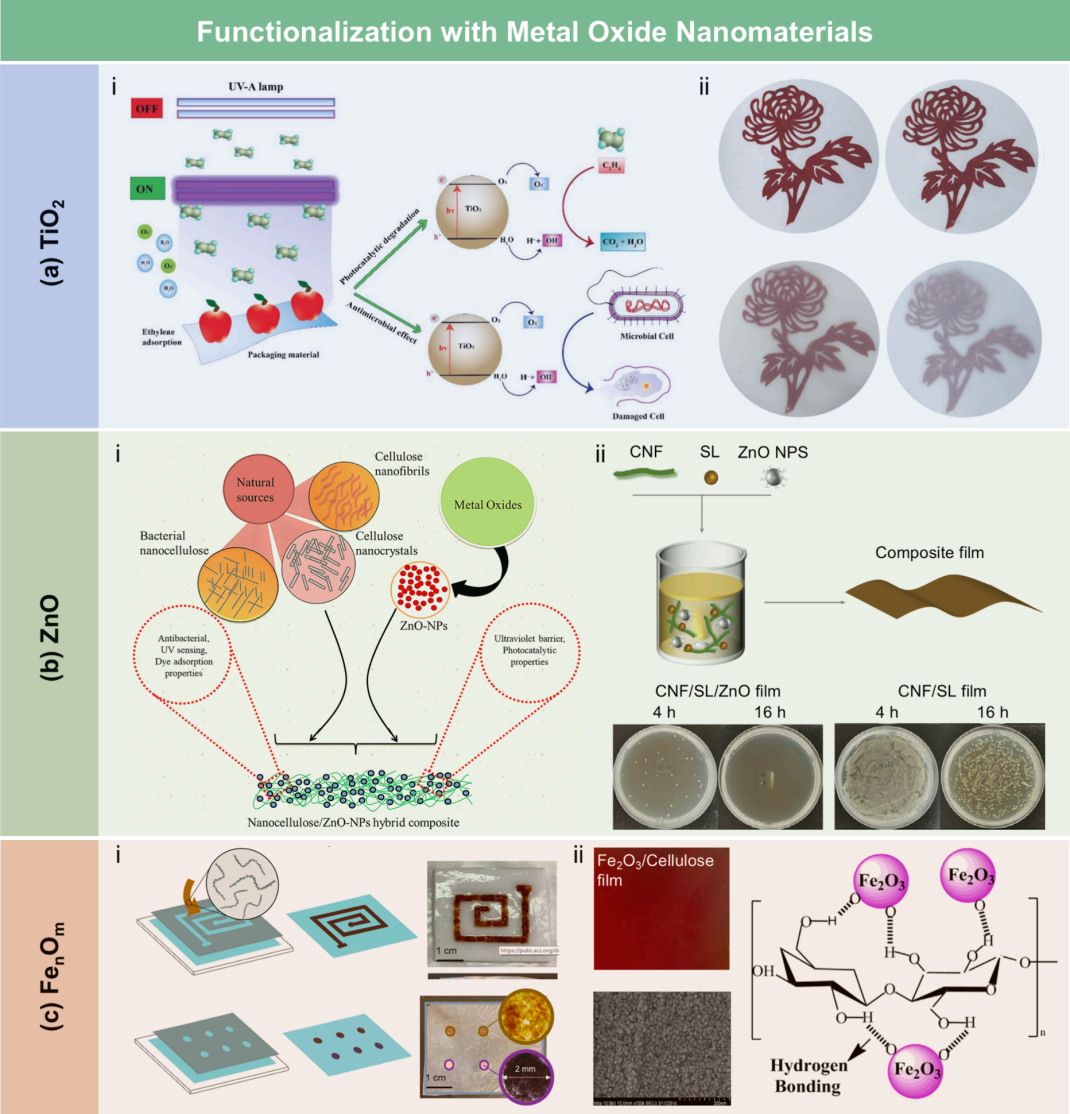
Functionalization of LCFs with metal-oxide nanomaterials. (a) Photocatalytic activity, ethylene scavenging, and antimicrobial properties of films containing TiO_2_ nanoparticles (i). Adapted with permission from ref 660. Copyright 2021 Elsevier. Images of PVA-based films containing different CNF/TiO_2_ nanoparticles above a background of a red flower (ii). Adapted with permission from ref 661. Copyright 2019 Elsevier. (b) Nanocellulose/ZnO-nanoparticles nanocomposite material production and properties in a schematic illusion (i). Adapted with permission from ref 662 Copyright 2020 Elsevier. Schematic diagram of the preparation and antibacterial properties of the CNF/SL/ZnO film (ii). Adapted with permission from ref 663. Copyright 2024 Elsevier under CC BY 4.0 (https://creativecommons.org/licenses/by/4.0/). (c) Cellulose fibers-superparamagnetic iron oxide nanoparticles applied to one side of the film and film functionalized with superparamagnetic iron oxide nanoparticles or AuNPs on the surface (i). Adapted with permission from ref 664. Copyright 2021 American Chemical Society. Chemical structure of Fe_2_O_3_/cellulose nanocomposite films (ii). Adapted with permission from ref 665. Copyright 2014 Elsevier.

ZnO nanoparticles are valued for their nontoxicity, environmental friendliness, affordability, and widespread availability.^635,669^ They exhibit excellent antibacterial, antioxidant, and UV shielding properties (Figure 25bi).^662^ When integrated with lignocellulose, composite films offer significant potential in applications such as food packaging and UV protection. For instance, Zhu and the team utilized sodium lignosulfonate (SL) and ZnO nanoparticles as functional fillers and structural components in CNF films (Figure 25bii).^663^ This integration resulted in the development of CNF/SL-ZnO films with antiultraviolet, antioxidant, and antibacterial characteristics. These films exhibited extended shelf life in food packaging, showcased excellent biodegradability, and had minimal impact on the soil microenvironment. In UV shielding applications, Roy et al. created a novel biobased nanocomposite film using gelatin and CNF as polymer matrices with ZnO nanoparticles as fillers.^670^ This composite film exhibited excellent UV blocking (>95%) while maintaining high transparency (>75%). CuO nanoparticles also possess diverse properties that hold promise in photoconductors, biosensors, biomedicine, and magnetic storage media applications. However, their tendency to self-aggregate limits their efficiency and practical utility. Lignocellulose acts as a carrier and dispersion stabilizer when combined with CuO nanoparticles, effectively addressing these challenges. For example, Zhou et al. demonstrated that the hydroxyl groups of CNC can interact electrostatically with Cu^2+^, preventing CuO nanoparticle agglomeration and thereby improving dispersibility and catalytic activity.^635^ Valencia et al. synthesized CuO nanoparticles in situ on TEMPO-oxidized CNF membranes to create hybrid membranes with superior dye removal efficiency and antibacterial properties in water applications.^671^

Over recent decades, the synthesis and application of Fe*_n_* O_*m*_ nanoparticles have garnered significant attention due to their nanoscale size, high specific surface area, and superparamagnetism.^635^ In the realm of Fe_3_O_4_ nanoparticles and cellulose film composites, innovative approaches have been developed to exploit these properties. For instance, Mira-Cuenca et al. employed a screen-printing process to deposit superparamagnetic Fe_3_O_4_ nanoparticles in specific regions of cellulose films, creating patterned surfaces capable of noninvasive tissue deformation and adhesion monitoring (Figure 25ci).^664^ In another study, Yadav et al. impregnated Fe_2_O_3_ nanoparticles into RC membranes, producing high-performance nanocomposite membranes (Figure 25cii).^665^ These membranes demonstrated significant improvements in tensile strength and elasticity compared to pure RC, with modulus increases of approximately 39% and 57%, respectively.

In summary, incorporating metal oxide nanoparticles significantly enhances the mechanical strength, electrical conductivity, and thermal conductivity of lignocellulose films. These nanoparticles also confer specific optical functions to the films, such as UV protection and photocatalysis. However, the high preparation costs and potential environmental impacts of producing and using certain metal oxide nanoparticles limit their large-scale application. Future research should focus on optimizing the performance of metal oxide nanoparticle-lignocellulose composite films while developing methods to reduce preparation costs and minimize environmental impacts.

#### 4.3.4 2D Nanomaterials

2D nanomaterials are characterized by their nanoscale thickness and lateral dimensions that can extend to micrometerss or more. Typical examples include graphene and its derivatives, disulfides (e.g., molybdenum disulfide (MoS_2_), tungsten disulfide (WS_2_)), boron nitride (BN), MXenes, black phosphorus, and carbon nitride (C_3_N_4_).^672^ The primary structural feature of these materials is the highly ordered arrangement of atomic layers, forming an extremely thin 2D layered structure. This configuration imparts unique anisotropic properties, exceptional mechanical properties, and a high specific surface area. Depending on their composition, these materials also exhibit varying degrees of thermal conductivity, electrical conductivity or insulation, and optical properties.^673^ Such characteristics make them highly suitable for applications in flexible electronic devices, sensors, energy storage, and other areas. Incorporating 2D nanomaterials with lignocellulose can significantly enhance the mechanical properties of lignocellulose films. However, realizing this potential depends critically on addressing fundamental engineering challenges. The successful integration of these materials hinges on sophisticated design principles, primarily focused on achieving uniform dispersion, ensuring strong interface adhesion with the lignocellulose matrix, and implementing precise orientation control of the nanosheets. These key factors are summarized in Table 7 (factor vs outcome). For example, oxidized graphene or MXene with surface −OH/–COOH groups forms hydrogen bonds with cellulose, enhancing stiffness and barrier properties.^674^ Effective dispersion and interface adhesion are foundational for creating high-performance composites, preventing aggregation of the nanomaterials and ensuring efficient load transfer. Researchers have developed various strategies to overcome the inherent incompatibility between hydrophilic cellulose and often hydrophobic or chemically distinct 2D materials. For instance, chemical functionalization is a common approach, such as the use of carboxymethylated cellulose nanofibers to prepare stable multilayer Ti_3_C_2_T_*X*_ EMI shielding films.^675^ Beyond simple dispersion, controlling the orientation of anisotropic 2D nanosheets is a powerful strategy for tuning material properties. By aligning the planar materials within the matrix, typically parallel to the film surface, properties like thermal and electrical conductivity can be dramatically enhanced in a specific direction. Also, the orientation of sheets can drastically affect anisotropy: aligned, layered structures yield high in-plane mechanical modulus and conductivity, whereas random or misaligned sheets reduce these benefits. In CNF-GO composites, even 0.1 wt % GO markedly increased cellulose fibril alignment and in-plane Young’s modulus.^676^ Dispersion quality controls percolation: uniform exfoliation of nanosheets prevents aggregation (which creates stress concentrators and breaks conductive paths). Achieving monolayer or few-layer dispersion (via exfoliation, surface modifiers, or LbL assembly) maximizes surface area contact. These strategies are essential for effectively translating the nanoscale properties of the fillers into macroscale performance enhancements in the final composite film. Below are some specific research examples detailing the utilization of 2D nanomaterials to achieve functionalization of LCFs.

**Table 7:** Design Principles for 2D Nanomaterial–LCFs

design factor	well engineered case	outcome	poor case	outcome	ref
interface adhesion	–OH/–COOH rich surface, covalent/H bonds	high stress transfer, toughness; excellent barrier	hydrophobic mismatch, weak bonding	slippage, delamination, low strength	^674,677^
sheet orientation	aligned 2D layers/CNF parallel to plane	enhanced in-plane modulus/conductivity; anisotropic barrier	random orientation, mixed planes	isotropic but lower mechanical/thermal	^678^
nanosheet dispersion	individual, fully exfoliated sheets	continuous network; optimized water vapor barrier and UV-shielding properties	aggregated stacks/clusters	voids, weak spots, poor properties	^679^
filler aspect ratio	large-area, high aspect-ratio sheets	enhanced mechanical properties, lightweight	small, fragmented sheets	requires high loading, minimal effect	^680^

MoS_2_ and WS_2_ are exemplary 2D layered transition metal dichalcogenides. Their single-layer structure comprises a layer of molybdenum or tungsten atoms sandwiched between two layers of sulfur atoms, creating a graphite-like configuration. The honeycomb lattice structure of these materials confers excellent electronic, mechanical, and optical properties. For instance, Camilo et al.^681^ utilized a liquid exfoliation method to prepare MoS_2_/CNF nanocomposites (Figure 26ai). The resulting film exhibited anisotropic properties, narrowed absorption bands at energy values of 1.89 and 2.0 eV, and enhanced fluorescence gain. Additionally, Liu et al.^682^ developed a biomimetic strategy using sulfonated CNCs as exfoliation agents and stabilizers to efficiently delaminate WS_2_ nanosheets in water. The resulting CNC@WS_2_ hybrids exhibited exceptional stability across wide pH (1–13) and temperature (1–95 °C) ranges. Incorporation of these hybrids into cationic polyurethane (Cat-PU) produced flexible photonic films retaining the chiral nematic structure of CNCs (Figure 26aii), featuring vivid structural colors, improved mechanical strength, and multistimuli responsiveness (strain, humidity, and photothermal effects).

**Figure 26:**
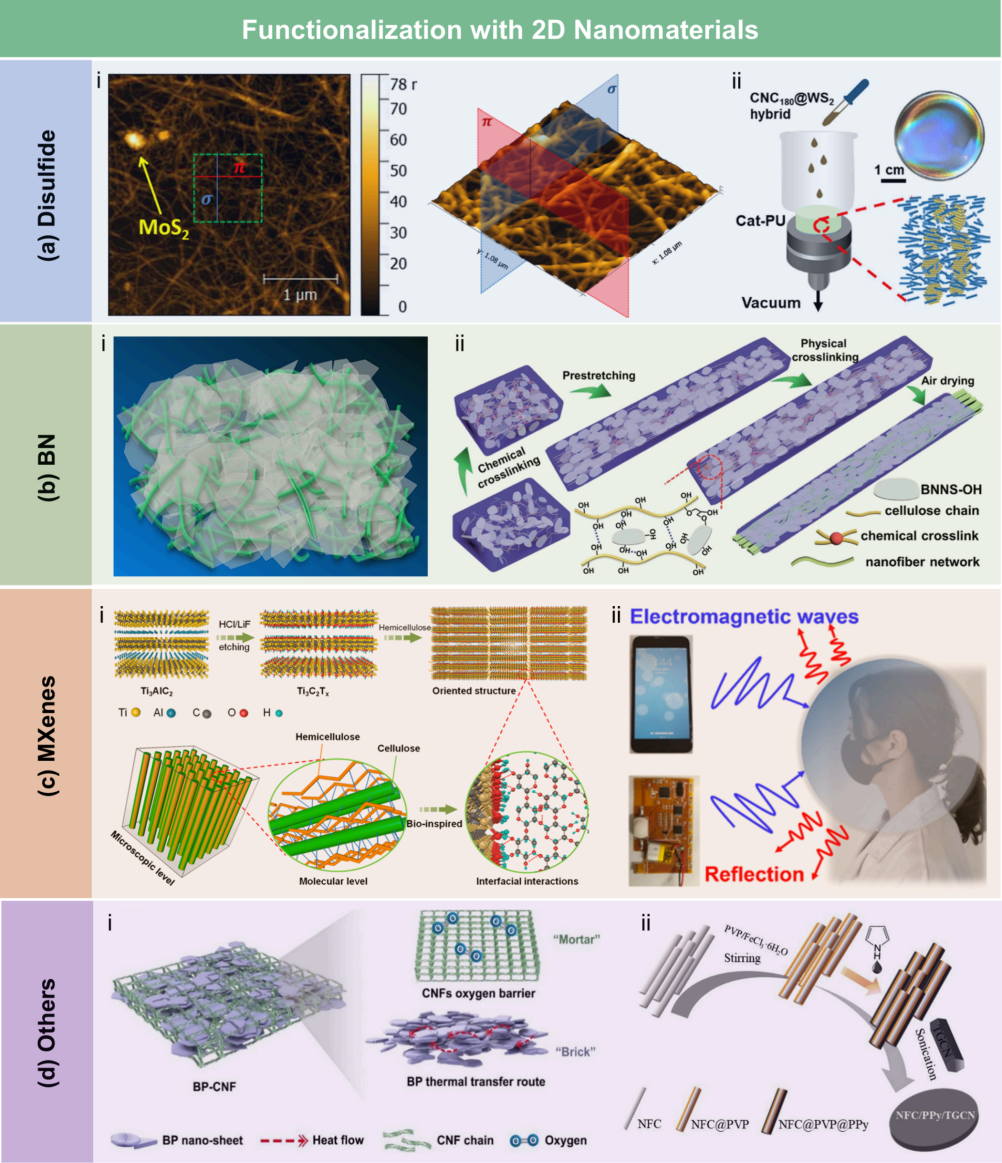
Functionalization of LCFs with 2D nanomaterials. (a) Topographic AFM image of the MoS_2_/CNF sample and CNF’s 3D zoomed showing the intersections of the planes (i). Adapted with permission from ref 681. Copyright 2019 Springer Nature. Schematic illustration of a photonic film with chiral nematic phase structure after vacuum filtration of cationic waterborne polyurethane (Cat-PU) and CNC@WS_2_ dispersion (ii). Adapted with permission from ref 682. Copyright 2022 Elsevier. (b) Schematic to show the structure of cellulose nanofiber with layered boron nitride nanosheets (i). Adapted with permission from ref 683. Copyright 2014 American Chemical Society. Schematic illustration of the anisotropic cellulose/BN–OH film construction (ii). Adapted with permission from ref 684. Copyright 2020 Royal Society of Chemistry. (c) Schematic illustration of wood-inspired MXene-hemicellulose films with oriented structures at the microscopic level and interfacial interactions (i). Adapted with permission from ref 685. Copyright 2022 American Chemical Society. Model prototype for protecting humans from electromagnetic radiation (ii). Adapted with permission from ref 686. Copyright 2021 Springer Nature. (d) Schematic illustration of the black phosphorus-CNF film (i). Adapted with permission from ref 687. Copyright 2021 IOP Publishing Ltd. The fabrication procedure of freestanding core–shell CNF/PPy/graphite-C_3_N_4_ composite film (ii). Adapted with permission from ref 688. Copyright 2019 Elsevier.

2D BN nanomaterials, such as hexagonal BN (h-BN), possess a structure analogous to graphene. This structure is formed by honeycomb planar layers composed of alternating nitrogen and boron atoms, stacked on each other via weak van der Waals forces. The strong covalent B–N bonds endow BN with high thermal stability and mechanical strength (Figure 26bi).^683^ Additionally, h-BN is a wide bandgap insulator, with a bandgap of approximately 5.9 eV, making it suitable for electrical insulation and thermal management applications. For example, Zhang et al. prepared high-quality holographic cellulose/boron nitride nanofilms using a holographic CNF-assisted ultrasonic treatment.^689^ These films demonstrated excellent flexibility and mechanical properties, achieving a high in-plane thermal conductivity of 11.78 W·m^–1^ ·K^–1^. Similarly, Tu et al. employed chemical-physical dual cross-linking and stretching orientation strategies to construct highly ordered CNF networks and edge-hydroxylated boron nitride nanofilms (Figure 26bii).^684^ These nanofilms exhibited a high tensile strength of 226 MPa and an in-plane thermal conductivity of 20.41 W·m^–1^·K^–1^, which is nearly 30 times higher than their through-plane thermal conductivity, thus demonstrating significant anisotropy.

In 2011, Naguib et al.^690^ first identified the carbides, nitrides, and carbonitrides of 2D transition metals such as titanium, scandium, and vanadium, collectively known as MXenes. These emerging 2D materials are characterized by a layered structure of alternating transition metals and carbon or nitrogen atoms. MXenes exhibit excellent hydrophilicity, conductivity, and mechanical properties, making them widely applicable in electronics, electromagnetic shielding, catalysis, and biology.^691^ Chen et al. utilized short-chain hemicellulose (xylo-oligosaccharides) as molecular adhesives to bind MXene nanosheets, resulting in MXene-hemicellulose films with high conductivity (64,300 S·m^–1^) and mechanical strength (125 MPa) without introducing a substantial insulating phase (Figure 26ci).^685^ Li et al. proposed a straightforward method for preparing multilayer Ti_3_C_2_T_*X*_ EMI shielding composite films using aminosulfonic acid-modified cellulose nanofibers.^692^ These films demonstrated high EMI shielding effectiveness (45.02 dB) at 12.4 GHz and an EMI shielding efficiency of 99.996%. Xu et al. developed a composite film using bacterial cellulose and MXene, which exhibited significant shielding performance (19,652 dB·cm^2^·g^–1^) (Figure 26cii).^686^ In another study, Chang et al. synthesized a composite film composed of Ti_3_C_2_T_*X*_, CNF, and sodium lignin sulfonate via a hydrothermal method.^693^ The resulting film exhibited a tensile strength of 133 MPa and a conductivity of up to 1.75 × 10^5^ S·m^–1^. The flexible solid-state supercapacitor electrode assembled from this composite film demonstrated an excellent volumetric specific capacitance (748.96 F·cm^–3^).

Other 2D nanomaterials, such as black phosphorus, C_3_N_4_ nanoparticles, and 2D bentonite nanosheets, can also be integrated with lignocellulosic to produce thin films with enhanced functionalities, including thermal conductivity, antibacterial properties, UV shielding, fire resistance, and capacitor performance.^694,695^ For instance, Wei et al. developed black phosphorus-CNF thin films through vacuum filtration of black phosphorus-CNF solutions, achieving a high in-plane thermal conductivity of 22.3 W·m^–1^·K^–1^ at room temperature (Figure 26di).^687^ Li et al. fabricated core–shell CNF/PPy/graphite-C_3_N_4_ composite films using simple polymerization and vacuum-induced self-assembly techniques (Figure 26dii).^688^ The resulting electrode exhibited a surface capacitance of 2.53 F·cm^–2^ at a current density of 5 mA·cm^–2^, corresponding to a specific capacitance of 158 F·g^–1^.

Although nanomaterials have demonstrated tremendous potential in enhancing the functionality of lignocellulose composite films, challenges such as their dispersibility, compatibility within lignocellulosic matrices, and effective interface bonding still require thorough investigation and resolution. Moreover, the high preparation costs of nanomaterials and potential environmental impacts associated with some materials necessitate comprehensive assessments encompassing both material preparation and environmental consequences before industrial-scale application. Achieving functionalization of lignocellulose films through nanomaterials combined with chemical modification is one of the potential solutions to the aforementioned problems.^696^ Furthermore, when considering applications in biomedicine or medical sensors, it is crucial to evaluate their biological toxicity and potential implications for human health meticulously. These considerations are essential steps toward ensuring the safe and effective deployment of nanomaterial-enhanced lignocellulose composite films across various domains.

### 4.4 Functionalization with Conducting Polymers

Although lignocellulose is inherently nonconductive, intrinsically conducting polymers (ICP) such as poly­(3,4 ethylenedioxythiophene) (PEDOT), polyaniline (PANI), and PPy have been incorporated into lignocellulosic substrates to prepare conductive composites for energy and electronic applications.^697^ However, several key challenges remain, including weak interfacial adhesion, uneven dispersion, limited mechanical durability, chemical instability, and difficulty in establishing continuous conductive pathways.^698,699^ To overcome these limitations, strategies such as coating, blending, and matrix mixing with lignocellulose have been explored.^700^ For instance, Unuma et al.^701^ investigated the terahertz (THz) transmission properties of nanocellulose (NFC)-PEDOT:PSS composite films by analyzing their conductivity at PEDOT:PSS contents ranging from 0% to 50%. Figure 27ai shows that the real part of the THz conductivity of the composite approached a finite positive value, correlating with the PEDOT:PSS content. This behavior indicates the formation of continuous conductive pathways within the NFC matrix. Increased PEDOT:PSS further enhanced charge transport by reducing carrier localization. This research suggests that NFC-based composites hold potential for tunable optoelectronic applications in the THz frequency range. Lapka et al.^702^ developed flexible conductive NFC composite films by incorporating multiwalled MWCNT, PPy nanotubes (PPy-NT), and carbonized PPy nanotubes (C-PPy-NT). Figure 27aii shows images of the fabricated NFC composite films, demonstrating their flexibility and mechanical stability. Among the tested samples, the PPy-NT/NFC composites demonstrated the highest electrical conductivity of 1.16 S·cm^–1^ and a specific capacitance of 209.7 F·g^–1^ at a scan rate of 10 mV·s^1^. Moreover, these composites achieved 75% electromagnetic interference (EMI) shielding effectiveness within the C-band frequency range (5.85–8.2 GHz). Dong et al.^703^ fabricated high-performance lignin-containing cellulose nanofiber (LCNF) composite films by introducing a lignin/PPy (LS/PPy) interpenetrating network, as illustrated in Figure 27aiii. The resulting composite films demonstrated a tensile strength of 20.46 MPa and an areal capacitance of 2,567 mF·cm^–2^ at 1 mA·cm^–2^ current density. The study showed that lignin contributed pseudocapacitance via reversible quinone/hydroquinone conversion while influencing LCNF fibrillation, thereby enhancing both mechanical properties and capacitive performance. In addition, the optimized all-solid-state supercapacitor (ASSC) exhibited an excellent energy density of 88.6 μWh·cm^–2^ and a high Coulombic efficiency (CE) (∼98%), highlighting the application potential of LCNF composites in sustainable energy storage.

**Figure 27:**
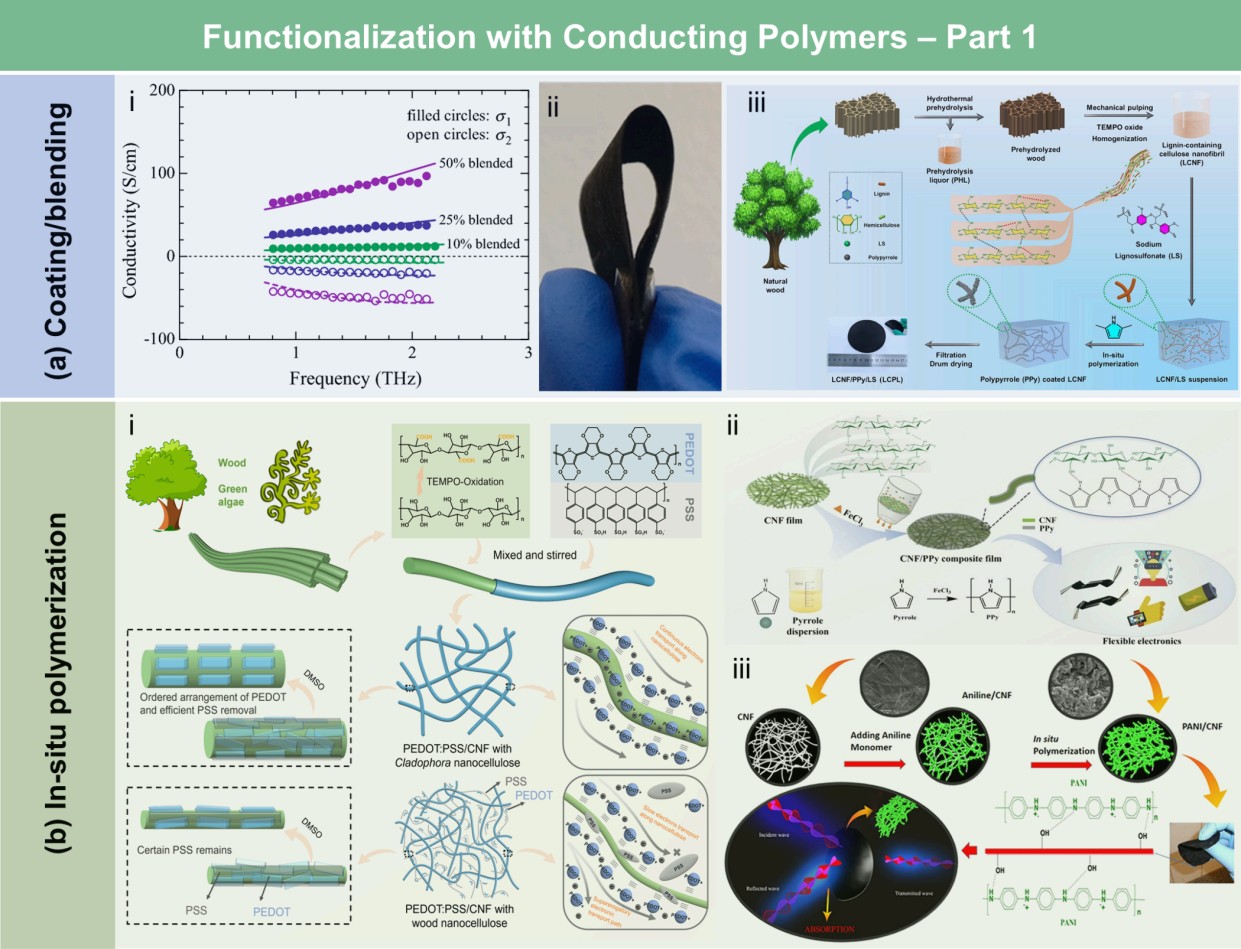
(a) THz complex conductivity spectra of NFC composite films containing varying PEDOT:PSS (i). Adapted with permission from ref 701. Copyright 2019 Springer. Photograph of the polypyrrole nanotube/NFC composite film (ii). Adapted with permission from ref 702. Copyright 2021 Elsevier. Schematic illustration of the preparation of LCNF/PPy/LS (LCPL) nanocomposite film (iii). Adapted with permission from ref 703. Copyright 2023 Elsevier. (b) Comparison schematic illustrating nanofibrous networks, compositional differences, and electron transport mechanisms in conductive PEDOT:PSS/CNF nanopapers fabricated from two types of nanocellulose (i). Adapted with permission from ref 705. Copyright 2024 Elsevier. Schematic illustration of in situ polymerization of pyrrole on cellulose nanofiber film (ii). Adapted with permission from ref 706. Copyright 2024 Elsevier. Schematic diagram of the preparation of flexible cellulose nanofiber-based polyaniline paper and its application as a microwave absorber (iii). Adapted with permission from ref 707. Copyright 2018 American Chemical Society.

Blending is a common strategy for preparing ICP/LCFs, offering advantages such as simple processing, scalability, and partial retention of the mechanical properties of lignocellulosic materials while leveraging the functionalities of conductive polymers. However, this approach is limited by poor dispersion of conductive polymers and weak interfacial bonding, often resulting in discontinuous pathways that compromise conductivity and stability. In-situ polymerization has therefore been adopted as an effective strategy to address these challenges. For example, Du et al.^704^ prepared highly conductive PEDOT:PSS/CNF nanopaper (PEDOT:PSS/CNP) via vacuum filtration combined with a DMSO treatment method for flexible supercapacitors. DMSO treatment significantly boosted the conductivity of PEDOT:PSS/CNP films 16-fold by removing excess PSS, thereby exposing PEDOT-rich domains and enabling continuous conductive pathways for improved charge transport. The optimized PEDOT:PSS/CNP also demonstrated tensile strength of 72 MPa. The assembled symmetric supercapacitor demonstrated an ultrahigh areal capacitance of 854.4 mF·cm^–2^ and a volumetric capacitance of 122.1 F·cm^–3^, operating stably at a high energy density of 30.86 μWh·cm^–2^ (4.41 mWh·cm^–3^). Additionally, after 10,000 cycles of charge-discharge, the device retained 95.8% of its capacitance, exhibiting outstanding stability. Chen et al.^705^ employed an external surface area-induced phase separation strategy to fabricate highly ordered PEDOT:PSS CNP, significantly enhancing its conductivity. Figure 27bi presents structural comparisons of different nanocellulose-based conductive nanopapers, revealing variations in nanofiber networks, phase separation characteristics, and electron transport pathways. The optimized nanopaper achieved an impressive electrical conductivity of 252 S·cm^–1^, surpassing conventional PEDOT:PSS/cellulose composites. With a thickness of only 6 μm, the material achieved a significant specific shielding of 33,122 dB·cm^2^·g^–1^, highlighting its excellent suitability for both supercapacitor and EMI shielding applications. Using pyrrole monomer and FeCl_3_ oxidant, Zheng et al.^706^ synthesized conductive composite films of CNF/PPy through in situ polymerization. The film with a FeCl_3_-to-pyrrole molar ratio of 2:1 demonstrated the highest electrical conductivity (0.99 S·m^–1^) and specific capacitance (138.26 F/g). Figure 27bii illustrates the fabrication process of the CNF/PPy composite film and highlights its application potential in wearable energy storage devices and flexible electronics. Additionally, Du et al.^529^ fabricated multifunctional CNP by CS impregnation followed by in situ PPy polymerization, where CS functionalization promoted hydrogen bonding with pyrrole species, yielding a more uniform and smoother PPy coating. The resulting CNP/CS/PPy composite film exhibited excellent resistance against water, bacteria and high conductivity. The composite’s tensile strength at the wet state reached 80 MPa (10 times higher than pure CNP), with a high electrical conductivity of 6.5 S·cm^–1^, making it suitable for EMI shielding (18 dB shielding effectiveness). Moreover, the material demonstrated antibacterial activity, achieving a bactericidal rate of 99.28% against *Staphylococcus aureus* and 95.59% against *Escherichia coli*. Similarly, Gopakumar et al.^707^ employed in situ polymerization to deposit PANI on CNF, producing a lightweight, flexible, and conductive nanopaper for X-band EMI shielding in the range of 8.2–12.4 GHz, as shown in Figure 27biii. The resulting nanopaper exhibited a conductivity of 0.314 S·cm^–1^ and a total shielding effectiveness of −23 dB at 8.2 GHz, effectively attenuating over 99% of incident electromagnetic waves. Furthermore, the material primarily absorbed (87%) rather than reflected (13%) EM waves, reducing secondary electromagnetic pollution.

Despite the excellent conductivity, flexibility, and processability of individual ICPs, they still have certain limitations. For instance, PEDOT:PSS exhibits superior conductivity and environmental stability but suffers from poor mechanical properties, with PSS potentially disrupting its conductive network. Meanwhile, PANI and PPy possess high electrochemical activity but are relatively brittle and prone to degradation under high temperatures or prolonged use. Therefore, synergizing multiple conductive polymers has emerged as an effective strategy to optimize their properties. For example, Lay et al.^708^ developed smart conductive nanopaper based on CNF, PEDOT:PSS, and PPy, as shown in Figure 28ai. The optimized CNF-PEDOT:PSS–PPy nanopaper exhibited high specific capacitance (315.5 F·g^–1^) and electrical conductivity (10.55 S·cm^–1^), demonstrating the synergistic enhancement effect of PEDOT:PSS and PPy, significantly improving its electrochemical performance. Similarly, Li et al.^709^ fabricated flexible PEDOT:PSS/PPy/paper-based thermoelectric (TE) composite films using a two-step approach, as shown in Figure 28aii. First, PPy/paper composite films were synthesized via in situ polymerization, followed by doping with PEDOT:PSS in DMSO to enhance conductivity. The optimized composite film exhibited a 6-fold increase in electrical conductivity (0.365 S·cm^–1^) and a 3-fold enhancement in the Seebeck coefficient (16.0 μV/K at ∼300 K) in comparison to PPy/paper films.

**Figure 28:**
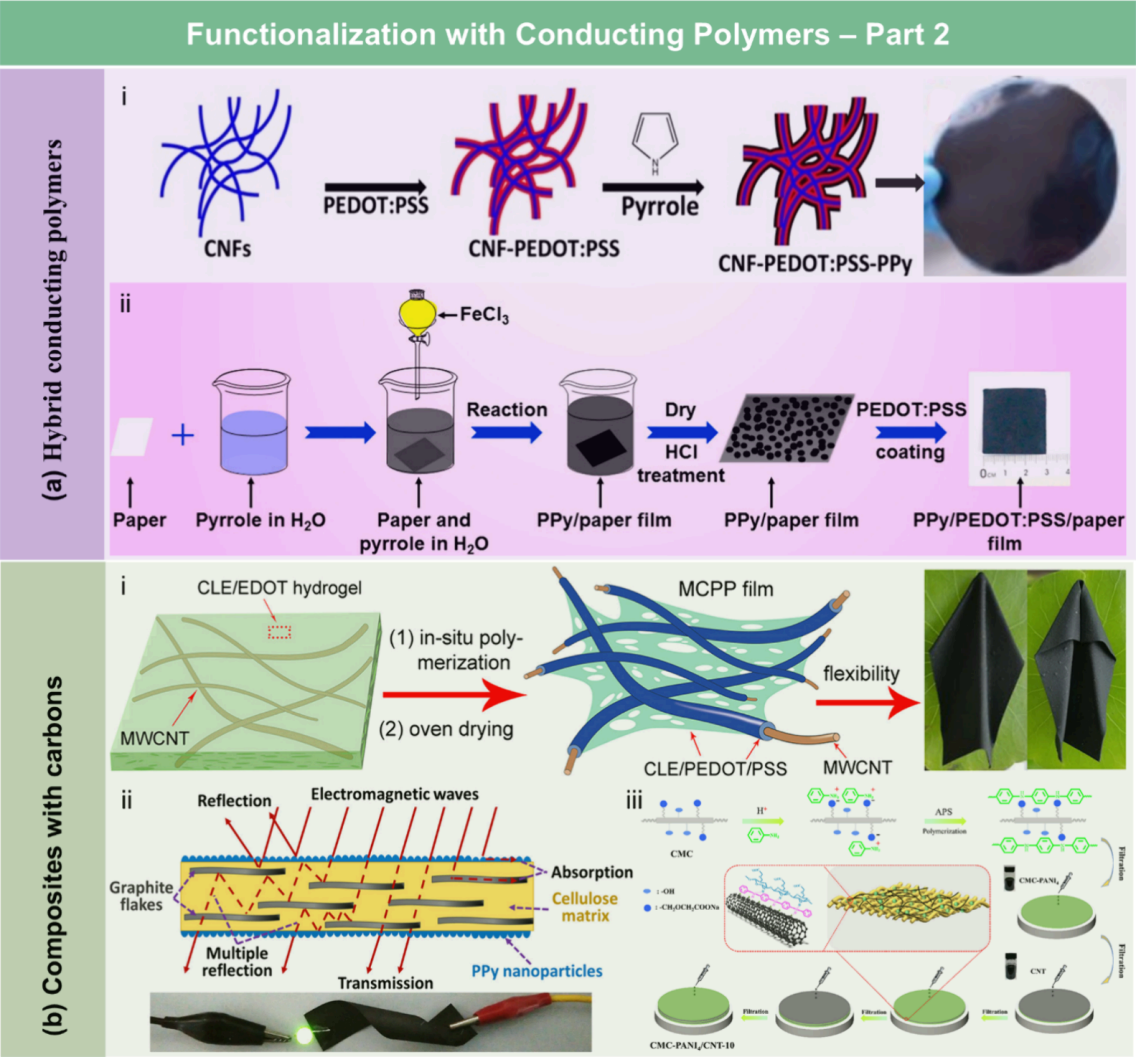
(a) Preparation of CNF-PEDOT:PSS–PPy composite film (i). Adapted with permission from ref 708. Copyright 2023 Elsevier. The fabrication process of the PEDOT:PSS/PPy/paper composite films (ii). Adapted with permission from ref 709. Copyright 2017 MDPI under CC BY 4.0 (https://creativecommons.org/licenses/by/4.0/). (b) Schematic illustration showing the preparation route of MWCNT-reinforced cellulose/PEDOT:PSS (MCPP) films (i). Adapted with permission from ref 710 Copyright 2017 American Chemical Society. Schematic representation of shielding phenomena of the cellulose/GP/PPy film and the conductivity of the film (ii). Adapted with permission from ref 711. Copyright 2015 American Chemical Society. Schematic illustration of the fabrication process of CMC-PANI/CNT film (iii). Adapted with permission from ref 712. Copyright 2022 Elsevier.

To further enhance conductivity and functionality, researchers have incorporated carbon nanomaterials into conductive polymer composites. Zhao et al.^710^ employed an ionic liquid-assisted supramolecular assembly to fabricate PEDOT:PSS/MWCNT/cellulose composite films (MCPP) for flexible supercapacitor electrodes (Figure 28bi). The optimized MCPP film demonstrated excellent electrochemical performance, with a specific capacitance of 485 F·g^–1^ at 1 A·g^–1^, a low resistance of 0.45 Ω, and 95% capacitance retention over 2,000 charge-discharge cycles. When employed in an all-solid-state symmetric supercapacitor, the film exhibited a high volumetric capacitance of 50.4 F·cm^–3^ and outstanding mechanical flexibility, highlighting its potential for next-generation flexible energy storage systems. Chen et al.^711^ synthesized highly thermally stable and conductive cellulose/graphite/PPy composite films via in situ polymerization and vapor deposition to enhance conductivity and heat resistance. The introduction of graphite powder (GP) significantly improved conductivity, achieving 0.55 S·cm^–1^, seven times higher than that of cellulose/PPy composite films. Additionally, the composite film exhibited excellent EMI shielding properties, achieving a shielding effectiveness of up to 30 dB, indicating its suitability for applications in lightweight EMI shielding. Figure 28bii illustrates conductivity trends at different GP contents, PPy deposition amounts, and the EMI shielding mechanism, highlighting the synergistic effect of GP and PPy in enhancing electromagnetic wave absorption and reflection. Similarly, Xu et al.^712^ developed highly flexible and electrochemically active carboxymethyl cellulose-polyaniline/carbon nanotube (CMC-PANI/CNT) composite films as binder-free supercapacitor electrodes, as shown in Figure 28biii. This hybrid structure was fabricated via in situ polymerization and layer-by-layer assembly, allowing PANI nanoparticles to vertically and orderly grow on CMC fibers, while CNTs enhanced conductivity and mechanical stability by creating an interwoven network structure. The optimized CMC-PANI4/CNT-10 composite film exhibited a gravimetric capacitance of 348.8 F·g^–1^ (at 0.5 A·g^–1^) and an areal capacitance of 3106.3 mF·cm^–2^ (at 5 mA·cm^–2^), and it retained 89.2% of its capacitance following 5000 cycles of charging and discharging. At a power density of 400.02 μW·cm^–2^, the assembled symmetric supercapacitor (SSC) achieved a significant energy density of 99.89 μWh·cm^–2^.

## 5 Applications of Lignocellulosic Films

### 5.1 Food Packaging

The barrier properties of LCFs, including water vapor and oxygen impermeability, are critical parameters that determine their suitability as sustainable packaging materials. These aspects have been thoroughly discussed and compared in section 3.3.2, where the intrinsic barrier performance of various types of LCFs was analyzed based on structure, composition, and processing method. In this section, we shift the focus toward the development of active food packaging, which goes beyond passive protection by actively interacting with the food or its environment to prolong shelf life, ensure food safety, and maintain quality.

Active food packaging is designed to control gases (e.g., oxygen, nitrogen, carbon dioxide), water vapor, light, and microbial growth that could lead to food spoilage during storage and transportation. Intelligent food packaging, also known as smart packaging, is designed to monitor the condition of packaged food or the environment surrounding the food.^713^ LCFs have many attractive features for designing active and intelligent food packaging, including flexibility, UV-blocking property, biodegradability, and sustainability. Lignocellulose based antimicrobial, antioxidant and pH/temperature-responsive films have been extensively investigated for active and intelligent food packaging. This section aims to critically analyze recent developments of antimicrobial LCFs for active packaging, lignin-containing cellulose films for active packaging, and colorimetric LCFs for intelligent packaging.

#### 5.1.1 Antimicrobial Lignocellulosic Films for Active Packaging

Antimicrobial materials can be introduced into LCFs via physical mixing or chemical grafting approaches. Thus far, various antimicrobial materials, including metal (e.g., Ag, AgO, CuO) nanoparticles (NPs), carbon dots (CDs), carbon quantum dots (CQDs), metal-organic frameworks (MOFs), polymers with tertiary amino groups, CS and its derivatives, antimicrobial peptides (AMPs), and lauric arginate, have been applied to develop antimicrobial LCFs for active food packaging. Depending on the types, antimicrobial materials can also improve mechanical strength, thermal stability, barrier and UV-blocking properties of LCFs. Metal NPs have broad-spectrum and long-term antimicrobial activities via deactivating microorganisms due to their continuous release of metal ions to microorganisms, and their antimicrobial activities can be enhanced via photocatalysis.^714^ The RC based antimicrobial films were prepared by dissolving cellulose in LiCl/DMAc and then adding 3–7% ZnO NPs.^715^ The RC/ZnO composite film effectively inhibited the growth of *B. cereus*, *S. aureus*, *L. monocytogenes*, *E. coli*, *S. typhimurium*, and *V. para-hemolyticus* bacteria. The addition of 3% ZnO also reduced the oxygen permeability of the RC composite film by 37%, and the addition of 7% ZnO greatly enhanced the thermal stability of the RC composite film. The CMC/ZnO NPs composite films can be readily fabricated without using cellulose solvents such as LiCl and DMAc. The CMC/ZnO NPs composite films were prepared by casting.^716^ The CMC composite film incorporated with 1% ZnO NPs showed strong antimicrobial activity against *E. coli* and *L. monocytogenes*. The addition of 2% ZnO NPs also improved the mechanical properties of the CMC composite film. The combination of ZnO NPs with other NPs such as Ag and CuO can also improve the mechanical properties of the CMC composite film.

Due to the hydrophilic characteristic of RC, the RC/ZnO composite films show poor barrier properties against water/water vapor and poor mechanical strength as exposed to them. To synergistically improve barrier property against water vapor and mechanical strength, Xie et al.^717^ first cross-linked cellulose using epichlorohydrin (ECH) to form a cellulose network and then *in situ* synthesized ZnO NPs on the cross-linked cellulose network by a hydrothermal process to fabricate cellulose/ZnO nanopillars composite film (ZnO NPs@Zn^2+^/Cel) (Figure 29ai). This film showed excellent mechanical strength (tensile, puncture) and water vapor barrier properties. This film had remarkable antimicrobial activities against *S. aureus* and *E. coli* and good preservation of black grape. Without UV excitation, this film inactivated the microbial cells by mechanical rapture of ZnO NPs. After UV excitation, this film inactivated the microbial cells via the synergistic action of photocatalysis and mechanical rupture of ZnO NPs (Figure 29aii).

**Figure 29:**
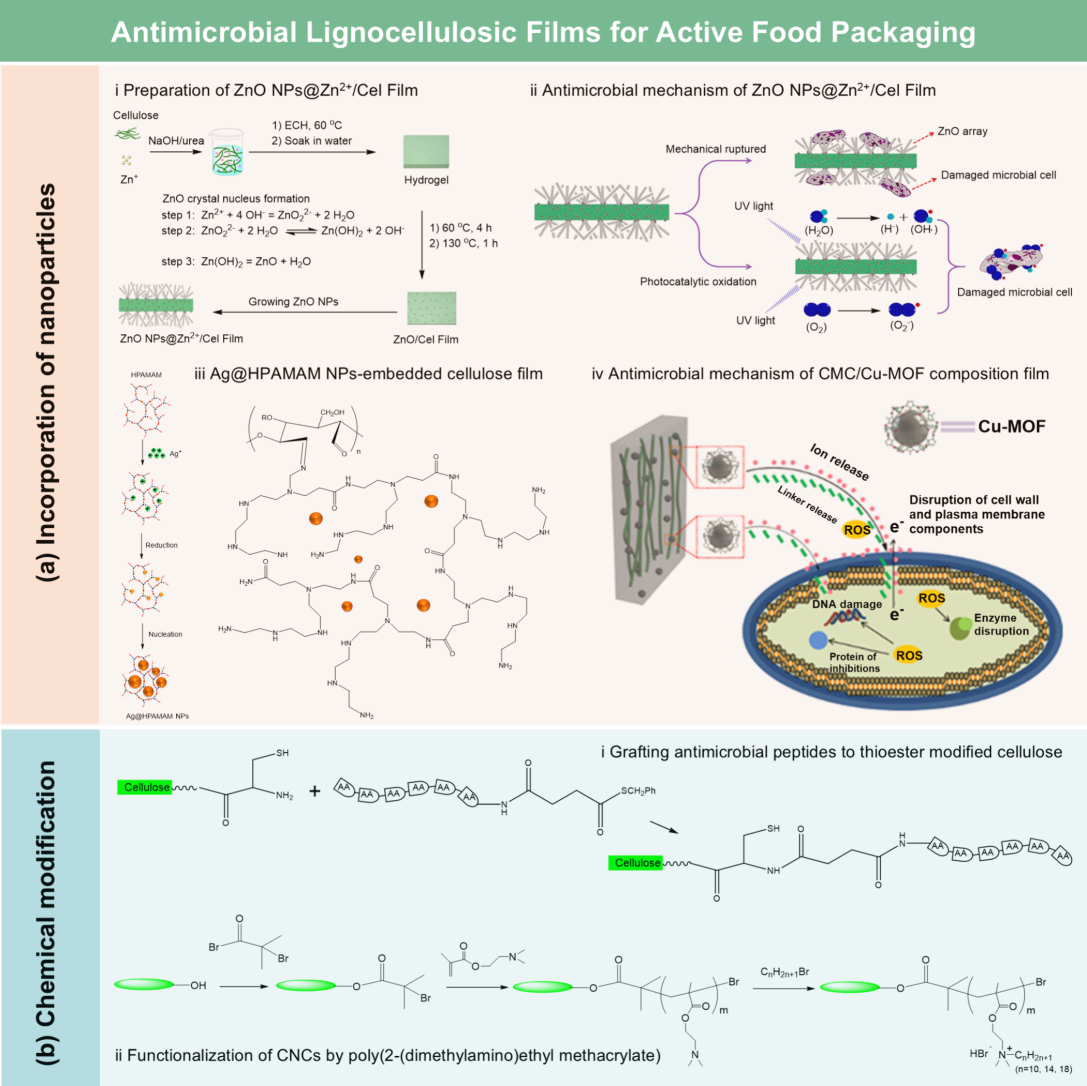
(a) Schematic illustrations of the preparation (i) and antimicrobial mechanism (ii) of the ZnO NPs@Zn^2+^/Cel film. Adapted with permission from ref 717. Copyright 2022 Elsevier. Illustration of developing Ag@HPAMAM NPs-embedded cellulose film (iii). Adapted with permission from ref 719. Copyright 2019 American Chemical Society. Schematic illustration of the antibacterial mechanism of the CMC/Cu-MOF composite film (iv). Adapted with permission from ref 722. Copyright 2023 Springer Nature. (b) Illustration of grafting AMP to the thioester modified cellulose (i). Adapted with permission from ref 726. Copyright 2020 American Chemical Society. Illustration of functionalizing CNCs by poly­(2-(dimethylamino)­ethyl methacrylate) with quaternary ammonium groups (ii). Adapted with permission from ref 727. Copyright 2018 Elsevier.

Metal NPs can adhere to food surfaces and then cause human health issues. To mitigate this, metal NPs should be incorporated into carriers to avoid as much contact as possible with food. Carriers can also prevent agglomeration of metal NPs to produce smaller NPs with stronger antimicrobial properties due to larger surface areas. Additionally, antimicrobial and antioxidant carriers can respectively contribute to antimicrobial and antioxidant activities of cellulose/metal NPs composite films. The antioxidant property helps cellulose/metal NPs composite films to increase the shelf life of food by absorbing oxidizing agents. EI Guerraf et al.^718^ first introduced antimicrobial PANI and poly­(3,4-ethylenedioxythiophene) (PEDOT) to cellulose and then AgNPs using PANI and PEDOT as carriers. The developed cellulose composite film exhibited noticeable antibacterial effects against *E. coli* and *S. aureus* strains via the combined actions of AgNPs, PANI, and PEDOT. This composite film also exhibited strong antioxidant property because PANI was an antioxidant, and its scavenging activity was greatly enhanced by AgNPs via accepting or donating electrons. Alternatively, carriers/metal NPs composites can be grafted onto modified cellulose films. Gu et al.^719^ grafted hyperbranched polyamide-amine (HPAMAM)-embedded AgNPs onto 2,3-dialdehyde cellulose (DAC) film (Figure 29aiii). The resultant Ag@HPAMAM NPs-embedded cellulose film showed a lower silver leakage (<10%) and strong antibacterial effects on *E. coli* and *S. aureus* and effectively extended the storage life of cherry tomatoes. The Ag@HPAMAM NPs also improved the mechanical strength properties of the DAC composite film. Mukta et al.^720^ cross-linked sericin-embedded AgNPs with DAC film via Schiff reaction.

MOFs are effective antimicrobial materials due to metal ions or clusters linked by organic bridging ligands.^721^ Antimicrobial activities and metal ions release of MOFs can be optimized by tuning their compositions and structures. The CMC/Cu-MOF (345–635.5 nm) composite film was prepared by *in situ* synthesis of Cu-MOF with the CMC fiber as supporting medium and its carboxyl groups as reactive sites.^722^ The antimicrobial mechanism of the CMC/Cu-MOF composite film was attributable to the releases of metal ions and ligands from the MOF skeleton (Figure 29aiv). It was hypothesized that the metal ions entered the cell membrane and disrupted cellular components. In addition, the functional groups of organic ligands in Cu-MOF presumably bonded with cations (Ca^2+^ and Mg^2+^) in cell and then ruptured the cell membrane and caused cytoplasmic efflux leading to bacterial death. Due to the weak photocatalytic effect of Cu-MOF, the CMC/Cu-MOF composite film exhibited high photostability. The incorporation of Cu-MOF enhanced the UV-blocking property and mechanical strength of the CMC composite film. Similarly, the CMC/Cu-MOF (HKUST-1) composite film was fabricated using the *in situ* synthesis of Cu-MOF.^723^ The CMC/Cu-MOF composite film was able to completely inhibit the growth of *E. coli*. Metal NPs can be loaded within MOFs to further enhance their antimicrobial activities. The CMC/AgNPs@Cu-MOF (HKUST-1) composite film was also prepared by *in situ* syntheses of Cu-MOF (HKUST-1) and then AgNPs in the pores and/or onto the surfaces of Cu-MOF.^724^ The CMC/AgNPs@Cu-MOF composite film exhibited a much higher antibacterial activity against *S. aureus* growth than the CMC/Ag NPs composite film or the CMC/Cu-MOF composite film. Antioxidants can be incorporated into cellulose/MOFs films to introduce antioxidant properties. Curcumin was introduced to cellulose acetate/K-MOFs film.^725^ The film had antioxidant property and successfully monitored shrimp freshness at different levels.

Organic antimicrobial materials can be potentially less toxic than metal NPs. Li et al.^727^ modified CNCs film via first introducing poly­(2-(dimethylamino)­ethyl methacrylate) (PDMAEMA) and then transferring the tertiary amino groups into quaternary ammonium groups (Figure 29bii). The antibacterial activity of the modified CNCs film against *E. coli* and *S. aureus* was decided by the quaternary group structure. The film containing C10 alkyl in quaternary group exhibited the best antibacterial efficiency (98.6%). Biodegradable antimicrobial materials are environmentally friendly alternatives to nonbiodegradable antimicrobial ones. AMPs are biodegradable and can be used to modify cellulose films via various reactions. Wu et al.^728^ modified DAC film with nisin via Schiff’s base reaction. The nisin modified DAC film exhibited an effective antibacterial activity against *E. coli* and *S. aureus* bacteria and inhibited the adhered bacteria of fresh pork to extend the shelf life. Sperandeo et al.^726^ modified cellulose film with AMP (lasioglossin-III and TBKKG6A) thioesters via chemical ligation reactions (Figure 29bi). The AMP-modified cellulose film was effective against *E. coli* growth. However, AMPs have some limitations such as complex structures, scalable production and expensive. By comparison, CS is more attractive due to its abundance and low cost. Liao et al.^729^ cosolubilized carboxymethyl CS (CMCS) with cellulose in aqueous ZnCl_2_ solution and successfully prepared highly transparent and robust composite films via casting. The obtained Cel/CMCS composite film exhibited excellent antimicrobial ability against *E. coli* and *S. aureus*. Taking the advantage of excellent film-forming capability of cellulose microcrystals, Bajpai et al.^730^ facilely developed the antimicrobial cellulose composite film by casting of the mixture of cellulose microcrystals and CS. Besides physical mixing, CS can be also grafted onto oxidized cellulose films. Wu et al.^731^ grafted CS onto the DAC film via Schiff’s base reaction. The obtained DAC-*g*-CS film showed excellent antimicrobial properties against *E. coli* and *S. aureus* and a better performance than PE in preserving sausage. Food-grade antimicrobial materials are highly desirable. Shahbazi et al.^732^ modified cellulose using food-grade lauric arginate. The lauric arginate-modified cellulose film showed great inhibitory effects against *L. monocytogenes*, *S. enterica*, and *E. coli.*

Antimicrobial and antioxidant properties can be simultaneously achieved by respectively incorporating metal NPs and antioxidants into LCFs. The CMC based antimicrobial and antioxidant films were prepared by incorporating ZnO (1%) and antioxidant curcumin (0.5–1%).^733^ The CMC/curcumin/ZnO composite film showed effective antimicrobial activities against *E. coli* (completely disrupted after culturing for 9 h) and *L. monocytogenes* (reduced by about 2 log cycles after culturing for 12 h) growths and antioxidant activity (about 70–92% 2,2′-azinobis (3-ethylbenzothiazoline-6-sulfonic acid) inhibition). The incorporation of ZnO and curcumin also improved the tensile strength by 16.7% and water vapor barrier property by 9.5% of the CMC composite film. Antimicrobial, antifungal. and antioxidant properties can be also simultaneously achieved incorporating antimicrobial, antifungal and antioxidant CD or CQD into LCFs. Riahi et al.^734^ fabricated the CMC based composite film via integrating with CS-derived CQD for active food packaging. This CMC/CQD composite film showed strong antibacterial activities against *E. coli* and *L. monocytogenes* growths and preserved lemon fruits well without mold growth after 21 days of storage. The CQD also endowed the CMC composite film with antioxidant activity and antifungal activity against *A. niger* and *P. chrysogenum*. CNF/GCD (glucose-derived CD) composite films showed high antimicrobial and antifungal activities against *E. coli*, *L. monocytogenes*, and *A. flavus* growth.^735^ The antimicrobial, antibacterial and antifungal activities of CD can be further improved by introducing nitrogen-containing functional groups such as amide and amine. The CNF/NGCD (nitrogen-functionalized CD) composite film showed stronger antibacterial and antifungal activities than the CNF/CD composite film. The strong antimicrobial activity of the CNF/NGCD composite film was presumably due to the antimicrobial amide and amine functional groups of NGCD. The CNF/CD composite film also showed high antioxidant. As coated with tangerine and strawberry fruits, the CNF/GCD or CNF/NGCD composite films inhibited fungal growth on the fruit surface and extended their shelf life by more than 10 d and 20, respectively.

In summary, the cross-linking of antimicrobial LCFs can enhance their barrier properties against water/water vapor and mechanical strength as exposed to water/water vapor. Metal ions migration of antimicrobial LCFs can be mitigated using polymeric carriers. Antimicrobial activities of MOFs-based antimicrobial LCFs can be tuned and optimized by incorporating metal NPs, antioxidants, and changing compositions and structures of MOFs. Organic antimicrobial materials, especially CS and its derivatives, AMPs, and food-grade lauric arginate, may be less toxic than metal NPs and MOFs. Antioxidant and antifungal properties of antimicrobial LCFs can be introduced using antioxidants and CDs to improve their performance in extending the shelf life of food. In spite of these significant developments, further studies are still needed to address metal ions migration issues, improve water resistance, and evaluate toxicity of antimicrobial LCFs.

#### 5.1.2 Lignin-Containing Cellulose Films for Active Packaging

Because of its unique aromatic structure, lignin has inherent antimicrobial, antioxidant, and UV-blocking properties.^736,737^ Lignin is an effective antioxidant due to its radical-scavenger phenolic structure, and can absorb UV through alpha-carbonyl, biphenyl, and ring-conjugated double bonds functional groups. It is also an effective antimicrobial agent because it can disrupt the cell membrane of bacteria and cause bacteria disorganization and dysfunction (Figure 30a).^736^ Thus, lignin-containing cellulose films have antimicrobial, antioxidant, and UV-blocking properties. Lignin-containing cellulose films can be developed from lignin-containing cellulose by physically blending cellulose with lignin or by chemically cross-linking cellulose with lignin. When physically blending lignin and cellulose, the mechanical strength of the resultant lignin-containing cellulose composite films mainly depends on physical entanglement and hydrogen bonds. Chemical bonding between cellulose and lignin can further enhance the mechanical, barrier and water-resistance properties of the lignin-containing cellulose films. Antimicrobial materials such as metal NPs and polymers can be included in the lignin-containing cellulose films to further enhance their antimicrobial activities.

**Figure 30:**
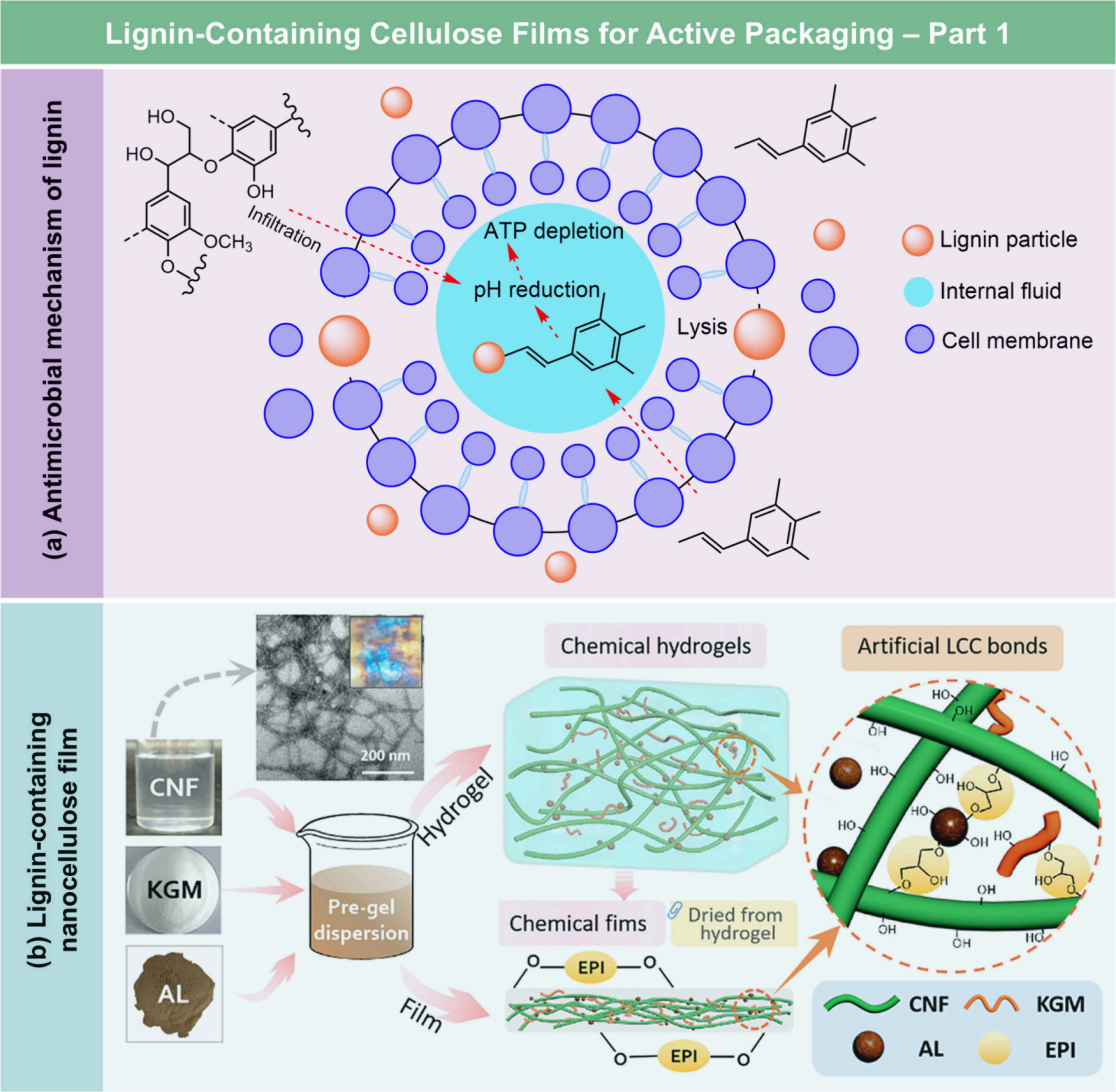
(a) Proposed antimicrobial mechanism of lignin. Adapted with permission from ref 736. Copyright 2024 Springer Nature. (b) Schematic of fabricating lignin-containing nanocellulose film with artificial lignin–carbohydrate complex (LCC) bonds. Adapted with permission from ref 738. Copyright 2022 Elsevier.

Guo et al.^739^ fabricated transparent (around 72–79% at 800 nm) cellulose/lignin composite films by dissolving cellulose (8%) in LiCl/DMAc and then adding 5% lignin (ethanol organosolv lignin, DES lignin, soda/anthraquinone lignin, sodium salicylate hydrotrope lignin) into the resulting cellulose solution. Ethanol organosolv lignin and soda/anthraquinone lignin had more total phenolic hydroxyl groups (3.37 and 3.23 mmol·g^–1^, respectively), thus the cellulose films containing ethanol organosolv lignin or soda/anthraquinone lignin showed greater antioxidant and UV-shielding (280–315 nm) properties. *In situ* lignin regeneration is a facile strategy to fabricate lignin-containing cellulose films, because it does not need to first separate and purify lignin from cellulose and then add it back to cellulose. During the *in situ* lignin regeneration process, cellulose can be fibrillated into micro/nanofibrils and even chemically modified, which can fabricate lignin-containing cellulose films with enhanced mechanical strenght.^370^ Xia et al.^370^ fabricated lignin-containing cellulose film (lignocellulosic bioplastic) via *in situ* lignin regeneration strategy using DES (choline chloride/oxalic acid) as lignin solvent (Figure 31ai). During the regeneration process, the DES dissolved lignin and concurrently fibrillated cellulose fibers into micro/nanofibrils, and oxalic acid partially esterified cellulose. Due to the entanglement of cellulose micro/nanofibrils and lignin-induced adhesion (Figure 31aii), the fabricated film showed a high tensile strength (about 128 MPa). This film also showed excellent UV blocking property (200–400 nm), thermal stability (a 357 °C degradation temperature), water resistance, and biodegradability. Metal NPs and antimicrobial polymers can be introduced to enhance the antimicrobial activities and properties of lignin-containing cellulose films. Zhu et al.^663^ developed lignin-containing nanocellulose antibacterial film via casting of the mixture of ZnO NPs, sodium lignosulfonate (SL) and cellulose nanofibers. The prepared CNF/SL-ZnO film showed an excellent antimicrobial activity against *E. coli* and *S. aureus* growth and effectively extended the shelf life of tomato. Due to the interconnected polymeric structure and more hydrogen bonds among cellulose, lignin and ZnO, the CNF/SL-ZnO films possessed good mechanical properties (20–70 MPa tensile strength), thermal stability (261–292 °C maximum mass loss temperatures), and moisture barrier property (about 9 g·m^–1^·s^–1^·Pa^–1^ WVP) as well as superior biodegradability with negligible side effect on the soil microenvironment. To simultaneously enhance antibacterial and antioxidant activities and other properties (e.g., mechanical strength, thermal, barrier), He et al.^740^ fabricated lignin-containing cellulose antibacterial film (Cel/PL/ε-PL) via casting of the mixture of ε-polylysine (ε-PL, 3%), cellulose (Cel), and anionic phenolated lignin (PL, 10–30%). The addition of ε-PL effectively enhanced the antibacterial activity against *E. coli* and *S. aureus*, antioxidant activity (about 38% DPPH inhibition), tensile strength (75.9 MPa) and water vapor and oxygen barrier properties of the resultant film. The prepared Cel/PL/ε-PL composite film effectively extended the shelf life of shrimp. Water resistance, wet mechanical strength, and properties of lignin-containing cellulose films can be enhanced by cross-linking of lignin with cellulose and/or constructing artificial lignin-carbohydrate complex (LCC) bonds. Guo et al.^280^ cross-linked lignin nanoparticles (LNPs, 50–150 nm) with DAC nanofibers (DACNFs) via condensation reactions to fabricate lignin-containing nanocellulose film. Due to the stable chemical cross-linking between cellulose and lignin, this film showed a long-term hydrophobicity and kept about 18.9% initial tensile strength after immersion in water for 1 h. Shen et al.^741^ developed a strong and flexible lignin-containing cellulose film through the molecular reconstruction of cellulose and lignin to form a chemically cross-linked network using EPI (Figure 31b). The synergistic effect of hydrogen bonding and chemical cross-linking imparted exceptional mechanical strength to the film, achieving a tensile strength of approximately 132 MPa. The film also exhibited excellent water resistance, with a wet strength of around 70 MPa. Halloub et al.^742^ prepared lignin-containing nanocellulose film by cross-linking EPI-modified lignin with EPI-modified CNCs (1–4%, about 300 nm). The EPI modification created a cross-linked structure between cellulose and lignin, which led to enhanced strength (a maximal 4.21 MPa tensile strength and 94 MPa Young’s modulus with 2% CNCs) and UV-blocking (200–450 nm) properties. The film enhanced the shelf life of red cabbage via effectively blocking the UV light.

**Figure 31:**
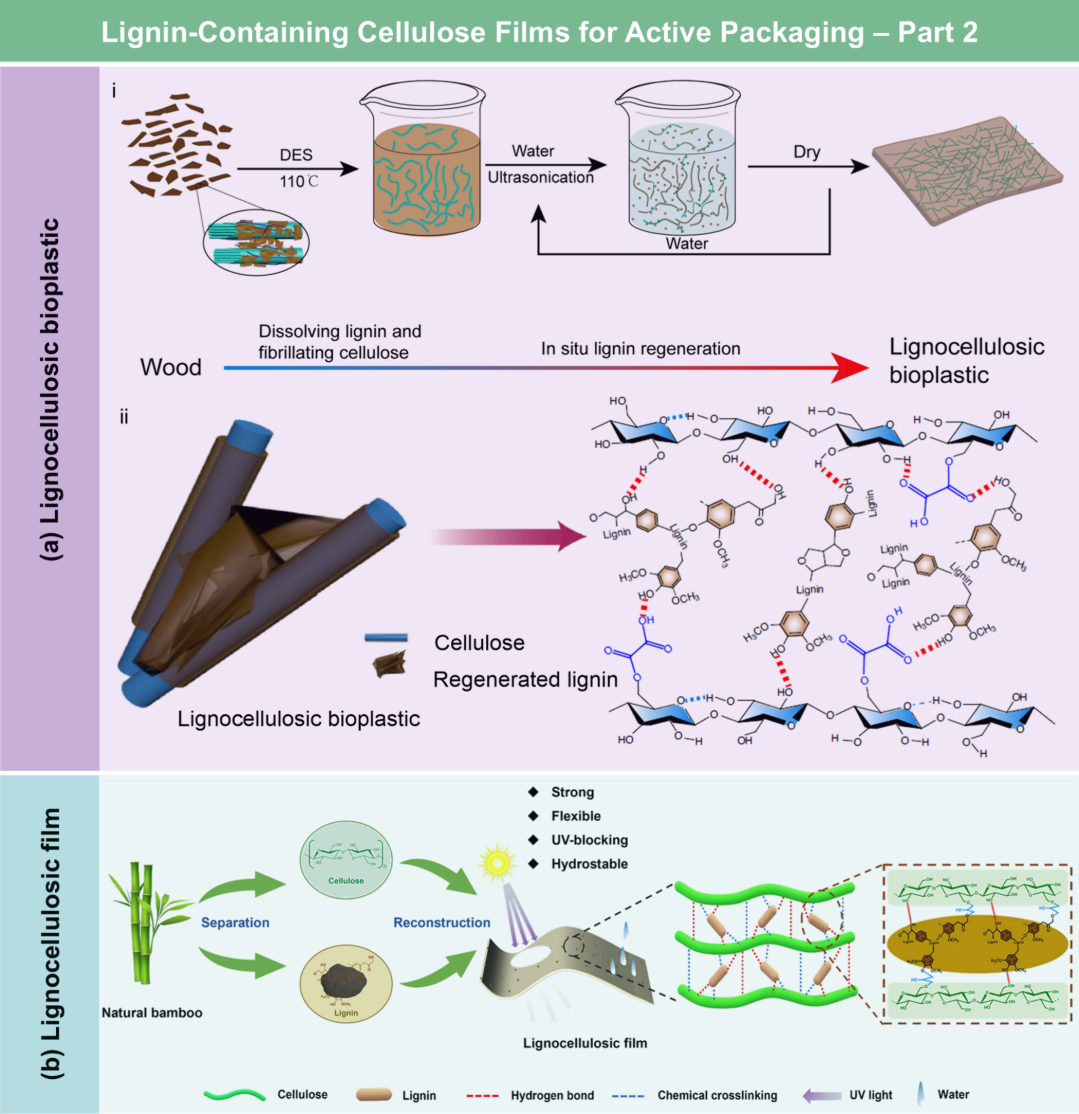
(a) Schematic illustrations of the fabrication (i) and hydrogen bonds of lignin with cellulose and esterified cellulose of lignocellulosic Bioplastic (ii). Adapted with permission from ref 370. Copyright 2021 Springer Nature. (b) Schematic representation of the fabrication of lignocellulosic film via molecular reconstruction of cellulose and lignin to form chemical cross-linking network using epichlorohydrin. Adapted with permission from ref 741. Copyright 2023 Elsevier.

Ma et al.^738^ developed lignin-containing nanocellulose film with LCC and cross-linking bonds using alkali lignin (AL), 2,2,6,6-tetramethylpiperidine-1-oxyl radical (TEMPO) oxidized CNF, konjac glucomannan (KGM) and EPI (Figure 30b). The combined LCC and cross-linking bonds enhanced the mechanical properties of film, with the tensile stress and strain respectively reaching about 110 MPa and 8.27%. The film showed strong water resistance while soaking in water for 180 days.

Lignin-containing cellulose films can be developed directly from lignocelluloses, which eliminates the needs of chemically separating lignin and cellulose and subsequently cross-linking them. Zhou et al.^743^ fabricated the lignocellulosic film via dissolving lignocellulose using a 70% metal salt (ZnCl_2_/CaCl_2_, 20:1, wt/wt) solution. This film showed excellent mechanical strength, water, solvent, and temperature stabilities and biodegradability. This film also showed high tensile strength (136 MPa), high thermal stability (about 310 °C decomposition temperature) and excellent biodegradability. Films made from lignocellulose containing nanocellulose can have better strength and barrier properties. Lignocellulosic film was directly fabricated using lignin-containing cellulose nanofibrils (LCNF) via the combined slow filtration and hot-pressing methods.^744^ This lignocellulosic film showed good strength (12–14 MPa tensile strength) and oxygen barrier property (3.6–3.8 cm^3^·m^–2^·d^–1^ oxygen transmission rates and 2.6–3.1 cm^3^·μm·m^–2^·kPa^–1^·d^–1^) oxygen permeability at 50% RH), water vaper barrier property (125–134 g·m^–2^·d^–1^ WVTR and 5.6–5.7 × 10^–11^ g·m^–1^·s^–1^·Pa^–1^ WVP). Charged chemical groups can be introduced to improve the antimicrobial properties of lignocellulosic films. Sirviö et al.^745^ first prepared lignin-containing cationic wood nanofibers (CWNFs, around 1.5 mmol·g^–1^ cationic group) and then fabricated CWNF film via casting. The CWNF film showed an excellent antimicrobial activity against *E. coli* and *S. aureus* growths, good oxygen barrier property (351–498 cm^3^·μm·m^–2^·atm^–1^·d^–1^) permeability at 50% RH) and good mechanical strength (60–70 MPa tensile strength).

In summary, the separations of lignin and cellulose can be eliminated in developing lignin-containing cellulose films via either in situ lignin regeneration or using lignocellulose. Antimicrobial acidities of lignin-containing cellulose films can be achieved using metal NPs, antimicrobial polymers and introducing charged chemical groups. Water resistance, wet mechanical strength and barrier properties of lignin-containing cellulose films can be simultaneously achieved via cross-linking lignin with cellulose and/or constructing artificial lignin-carbohydrate complex. Further studies are needed to improve water resistance of lignin-containing cellulose films and evaluate their biodegradability if cross-linked.

#### 5.1.3 Colorimetric Lignocellulosic Films for Intelligent Packaging

pH-responsive materials (e.g., shikonin, methyl red, phenol red, chlorophenol red, bromocresol purple, bromothymol blue, flavylium, cyanidin-3-*O*-diglucoside-5-*O*-glucoside) undergo color changes in response to variations in the food’s pH.^746^ Similarly, temperature-responsive materials, such as cyanidin-3-*O*-diglucoside-5-*O*-glucoside and polydiacetylene, exhibit color shifts in reaction to temperature fluctuations. Amine-responsive materials, including fluorescein isothiocyanate, change color in the presence of biogenic amines released during food spoilage. These color changes serve as visual indicators of food freshness and safety. As summarized in Table 8, various pH-, temperature-, and amine-responsive materials have been incorporated into LCFs via adsorption, complexation or chemical bonding to develop colorimetric films for intelligent packaging to indicate freshness and safety of food. These responsive materials can also improve antioxidant activity, antibacterial activity, thermal stability, hydrophobicity, barrier, UV-blocking, and other properties of LCFs.

**Table 8:** Colorimetric LCFs for Intelligent Packaging^a^

lignocellulose type	colorimetric materials	pH change	color change	tested application	ref
cellulose	SHK	5.6 → 6.9	red → light brown → dark purple	fish	^747^

cellulose	AZ	5.9 → 6.5	red → light brown → dark purple	pork	^748^
		5.6 → 4.7 → 3.8	violet → gray → yellow	kimchi	
	ACN		gray → light yellow		
	SHK		pale blue → colorless		

cellulose	MR	5.6 → 5.9	red → yellow	beef	^749^
	BCP		yellow → purple		

cellulose	PR		yellow/orange → red	perch, haddock, catfish	^750^
	CR		wine red → purple		
	BCP		green → blue/purple		

CA/e-CNC	BCP	2 → 10	light yellow → light violet		^751^
CNF	RZ/CD	4 → 7	yellow → brownish brown	shrimp	^752^

CNF	ACN/CD	5.8 → 7.1	red → colorless	fish	^753^
		5.8 → 6.8	red → yellow	pork	
		6.3 → 7.2	red → yellow	shrimp	

HPTEA-cellulose	BB	4 → 8	yellow → blue		^754^
cellulose	CyDG	2 → 9	pink → purple gray/gray		^755^
CMC	PDA/AgNPs		purplish-blue → reddish-purple		^756^
CA	ITC/PPIX		red → yellow → green	shrimp, crab	^25^

a Note: e-CNC, esterified cellulose nanocrystals; HPTEA-cellulose, hydroxypropyltriethylamine modified cellulose; SHK, shikonin; AZ, alizarin; ACN, anthocyanin; MR, methyl red; BCP, bromocresol purple; PR, phenol red; CR, chlorophenol red; RZ, resazurin; BB, bromothymol blue; CyDG, cyanidin-3-*O*-diglucoside-5-*O*-glucoside; PDA, polydiacetylene; ITC, isothiocyanate; PPIX, protoporphyrin IX.

A cellulose colorimetric film was prepared from cellulose by adsorbing a natural pigment shikonin (SHK).^747^ The film changed from red to first light brown and then dark purple when the pH of fish increased from 5.6 to 6.9. The addition of SHK also increased antioxidant activity, thermal stability, and water resistance properties of film. Water-soluble colorimetric materials can give more obvious color changes than alcohols-soluble ones to LCF colorimetric films. Oun et al.^748^ found water-soluble anthocyanin (ACN) made the cellulose film more effective in changing color than alcohol-soluble (AZ) and SHK as exposed to produced lactic acid and acetic acid during kimchi storage. To enhance color sensitivity, Luo et al.^750^ developed cellulose gradient colorimetric films by depositing a continuous gradient of two or three dyes using piezoelectric inkjet-printing. The developed colorimetric film exhibited striking color changes when the fish (perch, haddock, catfish) fillets spoiled during storage.^750^ Two or more colorimetric materials can be more sensitive and accurate than one colorimetric material for food freshness monitoring. A cellulose film containing two colorimetric materials was prepared using methyl red (MR) and bromo cresol purple (BCP).^749^ This film accurately detected the beef decay, with the MR changed from red to yellow while the BCP changed from yellow to purple. The physically adsorbed colorimetric materials can be released from film to cause color stability issues.^748^ To address this challenge, colorimetric materials can be strongly complexed or chemically bond with cellulose nanomaterials or carbon dots and then incorporated into cellulose films. Activated bromocresol purple (a-BCP) was covalently bond with esterified cellulose nanocrystals (e-CNC) and then e-CNC/a-BCP was incorporated into CA to develop cellulose colorimetric film.^751^ The film containing 10% e-CNC/a-BCP showed excellent leaching resistance under acidic conditions. Resazurin was first complexed with carbon dots (R-CD), and the CNF colorimetric film was then fabricated by incorporating R-CD into CNF matrix (Figure 32a).^752^ The CNF/R-CD film showed excellent color stability and rapidly changed from yellow to brownish-red when shrimp began to decompose during storage. The addition of R-CD also improved the thermal stability, hydrophobicity and UV-blocking properties (about 87.7–98.3%) of the CNF film. Similarly, the CNF colorimetric film was developed by incorporating ACN-CD complex into the CNF film.^753^ The incorporation of 1.5% CD and 6% ACN also improved the UV-blocking, antibacterial, and antioxidant properties of the CNF film. Alternatively, colorimetric materials can be chemically bonded with cellulose derivatives. The cellulose colorimetric film was prepared by chemically bonding BB with hydroxypropyl triethylamine (HPTEA) modified cellulose and showed no release of BB.^754^ LCF colorimetric films can also respond to the change of food storage temperature, in addition to the pH change. The cellulose colorimetric film fabricated by adsorbing cyanidin-3-*O*-diglucoside-5-*O*-glucoside (CyDG) showed the color change as a function of food storage temperature.^755^ Temperature-responsive cellulose films can be fabricated to monitor temperature-dependent freshness of food. The CMC-based temperature-responsive film was facilely fabricated by incorporating temperature-responsive polydiacetylene (PDA) and AgNPs (to enhance the sensitivity to temperature change).^756^

**Figure 32:**
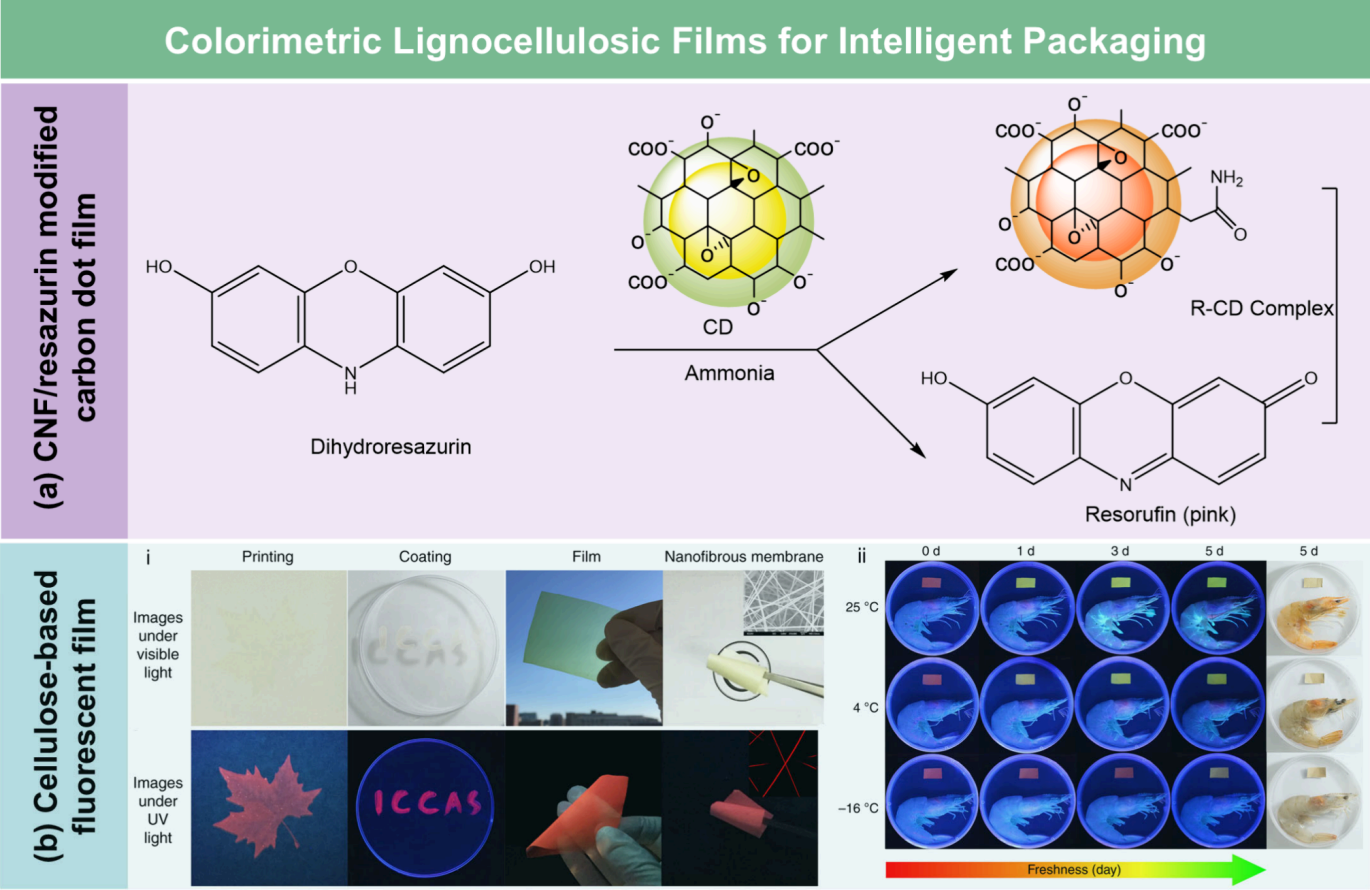
(a) Schematic illustration of the color change tuned by resazurin CD complex (R-CD). Adapted with permission from ref 752. Copyright 2023 Elsevier. (b) Photographs showing the excellent processability of cellulose-based fluorescent film (i); Photographs showing the cellulose-based fluorescent film with a red initial fluorescence as a smart trademark for monitoring the freshness of shrimps stored at different conditions (ii). Adapted with permission from ref 25. Copyright 2019 Springer Nature under CC BY 4.0 (https://creativecommons.org/licenses/by/4.0/).

Amine-responsive fluorescent materials absorb light and then emit their own light, making them brighter, more vivid and more accurate than traditional colorimetric materials. Jia et al.^25^ prepared CA-based amine-responsive fluorescent film by blending green-emitting isothiocyanate (FITC, indicator) functionalized CA with red-emitting protoporphyrin IX (PPIX, internal reference) functionalized CA. The CA fluorescent film can be easily shaped into various forms (Figure 32bi). The FITC component of the CA fluorescent film rapidly reacted with amine to emit green. Based on this mechanism, this CA fluorescent film monitored the freshness of shrimp and crab stored at different temperatures (−16 °C, 4 °C, and 25 °C) by changing the color from red (*I*_red_ ≫ *I*_green_) to yellow and then green (*I*_red_ ≪ *I*_green_) as the spoilage occurred to shrimp and crab (Figure 32bii). Wang et al.^757^ developed amine-responsive fluorescent film via drop-on-demand printing of FITC (indicator) and rhodamine B (internal inference). The film showed high sensitivity to the amines generated from meat spoilage and excellent reproducibility.

In summary, color sensitivity of colorimetric LCFs can be enhanced by using water-soluble colorimetric materials and multiple colorimetric materials. Color stability of colorimetric LCFs can be improved by complexation or chemical binding of colorimetric materials. Temperature-responsive property of lignocellulose colorimetric films can be introduced using temperature-responsive materials. Detection accuracy of colorimetric LCFs can be enhanced via using amine-responsive fluorescent materials. More studies are needed for colorimetric LCFs to further improve the color sensitivity and stability, detection accuracy, and performance in classifying and quantifying different volatile organic compounds from food spoilage.

In conclusion, LCFs have emerged as promising alternatives to petroleum-based films for active and intelligent food packaging. LCFs have advantages in flexibility, UV-blocking property, biodegradability, and sustainability. Even with these advantages, the broad implementation of LCFs for packaging is still challenging due to the inherent unsatisfactory barrier properties and the lignin color issue. Approaches such as chemical modification and barrier coatings are being actively pursued to enhance barrier properties of LCFs. Production scalability, economic viability and recycling also affect the application of LCFs for food packaging.

### 5.2 Flexible Electronics

Flexible electronic devices, including transistors, organic light-emitting diodes (OLEDs), touchscreens, actuators, and EMI shielding systems, require materials that combine excellent optical transparency, mechanical flexibility, thermal stability, electrical compatibility, and environmental resilience. In addition to these baseline performance criteria, increasing emphasis on biodegradability, sustainable sourcing, and functional tunability has accelerated the exploration of biobased materials for such applications.

LCFs, particularly those derived from cellulose (e.g., CNFs, CNCs) based films and lignin-based films, offer an attractive combination of performance and sustainability. Compared with conventional substrates such as polyethylene terephthalate (PET), polyethylene naphthalate (PEN), or indium tin oxide (ITO) glass, LCFs demonstrate several key advantages: a low coefficient of thermal expansion (as low as ∼0.1 ppm·K^–1^) that ensures dimensional stability, nanoscale surface smoothness that enables high-quality thin-film deposition, and high optical transparency (up to ∼90%) suitable for display applications. Moreover, their mechanical compliance allows excellent tolerance to cyclic bending, while their green processability, being derived from renewable biomass, aligns with the principles of sustainable manufacturing. In the following subsections, we explore how LCFs have been applied across five representative types of flexible electronic devices, demonstrating their unique material–function advantages.

#### 5.2.1 Transistors

Transistors are semiconductor devices used to amplify or switch electronic signals and electrical power. They serve as fundamental components in a wide range of electronic devices, including computers, mobile phones, and digital circuits. As the demand for flexible electronics increases, rigid devices are being gradually replaced by their flexible counterparts. Flexible transistors, in particular, have been increasingly proposed for applications in wearable electronics, flexible displays, and sensors, all of which require high mechanical flexibility. Therefore, it is particularly important to find materials with high flexibility for the preparation of flexible thin-film transistors.^758–760^

Flexible LCFs, especially those derived from cellulose derivatives and nanocellulose, have shown significant potential as substrates and dielectric layers in flexible transistors.^761,762^ Their combination of mechanical flexibility, high dielectric constant, thermal stability, optical transparency, nanoscale surface roughness, and low CTE makes them highly suitable for use in organic field-effect transistors (OFET) and oxide thin-film transistors (TFT). Huang et al.^763^ initially reported the successful fabrication of flexible OFET on transparent nanocellulose films with nanoscale surface roughness (Figure 33ai). The fabricated nanopaper-based OFET demonstrated excellent electrical performance, exhibiting only a 10.2% reduction in mobility when the device was bent in a direction parallel to the conduction channel during bending tests. This highlights the mechanical flexibility and robustness of the device.

**Figure 33:**
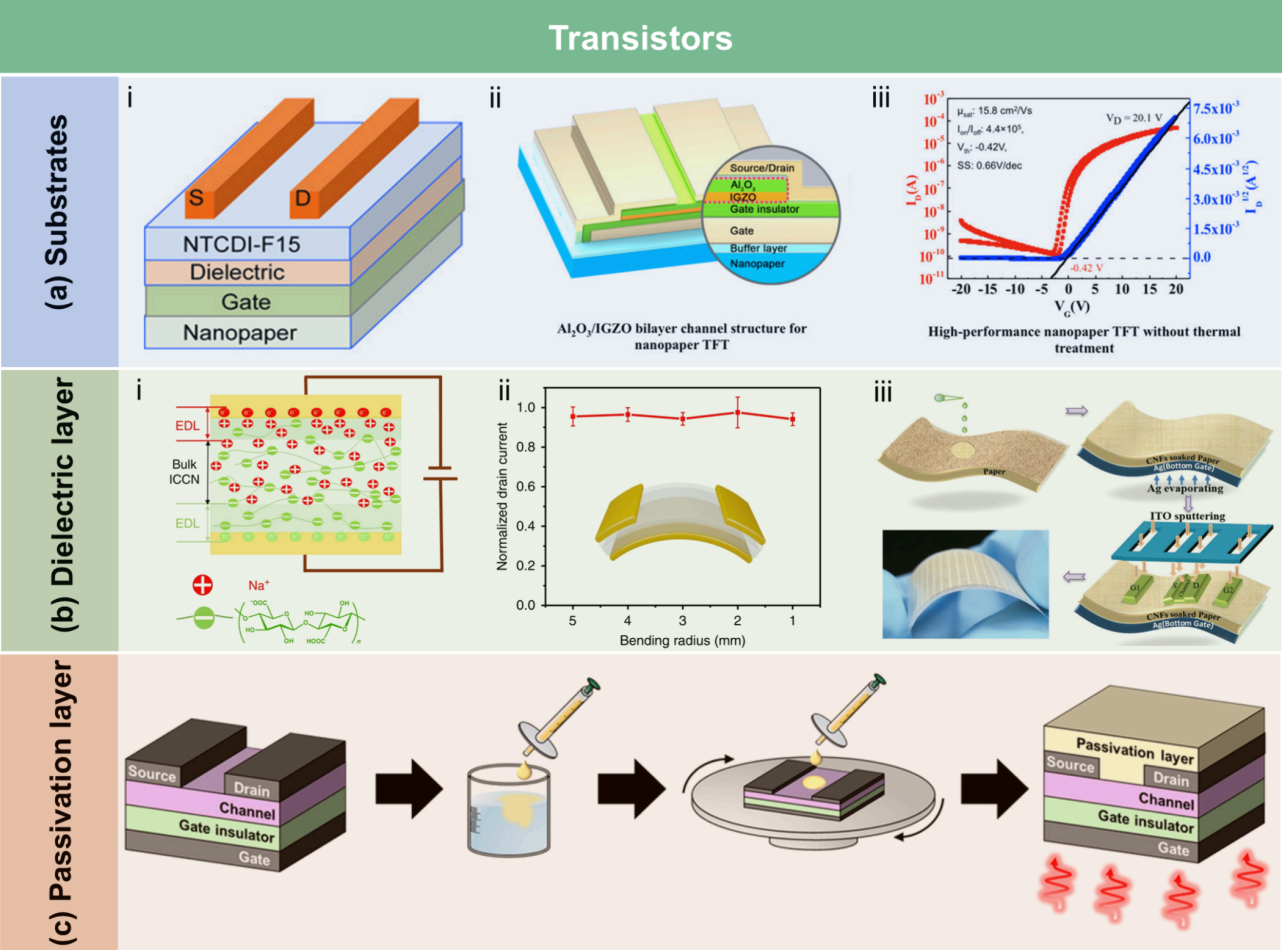
(a) Schematic drawing of the nanopaper organic field-effect transistors (i). Adapted with permission from ref 763. Copyright 2013 American Chemical Society. The structure of nanopaper OFET (ii). The performance of nanopaper TFT (iii). Adapted with permission from ref 765. Copyright 2017 American Chemical Society. (b) Schematic diagram demonstrates the formation of electric-double-layer in ICCNs under the action of electric field (i). Schematic illustration of the ICCN based OFET with a bottom gate top contact architecture (ii). Adapted with permission from ref 764. Copyright 2018 Springer Nature under CC BY 4.0 (https://creativecommons.org/licenses/by/4.0/). The fabrication scheme of flexible TFT with CNF-processed paper gate dielectric (iii). Adapted with permission from ref 766. Copyright 2019 Wiley-VCH. (c) Process to fabricate TFT. Adapted with permission from ref 767. Copyright 2017 American Chemical Society.

Nanocellulose film has also been used as a flexible dielectric material, providing stable performance under bending conditions, which is crucial for wearable and flexible electronic devices. Dai and co-workers^764^ reported the use of nanocellulose film as both a substrate and solid-state dielectric material (ICCN) for low voltage OFET due to its intrinsically ionic conductivity, nanoscale surface roughness (∼0.59 nm), and high transparency (Figure 33bi–ii). The migration of sodium ions, introduced during the TEMPO oxidation process, facilitated the formation of an electric double layer at the interface, thus leading to the high capacitance of the ionic conductive nanocellulose film. This property is crucial for achieving low-voltage operation in OFET. As a result, the OFET fabricated with this nanocellulose film exhibited excellent electrical performance at low operating voltages (below 2 V). The devices maintained stable performance even when bent to a radius of 1 mm, demonstrating both flexibility and durability. However, although progress has been made in combining LCFs with OFET, the low mobility of OFET poses a significant challenge for their use in next-generation displays.

Oxide TFT are regarded as promising candidates for next-generation displays for their advantages such as high carrier mobility and excellent electrical uniformity. However, the high-temperature annealing process typically required for oxide TFT fabrication poses a challenge for the application of LCFs. To obtain high performance oxide TFT on lignocellulose films, room temperature fabrication methods are highly desired. Ning et al.^765^ designed a bilayer channel structure (IGZO/Al_2_O_3_) for fabricating high-performance oxide TFT on nanopaper substrates without thermal annealing (Figure 33aii–iii). The device exhibits a saturation mobility of 15.8 cm^2^·V^–1^·s^–1^, an *I*_on_/*I*_off_ ratio of 4.4 × 10^5^, a threshold voltage (*V*_th_) of −0.42 V, and a subthreshold swing (SS) of 0.66 V·dec^–1^.

Furthermore, lignocellulose films can also be utilized as gate dielectric layer for oxide TFT. Liu et al.^766^ successfully prepared oxide TFTs using paper impregnated with nanocellulose as both the gate dielectric and the substrate (Figure 33biii). The TFT were able to operate at voltages as low as 2.0 V and demonstrated a relatively high *I*_on_/*I*_off_ ratio (7.5 × 10^6^). Notably, the TFT did not show significant electrical degradation at different bending radii. In addition to this, LCFs can also be used as passivation layers. Shin et al.^767^ used nitrocellulose as a passivation layer for amorphous indium gallium zinc oxide thin film transistors (a-IGZO TFTs) (Figure 33c). The a-IGZO TFT with a nitrocellulose passivation layer (NC-PVL) showed improved electrical characteristics and stability compared to oxide TFT without PVL.

Compared to PET and PI substrates, which offer good thermal stability but limited sustainability, CNF-based nanopaper provides a superior platform with nanoscale flatness, high dielectric compatibility, and environmental friendliness. Despite the promising potential, LCFs in transistors face challenges such as limited stability under high voltage. Future research should focus on improving the dielectric properties of LCFs through chemical modification and enhancing their compatibility with other electronic components. The development of hybrid materials and new processing techniques could help achieve better performance in flexible and wearable electronic applications.

#### 5.2.2 Organic Light-Emitting Diodes

OLEDs are devices that utilize organic materials to emit light when an electric current is applied. OLEDs are widely employed in various display technologies, including televisions, mobile phones, and flexible displays, due to their high efficiency, low power consumption, and ability to produce vibrant colors. The core structure of an OLED comprises two electrodes (anode and cathode) with an intermediate layer of organic semiconductor material. At least one of the electrodes must be transparent to facilitate light emission from the device.^768,769^

The application of LCFs in OLED technology has garnered significant attention owing to their high transparency, haze, excellent mechanical flexibility, and thermal stability.^770–774^ Their high transparency makes them suitable for use as substrates in OLEDs, enabling efficient light transmission. The high haze enhances light management, improving the overall efficiency of the light-emitting device. Furthermore, their mechanical flexibility is crucial for foldable and rollable display technologies. Additionally, LCFs have demonstrated potential as hole injection layers in OLED devices, further broadening their applicability in this field.

Transparent cellulose nanofiber films have been used as substrates for OLEDs, providing both flexibility and excellent light transmission. Zhu et al.^775^ used TEMPO-oxidized nanocellulose to prepare films with high transparency (93%), excellent mechanical strength (287 MPa), and high service temperature (up to 200 °C), which were applied as substrates for OLED devices. Compared with traditional plastic substrates. The nanocellulose film substrate can release the stress during bending more effectively resulting in a more flexible and even bendable device (Figure 34a).

**Figure 34:**
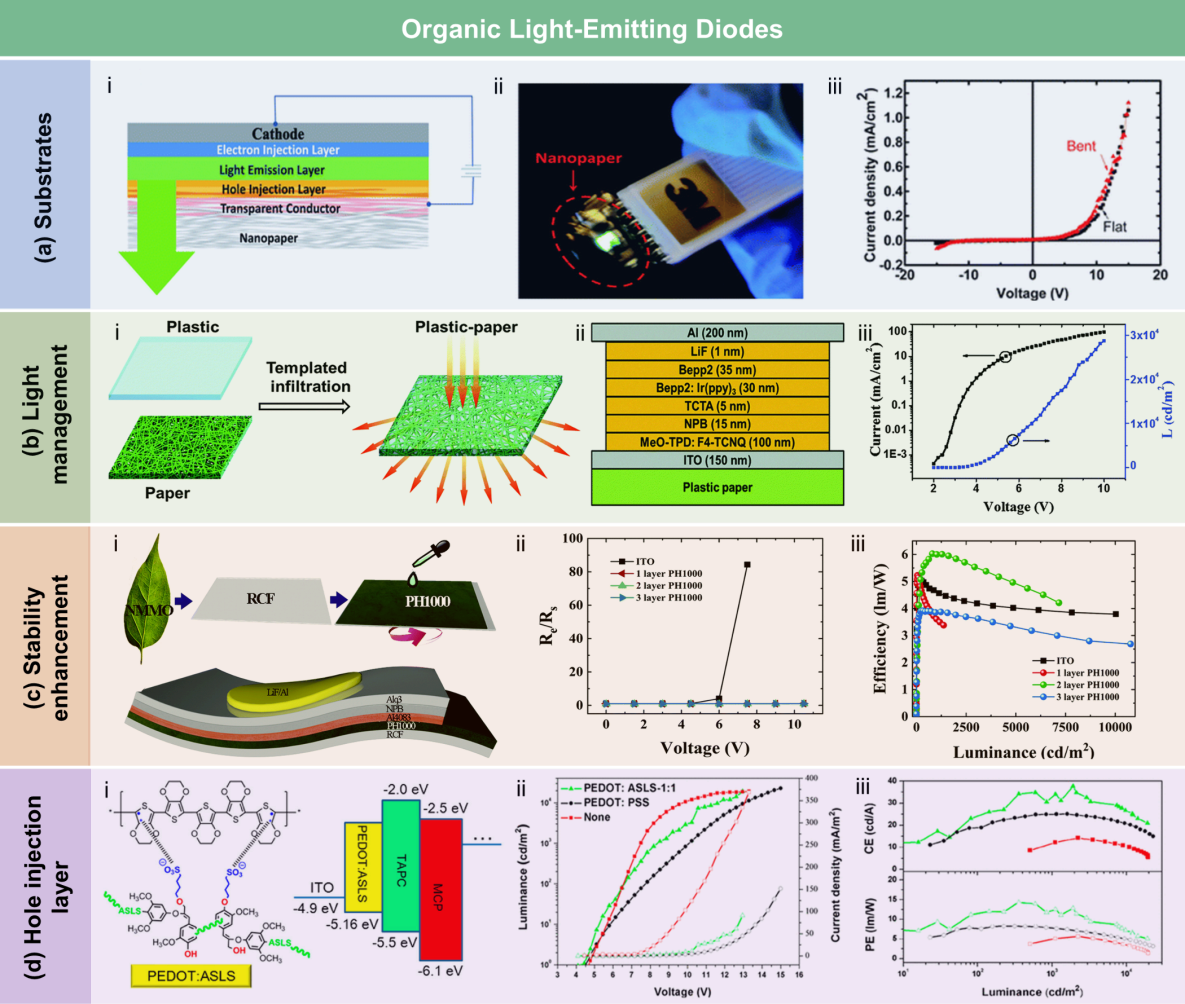
(a) Schematic drawing of a nanopaper OLED device (i). Picture of the OLED in operation (ii). *J*–*V* curve of the flexible OLED in the flat and bent states, respectively. The bending radius is 1.5 mm (iii). Adapted with permission from ref 775. Copyright 2013 Royal Society of Chemistry. (b) Schematic showing the combination of plastic and paper (i). Schematic showing the OLED device structure built on a plastic paper substrate (ii). Current and light output efficiency as a function of OLED operation voltage for the OLED built on plastic paper (iii). Adapted with permission from ref 776. Copyright 2016 Royal Society of Chemistry. (c) Flowchart for the preparation of the PH1000/RCF electrode, and structure of the OLED based on PH1000/RCF (i). Resistance of the electrodes after being energized at different voltages for 30 min (ii). Power efficiency–luminance of OLEDs based on PH1000/RCF and ITO electrodes (iii). Adapted with permission from ref 777. Copyright 2021 Royal Society of Chemistry. (d) As an efficient hole injection layer, sulfobutylated lignin doped PEDOT showed highly enhanced performances in OLEDs (i). Current density *J* versus voltage curve (hollow symbols) and luminescence-voltage curve (solid symbols) (ii). Current/power efficiency versus luminance curves of OLEDs with PEDOT:PSS and PEDOT:ASLS, respectively (iii). Adapted with permission from ref 778. Copyright 2016 American Chemical Society.

To further improve optical management performance, LCFs with high haze have been employed to enhance light scattering in light-emitting devices. Yao et al.^776^ developed a plastic paper using PDMS-cellulose composite, which exhibited over 85% light transmittance and more than 90% haze, along with excellent flexibility and solvent resistance. This plastic paper was applied as a light management layer in OLED devices, leading to significant improvements in both current efficiency and luminous efficiency. Compared to OLEDs fabricated on glass or PE naphthalate substrates, those constructed on this plastic paper demonstrated a 35–50% increase in current efficiency and a 5–20% improvement in luminous efficiency (Figure 34b).

Zheng et al.^777^ have made progress in improving the stability of OLED devices. They prepared transparent conductive electrodes for OLEDs using RC film as substrate and composite PEDOT:PSS. The transparency of the transparent electrode is up to 88%, and the calculated light extraction efficiency of the OLED device from the active layer to the RCF substrate is 59.5%. The transparent electrodes show ultrahigh stability after multiple bending and long-time energization, and the resistance of the electrodes is almost unchanged when energized at 7.5 V for 30 min (Figure 34c).

LCFs also show potential for application as hole injection layers in OLED devices. Li et al.^778^ prepared thin films by doping PEDOT with water-soluble alkyl chain sulfobutylated lignosulfonate (ASLS) as the raw material, which were employed as hole injection layers in OLED devices. These films achieved maximum current and power efficiencies of 37.65 cd/A and 12.84 lm/W, respectively, outperforming devices that utilized PEDOT:PSS as the hole injection layer. The powerful aggregation effect of lignosulfonates and their oxidizing properties further enhance the cavity injection capability and improve the overall efficiency of OLED devices (Figure 34d).

While conventional OLED substrates such as PEN offer mechanical flexibility, they lack sufficient haze and biocompatibility. LCFs, with their high haze and optical transparency, provide a natural means of light management while enabling sustainable and bendable devices. They offer significant potential for OLED technology, providing advantages in terms of flexibility, light management, and efficiency enhancement. Their potential applications as substrates, light management layers, and hole injection layers make them promising candidates for improving OLED device performance. However, challenges persist, particularly in achieving sufficient conductivity while maintaining transparency when incorporating conductive materials. Future research should focus on developing innovative approaches to integrate conductive nanomaterials into LCFs without compromising their optical properties. Moreover, improving the barrier properties of these films will be essential for enhancing the performance and extending the longevity of OLED devices.

#### 5.2.3 Actuators

An actuator is defined as a device that converts energy into mechanical motion, thereby enabling it to perform tasks such as moving or controlling mechanisms and systems. Actuators are employed in a multitude of fields, including robotics, medical devices, soft electronics, and smart wearables. Actuators are indispensable components in systems that necessitate movement or responsiveness to external stimuli, including automated manufacturing equipment, artificial muscles, and adaptive sensors.^779,780^

LCFs have demonstrated considerable potential for use in actuator applications, due to their stimuli-responsive properties, sustainability, and excellent mechanical flexibility. These films can respond to a range of external stimuli, including humidity, light, electric fields, and magnetic fields, making them suitable for incorporation into soft robotics, environmental sensing, and wearable devices.^781–785^ Li et al.^786^ developed a cross-linked cellulose nanofiber (CCNF) monolayer actuator with remarkable humidity responsiveness using environmentally friendly click chemical modification and intercalation-modulated plasticization (IMP) techniques (Figure 35a). This actuator displays excellent water resistance and mechanical properties, as well as noteworthy flexibility and toughness. These materials not only exhibited remarkable electrical conductivity but also demonstrated rapid-response actuation behavior, expanding material options for flexible electronics and soft robotics technologies.

**Figure 35:**
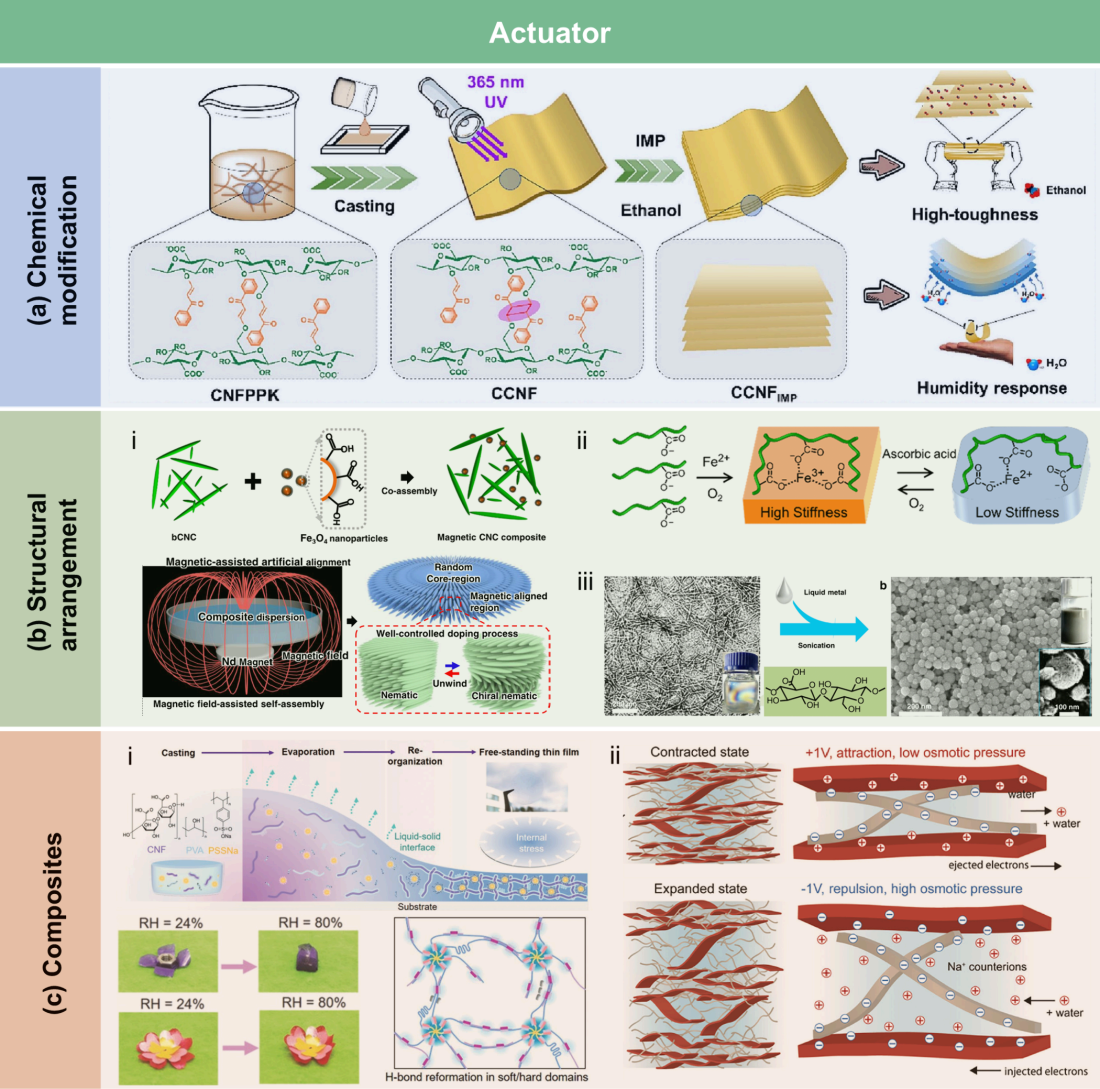
(a) Schematic illustration of fabrication of the CCNF and its high toughness and humidity responsive. Adapted with permission from ref 786. Copyright 2024 Elsevier. (b) Fabrication of magnetic bCNC composites via coassembly of bCNC and Fe_3_O_4_ nanoparticles (i). Adapted with permission from ref 787. Copyright 2022 Springer Nature under CC BY 4.0 (https://creativecommons.org/licenses/by/4.0/). Schematic preparation of the Fe^3+^-CMC gel under aerobic conditions and switchable stiffness changes by reducing and oxidizing chemical agents (ii). Adapted with permission from ref 788. Copyright 2024 American Chemical Society. Evaporation-induced sintering of EGaIn droplets with biological NFs for free-standing films and coatings (iii). Adapted with permission from ref 789. Copyright 2019 Springer Nature under CC BY 4.0 (https://creativecommons.org/licenses/by/4.0/). (c) Preparation and characterization of the cellulose/PVA/PSS (CAS) films (i). Adapted with permission from ref 790. Copyright 2022 Wiley-VCH under CC BY 4.0 (https://creativecommons.org/licenses/by/4.0/). Schematic sketch of the electrochemical osmotic (ECO) hydrogel actuators (ii). Adapted with permission from ref 791. Copyright 2023 Wiley-VCH under CC BY 4.0 (https://creativecommons.org/licenses/by/4.0/).

Recent research has focused on improving the structural arrangements of LCFs by combining them with other functional materials to improve their responsiveness and mechanical properties for use in actuators. For example, Zhang et al.^787^ conducted further investigations into the arrangement and assembly of magnetic nanoparticles-modified bacterial cellulose nanofibers (bCNCs) under the influence of a magnetic field (Figure 35bi). They discovered that high shear rates induced by the magnetic field could promote unidirectional alignment of bCNCs, facilitating a transition from a helical to a columnar (cholesteric) phase. This offers new avenues for the development of actuator materials with rapid response times and high optical transparency. Furthermore, Baretta et al.^788^ developed Fe^3+^/Fe^2+^ cross-linked CMC gels that enable light-triggered, transient mechanical bending, a feature highly beneficial for soft robotics (Figure 35bii). Moreover, Li et al.^789^ have shown that the combination of liquid metal droplets with cellulose nanofibers has resulted in the development of responsive actuators that exhibit remarkable flexibility and rapid actuation capabilities (Figure 35biii).

In addition to modifications and structural arrangements of lignocellulosic materials, the integration of LCFs with other functional materials represents a viable approach. This method not only reduces the response time but also enhances the characteristics and functionalities of composite actuators. Chen et al.^790^ utilized PVA and polystyrenesulfonate (PSS) as polyelectrolyte fillers for cellulose nanofibers (Figure 35ci). The film exhibits reversible anisotropic bending deformation in response to a humidity gradient. The hydrogen bonding network facilitates effective energy dissipation, thereby conferring high toughness during stretching and self-healing capabilities when saturated with moisture. Moreover, Li et al.^791^ combined ionic and electronic conductivity, creating composite films from one-dimensional flexible nanocellulose and two-dimensional MXene (Figure 35cii). The films exhibited electrochemical permeability-driven actuation, whereby low voltages induced rapid and reversible shape changes. This versatility renders the material suitable for potential applications in biosensing, energy storage, and soft robotics.

Synthetic elastomers like PDMS often require high voltages or complex fabrication to achieve actuation. In contrast, LCF-based actuators provide biocompatible, low-energy actuation with comparable strain response, and open up new directions for green soft robotics. Despite these advancements, the utilization of LCFs in actuators continues to be constrained by several challenges, including restricted conductivity, diminished durability under extreme environmental conditions and intricate manufacturing processes that may impede large-scale production. Furthermore, the mechanical properties of LCFs are susceptible to deterioration in high humidity, which restricts their suitability for applications that require long-term stability. Future developments in this field should prioritize enhancing the electrical conductivity of LCFs, improving their environmental stability, and developing scalable, cost-effective manufacturing techniques. In order to fully realize the potential of LCFs in advanced actuator systems, it is essential that innovations are made in the following areas: chemical modification, hybrid material development and structural engineering. Furthermore, the integration of intelligent functionalities, such as self-healing and adaptive responses, into lignocellulosic actuators will facilitate their utilization in next-generation smart devices.

#### 5.2.4 Touchscreens

Touchscreens are interactive displays that enable users to input commands by directly touching the screen. They are widely employed in consumer electronics, including smartphones, tablets, and interactive kiosks. Flexible touchscreens are gaining increasing significance for wearable devices, foldable phones, and other applications where mechanical flexibility is essential. The excellent mechanical flexibility, high transparency, and extremely low CTE (0.1 ppm·K^–1^) of LCFs make them ideal for use in flexible touchscreens.^683,792–795^

Research has focused on enhancing the conductivity and durability of LCFs for touchscreen applications. By integrating lignocellulosic substrates with conductive coatings such as AgNWs or graphene, researchers have developed transparent conductive films suitable for touch panel applications. Fang et al.^796^ designed and assembled a four-wire analog resistive paper touchscreen, utilizing transparent and conductive hybrid paper as the top transparent electrode (Figure 36a). Silver patterns on the carbon nanotube (CNT)-coated transparent paper were created using a screen-printing method and subsequently dried at room temperature. The CNT-coated transparent paper was then employed to assemble the paper touchscreen. This four-wire touchscreen can detect physical touches and transmit signals to a display device. When the screen surface is touched, the contact between the two conductive layers at that point creates a voltage divider, enabling the determination of the *Y*-coordinate from the voltage reading by the *X*-electrodes. The process is repeated with the *X*-coordinate obtained from one of the Y-electrodes, allowing the controller circuit to identify the two-dimensional position of the contact. The word “paper” was successfully displayed on a computer screen when the pattern was drawn on the paper touchscreen using a stylus pen. Additionally, a capacitive touchscreen was fabricated on a highly transparent and clear CNF film using graphene as the conductive material (Figure 36b).^797^ A single layer of graphene, obtained by CVD, was dry-transferred onto the CNF film through van der Waals interactions to prepare a highly transparent (89%) and conductive (445 Ω·sq^–1^) CNF film-based electrode. The resulting capacitive CNF film-based touchscreen not only demonstrates excellent linearity and optical properties comparable to high-performance commercial touchscreens but also exhibits superior mechanical durability compared to commercial touch products. These lignocellulosic-based touchscreens offer a sustainable alternative to conventional indium tin oxide (ITO) films, which are brittle and costly.

**Figure 36:**
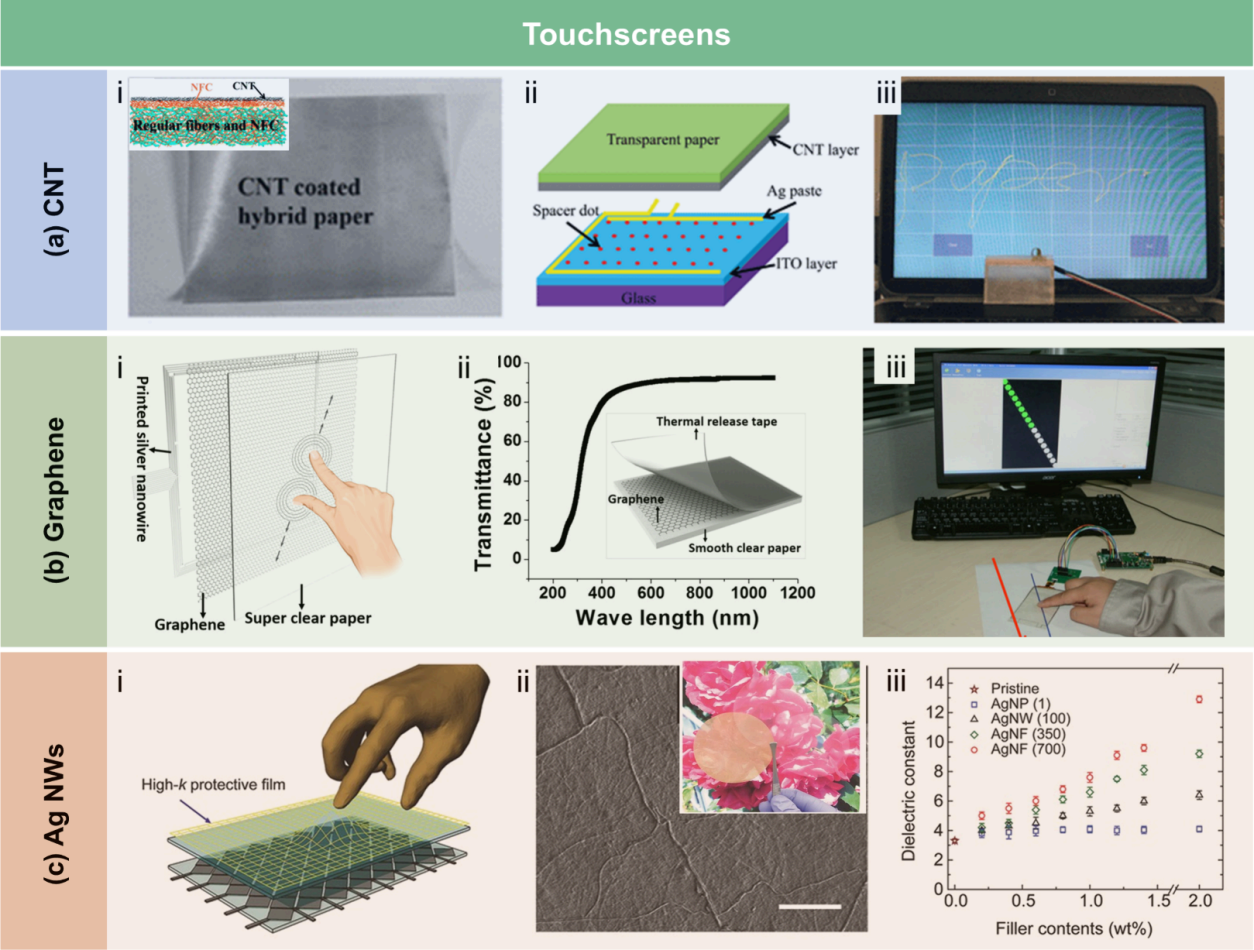
(a) Picture of the hybrid paper after CNT coating based on aqueous ink and a cross-section schematic structure of the transparent and conductive hybrid paper (i); a schematic structure of a transparent four-wire touch screen with nanostructured paper (ii); an assembled paper touch screen was used to simulate typical “paper” with the help of eGalaxTouch software from TVI Electronics LLC (iii). Adapted with permission from ref 796. Copyright 2013 Royal Society of Chemistry. (b) A schematic of paper based multitouch screen (i); optical transmittance of whole device, the inserted schematic shows the dry transferring process for graphene on clear paper (ii); measurement of linearity of paper touch screen (iii). Adapted with permission from ref 797 Copyright 2015 American Chemical Society. (c) Illustration of highly sensitive touchscreen panels (TSPs) integrated with a high-*k* protective cover layer (i); AgNF-CNF film at 1.2 wt % nanofiller contents (ii); dielectric constant of CNF films as a function of nanofiller contents from 0 to 2 wt % at the frequency of 120 kHz (iii). Adapted with permission from ref 799. Copyright 2017 Wiley-VCH.

Additionally, a key metric for wearable devices is the high touch sensitivity of flexible touchscreen panels. High dielectric constants (κ) are crucial for achieving this sensitivity. By maintaining conductive Ag nanowires (NWs) in a nonpercolative state within a nanocellulose network, the dielectric constant of the nanocellulose film substrate, which naturally has a κ value of up to 5.3, can be increased.^798^ This enhancement in the κ value was attributed to the formation of micro capacitor networks formed by neighboring conductive Ag NWs and the insulating nanocellulose between them, as well as the entrapment of free charges at the interface of the Ag NWs and the nanocellulose. This high-κ nanocellulose-based film enabled a printed antenna on its surface to be downsized by half while maintaining the device’s sensitivity and flexibility. Park et al.^799^ further increased the κ value of flexible and transparent nanocellulose films for touchscreen panels by incorporating ultralong Ag nanofibers (Figure 36c). Consequently, the resulting nanocellulose-based film exhibited a high κ value of up to 12.9, significantly enhancing the sensitivity and reliability of the nanocellulose film touchscreen.

Unlike brittle ITO-coated glass or costly synthetic transparent conductors, LCF-based electrodes combine mechanical flexibility, reasonable conductivity, and biodegradability, offering new directions for wearable and foldable electronics. The incorporation of conductive materials into LCFs can enhance their conductivity and dielectric constants, thereby demonstrating the potential to improve device performance when used as substrates. However, challenges in utilizing LCFs for touchscreens include achieving high conductivity while maintaining transparency and mechanical flexibility. Future research should focus on developing novel coating techniques that enable the uniform deposition of conductive materials on LCFs. Additionally, enhancing the mechanical robustness of these films to withstand repeated touch inputs without degradation is a critical area for future development.

#### 5.2.5 EMI Shielding

The process of EMI shielding involves the blocking of unwanted electromagnetic radiation, which has the potential to disrupt the functionality of electronic devices. The necessity of such shielding is evident in a multitude of industries, including telecommunications, medical devices, and aerospace, where it plays a pivotal role in ensuring the dependable functioning of intricate electronic components.^800,801^

LCFs, particularly those comprising cellulose nanofibers, have demonstrated considerable potential for use in EMI shielding applications. Their distinctive combination of flexibility, biodegradability and potential for combination with conductive fillers makes them an excellent candidate for use in next-generation electronic devices.^802,803^ Yang et al.^804^ successfully developed graphene composite films with exceptional EMI shielding effectiveness, conductivity, and thermal conductivity by utilizing NFC-assisted dispersion of graphene nanosheets (GNs) in conjunction with mechanical compression techniques (Figure 37a). This research highlights the potential of lignocellulosic materials in the field of electromagnetic shielding.

**Figure 37:**
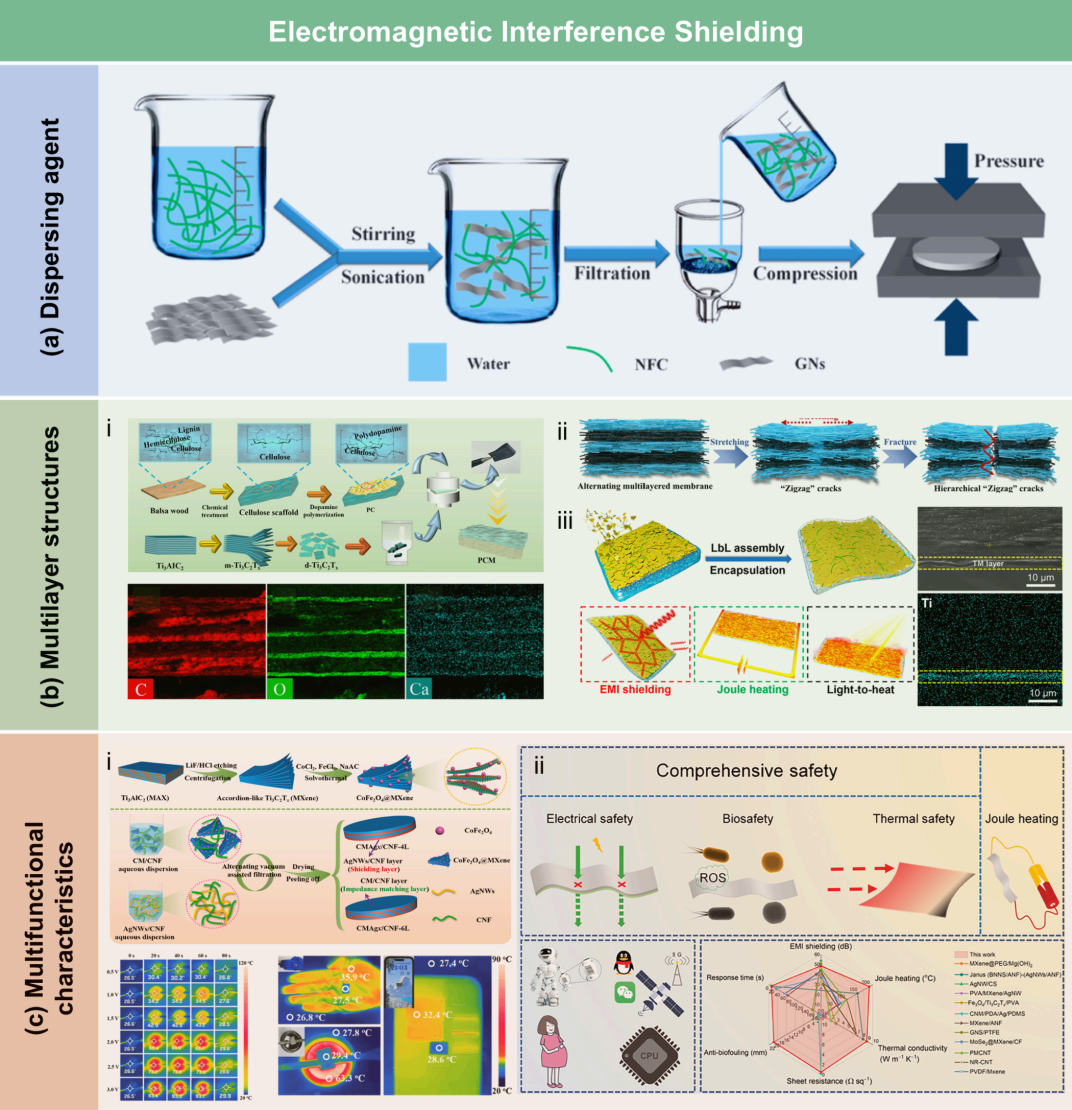
(a) Preparation schematic and EMI shielding performance of the GNs/NFC films. Adapted with permission from ref 804. Copyright 2017 American Chemical Society. (b) Schematic illustration of preparation process of PCM composite films and their properties (i). Adapted with permission from ref 808. Copyright 2024 Elsevier. Schematic diagram of Ca^2+^-precomplexed vacuum-assisted self-assembly process (ii). Adapted with permission from ref 809. Copyright 2024 Springer Nature. Fabrication strategy and functions of silicone- coated multifunctional Si-TM/BC film (iii). Adapted with permission from ref 810. Copyright 2021 American Chemical Society. (c) Preparation and EMI shielding of the alternating multilayered CoFe_2_O_4_@MXene-AgNWs/CNF films (i). Adapted with permission from ref 811. Copyright 2023 Elsevier. Preparation and characterization of the EMI shielding and antibacterial of the preparation of the CNF@BNNS/AgNW/BC composite films (ii). Adapted with permission from ref 812. Copyright 2024 Wiley-VCH under CC BY 4.0 (https://creativecommons.org/licenses/by/4.0/).

The incorporation of conductive materials, including MXene, AgNWs, and CNTs, enables these films to achieve high shielding effectiveness while maintaining the flexibility essential for wearable electronics. The utilization of multilayer structures, comprising alternating layers of CNF and MXene or AgNW composites, has been demonstrated to enhance electromagnetic shielding performance through mechanisms such as multiple reflections, absorption, and reabsorption of electromagnetic waves.^805–807^ This design creates a distinctive “absorption-reflection-reabsorption” shielding behavior, enabling high shielding effectiveness while maintaining a relatively low overall thickness. Liu et al.^808^ developed a bilayer cellulose-based scaffold/MXene composite film (PCM) through the chemical treatment of pine wood, whereby the lignin and hemicellulose were partially removed (Figure 37bi). This innovative film displays excellent mechanical strength (235.28 MPa) and superior electromagnetic shielding effectiveness (44.6 dB), in addition to exhibiting remarkable electrothermal performance. Furthermore, Zeng et al.^809^ developed multilayered CNF&Carbon nanotube/PE oxide (CNF&CNT/PEO) composite membranes through a vacuum-assisted self-assembly process combined with calcium ion precomplexation, achieving a shielding effectiveness of 43.3 dB (Figure 37bii). Similarly, Zhou et al.^810^ fabricated densely packed Ti_3_C_2_ MXene/nanocellulose composite films with an EMI shielding effectiveness of 60 dB, while also imparting photothermal heating properties suitable for ice deicing applications (Figure 37biii).

The multifunctionality of lignocellulose-based films is designed to integrate a range of functions, including electromagnetic shielding, thermal management, antimicrobial properties and environmental adaptability. This broadens their potential applications in wearable electronics and smart devices. Guo et al.^811^ devised a multilayered structure comprising CoFe_2_O_4_@MXene/CNF and AgNW/CNF layers, which exhibited an exceptional shielding effectiveness of 87.8 dB (Figure 37ci). Additionally, it demonstrated excellent infrared stealth performance, rendering it a suitable choice for deployment in a variety of situations. Similarly, Li et al.^812^ employed a simple sequential vacuum filtration technique to fabricate a sandwich-structured electromagnetic shielding composite film comprising cellulose nanofibers@boron nitride nanosheets/silver nanowires/bacterial cellulose (CNF@BNNS/AgNW/BC) (Figure 37cii). The film not only exhibits remarkable electromagnetic shielding effectiveness but also integrates electrical, biological, and thermal safety, thereby underscoring its extensive potential for application in wearable electronics.

Traditional metal-based EMI shielding materials achieve high shielding but are heavy, rigid, and opaque. LCF composites, particularly those integrated with MXene or AgNWs, balance shielding effectiveness with lightweight, flexibility, and multifunctionality including thermal and biological safety. However, the development of LCFs for EMI shielding poses several challenges, particularly in achieving high shielding effectiveness while maintaining flexibility, transparency, and lightweight properties. While the incorporation of conductive fillers can enhance EMI shielding performance, it may also lead to increased material density, reduced flexibility, and higher costs. Additionally, the interfacial bonding between these fillers and the lignocellulosic matrix is crucial for ensuring stable performance. Future research should concentrate on improving the compatibility between lignocellulosic substrates and conductive fillers through innovative interface engineering techniques. For instance, sequential reinforcement of intra- and interlayer interfaces has demonstrated the potential to enhance both the mechanical and electrical properties of multilayer composites, thereby making them more effective for EMI shielding applications in green electronics. Moreover, there is growing interest in integrating functionalities such as thermal conductivity, Joule heating, and infrared stealth into lignocellulosic EMI shielding films, thereby broadening their applicability in wearable electronics and military applications. Ultimately, the future of these films lies in developing materials that provide high shielding effectiveness while addressing safety, environmental impact, and multifunctionality. Recent advancements, such as the incorporation of antibacterial agents and the optimization of thermal management properties, indicate that researchers are making significant strides toward safer and more practical wearable electronic devices.

### 5.3 Energy Storage Systems

Electrochemical energy storage systems are essential for advancing sustainable energy, particularly in the emerging era of widespread adoption of the Internet of Things, flexible/wearable devices, electric vehicles, and large-scale electrical storage.^813–815^ Recently, lignocellulosic materials have been explored as promising environmentally friendly alternatives to conventional petroleum-based materials utilized in various energy storage systems (such as supercapacitors (SCs) and Li systems). The unique 1D fibrous structure and chemical functionalities of lignocellulosic materials render them suitable for applications such as functional substrates, electrode components, and separators, among other uses. The following sections present examples of lignocellulosic materials utilized in various energy storage systems, focusing on components, as well as the preparation and performance (Figure 38).

**Figure 38:**
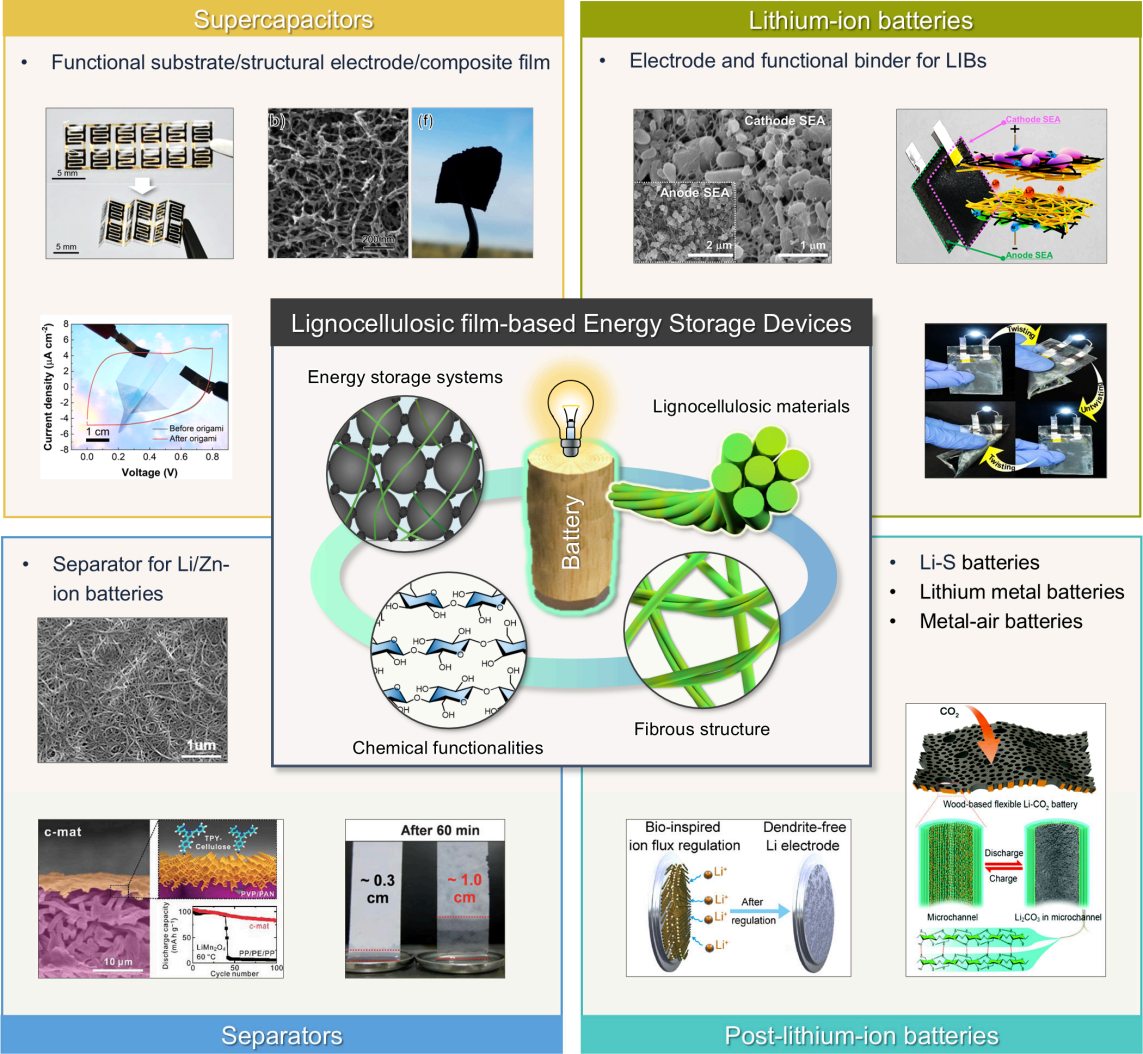
Schematic illustration of energy storage and conversion technologies using lignocellulosic film-based materials, highlighting supercapacitors, lithium-ion batteries, separators, and advanced postlithium-ion battery systems.

#### 5.3.1 Supercapacitors

SCs have been explored as promising energy storage systems because of their simple manufacturing process, fast charging/discharging rates, and stable cycle performance.^816,817^ SCs can be categorized into two systems based on their energy storage mechanism: (i) electrical double-layer capacitors store energy through the reversible adsorption of ions onto the surface of the electrodes, and (ii) pseudocapacitors involve a faradaic mechanism that includes reversible surface redox reactions.^818,819^ However, the development of high-performance SCs has been constrained by the limitations of conventional materials, such as petroleum-derived polymers, fossil-based carbon frameworks, and synthetic substrates, which often suffer from poor flexibility, weak interfacial adhesion, insufficient porosity, and reliance on toxic processing methods. These issues hinder their suitability for flexible, miniaturized, and environmentally sustainable devices. For instance, PET lacks deformability and surface functionality; fossil-derived carbon electrodes offer limited pore structure and tunability; and many binders or composites fail to maintain stable interfaces under mechanical or electrochemical stress.

Lignocellulosic materials, derived from abundant and renewable biomass, have emerged as multifunctional platforms to address these challenges. Their 1D fibrous morphology and hierarchical porosity enable flexible, foldable substrates with enhanced ion transport and surface modifiability. Rich in hydroxyl and carboxyl groups, these materials also provide a chemically compatible framework for the uniform integration of conductive nanomaterials, such as MXenes, CNTs, and conductive polymers, via hydrogen bonding, electrostatic interactions, or π–π stacking, thereby improving both conductivity and structural cohesion.

These features position lignocellulosic materials not merely as green alternatives, but as versatile building blocks for sustainable, high-performance supercapacitor systems. While many demonstrations remain at the proof-of-concept stage, growing evidence highlights their capacity to overcome longstanding challenges in both material development and device integration. This section highlights representative studies that demonstrate how lignocellulosic materials have been applied as substrates, electrodes, and composites in supercapacitor systems, and how their material characteristics contribute to overcoming the limitations of conventional components. The research of lignocellulosic materials in SCs is categorized in Figure 39ai. Lignocellulosic-derived films and 3D porous structure have been explored as structural substrates and composite frameworks, owing to their mechanical resilience and surface reactivity. Moreover, surface-engineered lignocellulosic materials have been developed into multifunctional electrode films, combining mechanical flexibility with electrochemical activity (Figure 39aii).

**Figure 39:**
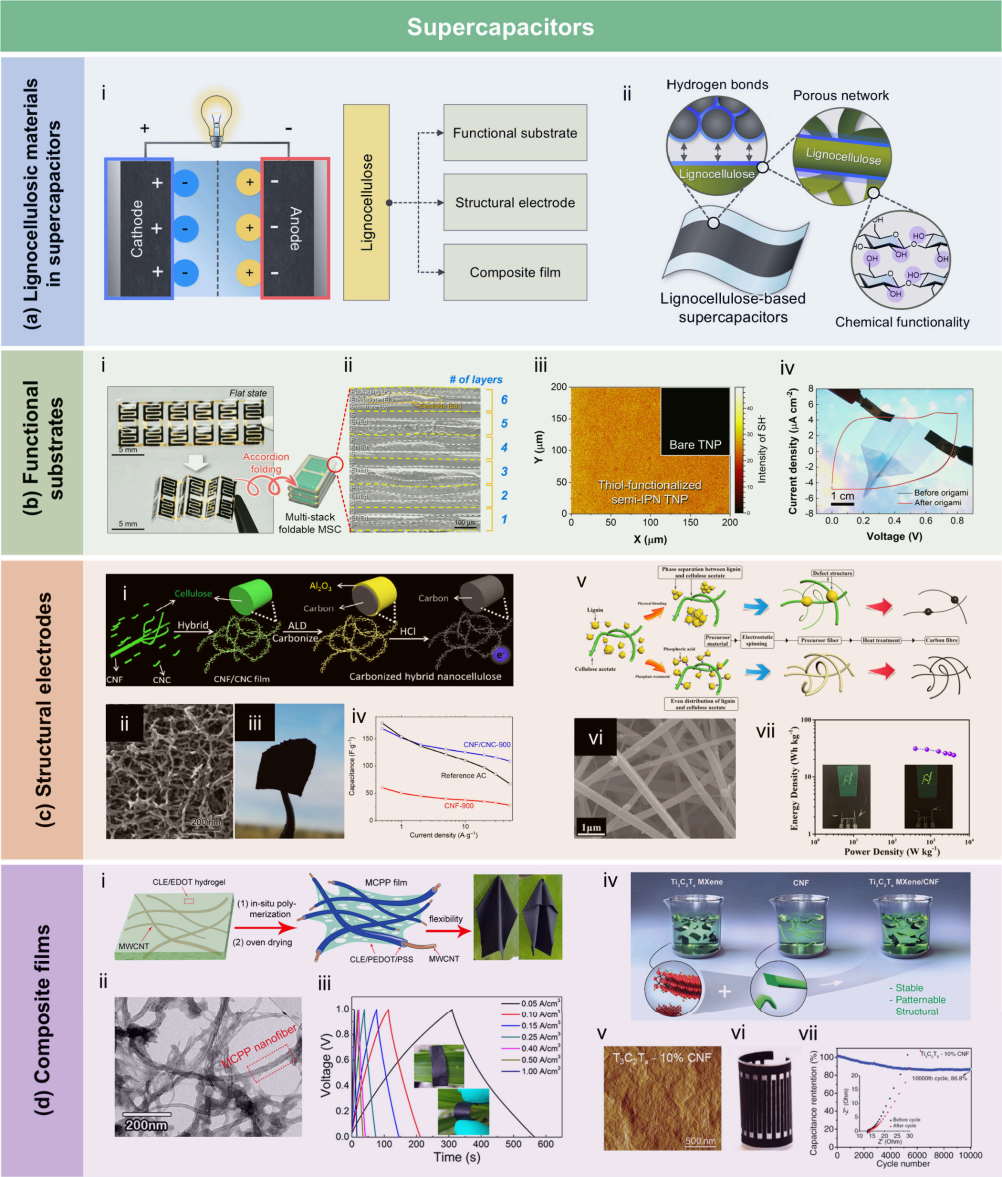
Supercapacitors based on lignocellulosic materials. (a) The development of lignocellulosic materials for structural substrates, carbon materials, and current collectors in supercapacitors (i). Schematic illustrations depicting the characterization of lignocellulosic materials (including chemical functionality, porous network structure, and abundant hydrogen bonds) (ii). (b) Functional substrates. Photograph of the af-MSC array on the CNF substrate (i). Cross-sectional SEM image of the af-MSC illustrating the dense stacking of its individual cells (ii). Adapted with permission from ref 821. Copyright 2023 Wiley-VCH GmbH. TOF-SIMS image of SH^–^ fragments in the thiol-functionalized CNF-based transparent substrate (iii). CV profile (at a scan rate of 50 mV·s^–1^) along with a photograph (inset) showing the SCs in the origami airplane-shaped configuration (iv). Adapted with permission from ref 822. Copyright 2022, Wiley-VCH GmbH under CC BY 4.0 (https://creativecommons.org/licenses/by/4.0/). (c) Structural electrodes. Depiction of the fabrication process for carbonized hybrid nanocellulose (i). Scanning He-ion microscopy of the hierarchical structure of the carbonized film (ii) and photograph (iii). Rate capability of the carbonized hybrid nanocellulose film shown as specific capacitance vs current density (iv). Adapted with permission from ref 826. Copyright 2017, Springer Nature. Diagram illustrating the mechanism of phosphoric acid-functionalized carbon fiber (v). SEM image of the carbon fiber (vi). Ragone plots for the supercapacitor, with the inset photograph showing a series fiber supercapacitor powering an LED (vii). Adapted with permission from ref 827. Copyright 2020 American Chemical Society. (d) Composite films. Schematic illustration of the fabrication process for the MCPP films; photographs depicting the flexibility of the MCPP (i). TEM image of the cellulose/PEDOT:PSS matrix attached to MWCNT surface (ii). Electrochemical performance of the MCPP-based flexible SC (iii). Adapted with permission from ref 710. Copyright 2017 American Chemical Society. Schematic representation of Ti_3_C_2_T_*x*_ MXene/CNF hybrid dispersion (iv). AFM image of the surface of the Ti_3_C_2_T_*x*_-10% CNF film (v). Digital photograph of a roll status of Ti_3_C_2_T_*x*_-10% CNF-based microsupercapacitors (vi). Cycling performance of Ti_3_C_2_T_*x*_-10% CNF-based microsupercapacitors at a constant charge-discharge current density of 0.57 mA·cm^–2^ (vii). Adapted with permission from ref 830. Copyright 2019, Wiley-VCH under CC BY 4.0 (https://creativecommons.org/licenses/by/4.0/).

The practical utilization of conventional microsupercapacitors (MSCs) is constrained by their low areal energy density. To address this problem, prior studies have attempted to enhance energy density by utilizing thick electrodes, but these approaches face issues such as poor mechanical stability during production and uneven electrochemical reactions.^820^ Lee et al.^821^ developed a foldable MSC via an accordion folding strategy, introducing a novel approach called high areal energy density micro power sources (Figure 39bi). Achieving multifolding strategies necessitates substrates with suitable mechanical properties. Conventional synthetic polymers such as polyethylene terephthalate (PET) are insufficient in flexibility, surface roughness, and adhesion. The designed cellulose nanofiber (CNF) substrate incorporates a zwitterionic plasticizer (betaine) to enhance mechanical strength and flexibility beyond that of the PET film. The MSCs were printed in a confined area of 22.75 mm^2^ to minimize intercell distance and maximize electrode fill factor. The integrated 12 MSC unit cells demonstrate high space utilization, achieving an areal capacitance of 180.7 mF·cm^–2^ and an areal energy density of 89.2 μWh·cm^–2^ (Figure 39bii). Kim et al.^822^ presented a novel class of transparent nanocellulose paper MSCs, surpassing conventional synthetic materials. Via a polycondensation reaction, the transparent CNF substrate reacted with 3-mercaptopropyltrimethoxysilane (MPTMS), forming thiol-functionalized CNF substrate capable of establishing intermolecular bonds with neighboring AgNWs conductive layers. Time of flight secondary ion mass spectrometry (TOF-SIMS) 2D mapping images exhibited the uniform dispersion of thiol groups across the CNF substrate (Figure 39biii). These thiol-functionalized CNF-based transparent SCs offer high optical transparency (∼85%) and mechanical flexibility (foldable into a paper plane), outperforming previously reported synthetic transparent MSCs (Figure 39biv).

In addition to functional substrates, high-specific-surface-area electrodes are crucial for creating high-performance SCs.^823,824^ Lignocellulose, with its inherent porosity and large specific surface area, offers ideal characteristics for this purpose. Furthermore, their surface is abundant in hydroxyl groups, rendering it suitable for various chemical functionalities.^825^ Li et al.^826^ incorporated CNFs and CNCs to construct a two-level hierarchical porous structure, where long CNFs formed a microporous framework and short CNCs assembled around it to create interconnected micro- and mesopores structure. This architecture, with a specific surface area of 1,244 m^2^·g^–1^, was maintained during carbonization through the application of a thin atomic layer deposition (ALD) conformal coating (Figure 39ci). The resulting carbonized hybrid nanocellulose film served as a highly conductive, free-standing electrode for supercapacitors, delivering a specific capacitance of 170 F·g^–1^ at a high current density of 50 A·g^–1^ (Figure 39cii and Figure 39ciii). This performance surpassed that of reference activated carbon-based supercapacitors (Figure 39civ). In another structural electrode study, Cao et al.^827^ investigated the use of a simple phosphating process to modify CA and lignin, resulting in precursor fibers designed for energy storage applications. A spinning solution was prepared by mixing poplar lignin, H_3_PO_4_, and CA (Figure 39cv). The interaction between lignin’s phenolic groups and H_3_PO_4_ improved the solution’s spinnability and the fibers’ thermal stability. These precursor fibers maintained a balance of flexibility and strength, preserving their fibrous structure and well-developed pores during preoxidation and carbonization. The resulting carbon fibers (CFs) exhibited a high specific surface area of 837.4 m^2^·g^–1^ and a pore volume of 0.49 m^3^·g^–1^ (Figure 39cvi). When used in a supercapacitor device with a 1 M Na_2_SO_4_ electrolyte, the CFs achieved an energy density of 31.5 Wh·kg^–1^ at a power density of 400 W·kg^–1^, demonstrating excellent performance and stability (Figure 39cvii). This innovative method offered an efficient and sustainable approach for producing high-performance biomass-derived CFs, showing great promise for advanced energy storage systems.

Lignocellulosic material-based composites have emerged as promising materials with diverse applications in energy storage systems. Notably, cellulose composites incorporating conductive materials such as poly­(3,4-eth­yl­ene­di­oxy­thio­phene):poly­(styrenesulfonate) (PEDOT:PSS),^828^ and MXenes (Ti_3_C_2_T_*x*_) nanosheets^829^ have been reported for supercapacitors. The interaction between these composite materials is driven by hydrogen bonds and van der Waals forces, synergistically optimizing the performance of the supercapacitors.

PEDOT, a conductive polymer, is recognized as an attractive material for supercapacitor electrodes because of its flexibility, high electrical conductivity, and pseudocapacitive charge-storage properties.^828,829,710^ However, conventional polymerization processes of PEDOT:PSS have encountered challenges such as low electronic conductivity and insufficient pore structure during scale-up. Zhao et al.^710^ addressed these issues via in situ synthesis combined with nanocellulose, introducing multiwalled CNT (MWCNT)-reinforced cellulose/PEDOT (MCCP) composites for high-performance SCs (Figure 39di). By employing a CNF/EDOT supramolecular assembly with BMIMCl, an ionic liquid, they achieved homogeneous processing of both materials, resulting in a CNF/PEDOT:PSS composite matrix (Figure 39dii). This method yielded a higher electronic conductivity film of 30 S·cm^–1^ compared to that of the conventional dipping polymerization method. An even higher conductivity of 275 S·cm^–1^ was achieved by adding MWCNTs. The resulting MCCP film, fabricated through in situ polymerization, demonstrated uniformly distributed mesopores (∼20 nm), excellent flexibility, and electrochemical stability. The SC composed of the MCCP electrodes exhibited outstanding flexibility and a high volumetric capacitance of 50.4 F·cm^–3^ at a current density of 0.05 A·cm^–3^ (Figure 39diii).

MXenes have garnered significant attention in research for their remarkable electronic conductivity of up to 10,000 S·cm^–1^ and dispersion stability, establishing them as critical materials in the development of SCs and electronic devices.^831,832^ Tian et al.^830^ fabricated flexible SCs based on CNF/MXene hybrid nanocomposites. By scaffolding and binding 2D Ti_3_C_2_T_*x*_ MXene flakes with CNFs that have a thin width of 3.5 nm and micrometer-long structures, the hybrid nanocomposites effectively maintained the conductive pathway while achieving strong mechanical properties. The MXene/CNF composite was obtained by simply mixing colloidal dispersions, utilizing the negatively charged nature of both MXene and CNF to ensure uniform and stable dispersibility in water (Figure 39div). After drying the dispersion, the fabricated composite electrode exhibited strong mechanical properties. These properties are attributed to the robust interfacial interaction between the surface functional groups (−OH, −O, −F) of MXene flakes and the hydroxyl and carboxylic groups of CNFs, as well as the geometrical synergy of the 1D CNF and 2D MXene flakes. Atomic force microscopy (AFM) images revealed the 2D MXene flakes and individual CNF structures of hybrid composites (Figure 39dv). The Ti_3_C_2_T_*x*_-10% CNF-based MSCs demonstrated high flexibility (Figure 39dvi) and a capacitance retention of 86.8% after 10,000 cycles (at a current density of 0.57 mA·cm^–2^) (Figure 39dvii).

Building on the representative studies reviewed in this section, future work should aim to define robust design principles for the rational selection and engineering of lignocellulosic materials in supercapacitor applications. (1) Future efforts should prioritize the scalable implementation of surface functionalization strategies tailored for lignocellulosic systems. Approaches such as silane-mediated thiolization, phosphating, and conformal nanocoating (e.g., ALD) have demonstrated significant improvements in interfacial compatibility and electrochemical performance. However, their application remains largely limited to batch-scale or laboratory settings. (2) To ensure long-term mechanical and electrochemical durability, covalent interfacial chemistries should be explored as replacements for inherently weak hydrogen bonding or van der Waals interactions in lignocellulosic nanocomposites. Such approaches may significantly improve interfacial stability under cyclic deformation or prolonged operation. (3) Future electrode designs must carefully navigate the trade-off between electrical conductivity and ion accessibility. While densified composites promote charge transport, they often compromise porosity and hinder ion diffusion. Optimized architecture should balance matrix compaction with the preservation of hierarchical or open-cell porosity especially in thick-film electrodes aimed at achieving high areal energy density. These considerations form the basis for a next-generation design framework that moves beyond feasibility demonstrations and toward scalable, high-performance lignocellulosic supercapacitors.

#### 5.3.2 Lithium-Ion Batteries

Among several battery technologies, lithium-ion batteries (LIBs) have become indispensable rechargeable energy storage solutions for our modern lives, powering portable electronics, electric vehicles, and stationary grid storage systems.^833–835^ In this section, we explore the potential of lignocellulosic materials as a novel approach to promote the sustainable growth of LIBs. However, the broader advancement of lithium-ion battery technologies continues to be hindered by the intrinsic limitations of conventional components—particularly polymeric binders, separators, and metal-based current collectors. Conventional synthetic polymers such as polyvinylidene fluoride (PVDF) and polyethylene (PE) typically require toxic organic solvents, exhibit poor electrolyte wettability, and lack the chemical functionality or thermal resilience necessary for demanding operating conditions. These shortcomings often lead to interfacial instability, structural degradation, and limited durability, especially under high-temperature or high-loading scenarios.

Lignocellulosic materials, by contrast, offer a sustainable and multifunctional alternative. Their hierarchical fibrous architecture enables the design of porous, flexible battery components with improved ion accessibility and mechanical robustness. In addition, the abundance of surface functional groups facilitates interfacial compatibility and stable integration with conductive or protective additives, supporting the development of durable, high-performance battery systems. These materials demonstrate the ability to address a range of challenges encountered in existing LIB systems.

The research of lignocellulose approaches for LIBs is categorized in Figure 40ai. Lignocellulosic materials with hierarchical microstructures have been explored for potential utilization in electrodes, functional binders, and porous separators. Unlike conventional synthetic polymers (e.g., polyvinylidene fluoride (PVDF)), lignocellulosic materials exhibit well-defined pore structure within electrodes owing to their 1D fiber properties, facilitating unhindered Li ion migration (Figure 40aii).

**Figure 40:**
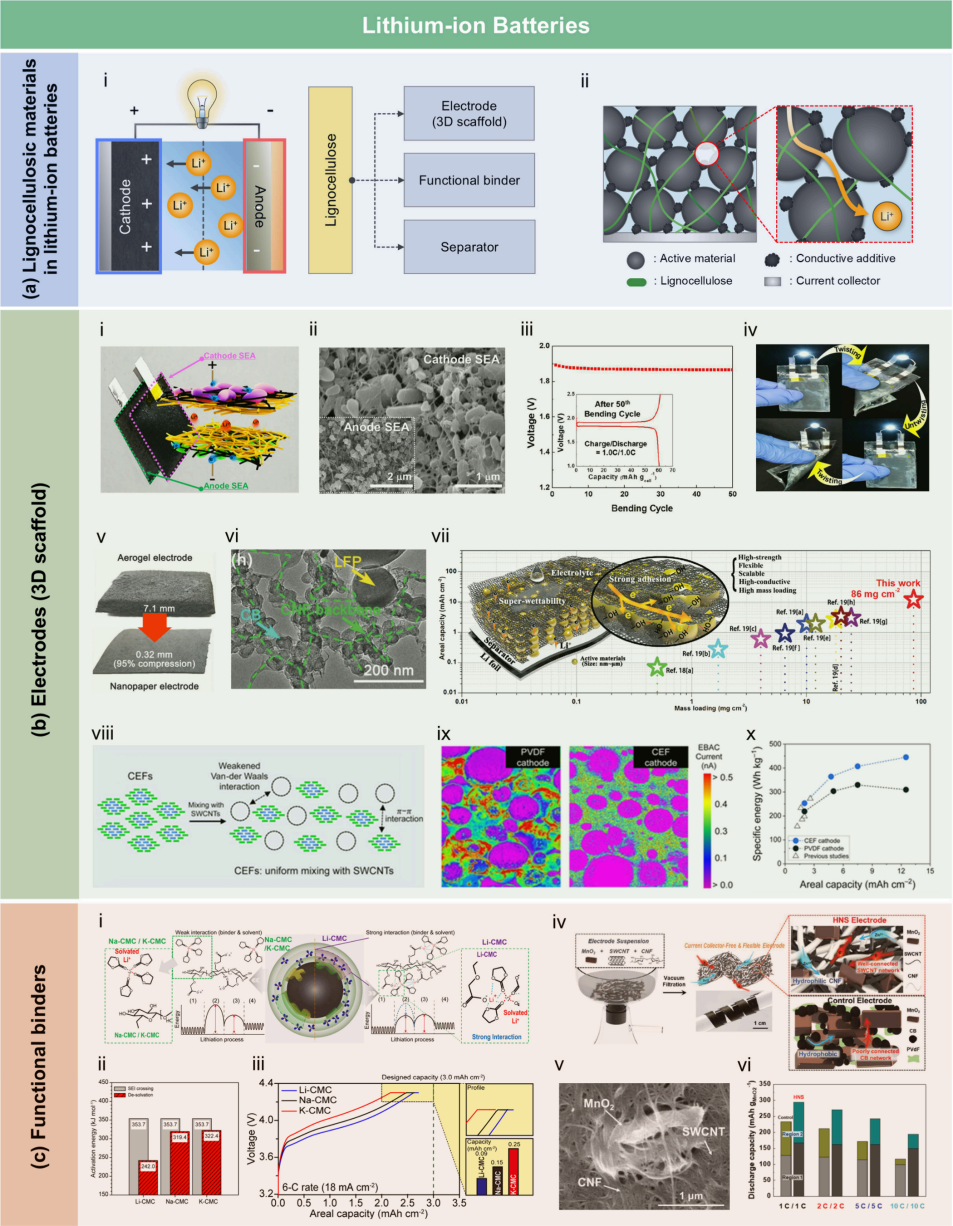
Lithium-ion batteries based on lignocellulosic materials. (a) The development of lignocellulose-based materials for electrodes, functional binders, and separators in lithium-ion batteries (i). Schematic illustrations depicting the characterization of lignocellulose-based electrode/binder describing the facile Li-ion transport pathways, and electrolyte accessibility (ii). (b) Electrodes (3D scaffold). A photograph of an h-nanomat full cell (i). Surface SEM images of flexible LFP cathode SEA (Inset image showing LTO anode SEA) (ii). Voltage changes in an h-nanomat cell over bending cycles, with an inset displaying the charge-discharge profile (at charge-discharge current density = 1.0/1.0C) after 50 cycles (iii). A photograph exhibits the electrochemical performance of an h-nanomat cell after the repeated deformation modes (iv). Adapted with permission from ref 836. Copyright 2014 American Chemical Society. A photograph of the freestanding porous LFP cathode prepared by the freeze-drying method (v). TEM image of a conductive 3D porous electrode of CB and CNF network via conformal electrostatic assembly (vi). Adapted with permission from ref 837. Copyright 2018 Wiley-VCH. Comparison of the mass loading of active materials in electrode/areal capacity: the novel aqueous 3C-binder electrode versus various LCO-based electrodes (vii). Adapted with permission from ref 838. Copyright 2021 Wiley-VCH. Schematic comparison of CNF and CEF binders in terms of their interactions with SWCNTs, emphasizing the improved dispersion achieved by CEFs (viii). EBAC images showing electron conduction pathways: PVDF-based cathodes exhibit localized conduction, while CEF-based cathodes display uniform current distribution (ix). Specific energy versus areal capacity plots comparing PVDF, CEF, and previously reported OLO-based cathodes (x). Adapted with permission from ref 839. Copyright 2025 Springer Nature under CC BY 4.0 (https://creativecommons.org/licenses/by/4.0/). (c) Functional binders. Schematic depiction of the anticipated impact of CMC modification on the surface concentration of Li^+^ ions and their intercalation (i). Activation energies of SEI crossing and desolvation of Li-, Na-, and K-CMC (ii). 6C fast charging profiles of Li-, Na-, and K-CMC cell (iii). Adapted with permission from ref 840. Copyright 2024 Wiley-VCH under CC BY 4.0 (https://creativecommons.org/licenses/by/4.0/). Schematic diagram illustrating the fabrication process of the HNS electrode and their bicontinuous ion/electron conduction pathways (iv). SEM image of the HNS electrode (v). Discharge capacity of the HNS electrode as a function of current density (vs control electrode) (vi). Adapted with permission from ref 841. Copyright 2020 Wiley-VCH.

Choi et al.^836^ introduced the heterolayer 1D-nanobuilding-element mat (h-nanomat), which comprises nanoporous CNF separator and SWCNT-based interwoven electronic conductive layers. This innovative architecture, termed the separator/electrode assembly (SEA), eliminates the need for metallic current collectors and polymeric binders (Figure 40bi). Fabricated through vacuum-assisted infiltration, SEAs yield a self-standing, porous structure of cathode or anode units, simplifying cell assembly for h-nanomat full cells (Figure 40bii). SEAs enhance shape flexibility and improve electron/ion transport, and mechanical stability. The h-nanomat cells maintain stable performance via repeated bending even after 50 cycles (Figure 40biii). Notably, the unique structure of SEAs allowed them to be repeatedly twisted and untwisted (Figure 40biv). Porous composites of lignocellulosic and conductive materials offer the potential to create scaffolds characterized by both mechanical flexibility and conductivity. Consequently, this approach enables the straightforward preparation of robust and thick battery electrodes using fibrous cellulose materials, a feat difficult to achieve with PVDF binder-based electrodes. Kuang et al.^837^ reported high-loading thick electrodes composed of 1D negatively charged CNFs network and carbon black (CB) (Figure 40bv). In the stacked electrode architecture, a mechanically interlocked conductive percolation network endures high electrode integrity and creates pores that facilitate the mass transport (Figure 40bvi). This concept can lead to tailored high-mass-loading electrodes (up to 60 mg·cm^–2^) possessing properties that are difficult to be achieved through conventional slurry-based manufacturing approaches. Wang et al.^838^ have introduced an innovative aqueous conductive binder composed of CNTs interwoven within 2D CNS. This architecture enables rapid charge distribution and maintains superior mechanical properties even in highly loaded and thick electrodes. This binder enabled the fabrication of flexible and robust LiCoO_2_ (LCO) cathodes, featuring a significant mass loading of 86 mg·cm^–2^ with an outstanding areal capacity of 12.1 mAh·cm^–2^. These results exhibited the highest-performing flexible electrodes reported to date (Figure 40bvii). In pursuit of high-energy-density lithium batteries, Hong et al.^839^ introduced cellulose elementary fibrils (CEFs) as a novel binder platform for high-mass-loading cathodes. CEFs, prepared by disassembling cellulose nanofibers through alkali and hydrotropic treatment, exhibit a deagglomerated fibrous morphology, high surface charge, and excellent dispersibility. These characteristics promote strong interactions with single-walled carbon nanotubes (SWCNTs), enabling the formation of continuous ion/electron conduction networks throughout the cathode (Figure 40bviii). Electron beam-absorbed current (EBAC) analysis further revealed that CEFs facilitated a uniform spatial distribution of electron conduction channels, in contrast to the heterogeneous and localized pathways observed with PVDF binders, owing to the improved SWCNT dispersion enabled by optimized CEF–SWCNT interactions (Figure 40bvix). When applied to overlithiated layered oxide (OLO) cathodes, CEFs ensured uniform component distribution, reduced electrode polarization, and enabled near-complete utilization of the OLO’s theoretical capacity (250 mAh·g^–1^), even at extreme areal mass loadings (up to 50 mg·cm^–2^). As a result, full cells (OLO||Li) incorporating CEF-based cathodes achieved a high specific energy density of 445.4 Wh·kg^–1^, significantly outperforming the PVDF-based control cells (Figure 40bx). Moreover, the anionic nature of CEFs enabled the chelation of transition metal ions, suppressing their dissolution and stabilizing both the cathode and lithium metal anode during prolonged cycling. This strategy demonstrates the multifunctionality of CEFs not only as structural binders but also as key enablers for scalable, high-performance electrode architectures.

Byun et al.^840^ presented a novel approach to accelerate the desolvation process by regulating the interaction between the cellulose-based binder and the solvent-Li^+^ complexes. They synthesized CMC derivatives substituted with three alkali metal ions (Li^+^, Na^+^, and K^+^), to investigate the influence of these metal ions on their performance (Figure 40ci). Via MD simulations, they observed a higher Li^+^ concentration at the interface between graphite and the binder for Li-CMC compared to Na- and K-CMC, suggesting an enhanced interaction between Li-CMC and Ethylene carbonate. Hence, Li-CMC exhibited the lowest activation energy for desolvation and the highest chemical diffusion coefficient (Figure 40cii). Significantly, a Li-CMC-based cell exhibited the longest time to reach a voltage of 4.3 V, marking the onset of charging in the CV mode. This observation suggests that the presence of Li^+^ within the binder facilitates the efficient intercalation of Li^+^ into the graphite anode (Figure 40ciii).

#### 5.3.3 Zinc-Ion Batteries

Biofriendly and nontoxic aqueous zinc (Zn)-ion batteries (ZIBs) are potential alternatives to LIBs.^842,843^ However, they have been hampered by low energy density, sluggish redox kinetics, and limited cyclability at high rates. These limitations arise from the constraints of the redox reaction at the electrode. Kim et al.^841^ designed an innovative Zn-ion electrode architecture incorporating 1D CNFs and SWCNTs to address these issues. They developed a manganese oxide (MnO_2_)-based heterofibrous network scaffold (HNS) cathode with electron and ion conduction channels through a facile vacuum-filtration process (Figure 40civ). The HNS exhibited excellent electrolyte uptake owing to its uniform bicontinuous structure and hydrophilic CNFs, which promoted the redox kinetics of MnO_2_ (Figure 40cv). As a result, the Zn-MnO_2_ full cell demonstrated superior charge-discharge rate performance compared to the control PVDF/CB binder-based electrode (Figure 40cvi). Furthermore, the well-dispersed SWCNT-based conductive network, without the need for a heavy SUS foil current collector, enabled the HNS electrode to achieve outstanding energy and power density.

#### 5.3.4 Post-Lithium-Ion Batteries

Unlike the SCs and LIBs described above, the application of lignocellulosic materials in newly emerging energy storage systems such as lithium–sulfur batteries (LSBs),^844,845^ lithium metal batteries (LMBs),^846–848^ and metal–air batteries (MABs)^849,850^ has rarely been reported. Li–S batteries are noted for their promising attributes, including high energy density, cost-effectiveness, and environmental benignity.^851^ However, their practical deployment faces significant impediments, mainly due to poor electrical conductivity and the Li polysulfide (LiPS) shuttle effect. In this context, lignocellulose is an ideal material candidate for Li–S batteries owing to its ability to anchor the active/conductive materials and suppress the shuttle effect of LiPS.^852^ Recently, Chen et al.^844^ reported a lignin-derived ultralow dosage binder (AL-Lys-D) for sulfur cathodes in Li–S batteries. Through electrophilic substitution and demethylation, amino acid fragments were introduced to enhance the adsorption capacity of alkali lignin for LiPSs (Figure 41ai). As a result, AL-Lys-D binders effectively mitigated the shuttle effect and increased the utilization rate of sulfur, leading to significant improvements in battery performance. Despite the binder’s low dosage of 2 wt %, Li–S batteries demonstrated an initial capacity of 864 mAh·g^–1^ at 0.5 C and maintained a capacity of 413 mAh·g^–1^ after 1000 cycles. In contrast to functionalized lignin-based binders such as AL-Lys-D, which rely on chemical modification to introduce LiPS-adsorbing groups, Li et al.^845^ developed a lignocellulosic pulp-based binder (LCNF) that leverages intrinsic material properties without requiring extensive purification or functionalization (Figure 41aii). Derived from bamboo via maleic anhydride pretreatment and nanofibrillation, the resulting amphiphilic network, which was composed of semirigid lignin and rigid cellulose nanofibrils, offered abundant LiPS-binding sites (e.g., –OH, –COOH) and maintained electrode integrity through a stable 3D hydrogen-bonded framework. Unlike molecularly dispersed systems, the loosely packed nanostructure of LCNF facilitated fast Li^+^ transport, enhancing electrochemical kinetics. These structural and chemical features enabled the fabrication of high-sulfur-loading cathodes (13.36 mg·cm^–2^) with an initial capacity of 996 mAh·g^–1^ and excellent long-term stability. Furthermore, an Ah-level pouch cell employing the LCNF binder achieved energy densities of 322 Wh·kg^–1^ and 432 Wh·L^–1^, underscoring its scalability for practical Li–S batteries. The tremendous need for electric vehicles and grid-scale energy storage has led to a focus on high-energy-density rechargeable batteries. Particularly, LMBs have garnered significant interest due to their promising ability to attain higher specific energy levels than conventional LIBs.^853^ However, the practical application of LMBs is impeded by obstacles, including uncontrolled dendritic lithium metal growth and electrolyte interface instability. In response to these challenges, various approaches have been adopted, such as the formation of artificial solid electrolyte interphase (SEI) layers and the implementation of solid-state electrolytes. Chen et al.^846^ introduced the CA-based thin layers on a Li metal surface as a beyond-synthetic material strategy for sustainable Li metal batteries. The C=O in the ester groups enhances charge transfer by accelerating the decomposition of lithium bis­(trifluoromethanesulfonyl)­imide (LiTFSI) salt to form a LiF-rich SEI, facilitating uniform and fast Li^+^ transfer. They examined the morphological changes and electrochemical performance of lithium electrodeposited on three types of electrodes: bare Cu, Cu covered with CNF (CNF-Cu), and Cu covered with CA (CA-Cu). Notably, lithium deposited on CA-Cu displayed smooth morphologies without dendrites, as illustrated in Figure 41bi. Consequently, the CA-coated Li anode with ex-situ doped LiTFSI exhibited an extended cycle performance, suppressed dendrite formation during Li deposition, and facilitated efficient Li stripping. Inspired by natural mechanisms, researchers engineered a nanowood solid electrolyte interphase to mimic pit membrane structure, aiming to regulate Li-ion flow (Figure 41bii).^847^ The nanowood SEI is a thin film structure with a thickness of 10 nm and a specific sheet size of 1 mm^2^. Derived from the interlamellar matrix of a partially removed cell wall, the nanowood SEI comprises parallel ultrafine cellulose fibrils measuring 3–5 nm. This nanowood SEI effectively modulates Li-ion concentration along the Li-metal surface, facilitating uniform distribution and deposition of Li ions and preventing dendritic Li growth (Figure 41biii).

**Figure 41:**
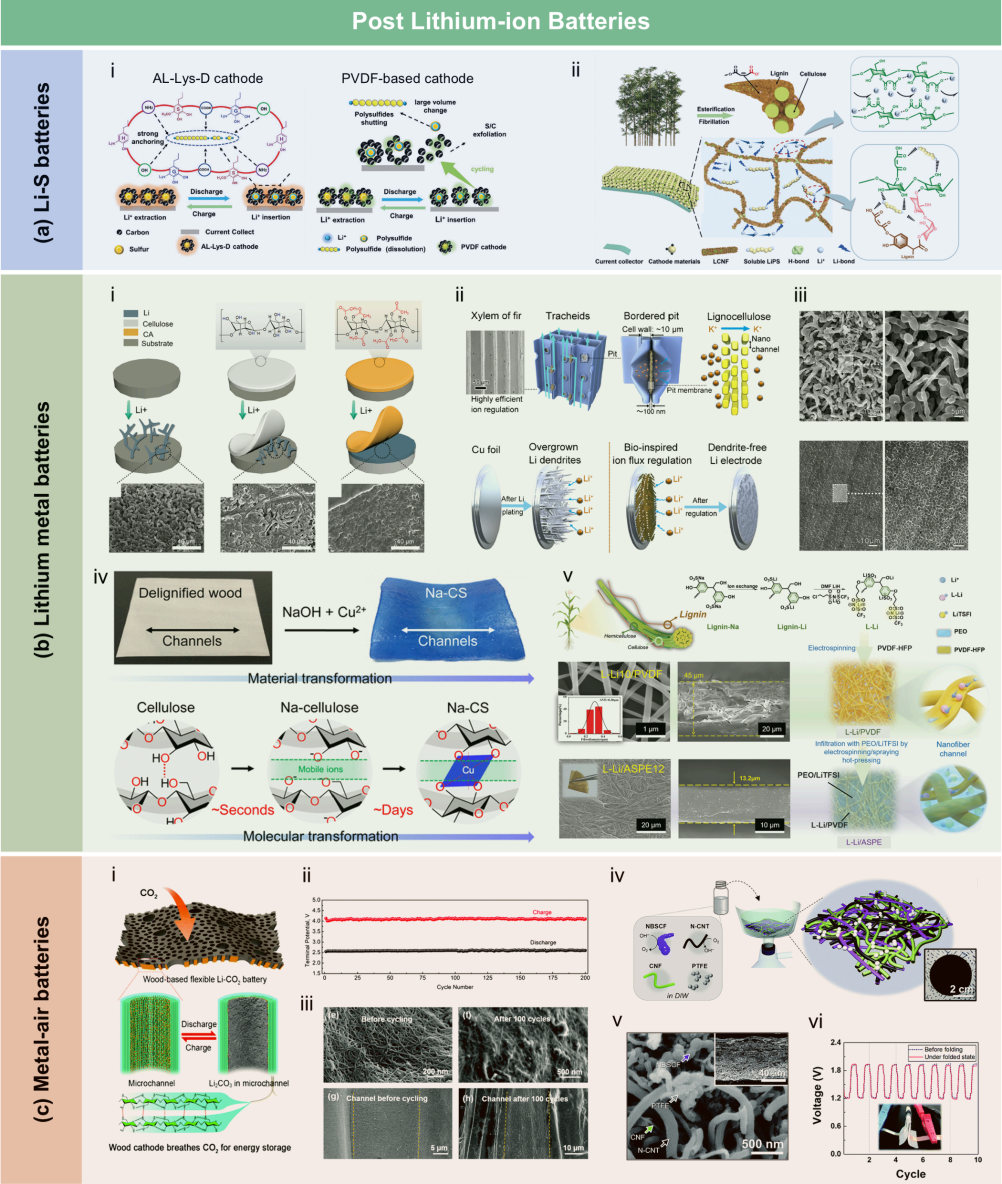
Postlithium-ion batteries based on lignocellulosic materials. (a) Li–S batteries. A schematic illustration of binding role of AL-Lys-D in sulfur cathode in Li–S battery (i). Lignin-based AL-Lys-D binder can effectively maintain the electrode stable and anchor polysulfides during the electrochemical process. Adapted with permission from ref 844. Copyright 2023 Wiley-VCH GmbH. Schematic of the LCNF binder in Li–S cathodes, illustrating its 3D fibrous network for structural stability, rapid Li^+^ transport through loosely packed domains, and multisite interactions with LiPS for shuttle suppression (ii). Adapted with permission from ref 845. Copyright 2025 Wiley-VCH GmbH. (b) Lithium metal batteries. Deposition process for the bare Cu, cellulose-Cu, and CA-Cu electrodes, followed by SEM imaging of metallic Li deposits at 4 mA·h·cm^–2^ (i). Adapted with permission from ref 846. Copyright 2021 Wiley-VCH GmbH. A conceptual illustration of the pit-membrane design and the construction of the wood-structure-based artificial SEI for Li-metal anodes (ii). SEM images of bare Cu foil and Cu substrate treated with pit-membrane-like nanowood artificial SEI after Li plating (iii). Adapted with permission from ref 847. Copyright 2021 American Chemical Society. Schematic and optical illustration of delignified wood transforming into Na-CS via Na^+^ complexation-induced chain opening and Cu^2+^ coordination, showing progressive color change and molecular ordering (iv). Adapted with permission from ref 848. Copyright 2023 American Association for the Advancement of Science (AAAS) under CC BY 4.0 (https://creativecommons.org/licenses/by/4.0/). Schematic illustration of the synthesis and integration of lignin-derived single-ion conductive networks (L-Li), including the fabrication of L-Li/PVDF and L-Li/ASPE membranes and their internal nanofiber architectures, along with SEM images showing the surface and cross-sectional morphologies of each structure (v). Adapted with permission from ref 854. Copyright 2024 Wiley-VCH GmbH. (c) Metal–air batteries. The scheme illustrates the Li-CO_2_ battery design using a flexible wood cathode that captures CO_2_ for energy storage (i). Terminal voltage of charge and discharge profiles (ii). SEM images of the morphological change of the cathode surface (top) and microchannels (bottom) after cycling (iii). Adapted with permission from ref 849. Copyright 2018 Royal Society of Chemistry. Schematic and photograph depicting the fabrication process of the MH paper air cathode (iv). Cross-sectional SEM image displaying the entangled network structure of the 1D cathode components (v). Galvanostatic charge-discharge cycle profiles before and after folding (vi). Adapted with permission from ref 850. Copyright 2019 Royal Society of Chemistry.

Further advancing the field, Yang et al.^647^ have reported solid polymer ion conductors by designing molecular channels with nanocellulose. By coordinating copper ions with 1D CNFs, they expanded the channels, thereby enhancing Li^+^ conductivity to 1.5 × 10^–3^ S·cm^–1^ at room temperature without liquid electrolyte. This cellulose-based ion conductor improved ionic conductivity and enhanced the structural stability of the electrolyte. Dong et al.^848^ developed Na^+^-containing cellulose supramolecular (Na-CS) solid electrolytes by molecularly engineering delignified wood into ordered sodium-coordinated channels (Figure 41biv). Through precise control of intermolecular hydrogen bonding and Na^+^ coordination, they achieved highly aligned ionic pathways that deliver fast Na^+^ conduction together with robust mechanical integrity, providing a sustainable route for solid-state sodium batteries. Lignin, a highly abundant aromatic biopolymer, offers exceptional thermal stability, structural rigidity, and a rich variety of functional groups (e.g., hydroxyl, methoxy, and carboxyl), making it an attractive precursor for solid polymer electrolytes (SPEs). Liu et al.^854^ reported a lignin-derived ultrathin SPE designed for all-solid-state lithium batteries, in which lignin serves not only as a structural scaffold but also as a chemically modifiable backbone for ion-conducting functionality. By sulfonating the lignin and incorporating it into a polyacrylonitrile (PAN) matrix, the authors fabricated a 3D nanofiber network that was subsequently grafted with LiTFSI-like groups to enable single-ion conduction (Figure 41bv). The resulting composite, only ∼10 μm thick, exhibited excellent mechanical integrity, flame retardancy, and flexibility, traits inherently linked to lignin’s rigid yet functionalizable structure. Critically, the covalently anchored anionic groups suppressed anion mobility and enabled a high lithium-ion transference number (∼0.91), overcoming the limitations of traditional dual-ion systems. The continuous 3D nanofiber architecture, formed via electrospinning of the lignin-PAN precursor, enabled fast and selective Li^+^ transport with ionic conductivities up to 2.1 × 10^–4^ S·cm^–1^ at 60 °C. This study highlights the dual role of lignin in SPEs, its aromatic backbone enhances mechanical and thermal resilience, while its chemically tunable functional groups enable the design of efficient single-ion conducting pathways. These findings underscore the potential of molecularly engineered lignin as a scalable and sustainable platform for next-generation solid-state lithium batteries.

Metal-air rechargeable batteries are proposed as promising next-generation power sources because their configuration utilizes limitless oxygen at air electrodes, leading to a high theoretical energy density.^855^ Xu et al.^849^ reported a mechanically flexible Li–CO_2_ battery employing a wood-based cathode structure, benefiting from the inherent microchannels and nanochannels in wood-CNFs that facilitate CO_2_ and electrolyte transport (Figure 41ci) The cathode incorporates a ruthenium (Ru)-decorated CNT network within the microchannels, offering large surface area for discharge product deposition. Utilizing this flexible wood-based cathode with a thickness of 50 μm, Li–CO_2_ batteries exhibit a constant overpotential at 1.5 V over 200 cycles with a specific capacity of 1000 mA·h·g_c_^–1^ (Figure 41cii). After cycling, the architecture and surface morphology of the cathode remain unchanged, preserving their pristine structure throughout the testing period (Figure 41ciii). Lee et al.^850^ introduced a monolithic heteronanomat (MH) air cathode as a novel approach, enabling stable electrochemical recharge capability in origami-folded Zn–air batteries. The MH paper air cathodes were constructed via vacuum filtration of the cathode suspension, resulting in the highly entangled network structure of the MH paper air cathode (Figure 41civ). The 3D hierarchical network structure of the MH paper air cathode facilitates the creation of a porous electrode structure, which is beneficial for accommodating aqueous electrolyte and air (Figure 41cv). The resulting Zn–air battery demonstrated versatility in form factors suitable for diverse flexible applications. It maintained stable charge-discharge voltage profiles at a current density of 1.0 mA·cm^–2^ even under fully folded conditions, as depicted in Figure 41cvi, with minimal deviation from the unfolded Zn–air battery profiles.

#### 5.3.5 Separators

The separator, technically called porous film, is a critical component in ensuring both the safety and performance of batteries. It serves as a barrier to prevent direct electrical contact between the cathode and anode while providing ion channels. For the high energy density post LIBs, an ideal separator must meet several requirements: (1) possessing well-developed porous structure for facile ion transport, and (2) exhibiting high mechanical/thermal stability to withstand the dendritic growth of the metal anode including zinc, lithium and sodium. Lignocellulose-based materials (e.g., CNFs, CNCs, and lignocellulose) have emerged as a promising alternative to conventional petroleum-based synthesized materials due to their high mechanical/thermal stability, excellent electrolyte wettability, efficient functionalization and eco-friendliness. Lee et al.^856^ pioneered a CNF-based separator with highly interconnected nanoporous structure by introducing isopropyl alcohol (IPA) and silica nanoparticles (SiO_2_) as disassembling agents. This structure and the intrinsic property of CNF allowed the CNF-based separator to have good electrolyte wettability, leading to high performance in LIBs (Figure 42ai). Owing to the abundant hydroxyl groups on nanocellulose, a modified CNF-based separator was proposed. Hydroxyapatite (HAP), a natural inorganic mineral (Ca_10_(PO_4_)_6_(OH)_2_), has high mechanical stability and excellent thermal stability with a melting point of 1,650 °C. By forming hydrogen bonds between the HAP and the CNF, the composite HAP/CNF hybrid separator was formed which has three-dimensional porous structure (Figure 42aii). This separator exhibited excellent thermal stability of up to 250 °C and outstanding flame retardancy (Figure 42aiii).^857^ Recently, a biodegradable lignocellulose nanofiber (LCNF) separator has been developed for aqueous zinc-ion batteries (AZIBs). The LCNF separator offers a hydrophobic surface with uniform nanopores (∼20 nm), high mechanical strength (47.8 MPa), and excellent ionic conductivity (18.1 mS cm^–1^). These features enhance zinc ion transport and deposition, significantly reducing zinc dendrite formation and improving cycling stability. In practical applications, this separator enables Zn||Mg_*x*_V_2_O_5_ (MgVO) full cell to achieve high-capacity retention over 2,000 cycles. In addition, its biodegradability ensures low environmental impact, making it a sustainable and high-performance solution for zinc-ion batteries (Figure 42aiv).^858^ In another study, nanoporous terpyridine (TPY)-functionalized CNF mat was added to the electrospun polyvinylpyrrolidone (PVP)/polyacrylonitrile (PAN) mat as a functional support layer (Figure 42av).^859^ The TPY molecules were conjugated with the oxidized CNF to chelate unwanted byproducts (dissolved heavy metal ions from the cathode) in the electrolyte. The TPY-CNF separator with well-designed chemical functionality enabled a significant improvement in the high temperature (e.g., 60 °C) cycling performance of LiMnO_2_ (LMO)-cathode-containing cells (Figure 42av, bottom right). However, these approaches are limited by the fabrication method of vacuum filtration, which is difficult to apply to large-scale batteries. To overcome this limitation, Park et al.^860^ developed an entire process to produce bacterial cellulose nanofiber (BCNF) membranes. They optimized the strain for higher yield and used a series of 30-L fermenters for large-scale production. The BCNF slurry containing polycarbonate (PC) as a pore-forming agent was coated on the PET film and was detached as a self-standing film (Figure 42bi). This BCNF-based separator exhibited remarkable cycle stability with 80% capacity retention even after 1,000 cycles in LiNi_0.6_Co_0.2_Mn_0.2_O_2_ (NCM622)/LiNi_0.85_Co_0.10_Al_0.05_O_2_ (NCA) (with a mixed ratio of 8:2) mixed cathode full cell. In addition, Yang et al.^861^ reported a novel nanoporous CNF-based separator utilizing the nonsolvent-induced phase separation (NIPS) method. This separator, called RC separator (RCS), exhibited a well-developed porous structure (porosity of 51–61%), and good electrolyte wettability (436% uptake), resulting in excellent performance in LIBs (Figure 42bii). However, a natural wood-based separator was proposed,^862^ which possesses extremely excellent mechanical properties and thermal stability. The modified natural wood films, achieved through a hydrothermal treatment, exhibited high tensile strength (58 MPa), excellent thermal stability, and high ionic conductivity (0.48 mS·cm^–1^). These separators provided efficient ion transport channels and improved cycling stability in lithium-ion batteries, outperforming commercial PP separators in both performance and environmental impact (Figure 42ci). CNC-based separator which has high mechanical strength and a novel chiral nematic porous structure, was also proposed. This unique chiral nematic structure enhances uniform Li-ion flux through its cholesteric channels, significantly reducing the formation for dendritic lithium and improving the overall cycling stability of lithium–metal batteries. The CNC-based separators demonstrated excellent ionic conductivity and mechanical robustness, making them a promising alternative to conventional separators in high-performance energy storage systems (Figure 42cii).^863^

**Figure 42:**
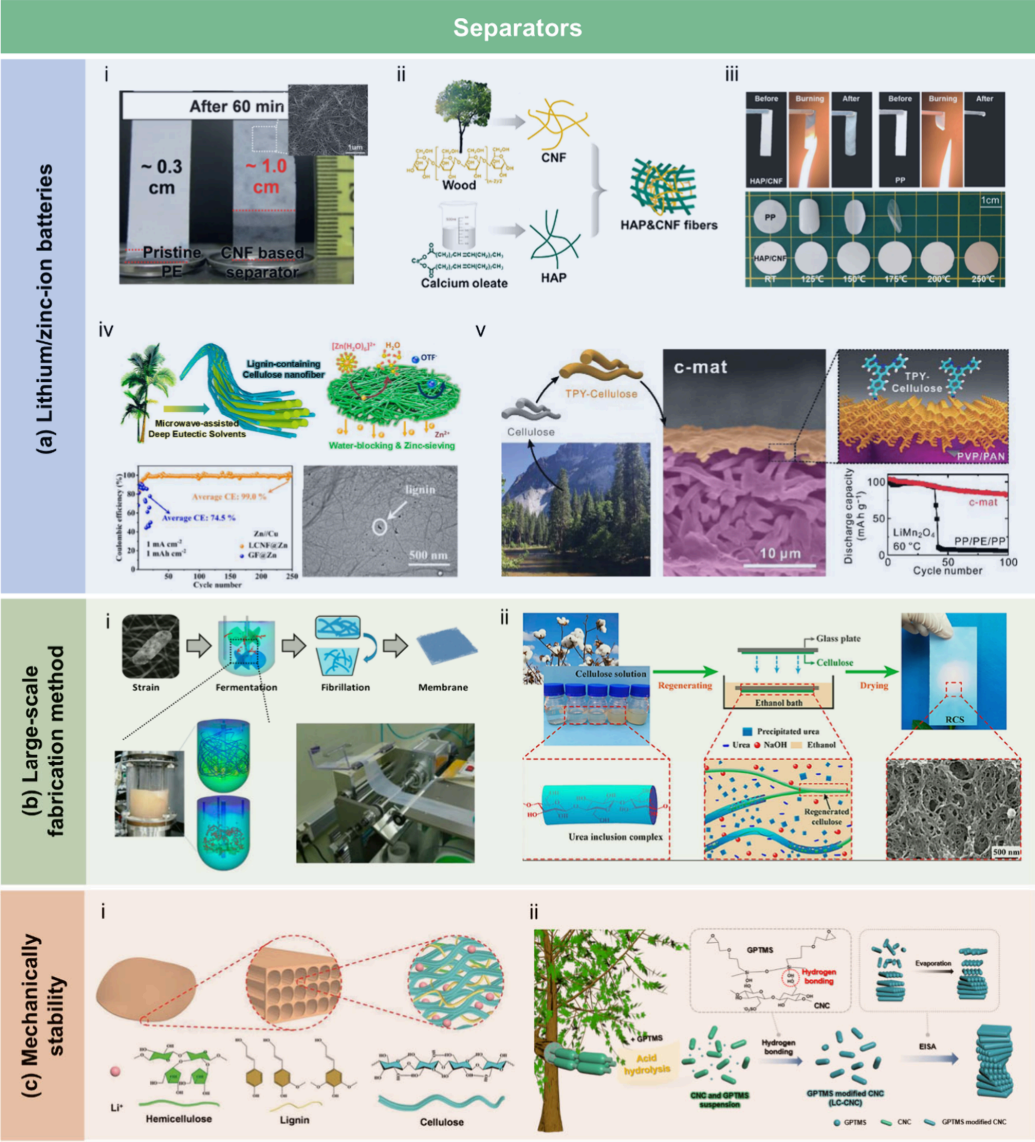
Lignocellulosic-based separators. (a) Lithium/zinc-ion batteries. A comparison of liquid electrolyte wettability between PP/PE/PP separator and CNF separator. SEM image of CNF separator (inset) (i). Adapted with permission from ref 856. Copyright 2012 American Chemical Society. Schematic illustration of HAP/CNF separators in LIBs (ii). Flame retardancy and thermal shrinkage (area-based dimensional change) of PP and HAP/CNF separator (iii). Adapted with permission from ref 857. Copyright 2023 American Chemical Society. The schematic diagram of the fabrication process of the LCNF membrane and the CE of the zinc plating/stripping behavior in the Cu//Zn batteries with LCNF and GF separators under 1 mA·cm^–2^ with the capacity of 1 mA·h·cm^–2^ (iv). Adapted with permission from ref 858. Copyright 2023 Elsevier. A schematic illustration of functionalized CNF-integrated nanomat separator (c-mat) that consists of TPY-CNF thin layer and electrospun PVP/PAN support layer (v). Adapted with permission from ref 859. Copyright 2016 American Chemical Society. (b) Large-scale fabrication method. Overall scheme for the construction of bacterial CNF membrane for battery separators and its application (i). Adapted with permission from ref 860. Copyright 2019 National Academy of Sciences. Schematic illustration of preparation of regenerated cellulose separator (RCS) and photo image and SEM image of actual RCS film (ii). Adapted with permission from ref 861. Copyright 2021, American Chemical Society. (c) Mechanically stability. Illustration of the chemicals and structure of wood film (i). Adapted with permission from ref 862. Copyright 2022 Royal Society of Chemistry under CC BY-NC 3.0 (https://creativecommons.org/licenses/by-nc/3.0/). Schematic illustration depicting the fabrication process of liquid crystalline-cellulose nanocrystal (LC-CNC) and its chemical structure (ii). Adapted with permission from ref 863. Copyright 2022 Elsevier.

In summary, lignocellulosic materials have demonstrated broad potential as multifunctional components in both lithium-ion and postlithium-ion battery systems, including roles as binders, separators, and structural frameworks. While many studies have highlighted their physicochemical advantages and tunable functionality, the majority of these findings remain at the proof-of-concept stage. To realize their potential in practical systems, future research must now focus on overcoming key limitations related to processing, structural uniformity, and chemical stability through scalable and integrated design strategies. (1) Although lignocellulosic materials offer intrinsic advantages such as hierarchical porosity and abundant surface functionalities, their practical implementation remains constrained by processing limitations. Most demonstrations to date have relied on lab-scale techniques (e.g., freeze-drying, vacuum filtration) that are incompatible with industrial-scale manufacturing. Overcoming this will require new fabrication strategies that preserve structural integrity while supporting continuous, high-throughput production. (2) Scalable application further demands systematic engineering to achieve uniform fibrillation, structural reproducibility, and reliable large-area dispersion, which are prerequisites for hybrid integration with nanocarbon materials and for constructing robust, ion- and electron-conductive architectures. (3) Finally, mitigating degradation in advanced batteries will require more robust stabilization strategies. Rather than depending solely on the weak hydrogen bonding inherent to lignocellulose, future systems should explore covalent bonding, ionic coordination, or chelation to suppress parasitic reactions and enhance component stability, especially in chemistries susceptible to metal-ion dissolution or dendritic growth. By addressing these interconnected challenges, lignocellulosic materials can transition from laboratory-scale feasibility to high-performance, scalable components in next-generation battery technologies.

### 5.4 Energy Harvesting

#### 5.4.1 Solar Cells

In the context of global climate change and the ongoing energy crisis, solar cells (SC), which convert sunlight into electricity, play a crucial role in advancing the strategic goal of sustainable development by utilizing clean and renewable solar energy.^864–866^ To mitigate the substantial material consumption and high production costs associated with commercial crystalline silicon SC, it is crucial to explore green and cost-effective alternative materials.^867,868^ Such alternatives aim to reduce dependence on traditional SC substrates and lower production costs of traditional SC substrates, making photovoltaic technology more economical and environmentally sustainable. Lignocellulose materials, widely recognized for their use as flexible substrates, offer several advantages, including lightweight, low cost, recyclability, and biodegradability.^369,869–871^ These characteristics position lignocellulose as a promising candidate to address resource and cost challenges in SC technology. Efficient solar energy conversion, however, requires effective thermal management and heat dissipation, both of which are essential to ensuring the performance and longevity of SC.^872,873^ Pure lignocellulose films, however, exhibit from poor thermal conductivity and instability during the heating process, potentially causing localized overheating in electronic devices. To overcome these limitations, researchers have undertaken numerous studies to enhance thermal conductivity and thermal management efficiency of lignocellulose-based materials.^874^ These efforts aim to optimize lignocellulose to better meet the thermal demands of SC, thus improving their overall efficiency and durability.

##### 5.4.1.1 Cellulose-Based Films for SC

Through chemical modification and the incorporation of functional materials, lignocellulose-based biofilms can achieve excellent thermal conductivity and mechanical strength, making them suitable for use in SC collectors (Figure 43ai).^875^ Cellulose films have garnered significant attention in the field of SC photothermal management due to their superior high transparency, high haze, and robust mechanical properties, as well as their ease of modification and compatibility with other materials.^876^ For instance, Wang et al. developed a nanocellulose/epoxy composite substrate using chemically modified cellulose nanofibers (Figure 43aii).^877^ This composite substrate demonstrated a reduction in the thermal expansion coefficient by 19 ppm·K^–1^ and an increase in the glass transition temperature by 71.8 °C. Additionally, it exhibited a high transmittance of 89% at 600 nm. The conductive layer deposited on this substrate maintained stable conductivity of approximately 835 S·cm^–1^ even after environmental thermal cycles. Similarly, Shirazi proposed an all-biobased cellulose film characterized by high anisotropic light scattering and superhydrophobicity (Figure 43aiii).^878^ The prepared film had a transmittance of approximately 94% and a haze of about 54% at a wavelength of 550 nm. When used as a light management layer, it enhanced the power conversion efficiency of perovskite SC by 6%. The film’s surface water repellency also imparted self-cleaning capabilities, ensuring the stability of incident light. In another study, Wu et al. developed CNP for organic SC, which improved efficiency and effective wide-angle light capture, achieving a maximum power conversion efficiency of 16.17%.^875^ This is among the highest efficiencies reported for natural materials used in organic SC. Cellulose film materials in SC can also provide functional characteristics such as energy level alignment, high mechanical strength, and recyclability (Figure 43aiv).^879,880^ Moreover, SC constructed from cellulose can be disassembled, and their main components can be separated and recycled through a low-energy consumption process at room temperature, demonstrating the high recyclability of SC.^14^ These examples illustrate that cellulose-based films can deliver meaningful optical and thermal functions. However, when compared to conventional glass or polymer substrates with well-established durability and cost structures, it remains uncertain whether the performance gains outweigh the complexities of chemical modification, potential processing challenges, and long-term stability under real-world conditions. Systematic comparative data, including lifetime testing under outdoor conditions and cost-per-area analyses, are needed to assess whether such cellulose composites can compete at scale or are better suited for niche applications, such as biodegradable or disposable devices.

**Figure 43:**
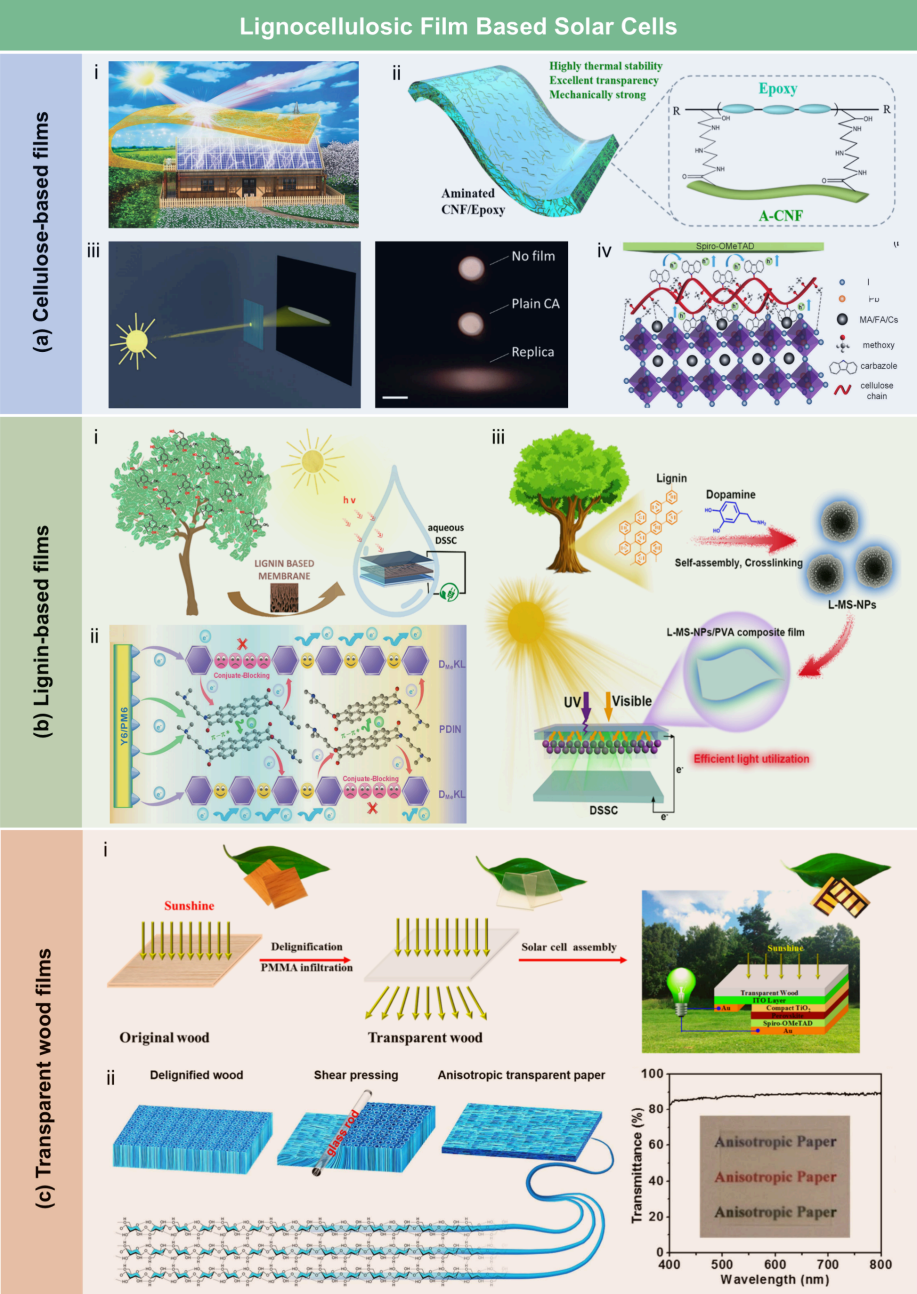
Application of LCFs in solar cells. (a) Organic solar cells based on CNP (i), hybrid cellulose-nanofiber/polymer substrates for the electrodes of flexible solar cells (ii), schematic of laser beam interference with the film and anisotropic scattering of light (iii), and schematic illustration of the interaction between 6-*O*-[4-(9*H*-carbazol-9-yl)­butyl]-2,3-di-*O*-methyl cellulose and perovskite (iv). Adapted with permission from refs 875,877–879 . Copyright 2019 Royal Society of Chemistry; Copyright 2020 American Chemical Society; Copyright 2024 Springer Nature under CC BY 4.0 (https://creativecommons.org/licenses/by/4.0/); Copyright 2023 Wiley-VCH under CC BY 4.0 (https://creativecommons.org/licenses/by/4.0/). (b) Lignin-based polymer electrolyte membranes for sustainable aqueous dye-sensitized solar cells (i), electron transfer 3D network fabricated by D_Me_KL/PDIN/Y6 (ii), and schematic showing preparation of lignin-derived multifunctional dopant and application of the lignin-derived multifunctional dopant/poly­(vinyl alcohol) composite film for solar cells (iii). Adapted with permission from refs 881–883 . Copyright 2021 American Chemical Society; Copyright 2020 Wiley-VCH; Copyright 2021 American Chemical Society. (c) Schematic sketch showing the process of transparent wood preparation and assembling of a solar cell on the transparent wood substrate (i), and graphical illustration of the design concept and “top-down” fabrication process for the anisotropic transparent paper directly from natural wood (ii). Adapted with permission from refs 356,884 . Copyright 2017 Elsevier; Copyright 2019 American Chemical Society.

##### 5.4.1.2 Lignin-Based Films for SC

Lignin has emerged as a promising active material in SC as well.^885^ Due to its high phenolic content, water-soluble lignin sulfonates, when mixed with poly­(3,4-ethylenedioxythiophene), exhibit high hole mobility, making them potential polymer semiconductors for SC applications.^867,886^ In the development of lignin-based solid electrolytes, de Haro et al. synthesized a novel bioderived polymer membrane by reacting preoxidized sulfate lignin with PE glycol diglycidyl ether (Figure 43bi).^881^ This membrane, incorporated as a quasi-solid electrolyte framework in water-based dye-sensitized SC, resulted in devices with excellent long-term stability under UV–visible light. In the realm of lignocellulose cathode interface layers, Hu et al. introduced a new cathode interface layer composed of demethylated sulfate lignin and amino-terminated substituted perylene diimide (Figure 43bii).^882^ This innovative approach yielded a nonfullerene acceptor organic SC with a short-circuit current density of 26.61 mA·cm^–2^ and a power conversion efficiency of 16.02%, surpassing traditional nonfullerene acceptor organic SC and achieving the highest efficiency among biomaterial intermediate layers. Similarly, Zhang et al. prepared a binary cathode interface layer for organic SC using industrial solvent fractionation of lignin.^886^ Their results demonstrated that this binary interface layer exhibited comparable or even superior efficiency compared to conventional binary cathode interface layers in existing organic SC. Moreover, selective removal of chromophores in lignin can produce lignin nanofilms with high optical transmittance, thermal stability, and ultraviolet absorption capabilities.^867^ For instance, Wang et al. leveraged the fluorescence and self-assembly properties of lignin to develop a lignin-derived multifunctional dopant (Figure 43biii).^883^ This dopant, when combined with PVA, was used to fabricate an optical composite film with haze, fluorescence, and room temperature phosphorescence properties, enhancing the power conversion efficiency of dye-sensitized SC to 4.1%. By integrating lignin into various components of SC, researchers are paving the way for more environmentally friendly and cost-effective photovoltaic technologies.

These results demonstrate lignin’s multifunctionality, yet a critical assessment is needed. Given the general heterogeneity of lignin, it is important to understand how these lignin-based layers fare in terms of long-term chemical stability, reproducibility of supply, and cost versus synthetic alternatives. Without clear cost-benefit analyses or accelerated aging studies, it remains unclear whether lignin components can move beyond laboratory-scale promise to reliable, scalable SC production.

##### 5.4.1.3 Lignocellulose-Based Films for SC

Due to the low DP of hemicellulose, its composite materials exhibit insufficient mechanical strength compared to those made from cellulose and lignin. Furthermore, hemicellulose demonstrates poor heat resistance and oxidation resistance, making it prone to degradation under light and thermal conditions, thereby limiting its service performance.^887^ Consequently, there are few reports on the use of films prepared primarily from hemicellulose for SC. On the other hand, the inherent complexity of lignocellulose raw materials often complicates the precise control of the target properties of composite films made from multiple components. This complexity affects critical attributes like thermal conductivity and optical properties, thereby limiting the application of such composite films in SC. Despite these challenges, some research has explored the potential of lignocellulose-based materials in SC applications. For instance, Premkumar et al. utilized lignocellulose extracted from pineapple crown leaves combined with TiO_2_ to prepare photoanodes for dye-sensitized SC.^643^ The JV characterization revealed an efficiency of 1.0034%, with a fill factor of 0.40644, an open-circuit voltage of 0.7058 V, and a short-circuit current density of 3.4906 mA·cm^–2^. In the investigation using composite biomass materials like wood for the fabrication of solar cell substrates, Li et al. accomplished a breakthrough by effectively incorporating perovskite solar cells processed at low temperatures (<150 °C) onto transparent wood film substrates. This achievement led to an impressive solar cell power conversion efficiency of up to 16.8% (Figure 43ci).^884^ Similarly, Jia and colleagues have introduced a novel anisotropic wood film material characterized by exceptional mechanical flexibility and outstanding optical attributes. This material boasts high transmittance (∼90%) and high haze (∼90%), making it an ideal light management coating for GaAs solar cells. The application of this innovative material has resulted in a significant efficiency enhancement of 14% (Figure 43cii).^356^ These advances show that lignocellulose-based substrates can, in specific configurations, approach competitive efficiencies. Yet, there are some broader questions about what the manufacturing costs, yield consistency, and lifetime under real-world exposure for such substrates are. Additionally, the complexity of lignocellulose composites complicates precise tuning of thermal conductivity, optical properties, and moisture resistance, which are critical for device reliability. More comprehensive benchmarking, such as comparing transparent wood perovskite modules versus glass-based modules in cost-per-watt, stability under outdoor cycling, and end-of-life recycling, would clarify whether these innovations represent viable alternatives or primarily intriguing demonstrations.

Through physical and chemical modification and functionalization, lignocellulose films can exhibit enhanced electrical conductivity, photoelectric conversion capacity, and some other desirable properties for use in SC. Table 9 benchmarks the performance of SC on LCF-based substrates against conventional materials (PET and glass). However, the purification and modification processes for cellulose and lignin are relatively complex, consuming substantial resources and energy, which contribute to increased production costs. To advance LCFs toward competitiveness, several strategies merit emphasis. First, systematic benchmarking must be integrated into research. Further studies should include parallel tests against conventional substrates or layers, reporting metrics such as cost-per-area, device lifetime under accelerated aging (such as thermal cycling, UV exposure, humidity), and environmental impact assessments. Second, design efforts should prioritize properties most crucial for SC performance and durability, which may include thermal conductivity and stability, optical transparency combined with haze control, mechanical robustness under repeated flexing, and moisture resistance. Hybridization strategies, such as combining cellulose with inorganic nanoparticles, conductive polymers, or lignin derivatives, can tailor these properties,^641,674,888^ but must be evaluated for trade-offs in processing complexity and long-term stability. Third, molecular or nanoconfinement approaches, including cross-linking to improve thermal stability, surface treatments to enhance moisture barrier properties, may extend film lifespan. Fourth, scalable, green processing methods, including water-based coatings, low-temperature curing, and use of abundant biomass feedstocks, should be prioritized to control costs and environmental footprint. Finally, application-specific targeting may yield nearer-term impact: rather than seeking to replace silicon modules broadly, LCFs may find initial roles in niche markets such as portable or wearable photovoltaics, biodegradable sensors, building-integrated coatings with self-cleaning or light-management functions. In such contexts, the unique advantages of LCFs, lightweight, flexible, biodegradable, can outweigh some performance limitations. Integrating these recommendations into future studies will help clarify when and how lignocellulose-based films can transition from intriguing demonstrations to viable components in sustainable solar technologies.

**Table 9:** LCF Substrates vs Conventional Materials in Solar Cells

substrate	power conversion efficiency (%)	stability	cost	flexibility	haze (%)	ref
glass	18–25	excellent	medium	no	<5	^889,890^
PET	11–21	moderate	high	yes	∼10	^891,892^
transparent wood	∼17	moderate	medium	yes	∼90	^356,884^
CNF/epoxy	∼16	good	medium	yes	54	^875,893^

#### 5.4.2 Nanogenerators

As traditional fossil fuel consumption surges, the exploration of alternative renewable energy sources has garnered significant attention.^894,895^ Over recent decades, extensive efforts have been dedicated to advancing renewable options, such as wind, hydro, ocean, and solar power, which have made substantial contributions to the global energy supply.^896–898^ However, conventional renewable energy solutions typically require massive infrastructure, heavy turbines, and expensive electromagnetic generators,^899,900^ posing challenges in harnessing low-density mechanical energies like gentle breezes, water flow, and human limb movement.^900,901^ Moreover, portable electronic devices and microsensors usually demand minimal power and are considerably smaller than traditional batteries. To address these challenges, a new class of devices known as nanogenerators has emerged, capable of efficiently capturing micromechanical energy and converting it into electricity.^902,903^ Dr. Zhonglin Wang pioneered the concept of nanogenerators (NGs), which, unlike traditional electromagnetic induction, convert small-scale mechanical energies into electricity. This innovation has led to the development of piezoelectric nanogenerators (PENGs)^904,905^ and triboelectric nanogenerators (TENGs).^906,907^ NGs have attracted significant interest from both researchers and industries due to their great potential for providing portable, cost-effective solutions for energy harvesting, and self-powered sensing.

The concept of nanogenerators (NGs) is fundamentally linked to Maxwell’s displacement current theory,^908,909^ which extends classical electromagnetic theory by accounting for time-varying electric fields in dielectric media. In the context of NGs, particularly TENGs, this theory provides a physical basis for understanding how alternating electric fields generated by mechanical motion can induce displacement currents, thereby enabling charge transfer and energy conversion. Understanding this fundamental basis helps bridge the knowledge gap for materials scientists aiming to tailor sustainable materials, such as cellulose, for efficient energy harvesting. Similarly, the piezoelectric effect occurs when a material produces an internal electric potential in response to an external force, causing a shift in the positions of its positive and negative ions. This shift creates an electric voltage, enabling the conversion between mechanical and electrical energy. PENGs leverage this principle to transform mechanical motion into electrical energy. Typically, these devices consist of flexible materials, external loads, and piezoelectric elements. For instance, Wang et al. introduced a PENG based on a single zinc oxide (ZnO) nanowire (Figure 44a).^910^ When force is applied perpendicular to the nanowire, its unique crystal structure allows it to generate electricity through bending or stretching. Initially, the ions within the nanowire are balanced; however, applying pressure compresses one side while extending the other, leading to charge separation. The high flexibility and deformability of ZnO nanowires make them particularly efficient at converting mechanical energy into electrical output, as structural changes during deformation generate charge and the semiconductor properties of ZnO facilitate current flow.

**Figure 44:**
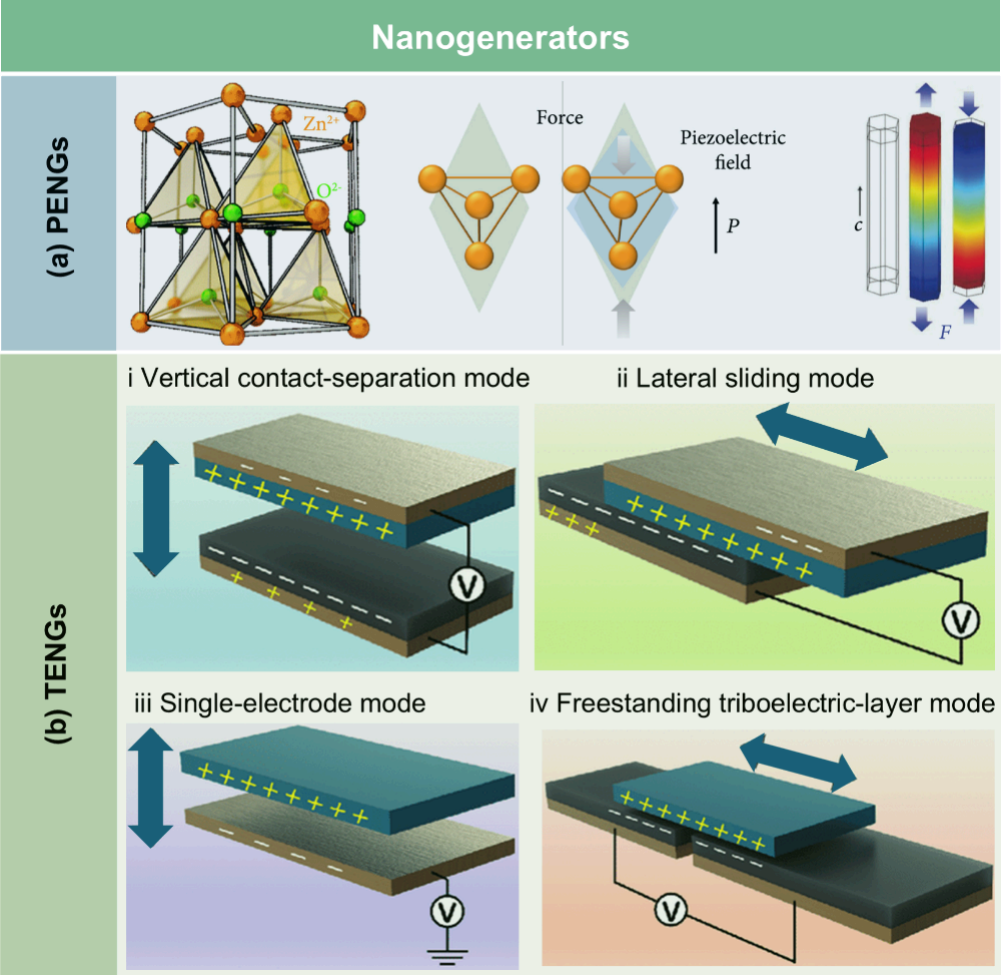
(a) Structure and working mechanism of the PENG based on ZnO nanowire. Adapted with permission from ref 910. Copyright 2006 American Chemical Society. (b) Four fundamental working modes of TENGs: vertical contact-separation mode (i), lateral sliding mode (ii), single-electrode mode (iii), and freestanding triboelectric-layer mode (iv). Adapted with permission from ref 911. Copyright 2022 Taylor and Francis under CC BY 4.0 (https://creativecommons.org/licenses/by/4.0/).

A TENG operates based on the principles of triboelectricity and electrostatic induction. When two distinct materials come into contact and then separate, they acquire opposite charges due to their differing electron affinities. Connecting these charged materials through an external circuit results in an electric current driven by the potential difference between them. TENGs are typically categorized into four working modes (Figure 44b): vertical contact-separation, lateral sliding, single-electrode, and freestanding triboelectric layer modes.^912^ These four operating modes differ in geometry and contact motion, but all rely on the same fundamental principle: generating and harvesting electrostatic charges through relative movement. For cellulose-based materials, which often serve as active layers in TENGs, choosing the right mode is essential to optimize charge transfer and durability under repeated mechanical stress. In the vertical contact-separation mode, the two contact layers become oppositely charged upon contact. As they separate, using air as the dielectric medium, the capacitance between them decreases with increasing distance. Even if the negative contact layer is nonconductive, its generated negative charges remain fixed. To balance the charge imbalance, electrons are transferred from the metal electrode connected to the negative layer to the positive layer via an external circuit. Conversely, when the layers approach each other and the capacitance increases, electrons near the positive layer return to the metal electrode behind the negative layer. In the lateral sliding mode, the configuration resembles that of the contact-separation mode; however, one contact layer slides across the other instead of repeatedly coming into and out of contact. Although the materials remain in continuous contact during sliding, the misalignment of frictional charges alters the effective polarization area. This variation in the overlap area drives the back-and-forth movement of electrons in the external circuit to counteract the changing electric field. The single-electrode mode distinguishes itself by connecting only one electrode to an external circuit. As depicted in Figure 44biii, the charges generated by the two contact materials charge a capacitor formed between the connected electrode and a reference electrode. Variations in the distance between the contact layers alter the capacitance, thereby inducing charge transfer between the capacitor plates through the external circuit. The freestanding TENG is an enhanced version of the single-electrode design. By incorporating a freestanding layer, this configuration fully harnesses the induced potential between two integrated electrodes. Theoretically, this design can achieve up to 100% efficiency. Depending on how the contact layers interact, the freestanding TENG can operate in either the contact-separation or sliding-friction mode, with its electricity generation mechanism being analogous to that of the vertical contact-separation and lateral sliding modes.

In recent years, a new generation of materials for NGs has emerged, with a particular focus on biobased materials as active components or substrates.^913,914^ Among these, LCFs have shown considerable promise. The selection of LCFs as a nanogenerator material is driven by several compelling advantages. First, cellulose is the most abundant biopolymer on Earth and is fully biodegradable, making it a sustainable alternative to synthetic polymers commonly used in NGs. Second, its molecular structure, rich in hydroxyl groups and lacking centrosymmetry, enables intrinsic piezoelectric and triboelectric behavior. These hydroxyl groups also facilitate facile chemical modifications, allowing fine-tuning of surface charge density, polarity, and compatibility with functional fillers or electrode interfaces.^915^ Moreover, cellulose exhibits excellent mechanical flexibility, hydrophilicity, and low cytotoxicity, which collectively make it attractive for wearable, implantable, or environmentally benign energy-harvesting devices. By leveraging both its functional and ecological merits, LCF-based NGs offer a promising route toward next-generation sustainable electronics.

##### 5.4.2.1 Lignocellulosic Films in PENGs

Early piezoelectric materials mainly included semiconductors (e.g., GaN, ZnO),^916,917^ ceramics (e.g., BaTiO_3_, KNbO_3_),^918^ and synthetic polymers (e.g., polyamides, PDMS)^919,920^ that are generally high-density and brittle.^921^ In contrast, natural piezoelectric materials, particularly cellulose, offer distinct advantages such as low-cost, renewability, biodegradability, and environmentally compatibility. Cellulose exists primarily in two crystalline forms: cellulose I, with a triclinic structure, and cellulose II, characterized by its monoclinic structure. Both forms lack a center of symmetry, and their hydroxyl groups exhibit strong polarity. This noncentrosymmetric arrangement of polar hydrogen bonds induces a net dipole moment, giving rise to piezoelectric behavior. These structural properties make cellulose crystals promising candidates for piezoelectric applications.^90,922^

Fukako first reported the piezoelectric effect of cellulose in 1955.^923^ The shear piezoelectric coefficient *d*_25_ = −*d*_14_ of wood cellulose fiber was measured to be on the order of 10^–9^, confirming cellulose’s piezoelectric activity. Similarly, Rajala et al.^924^ fabricated a self-standing, 45 μm thick CNF film as a piezoelectric sensor material (Figure 45ai). The films exhibited relative permittivity values of 3.47 at 1 kHz and 3.38 at 9.97 GHz, with corresponding loss tangents (tan δ) of 0.011 and 0.071, respectively. These films were employed as functional sensing layers in piezoelectric sensors (Figure 45aii), achieving sensitivity between 4.7 and 6.4 pC·N^–1^ under ambient conditions. Compared with PVDF, a standard reference piezoelectric material, the CNF film demonstrated considerable potential as a precursor for disposable piezoelectric sensors.

**Figure 45:**
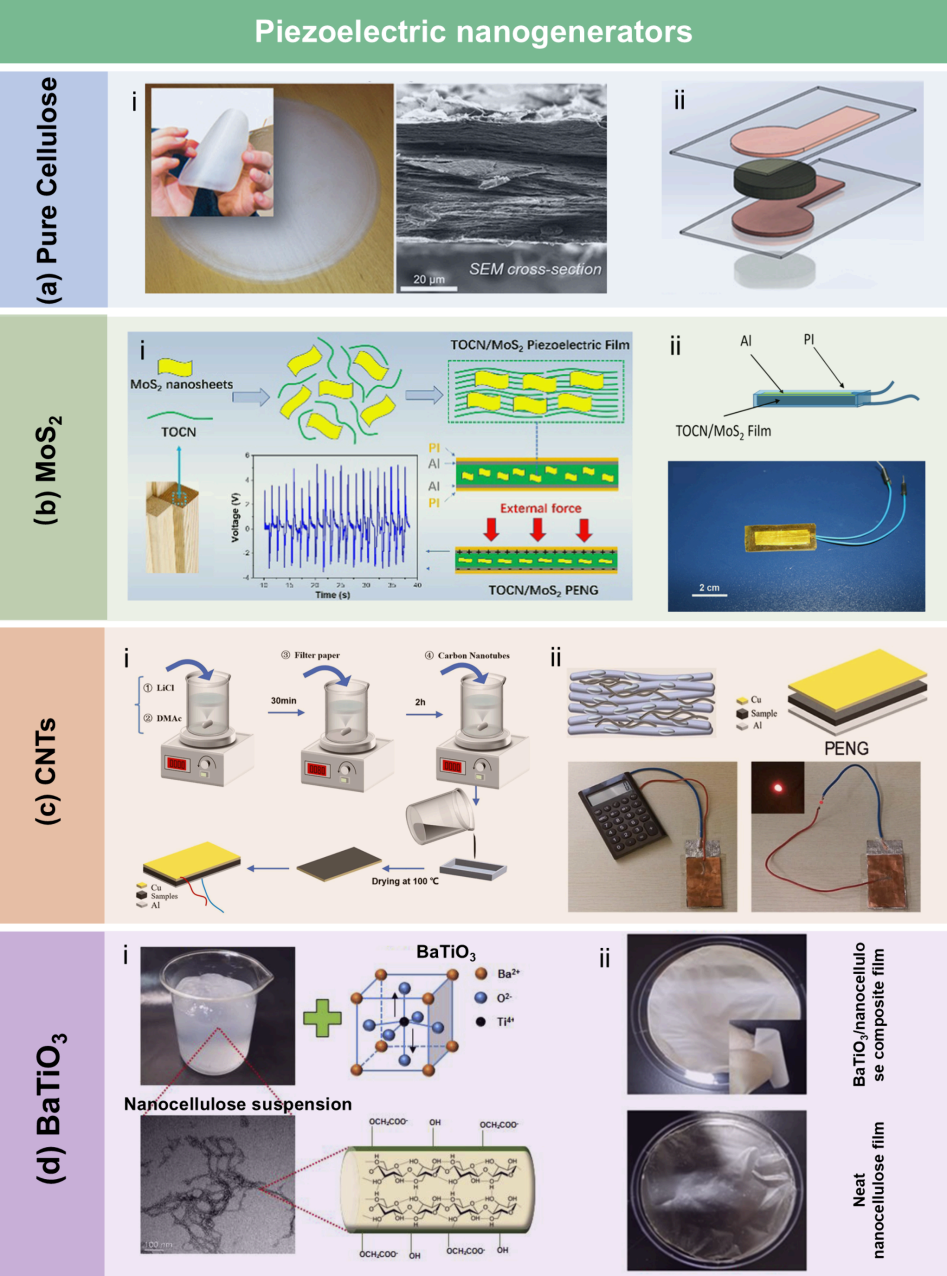
Application of LCFs in PENGs. (a) Photographs and SEM cross-section image of a fabricated self-standing CNF film (i); Schematic side view of the assembled sensor (ii). Adapted with permission from ref 924. Copyright 2016 American Chemical Society. (b) Schematic illustration of the TOCN/MoS_2_ composite film as a PENG (i) and device structure of the TOCN/MoS_2_ PENG (ii). Adapted with permission from ref 925. Copyright 2020 Elsevier. (c) Schematic illustration of the preparation process of CNTs/cellulose nanocomposite film for PENG (i); schematic diagram of the assembled PENG device and the demonstration for drive a calculator and light up a LED lamp. Adapted with permission from ref 926. Copyright 2023 Elsevier. (d) Schematic procedure to fabricate neat nanocellulose and BaTiO_3_/nanocellulose films (i), and the corresponding photographs (ii). Adapted with permission from ref 929. Copyright 2019 Elsevier.

However, the intrinsic piezoelectric output of pristine cellulose films is relatively weak, limiting its standalone performance in PENGs. To address this, various strategies have been employed to enhance its electromechanical response, primarily through the incorporation of high-dielectric or piezoelectric fillers.

Various nanocomposite strategies have been explored to enhance the piezoelectric performance of cellulose-based materials. For example, Wu et al.^925^ developed a piezoelectric film by fabricating a bionanocomposite consisting of TEMPO-oxidized cellulose nanofibrils (TOCN) and molybdenum disulfide (MoS_2_) nanosheets (Figure 45bi). The incorporation of MoS_2_ not only enhanced the film’s piezoelectric performance but also improved its mechanical properties (Figure 45bii). This structure achieved a longitudinal piezoelectric constant (*d*_33_) of 31 pC·N^–1^, significantly higher than the 12 pC·N^–1^ of neat TOCN, while also improving the film’s Young’s modulus and tensile strength (8.2 GPa and 307 MPa, respectively). However, the electrical output of the corresponding nanogenerator remained relatively low, with an open-circuit voltage of 4.1 V and current of 0.21 μA, which may limit its standalone applicability. In contrast, Hou et al.^926^ fabricated a flexible PENG using a CNT-doped cellulose-based nanocomposite film (CNTs/cellulose) that delivered milliampere level currents (Figure 45c). Meanwhile, Alam et al.^923^ demonstrated a hybrid approach using native cellulose microfibers (NCMF), PDMS matrix, and multiwall CNTs as conductive fillers, yielding a device with moderate voltage output (∼30 V) and power density (9.0 μW·cm^–3^), without requiring chemical modification or electrical poling. This work highlights the practicality of simple fabrication and lead-free composition, although it may compromise electrical current output.

Polymer blending has emerged as an effective route to enhance the piezoelectric performance of cellulose-based systems by promoting favorable crystalline phases and improving charge separation. Fu and colleagues^927^ employed CNC as nucleating agents within a PVDF matrix, leading to a notable increase in the electroactive β-phase content and overall crystallinity. With just 2 wt % CNC loading, the composite membrane delivered a peak output voltage of 60 V, demonstrating that minimal cellulose content can induce significant structural transformation and enhance dipolar alignment. However, the study primarily focused on voltage output, with limited discussion of current density or long-term mechanical stability. By contrast, Ponnamma et al.^928^ designed a more complex ternary hybrid composite incorporating nanocellulose, PVDF, and Fe-doped ZnO nanoparticles. This system yielded a peak-to-peak output voltage of 12 V and a current density of 1.9 μA·cm^–2^, representing 60-fold and 2.3-fold improvements over neat PVDF fibers, respectively. The synergistic effect of nanoparticle doping and nanocellulose reinforcement contributed not only to enhanced piezoelectricity but also to improved dielectric and mechanical properties. Nonetheless, the relatively lower voltage output compared to the CNC/PVDF system suggests a potential trade-off between charge mobility and overall dipole alignment in multiphase systems.

Incorporating high-permittivity inorganic fillers is a common strategy to enhance the piezoelectric performance of cellulose-based films. For instance, Choi and Jeong^929^ synthesized a nanocellulose composite film incorporating varying amounts of BaTiO_3_ nanoparticles (Figure 45d). They observed that the piezoelectric output voltage and current increased with BaTiO_3_ content up to 40 wt % but decreased at higher concentrations (50–60 wt %). This was attributed to a trade-off between enhanced dielectric polarization and the adverse effects of increased mechanical stiffness, which suppressed deformation under mechanical stimuli.

Enhancing the internal alignment of cellulose chains is another critical pathway to improve piezoelectric response, owing to the anisotropic nature of dipole orientation along the cellulose backbone. Theoretical calculations of hydrogen bonding within the unit cell structure suggest that CNCs could exhibit a piezoelectric coefficient as high as 4–36 pm·V^–1^.^930^ Wang et al.^931^ addressed the common limitation of random orientation by adapting confinement cell technology, originally used for colloidal assembly, to achieve large-scale vertical alignment of CNCs. By combining shear-induced torque and a high-energy PTFE interface, and further applying a DC electric field to unify dipole orientation, they fabricated vertically aligned CNC films with a *d*_33_ of 19.3 ± 2.9 pm·V^–1^. This performance rivals that of commercial PVDF (20–30 pm·V^–1^) and underscores how alignment engineering can unlock the intrinsic piezoelectricity of cellulose without relying on external fillers.

Generally, incorporating high-dielectric or piezoelectric fillers (e.g., BaTiO_3_, MoS_2_) significantly improves electrical output, but often compromises flexibility and mechanical compliance. Meanwhile, polymer blending (e.g., with PVDF or PDMS) enhances both flexibility and output, albeit at the cost of increased fabrication complexity and reduced biodegradability. Alignment strategies (e.g., CNC orientation) improve output but are difficult to scale up. Overall, cellulose-based PENGs have demonstrated a wide range of output performances depending on the type and amount of incorporated piezoelectric fillers. For instance, materials such as MoS_2_ and CNTs significantly boost piezoelectric output but may compromise film transparency or mechanical flexibility at high loadings. In contrast, PVDF-based hybrids offer higher piezoelectric constants but are less sustainable. Notably, optimal performance often results from a trade-off between electrical output and mechanical compliance. Therefore, rational design of cellulose composites should consider not only the piezoelectric enhancement but also the environmental impact and flexibility required for practical applications.

##### 5.4.2.2 Lignocellulosic Films in TENGs

Common materials used for TENGs include those with positive triboelectric properties such as polyamides^932^ and metals,^933^ and those with negative triboelectric properties, such as fluorinated ethylene propylene (FEP),^934^ polytetrafluoroethylene (PTFE),^932,935^ and PET.^936^ These materials are typically paired in TENGs due to their opposite tendencies to gain or lose charges upon contact. LCFs, particularly those derived from cellulose, have emerged as promising candidates for TENGs owing to their natural abundance, biodegradability, and electron-donating capabilities (Table 10). Notably, the cellulose molecular chain is rich in hydroxyl groups, and the lone electron pairs on the oxygen atoms enhance cellulose’s ability to donate or accept electrons when in contact with other materials. This characteristic endows cellulose with excellent triboelectric properties, making it a high electron-donating material.^922^

**Table 10:** Summary of Research Progress of Lignocellulosic Film-Based TENGs^a^

positive materials	negative materials	TENG output	ref
CNF film	FEP	∼30 V/∼90 μA	^937^
CNC film	PDMS	28 V/2.8 μA	^938^
CNF film	FEP	195 V/13.4 μA	^939^
BC/ZnO film	Teflon	49.6 V/4.9 μA	^940^
polyamide	PFOTES-CNF film	28.5 V/9.3 μA	^941^
chemically modified BC film	PTFE	64 V/5.94 μA or 76.7 V/6.35 μA	^942^
chemically modified cellulose film	chemically modified cellulose film	4.86 V	^943^
BC/SA/BTO composite film	FEP	530.1 V/62.4 μA	^944^
CNF/MXene film	PDMS	24.9 V/7.4nC	^945^
lignocellulosic film	PTFE	31 V/0.2 μA	^946^

a Note: CNF, cellulose nanofiber; FEP, fluorinated ethylene propylene; CNC, cellulose nanocrystal; BC, bacterial cellulose; PDMS, polydimethylsioxane; SA, sodium alginate; BTO, barium titanate; PTFE, polytetrafluoroethylene.

Wang’s group first demonstrated the functionalization of cellulose nanomaterials for TENG development in 2016.^937^ They fabricated a transparent and flexible thin film of CNF and paired it with FEP to assemble a TENG device (Figure 46ai). This CNF-based TENG was subsequently integrated into a fiberboard made from recycled cardboard fibers using a chemical-free cold-pressing method, demonstrating sustainable fabrication. Under the pressure of a normal human step, the fiberboard generated electric outputs of up to ∼30 V and ∼90 μA. Similarly, Shi et al.^946^ prepared a lignocellulose Bioplastic and developed an all-lignocellulosic triboelectric nanogenerator for self-powered medical monitoring (Figure 46aii). Although the electrical output was relatively lower than that of hybrid TENGs, this study highlights the potential of lignocellulosic systems in sustainable wearable electronics without reliance on synthetic polymers.

**Figure 46:**
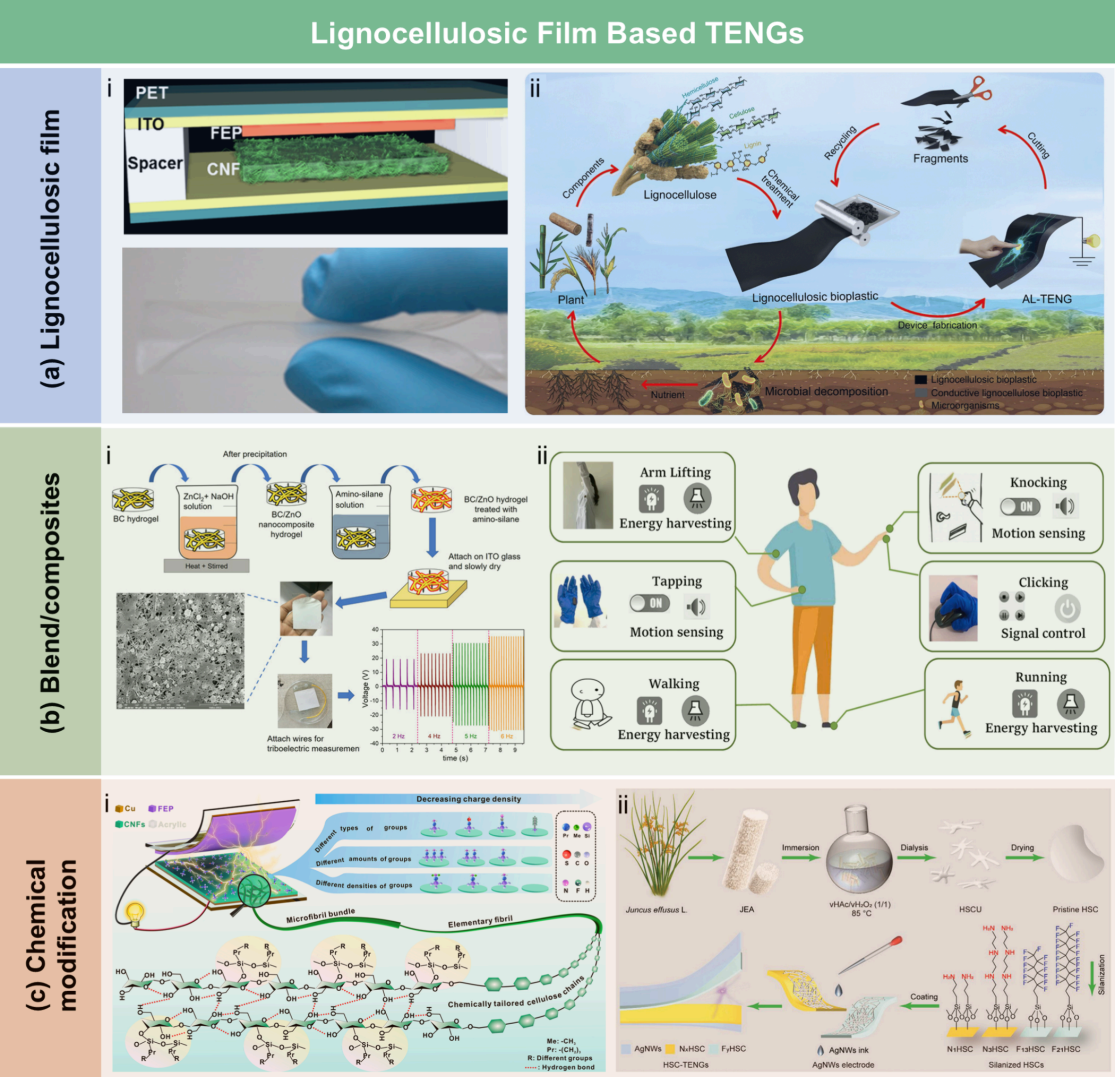
Application of LCFs in TENGs. (a) Schematic diagram of the CNF film-based TENG and a digital photo of the CNF film (i). Adapted with permission from ref 937. Copyright 2016 Elsevier. Schematic diagram of biodegradability and recyclability of the lignocellulose Bioplastic and all-lignocellulosic triboelectric nanogenerator (AL-TENG) (ii). Adapted with permission from ref 946. Copyright 2023 Royal Society of Chemistry. (b) Schematic illustration of the fabrication of BC/ZnO (ZBC) nanocomposite film for TENG (i). Adapted with permission from ref 940. Copyright 2020 American Chemical Society. Illustration of the self-powered wearable sensors for monitoring different human motions (ii). Adapted with permission from ref 944. Copyright 2023 Elsevier. (c) Schematic diagram of chemical functional groups tailored CNFs for manipulating the charge density (i). Adapted with permission from ref 947. Copyright 2021 Elsevier. Schematic diagram of the hollow stellate cellulose (HSC) film preparation process and the assembled TENG structures (ii). Adapted with permission from ref 943. Copyright 2023 Wiley-VCH.

To further enhance the output performance of TENGs fabricated from LCFs, several strategies have been explored, including chemical functionalization and polymer blending. For instance, Feng et al.^944^ prepared a transparent film based on bacterial cellulose (BC) and sodium alginate (SA) that incorporated barium titanate (BTO) nanoparticles. The addition of BTO increased the voltage and current by 174% and 193%, respectively. Furthermore, the designed TENG device can function as sensors for biomechanical monitoring, touch detection, and signal control, significantly promoting the development of high-performance, eco-friendly, and cost-effective TENGs based on naturally biodegradable materials (Figure 46bii). Similarly, Jakmuangpak et al.^940^ adopted a simpler, paste-free fabrication approach by directly drying bacterial cellulose (BC) hydrogel on an indium tin oxide (ITO)­electrode and subsequently impregnating ZnO nanoparticles into the BC network (Figure 46bi). Furthermore, ZnO nanoparticles were impregnated into the BC nanostructure, particularly at the film surface, to enhance both surface roughness and polarizability. Because the resulting BC/ZnO (ZBC) nanocomposite could not directly bond to the substrate, it was modified by amino-silane treatment prior to drying. Triboelectric measurements indicated that the bio-TENGs based on both BC and ZBC films generated relatively high open-circuit voltages (*V*_oc_) and short-circuit currents (*I*_sc_) compared to BC-TENGs produced via other methods. This result underlines the critical role of interface adhesion and filler localization in performance optimization. On the other hand, incorporating cellulose materials into MXene films can enhance TENG output while also improving the composite film’s mechanical strength, flexibility, and sensitivity to temperature and strain. Yang et al.^945^ found that when the CNFs/MXene ratio was set to 2/5, the maximum charge and output voltage reached 7.4 nC and 24.9 V, respectively. Additionally, the as-prepared TENG achieved a maximum instantaneous output power density of 1.2 mW·m^–2^. Moreover, the composite exhibited enhanced mechanical strength and environmental responsiveness, making it promising for flexible or wearable sensing platforms.

Beyond the incorporation of triboelectric fillers, chemical functionalization has emerged as a versatile strategy to tailor the surface properties of cellulose films and improve triboelectric output. For instance, Boonchai et al.^942^ applied phosphorylation and sulfonation treatments to bacterial cellulose (BC) films using aqueous phosphoric and sulfuric acid solutions, respectively. These chemical modifications introduced negatively charged phosphate and sulfonate groups, which increased the surface polarity and hydrophilicity of the BC network. SEM and AFM characterizations revealed a denser nanofibril structure and higher surface roughness after treatment. When paired with PTFE in a single-electrode TENG configuration, the modified BC films exhibited significantly higher open-circuit voltage and short-circuit current than pristine BC, mainly due to enhanced triboelectric charge density and better contact intimacy with the electrode. Chen et al.^943^ prepared hollow stellate cellulose (HSC) films and modified them via silane treatment with different functional groups: specifically, amino (−NH_2_) and fluoro (−F) groups. Since the amine group has the lowest tendency to attract electrons while the fluoro group has the highest, the differences in electronegativity significantly affected the free energy and polarizability of the friction surface, thereby influencing surface wettability and work function in TENG devices. As a result, these all-in-one HSC TENGs achieved a maximum output voltage of 4.86 V, which is 28 times higher than that of TENGs constructed with two pristine HSC films as triboelectric layers (PHSC-TENG) (Figure 46cii). Similarly, Liu et al.^947^ demonstrated that CNF films treated with different alkoxysilane coupling agents (bearing methyl, amino, or fluorinated terminal groups) exhibited tunable work functions and triboelectric polarities (Figure 46ci). The silane grafting process was carried out in ethanol solutions at 60 °C for 6 h, and XPS confirmed successful modification. The resulting films showed a strong correlation between charge density and surface electronegativity, and TENG output could be optimized by selecting appropriate donor–acceptor combinations. Notably, the authors also examined mechanical flexibility and surface adhesion after modification, which are often overlooked in triboelectric design.

For TENGs, trends indicate that surface functionalization (e.g., sulfonation, silanization) and polymer blending promote charge transfer and improve output, but often introduce trade-offs such as complex processing steps and reduced long-term mechanical durability. The use of high-permittivity nanoparticles enhances performance but may stiffen the material or reduce environmental compatibility. Additionally, pairing cellulose with strongly electronegative materials (e.g., PTFE, FEP) remains an effective route to maximize triboelectric difference. The triboelectric output of cellulose films varies depending on surface functionalization, filler incorporation, and electrode configuration. While chemical modification strategies, such as silanization, effectively enhance charge transfer, it may introduce processing complexity. Similarly, adding nanoparticles like BTO or ZnO improves output performance but can stiffen the film and reduce biodegradability. The choice of positive and negative material pairs also significantly influences performance. These trends highlight the importance of balancing triboelectric enhancement, material sustainability, and device scalability when designing cellulose-based TENGs.

In summary, lignocellulosic film-based nanogenerators hold significant potential for meeting the global demand for sustainable energy by harvesting small-scale mechanical energies, such as human movement and environmental vibrations. However, several challenges persist, with the most pressing being the enhancement of both the nanogenerators’ output and the films’ flexibility. Compared to flexible synthetic polymers, for example, PENG polymers like PVDF and TENG polymers such as polyamide, LCFs generally offer lower flexibility and reduced output. To overcome these limitations, incorporating flexible polymers with piezoelectric or triboelectric materials into the lignocellulosic matrix could improve both the flexibility and performance of these nanogenerators. For TENGs especially, the chemical functionalization of LCFs offers an additional promising approach. Despite these advances, achieving large-scale fabrication and practical device integration remains a critical hurdle. Continued interdisciplinary efforts in materials design, interface engineering, and device architecture will be essential to fully realize the potential of lignocellulosic nanogenerators in next-generation sustainable technologies.

### 5.5 Sensors

Sensors are analytical devices designed to detect and measure physical, chemical, or biological signals by converting them into electrical or optical outputs. They play a critical role in various fields, including healthcare, environmental monitoring, industrial control, and consumer electronics. LCFs, derived from renewable biomass, have emerged as promising materials for sensor development due to their biocompatibility, sustainability, large surface area, and ability to be easily functionalized. The diverse chemical composition of LCFs, which includes cellulose, hemicellulose, and lignin, offers a versatile platform for developing sensors that can detect mechanical deformations, chemical pollutants, and biological analytes with high sensitivity and specificity. LCF-based sensors, such as strain sensors, chemical sensors, and biosensors, hold significant potential for applications in medical diagnostics, wearable devices, environmental monitoring, and food safety, aligning with the growing demand for eco-friendly and cost-effective sensing solutions.

#### 5.5.1 Strain Sensors

Strain sensors are devices that monitor mechanical deformations, including stretching, bending, twisting, and compression, by transforming these physical changes into detectable electrical signals. They play a crucial role in fields such as wearable technology, soft robotics, healthcare monitoring, and structural integrity analysis. As the demand for environmentally friendly, flexible, and cost-effective sensing materials grows, research has increasingly focused on the development of LCF-based strain sensors to meet these requirements.

The working principle of LCF-based strain sensors typically involves the piezoresistive effect, where mechanical deformation leads to variations in electrical resistance. Conductive nanomaterials like CNTs, graphene, AgNWs, or MXene can be incorporated into the LCF structure to form a conductive network. When the film undergoes mechanical stress, the conductive pathways within the composite are disrupted, resulting in measurable changes in resistance. This mechanism allows LCF-based strain sensors to accurately detect and measure a wide range of mechanical movements, making them highly suitable for flexible and sustainable sensing applications.

LCFs can serve not only as passive mechanical substrates but also as active components that contribute to the flexibility, biocompatibility, and responsiveness of the sensing films. In some cases, LCFs actively participate in the sensing process by modulating the mechanical or electrical response, enhancing sensitivity, durability, or environmental resistance. Their high aspect ratio, intrinsic polarity, and compatibility with multiple fillers enable them to participate in the sensing process beyond passive support.

Nanocellulose, a native nanostructured form of cellulose with high mechanical strength and flexibility, plays a prominent role in the development of LCF-based strain sensors. Its high surface area and aspect ratio enable the formation of strong and flexible networks that can support the conductive fillers, ensuring the mechanical integrity of the sensor. Several nanocellulose-based composite films have been developed to detect various human motions. For instance, flexible CNF films modified by esterification and coated with AgNWs,^558^ hybridized with graphene quantum dots (GQDs),^948^ or integrated with rGO and nitrile rubber^949^ exhibit high flexibility, conductivity (up to 99 S/m), and structural stability, maintaining performance over thousands of deformation cycles. These films are capable of monitoring movements such as wrist and elbow bending, finger flexion, and other biomechanical activities with high sensitivity and real-time response. Additionally, piezoresistive sensors composed of CNC-rGO composites encapsulated in PDMS^950^ show rapid response times and durability under harsh chemical environments (Figure 47ai), while MFC-based piezoelectric films combined with PZT^951^ demonstrate excellent performance in plantar pressure monitoring and human health diagnostics (Figure 47aii).

**Figure 47:**
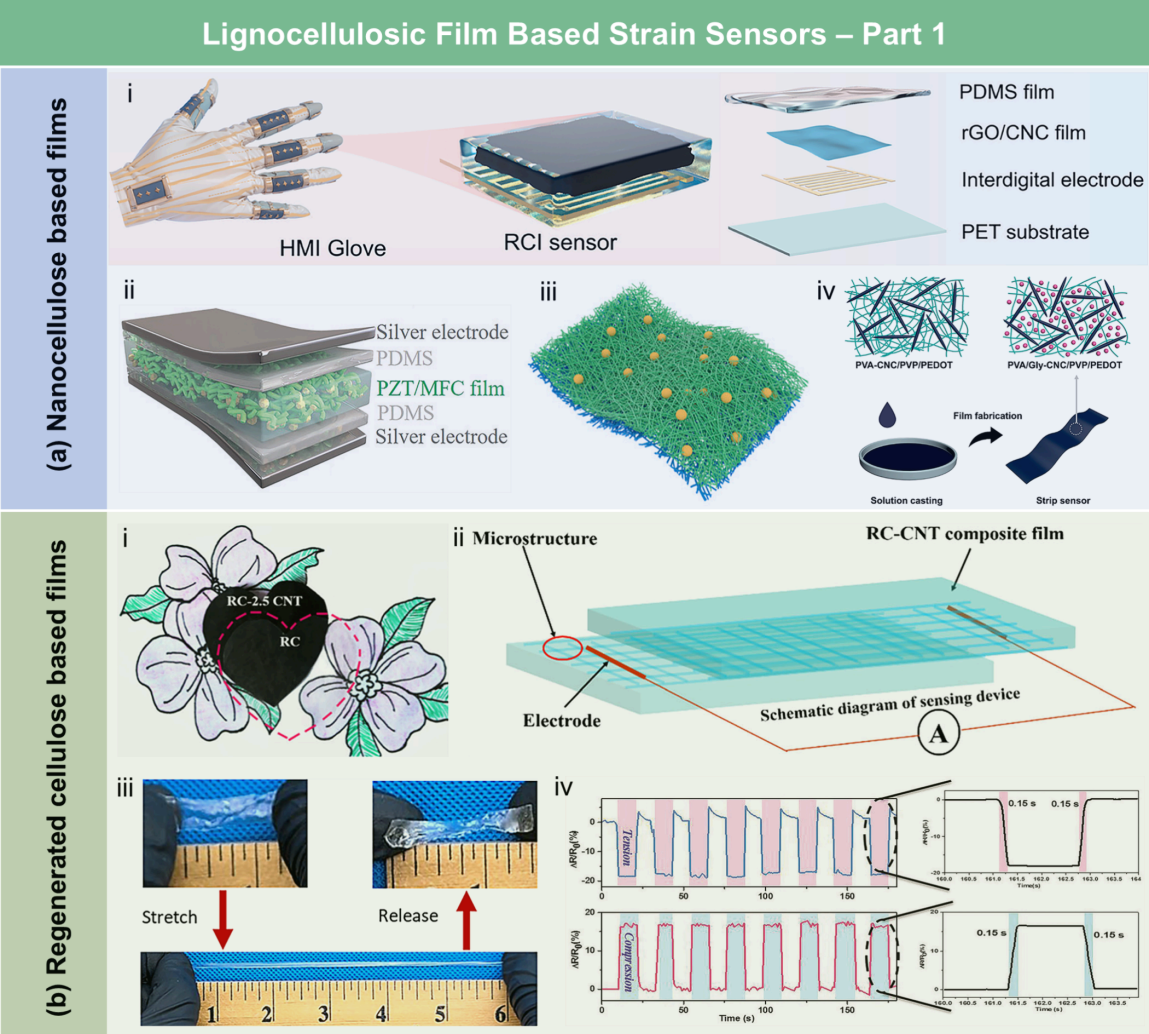
Application of LCFs in strain sensors. (a) Pressure sensor based on CNC/rGO film (i). Adapted with permission from ref 950. Copyright 2024 Elsevier. Pressure sensor based on MFC/PZT film (ii). Adapted with permission from ref 951. Copyright 2022 Royal Society of Chemistry. Schematic illustration of the CPNF membrane (iii). Adapted with permission from ref 954. Copyright 2021 Elsevier. Schematic images of the PVA/Gly-CNC/PVP/PEDOT film (iv). Adapted with permission from ref 955. Copyright 2021 American Chemical Society. (b) Appearance comparison of the pure RC film and RC-2.5CNT film (i); Schematic illustration of the RC-CNT film-based sensor (ii). Adapted with permission from ref 956. Copyright 2020 Springer Nature. Images of RC/PAA films under mechanical stretching (iii). Adapted with permission from ref 957. Copyright 2024 American Chemical Society. The normalized resistance changes in the tensile and compressive strain applied by bending the sensor from 30 to 25 mm. The inset shows the time response of the sensor applied tensile and compression strain (iv). Adapted with permission from ref 958. Copyright 2018 Royal Society of Chemistry.

The application of nanocellulose extends beyond piezoresistive and piezoelectric mechanisms. Cholesteric cellulose derivatives, such as cholesteric CNCs, enable the construction of optical strain sensors. These materials exhibit vivid structural colors due to their helical molecular alignment. Upon mechanical deformation, changes in the helical pitch cause observable color shifts, allowing for visual, contactless detection of strain. For example, flexible CNC-based optical sensors have demonstrated distinct and reversible color changes in response to stretching, enabling real-time visual monitoring of deformation and expanding the functionality of nanocellulose in smart optical sensing.^952,953^ Piezoelectric layered CNF films have also been developed for self-powered sensors,^954^ demonstrating stable output under repeated deformation (Figure 47aiii). Conductive CNC-PEDOT composite films^955^ offer high stretchability (up to 500%), excellent electrical performance, and long-term stability, detecting both subtle and dynamic motions such as wrist pulses and muscle twitches (Figure 47aiv).

RC films, produced through dissolution and regeneration processes, exhibit superior purity, uniformity, and mechanical strength. Their inherent flexibility and stable electrical properties position them as a promising material for strain sensing applications. For example, Xie et al.^956^ developed a flexible E-skin sensor using a composite film of RC and carbon CNT (Figure 47bi). The RC was prepared using a NaOH/urea aqueous solution, followed by the addition of CNT, forming a composite film through casting and regeneration in sulfuric acid (Figure 47bii). The RC–CNT sensor exhibited excellent flexibility, a tensile strength of 61.6 MPa, and high sensitivity to external pressure. The E-skin was able to detect subtle human body movements such as finger bending and throat swallowing, demonstrating stable sensing performance and excellent biocompatibility without cytotoxicity. In addition, Yang and colleagues developed tough and elastic cellulose composite hydrogels and films designed for flexible, wearable strain sensors.^957^ Using a water-ZnCl_2_ solution to dissolve cellulose, they created ionic gels through the in situ polymerization of acrylic acid (AA), forming an interpenetrating network with PAA. The resulting cellulose/PAA composite films exhibit high elasticity, with an elongation up to 1,300%, and a tensile strength of 10–15 MPa (Figure 47biii). These films were successfully applied as capacitive strain sensors, showing stable performance in detecting mechanical deformations such as bending and stretching, making them suitable for wearable sensor applications. Moreover, Xu et al.^958^ developed a multifunctional wearable sensor based on a rGO and inverse opal acetylcellulose (IOAC) film, capable of simultaneously monitoring human motion and sweat ion concentrations. The sensor demonstrated high sensitivity in strain sensing, with the resistance change being closely correlated to different bending motions. Specifically, as shown in Figure 47biv, when subjected to tensile bending, the resistance of the sensor decreased, whereas compressive bending resulted in an increase in resistance. The normalized resistance change under tensile stress was –18.5%, while under compressive stress, it increased by 16.6% when bending from 30 mm to 25 mm. This ability to distinguish between different bending directions highlights the sensor’s effectiveness in detecting complex, multidirectional human movements. Additionally, the sensor exhibited an immediate response time of 0.15 s, demonstrating its potential for real-time applications in wearable strain sensors.

Cellulose derivatives, modified through chemical functionalization, offer tunable properties that extend the versatility of cellulose-based films. These derivative films provide enhanced functionality and adaptability for strain sensors, particularly in specialized applications requiring specific chemical or physical attributes. For example, Wibowo et al.^959^ developed recyclable, stretchable wearable sensors using HEC and PEDOT:PSS hybrid films. These sensors demonstrated ultralow hysteresis (2.57% at 50% strain), high sensitivity, and durability over 5000 stretch-release cycles. They effectively detected human motions, such as finger bending and breathing, and maintained performance after recycling, making them ideal for sustainable wearable applications. Han and colleagues developed stretchable and conductive CMC/PEDOT:PSS composite films for on-skin strain sensors.^960^ These films showed high conductivity (∼1.0 × 10^–2^ S/cm) and excellent mechanical stability, with only a 1.25-fold resistance increase under 100% strain. The sensors effectively detected biosignals like respiratory humidity, drinking, and finger bending, with low power consumption, ideal for wearable health monitoring. Based on this, Han et al.^961^ developed highly conductive, flexible, and durable CMC/PEDOT:PSS composite films embedded with AgNWs (Figure 48aiii). These films, with a sheet resistance as low as 7 Ω/sq, were used in wearable heaters and on-skin sensors, effectively detecting human motions such as finger and wrist bending with high sensitivity and low power consumption. In addition, Du et al.^962^ developed a flexible and degradable MXene-methylcellulose piezoresistive sensor (as shown in Figure 48ai), achieving high sensitivity (19.41 kPa^–1^) and durability over 10,000 cycles. The sensor effectively detected human activities, such as pulse and joint movement, and can degrade in H_2_O_2_ within 2 days, making it environmentally friendly for wearable applications. Intriguingly, Varghese et al.^963^ fabricated a high-performance triboelectric nanogenerator (TENG) using CA nanofibers and micropatterned PDMS (Figure 48aii). The device produced a voltage of 400 Vand a power density of 0.9 W·m^–2^, demonstrating its potential as a self-powered vibrational sensor for monitoring machine vibrations and detecting anomalies in devices like CPU fans.

**Figure 48:**
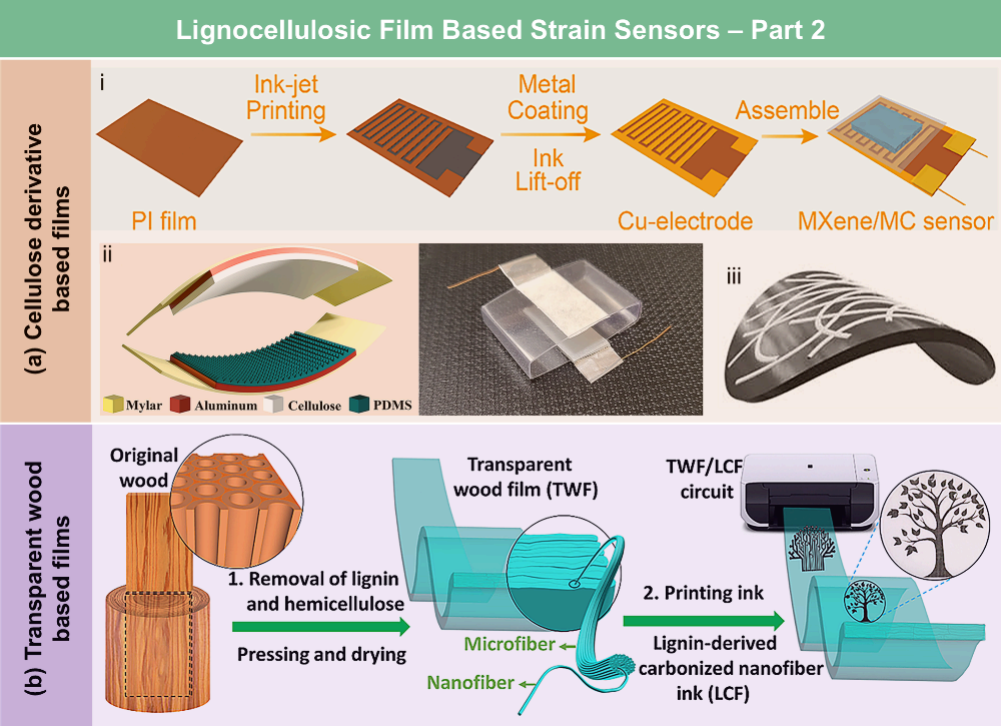
(a) Fabrication of methylcellulose/MXene film-based pressure sensor (i). Adapted with permission from ref 962. Copyright 2024 American Chemical Society. 3D rendered model and actual image of the PDMS and CANF based TENG device (ii). Adapted with permission from ref 963. Copyright 2022 Elsevier. CMC/PEDOT:PSS/AgNWs films for strain sensors (iii). Adapted with permission from ref 961. Copyright 2022 Springer Nature. (b) The fabrication of TWF for flexible electronics applications. Adapted with permission from ref 964. Copyright 2020 American Chemical Society.

LCFs, which retain the natural structure of both cellulose and lignin, present a more robust option for strain sensors. Their complex composition allows for unique mechanical and electrical properties, offering distinct advantages in applications where durability and environmental resistance are key. For instance, Li et al.^965^ developed lignin-containing cellulose nanofibers (LCNF) from walnut shells, combined with multiwalled MWCNTs to create a conductive film. The film showed excellent real-time pressure sensing capabilities, with a conductivity of 15.63 S·m^–1^ and tensile strength of 63.86 MPa, making it suitable for wearable biocompatible sensors. The inclusion of lignin also enhanced the film’s UV-shielding and thermal stability. Using a top-down approach to fabricate LCF is also an effective strategy for developing sustainable electronic devices. Fu et al.^964^ developed a fully wood-based flexible electronic circuit using a transparent wood film (TWF) and a lignin-derived carbon nanofiber conductive ink (Figure 48b). The TWF-based strain sensor demonstrated a relative resistance change of 40% under 2% strain, showing excellent sensitivity to mechanical deformation. After 10,000 folding/unfolding cycles, the sensor maintained stable electrical performance with only an 80% increase in resistance observed after 1,000 cycles, indicating high durability. During bending tests, the sensor attached to a finger joint showed a 30% change in resistance when bent at 90°, effectively tracking human motion, highlighting its potential for wearable strain sensor applications.

Despite the potential of hemicellulose and lignin films for strain sensing, research on these materials has been limited for several reasons. Hemicellulose, though abundant, is challenging to process into stable films due to its amorphous structure and low thermal stability, which makes it less attractive compared to cellulose. Lignin, while offering rigidity and potential conductivity, is highly heterogeneous and difficult to extract and purify in a consistent form, limiting its application in uniform film production. Additionally, the lack of well-established methods to modify and enhance the performance of these materials for strain sensing has further contributed to the scarcity of studies in this area. Consequently, more research is required to address these challenges and unlock the potential of hemicellulose and lignin in advanced sensing technologies.

While numerous examples demonstrate the diverse capabilities of LCF-based strain sensors, general trends are evident. These sensors commonly leverage the inherent flexibility and mechanical robustness of LCF matrices, which help maintain conductive network stability under deformation. Key factors such as filler type, dispersion, interfacial interactions, and structural hierarchy significantly influence sensing performance. Future advancements will depend on strategically optimizing these parameters to enhance sensitivity, durability, and functionality for targeted applications.

#### 5.5.2 Chemical Sensors

Chemical sensors are devices specifically designed to detect the presence and concentration of certain chemical species, such as gases, ions, or organic molecules. LCF-based chemical sensors can function either as passive support matrices or as active components contributing to recognition, transduction, or signal generation. They have shown great potential due to the natural abundance of functional groups (e.g., hydroxyl, carboxyl, and methoxyl groups) present in lignocellulosic materials. These functional groups can be chemically modified to interact with target analytes, thereby enhancing the sensitivity and selectivity of the sensors. To better clarify the sensing mechanisms and strategic role of lignocellulosic materials, this section categorizes LCF-based chemical sensors according to the functional role of LCFs, as either passive supports or active components involved in recognition, transduction, or signal generation. By incorporating conductive materials and specific receptor molecules into LCFs, these films can be used to detect humidity, gases, pollutants, and other chemicals with high sensitivity and tunability.

##### 5.5.2.1 Passive Matrix or Support

Humidity sensors based on LCFs leverage the hydrophilic nature of cellulose and its derivatives, which can absorb and release moisture depending on environmental humidity. This property makes LCFs ideal for detecting changes in RH. In most of these examples, cellulose primarily functions as a structural scaffold or passive matrix that supports the functional sensing materials. Modifications such as the addition of conductive polymers or nanoparticles can further enhance the humidity-sensing performance by improving the film’s electrical response to moisture. The combination of these features enables LCF-based humidity sensors to achieve high sensitivity and fast response times, making them suitable for environmental monitoring and industrial applications. Inspired by nacre, Han and Shen developed a TOCNF/MXene composite film based on 1D TOCNF and 2D MXene nanosheets via vacuum filtration (Figure 49ai). The TOCNF/MXene sensor showed a maximum humidity response of 90% at 97% RH, exhibiting high mechanical strength (128.1 MPa) and durability over 50 bending cycles, making it suitable for wearable electronics.^966^ Likewise, Li et al.^967^ designed a CNF/MXene film (Figure 49aii) that responded quickly to humidity changes, with an induced voltage of 63.7 mV and response/recovery times of 3.2/5.1 s, highlighting its potential in real-time humidity monitoring. In addition, Li et al.^968^ presented a CNF/GO composite film with excellent linear humidity response, fast response times (3–5 s), and stability after 30 cycles, making it ideal for environmental sensors. Wei et al.^969^ integrated CNTs and GO into a CNF-based humidity sensor that demonstrated rapid actuation, bidirectional bending, and long-term durability over 1,000 cycles, with a maximum bending angle of 180° (Figure 49aiii). This sensor was successfully used in a smart switch application, showing its versatility. Zheng et al.^970^ further explored a composite film of chiral-oriented CNC and PEO for optical fiber humidity sensing, achieving ultrahigh sensitivity (0.64 dB/%RH) and fast response times under varying humidity levels. Moreover, cellulose derivatives like CAB combined with rGO offer enhanced precision, with a sensitivity shift of 0.307 nm/%RH across a broad humidity range, providing high-resolution performance.^971^ Further enhancing functionality, films composed of RC and KOH exhibit excellent conductivity changes, with a 200-fold variation across humidity levels from 11.3% to 97.3%, alongside fast response/recovery times of 6.0/10.8 s and minimal hysteresis (0.57%).^972^ Although the hydrophilic nature of cellulose is essential for water vapor absorption, the sensing signal is often transduced by conductive components like MXene, GO, or CNTs.

**Figure 49:**
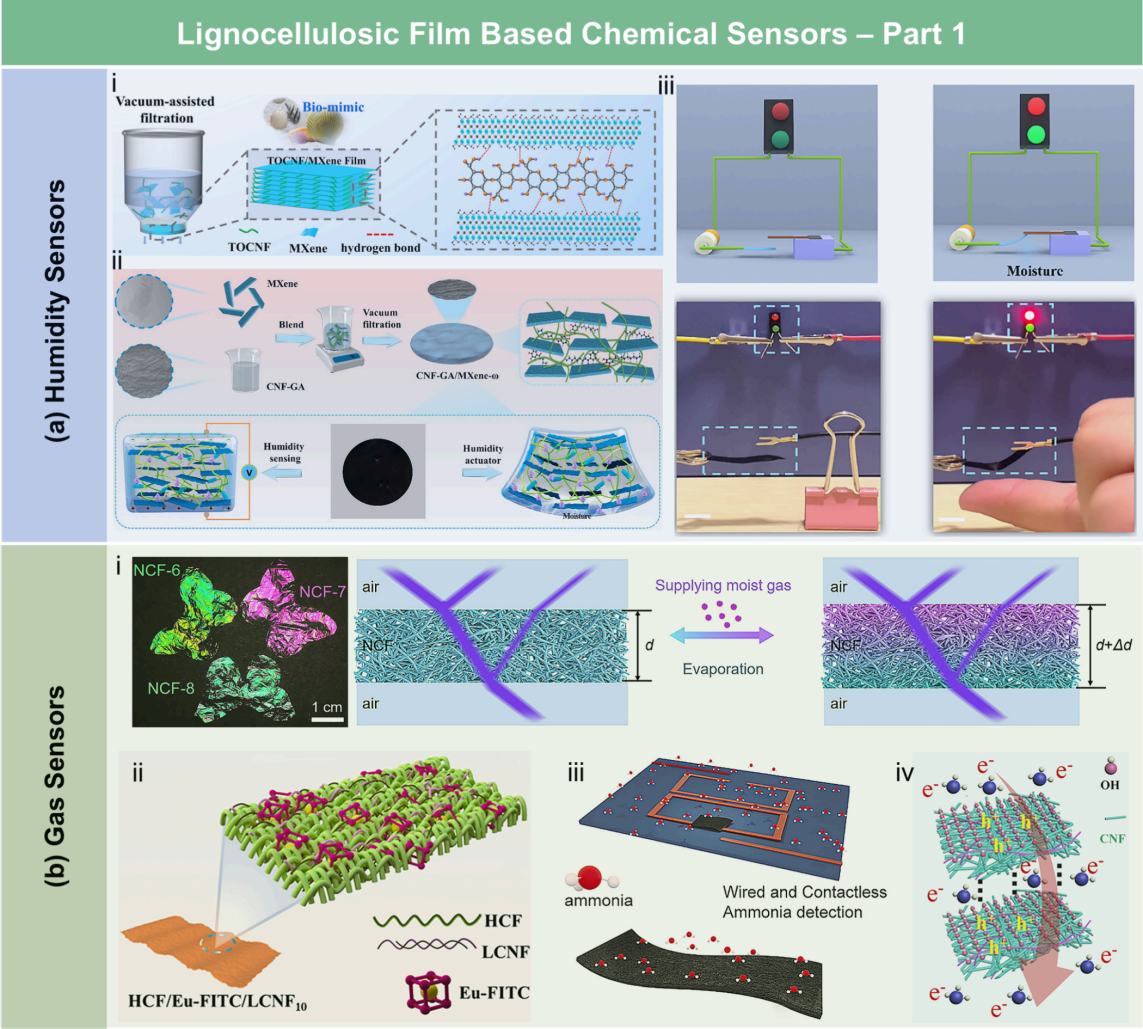
Application of LCFs in chemical sensors. (a) Schematic of the preparation of TOCNF/MXene composite film by a vacuum-assisted filtration strategy (i). Adapted with permission from ref 966. Copyright 2022 Elsevier. Schematic illustration of the strategy for constructing CNF-GA/MXene nanocomposite films (ii). Adapted with permission from ref 967. Copyright 2024 Elsevier under CC BY 4.0 (https://creativecommons.org/licenses/by/4.0/). Schematic illustration and photographs of the CNF/GO/CNT films based smart electric switch to turn on/off the circuit responding to humidity changes (iii). Adapted with permission from ref 969. Copyright 2021 American Chemical Society. (b) Photograph of the iridescent NCFs and schematic illustration of the changing thickness of an NCF during exposure to moist gas on its surface, resulting in the color change (i). Adapted with permission from ref 973. Copyright 2022 Elsevier. HCF/Eu-FITC/LCNF films for NH_3_ sensor (ii). Adapted with permission from ref 974. Copyright 2024 Elsevier. LCNF film for NH_3_ sensor (iii). Adapted with permission from ref 975. Copyright 2021 Elsevier. Schematic illustration of the NH_3_-sensing mechanism for CNF/PEDOT:PSS film-based sensors (iv). Adapted with permission from ref 976. Copyright 2023 American Chemical Society.

Gas sensors utilizing LCFs take advantage of the porous structure and functional groups of lignocellulosic materials, which can be engineered to interact with various gas molecules, such as nitrogen dioxide, ammonia, or carbon dioxide. By integrating conductive materials like metal oxides, CNTs, or graphene into LCFs, the sensitivity of the films to gas adsorption and subsequent electrical changes can be significantly enhanced. In these systems, cellulose frequently serves as a mechanically robust and flexible host for dispersing sensing materials, with limited direct participation in signal generation. For example, for NO_2_ detection, eco-friendly cellulose films combined with CVD-grown graphene, enhanced by halloysite nanotubes to increase surface area, demonstrated significant current changes at low NO_2_ concentrations.^977^ This combination makes these materials highly effective for real-time NO_2_ monitoring. Shifting to NH_3_ detection, CNF functionalized with HAP nanorods exhibited impressive performance with a sensitivity factor of 893% and a response time of 120 s, making them suitable for industrial gas monitoring.^978^ Similarly, lignocellulosic sensors based on rGO and PANI composites showed a 10-fold improvement in ammonia sensing, with a detection limit as low as 1 ppm and stable performance under moist conditions (Figure 49biii).^975^ Another approach, using cellulose nanofibers interwoven with PEDOT:PSS, achieved high ammonia sensitivity at 20.2%/ppm, with rapid response and recovery times of 4.8/4.0 s, further enhancing the applicability of these materials for detecting NH_3_ (Figure 49biv).^976^ For H_2_S sensing, CA nanofibers mixed with PPy and tungsten oxide (WO_3_) nanoparticles have shown high sensitivity at room temperature, detecting H_2_S concentrations as low as 1 ppm with a fast response time of 22.8 s, making them ideal for industrial environments where real-time detection of toxic gases is critical.^979^ Additionally, biogenic amine detection, particularly for food freshness monitoring, has been effectively achieved using LCNF composites (Figure 49bii). These sensors demonstrated a significant color change (Δ*E* = 113.11) at a detection limit of 1.83 ppm, offering a practical solution for real-time monitoring of food spoilage through the detection of biogenic amines.^974^ Moreover, transparent NFC-based bionanocomposite films have been developed for visual gas detection, providing an ultrafast response time of less than 1 s through colorimetric changes (Figure 49bi).^973^ This innovative approach allows for real-time, visual monitoring of gases in various environmental settings.

##### 5.5.2.2 Active in Recognition, Transduction, or Signal Generation

Beyond passive roles, certain cellulose-based systems actively contribute to the sensing process, particularly via optical, structural, or electronic changes derived from their intrinsic molecular configuration or surface chemistry.

A conceptually significant example is the work of MacLachlan et al. at the University of British Columbia, who developed chiral nematic CNC films capable of sensing volatile organic compounds.^980,981^ These films exhibit vivid, reversible color changes resulting from analyte-induced pitch modulation in the helicoidal structure. This direct structural response to vapor exposure, without the need for conductive additives, demonstrates CNCs’ active role in signal transduction and remains a foundational reference for optical cellulose-based sensors.

LCF-based pH sensors exploit the inherent reactivity of the hydroxyl and carboxyl groups in lignocellulosic materials to detect changes in the concentration of hydrogen ions. These functional groups can undergo protonation or deprotonation depending on the pH of the surrounding environment, leading to measurable changes in electrical conductivity or colorimetric signals. By incorporating additional pH-responsive materials or dyes, LCFs can offer precise and sensitive detection across a wide pH range, making them suitable for environmental and biomedical applications. Chen et al.^982^ fabricated chiral nematic CNC films, combined with CS and deacetylated chitin nanofibers (Figure 50ai). These films exhibited a wide-range pH response with visible colorimetric changes, offering high sensitivity and excellent reversibility, making them suitable for real-time pH detection. Similarly, Ding et al.^983^ developed a PVA/cellulose-based pH sensor modified with acidochromic dye, which demonstrated a distinct color change from yellow to brick-red and purple at pH values of 7, 10, and 12, respectively (Figure 50aii). This visual sensor was effectively used for monitoring shrimp spoilage by detecting pH shifts during decomposition. Moreover, Si et al.^622^ designed a lignin-derived carbon dot/cellulose nanofiber (CNF) composite film that exhibited a sensitive and linear pH response, useful for real-time food freshness monitoring (Figure 50aiii). The composite film’s fluorescence intensity changed based on pH variations, providing a promising strategy for portable, smartphone-integrated visual pH monitoring. Dong et al.^984^ introduced a superhydrophobic RC colorimetric film, inspired by the Canna plant, which displayed strong pH sensitivity and real-time food freshness monitoring capabilities, particularly under extreme conditions like freezing, thanks to its icephobic properties (Figure 50aiv). Together, these studies demonstrate the versatility and effectiveness of LCFs for pH sensing, offering real-time, visual, and portable solutions across various applications, including food freshness monitoring and intelligent packaging.

**Figure 50:**
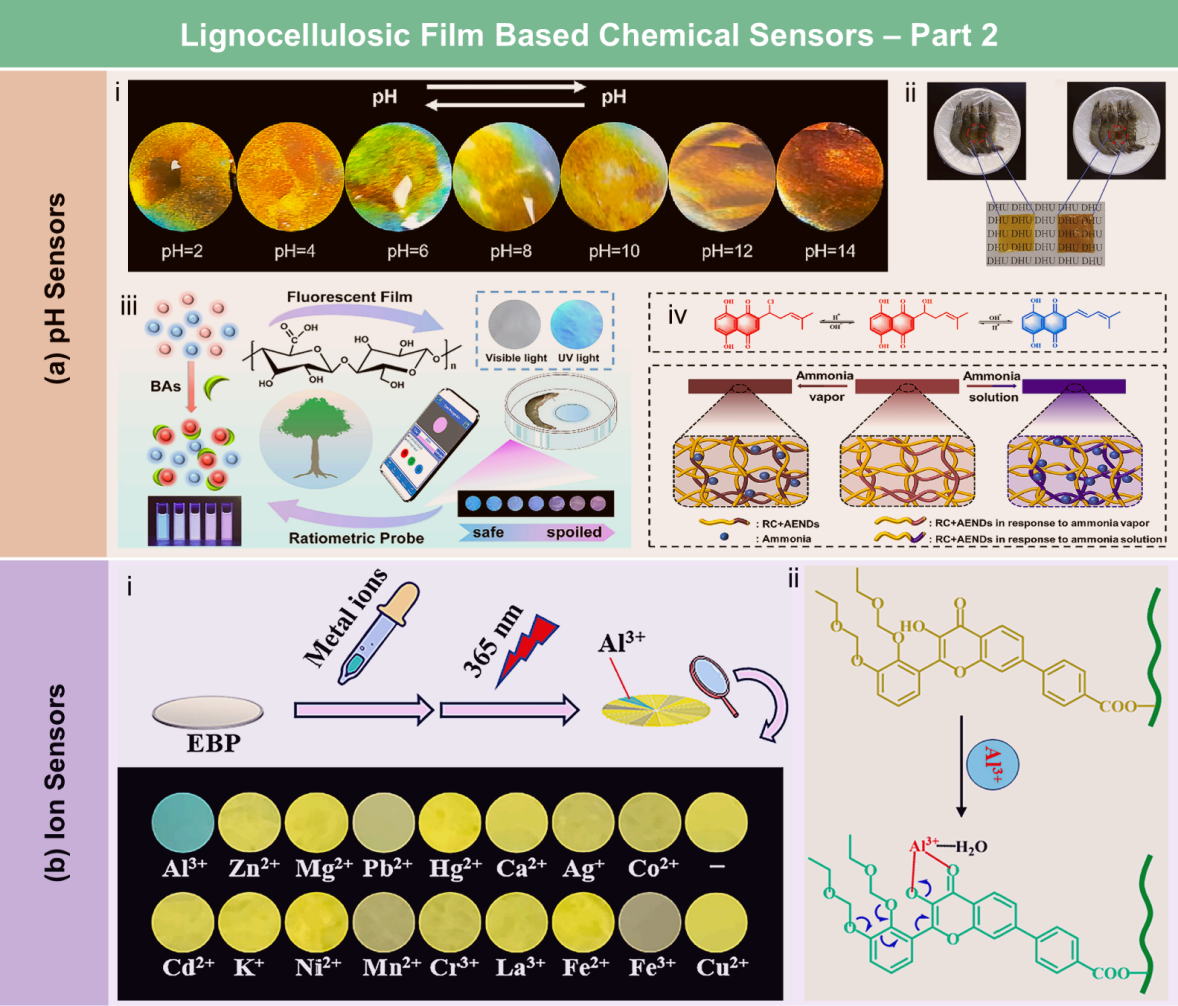
(a) Optical iridescence of CS-4 responsive to wide range of pH changes (i). Adapted with permission from ref 982. Copyright 2023 Elsevier. NH_3_ sensor based on PVA/ARC film for shrimp freshness detection (ii). Adapted with permission from ref 983. Copyright 2020 Elsevier. CDs/CNFs films-based sensor for real-time and visual food freshness monitoring (iii). Adapted with permission from ref 622. Copyright 2022 American Chemical Society. The schematic illustration of AENDs solution under acid–base condition. The schematic mechanism of the colorimetric film response of ammonia vapor and ammonia solution (iv). Adapted with permission from ref 984. Copyright 2023 Elsevier. (b) Photographs of EBP before and after binding to various metal ions under UV light (i) and the proposed detection mechanism for Al^3+^ (ii). Adapted with permission from ref 985. Copyright 2023 Elsevier.

For ion sensing applications, various LCF-based sensors have been developed to detect different types of ions, with impressive sensitivity and rapid response. Starting with lead ion (Pb^2+^) detection, Li et al.^986^ developed pyromellitic dianhydride-grafted deacetylated cellulose acetate (DCA-PMDA) films that enabled the simultaneous detection and removal of Pb^2+^ from water. The films exhibited a colorimetric response, changing from white to dark yellow-brown upon exposure to Pb^2+^, with a naked-eye detection limit of 0.048 mM and an adsorption capacity of 326.8 mg·g^–1^, making it highly effective for environmental applications. For aluminum ion (Al^3+^) detection, an ethylcellulose-based flavonol fluorescent sensor exhibited a remarkable fluorescence intensity increase (180-fold) upon binding with Al^3+^. This sensor, with a detection limit of 13.0 nM, was successfully applied in food samples and plant tissues, offering a broad pH range (4–10) and a fast response time of 3 min (Figure 50b).^985^ For copper ion (Cu^2+^) detection, a photoluminescent Eu-metal organic framework (MOF) integrated with TOCNF was developed. The sensor demonstrated excellent selectivity for Cu^2+^ in the presence of other metal ions, with a linear fluorescence decrease as Cu^2+^ concentration increased, making it an ideal candidate for detecting Cu^2+^ in water samples.^987^ In cadmium ion (Cd^2+^) sensing, a cellulose membrane stained with Victoria Blue B (VBB) enabled the rapid detection of Cd^2+^, showing a color change from yellow to blue-green with a limit of detection as low as 0.01 mg/L and a fast response time of 1 min.^988^ In another study, a colorimetric sensor based on CA films immobilized with dithizone (DTZ) showed fast response and selective detection of Hg^2+^, Cu^2+^, and Zn^2+^, displaying distinctive color changes within 1 min, making it highly practical for on-site heavy metal ion detection.^989^ These studies highlight the versatility of lignocellulosic materials in ion detection, providing solutions for the detection of aluminum, copper, mercury, cadmium, and other heavy metal ions. The sensors not only offer high sensitivity and selectivity but also demonstrate fast response times, making them suitable for real-time environmental monitoring and food safety applications.

In addition to humidity, gas, pH, and ion sensing, LCFs can be engineered to detect a wide range of organic and inorganic chemicals. By introducing specific receptor molecules, such as molecularly imprinted polymers, LCFs can be designed to selectively bind to pollutants, toxins, or other organic molecules.^990–995^ These interactions result in detectable changes in the film’s electrical, optical, or mechanical properties, enabling the development of highly sensitive chemical sensors for environmental, industrial, and biomedical applications.

#### 5.5.3 Biosensors

Biosensors are analytical devices that combine a biological recognition element with a signal transduction mechanism to detect analytes such as proteins, enzymes, nucleic acids, and small molecules. LCFs, especially those based on nanocellulose, offer unique advantages for biosensors including high surface area, abundant functional groups for bioconjugation, and the ability to stabilize enzymes. Unlike synthetic polymers, LCFs provide inherent biocompatibility and hierarchical porous structures that facilitate mass transport and biomolecule accessibility. In this section, we categorize representative LCF-based biosensors by analyte type and extract key design principles such as enzyme immobilization, signal amplification, and structural support roles.

Glucose sensors are the most widely developed due to their importance in diabetes management. LCFs such as CA, RC, and nanocellulose serve as effective supports for enzyme immobilization, while enhancing signal stability and reducing nonspecific adsorption. For example, a cellulose-ZnO hybrid film demonstrated a conductometric response for glucose detection in the range of 1–12 mM, with glucose oxidase (GOx) immobilized on the hybrid material (Figure 51ai).^996^ Additionally, an optical fiber glucose biosensor using CA and CQDs achieved real-time glucose detection with a detection limit of 6.43 μM.^997^ Gold nanorod-CA composites also improved glucose biosensing with a detection limit of 0.02 mM and sensitivity of 8.4 μA·cm^–2^·mM^–1^.^998^ Further, a cellulose-tin oxide nanocomposite demonstrated a linear response for glucose detection between 0.5–12 mM, suitable for clinical monitoring (Figure 51aii).^999^ Moreover, an electrospun CA nanofiber biosensor with rGO achieved impedimetric glucose sensing with high sensitivity and a detection limit of 0.1 mM.^1000^

**Figure 51:**
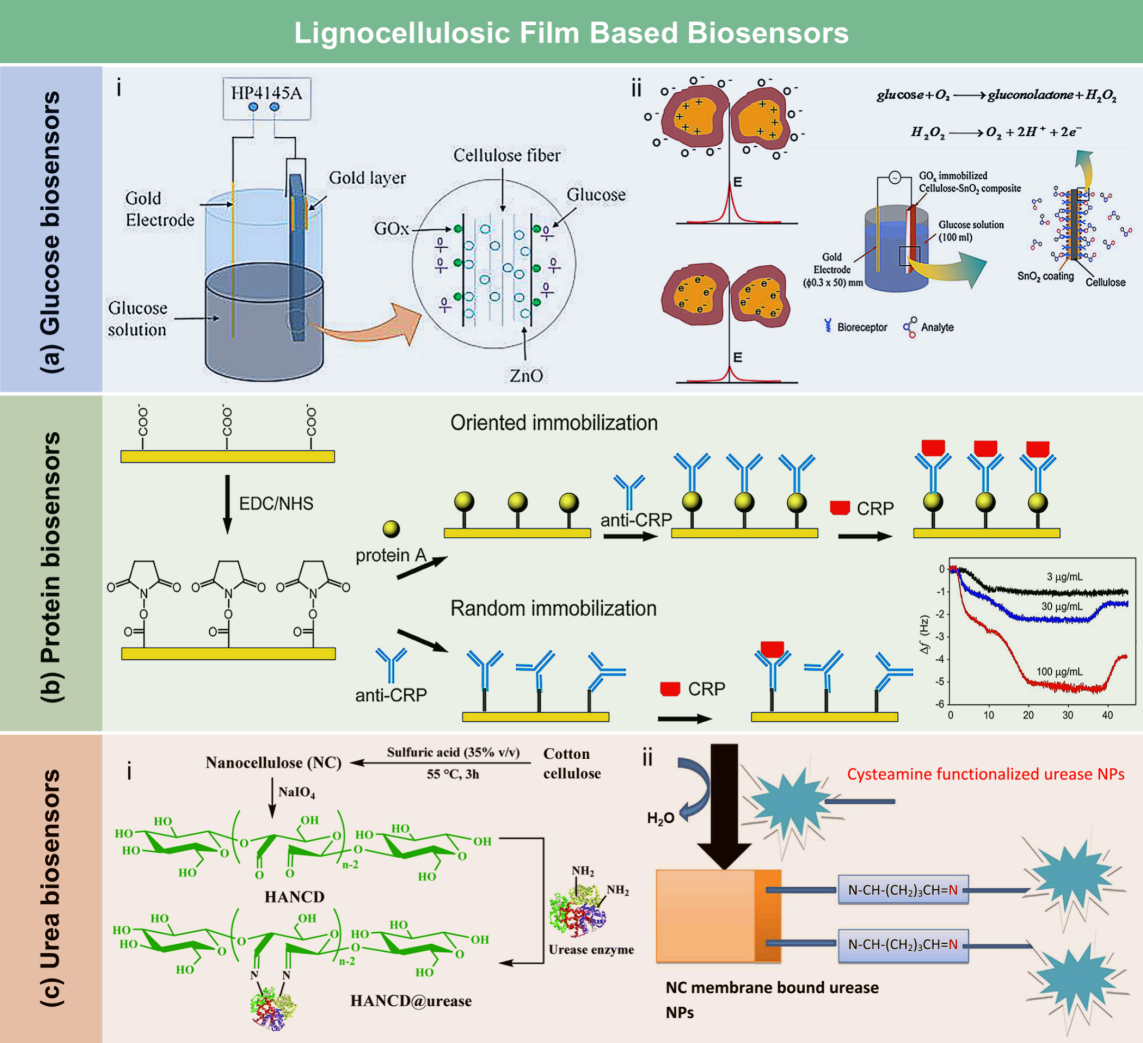
Application of LCFs in biosensors. (a) Schematic diagram of the glucose biosensor based on the cellulose ZnO hybrid film (i). Adapted with permission from ref 996. Copyright 2015 Taylor and Francis. Schematic representation of the detection mechanism of cellulose-SnO_2_ hybrid nanocomposite glucose biosensor (ii). Adapted with permission from ref 999. Copyright 2011 Elsevier. (b) Immobilization of anti-CRP on carboxylated CNF surfaces (tCNF or cCNF) via EDC/NHS coupling for CRP detection. Adapted with permission from ref 1001. Copyright 2016 American Chemical Society. (c) Schematic diagram of urease covalently immobilized on cotton-derived high-content nanocellulose-dialdehyde (HANCD) (i). Adapted with permission from ref 1002. Copyright 2019 Royal Society of Chemistry under CC BY 3.0 (https://creativecommons.org/licenses/by/3.0/). Schematic representation of steps/chemical reactions involved in the fabrication of urease NPs/NC film (ii). Adapted with permission from ref 1003. Copyright 2017 Elsevier.

Protein biosensors are essential for detecting biomarkers related to diseases such as cardiovascular conditions. Carboxylated CNF-based immunosensors were developed for detecting C-reactive protein (CRP), an important marker for inflammation and cardiovascular disease. These immunosensors demonstrated high specificity and sensitivity, proving effective in early disease detection (Figure 51b).^1001^

Urea biosensors based on cellulose nanomaterials exploit their abundant functional groups and high surface area for efficient enzyme immobilization. For example, urease was covalently immobilized onto cotton-derived high-content nanocellulose-dialdehyde (HANCD), forming a stable biocomposite film for urea detection (Figure 51ci).^1002^ The resulting urease/HANCD sensor exhibited excellent catalytic activity and strong binding stability, enabling rapid and sensitive electrochemical response toward urea. In another study, urease nanoparticles (NPs) were immobilized onto a nitrocellulose membrane to create a potentiometric urea biosensor. This sensor showed a rapid response time of 10–42 s, with an optimal working range of 2 to 80 μM and a detection limit of 1 μM/L. It also demonstrated high precision, with minimal interference from other ions, making it ideal for serum urea testing in clinical diagnostics (Figure 51cii).^1003^

Hydrogen peroxide (H_2_O_2_) detection is crucial in many biological and environmental contexts. A cellulose diacetate-ionic liquid biosensor immobilized myoglobin, enabling sensitive detection of H_2_O_2_ with a range of 5.0–100 μM and a detection limit of 2.0 μM.^1004^ Similarly, CNF-carbon dot nanopapers offered colorimetric detection of H_2_O_2_ with a smartphone-based method, achieving a detection limit of 0.93 μM, providing a reusable and practical sensing tool.^1005^

Xanthine biosensors for food spoilage monitoring benefit from nanocellulose’s high permeability and enzyme-supporting surface. A nanocellulose-based electrochemical sensor detected xanthine over 3–50 μM with a 47.96 nM detection limit, enabling real-time fish freshness assessment.^1006^

In summary, LCFs, especially nanocellulose, contribute to biosensing beyond passive support roles by enhancing enzyme stability, facilitating electron transport, and offering tunable surface chemistry. Their biocompatibility, nanoscale morphology, and chemical modifiability distinguish cellulose from other polymers and justify its strategic use in biosensor design. Future efforts should emphasize integrating LCFs with advanced biorecognition and transduction mechanisms to create more sensitive, selective, and multifunctional biosensors.

### 5.6 Water Treatment

It is known that lignocellulose is a composite material that consists of the following primary components: cellulose, hemicellulose, and lignin, where cellulose is the fundamental building block of lignocellulose. Cellulose, cellulose derivative, and nanocellulose-based membranes usually exclude the hemicellulose and lignin components from lignocellulose.

Cellulose-based membranes, derived from natural cellulose and its derivatives (e.g., cellulose acetate), have played an important role in water purification since the mid-20th century. These membranes are broadly used in processes such as microfiltration (MF), ultrafiltration (UF), nanofiltration (NF), and reverse osmosis (RO) to remove particles, salts, and contaminants from water.^1007^ Early membrane filters made from cellulose esters, including cellulose nitrate and cellulose acetate, were used for laboratory sterilization and virus/bacteria removal in the 1930s.^1008^ However, these early microporous filters had low water flux, limiting their practical use in large-scale water treatment. The development of high-performance asymmetric cellulose membranes in the late 1950s marked the breakthrough concept that enabled modern desalination and filtration technology.^1009^ Specifically, when Sidney Loeb and Srinivasa Sourirajan at UCLA developed the anisotropic (asymmetric) cellulose acetate (CA) membranes using a phase inversion process in 1960, this breakthrough technology came to realization. The typical Loeb-Sourirajan membranes could achieve ∼99% salt rejection with a water permeability on the order of 0.005 m^3^/m^2^·day·atm, about five times higher flux than previous cellulose filters.^1010–1012^ However, they also had limitations: cellulose acetate is prone to hydrolysis in extremes of pH (stable only in pH ∼4–6) and can be vulnerable to biological attack, thus limiting the membrane lifespan. Over time, CA formulations were improved, e.g. using cellulose triacetate for better stability and higher flux, or blending additives like acetylated polymers and plasticizers to enhance performance. Other cellulose derivatives were also employed, e.g., cellulose nitrate membranes were commonly used for microfiltration (though the system can be brittle and less stable), and regenerated cellulose membranes (pure cellulose reconstructed from solvents) were used in ultrafiltration and dialysis for hydrophilicity. Each generation of cellulose membrane technology brought significant improvements in flux and selectivity, as well as antifouling properties.

Lignocellulose has been employed to fabricate cellulose-based composite membranes for various applications such as oily wastewater treatment, membrane filtration, water disinfection, and solar desalination.^1013–1016^ The advantages of cellulose-based composite membranes over their counterparts are as follows: (1) Functionalizability and robustness. The hydroxyl groups in cellulose can be converted into a variety of other functional groups, such as carboxyl and amino groups, which endow the cellulose-based membranes with multiple functions.^1017–1019^ Additionally, the cellulose membrane itself, especially the native cellulose-based membrane, exhibits excellent thermal, mechanical, and physical properties due to its high crystallinity.^1013,1020^ (2) Hydrophilicity and water insolubility. It is interesting to note that cellulose exhibits superhydrophilicity, which offers antifouling properties to the membrane in water treatment.^1021^ However, the membrane retains good stability in water, making it ideally suitable for water filtration applications. (3) Biodegradability and chemical resistance. Cellulose is a well-known sustainable and biodegradable material on Earth. The strong hydrogen bonds between cellulose chains also provide the cellulose-based membrane with unique chemical resistance.^1022^ However, cellulose cannot be dissolved by typical organic solvents, thus, cellulose’s high chemical resistance sometimes limits its processability. The unique properties of cellulose offer a wide range of applications for cellulose-based membranes.

#### 5.6.1 Oil/Water Separation

Oily wastewater treatment is an important application in water treatment industries, including municipal wastewater, bilge water, and food industrial wastewater.^1023^ The disposal criteria for wastewater into the environment, as set forth by the EPA, is usually less than 10 ppm, with the aim of effectively protecting the environment.^1024^ The major challenges of oily wastewater treatment are two. (1) The broad size distribution of oil droplets. The typical size of oil droplets in oily wastewater ranges from a nanometer to a few microns, which requires the use of micro- and ultrafiltration membranes to remove oil droplets from contaminated wastewater at a high rejection ratio based on the separation mechanism of size exclusion. (2) Demand for antifouling properties. Fouling by organics and inorganics is a significant issue in all filtration processes, from microfiltration to RO desalination, with oily wastewater being particularly challenging in an ultrafiltration process.^1025–1029^ The permeation flux will decline when fouling occurs, where the membrane cartridges must be cleaned frequently to maintain a high permeation flux. Consequently, additional cleaning costs are often incurred for the filtration treatment, where a greater robustness is also required for the filtration membrane.

Conventional ultrafiltration membranes used for oily wastewater treatment are made from materials, such as PAN, PSF, PVDF, and RC hydrolyzed from cellulose esters.^1030–1032^ These materials can meet the material requirements, such as robustness and cost-effectiveness for practical membrane applications. However, these materials also have some apparent disadvantages. For example, the relatively hydrophobic surface of these materials can lead to membrane fouling issues with oil droplets or proteins.^1033,1034^ Additionally, the presence of dead-end pore structure and lower porosity (∼50%) within the membrane can greatly increase hydraulic resistance and reduce water permeability. Ideally, a composite membrane with a highly hydrophilic surface, high porosity, and interconnected pores is desired to achieve high filtration efficiency in terms of permeation flux, rejection ratio, and antifouling properties.^1022^

A thin-film nanofibrous composite membrane, consisting of three layers with different composition and/or fiber diameter in each layer, has been demonstrated to possess high filtration efficiency in terms of permeation flux, rejection ratio, and long-term usage against oily wastewater.^1035^ In this system, the bottom layer consists of a nonwoven substrate (e.g., PET nonwovens), which primarily provides the necessary mechanical properties for the membrane. The tensile strength of the PET substrate, as reported in the literature, is typically about 40 MPa.^1036^ The middle and top layers of the composite membrane demonstrate two key structural design innovations. The middle layer, deposited on the PET substrate, is a nonwoven nanofibrous mat typically prepared using electrospinning technology.^1037^ This highly porous layer offers three advantages to the membrane: (1) high porosity, typically 80% and higher, which reduces hydraulic resistance and affords the membrane a high permeation flux; (2) adjustable pore size, which ensures a high rejection ratio against various targets; and (3) an interconnected pore structure that is beneficial for high permeation flux and reduction in irreversible fouling. The second innovation is the top barrier layer, integrated with the middle porous support, which is composed of highly hydrophilic materials, including cross-linked PVA, CS, PEBAX, as well as cellulose and nanocellulose.^1038–1042^ The hydrophilic top barrier layer with low surface tension can reduce the fouling tendency, while the ultrathin middle layer (e.g., 100 to 500 nm) offers high permeation flux to the membrane. A representation of the nanofibrous composite membrane is shown in Figure 52ai.

**Figure 52:**
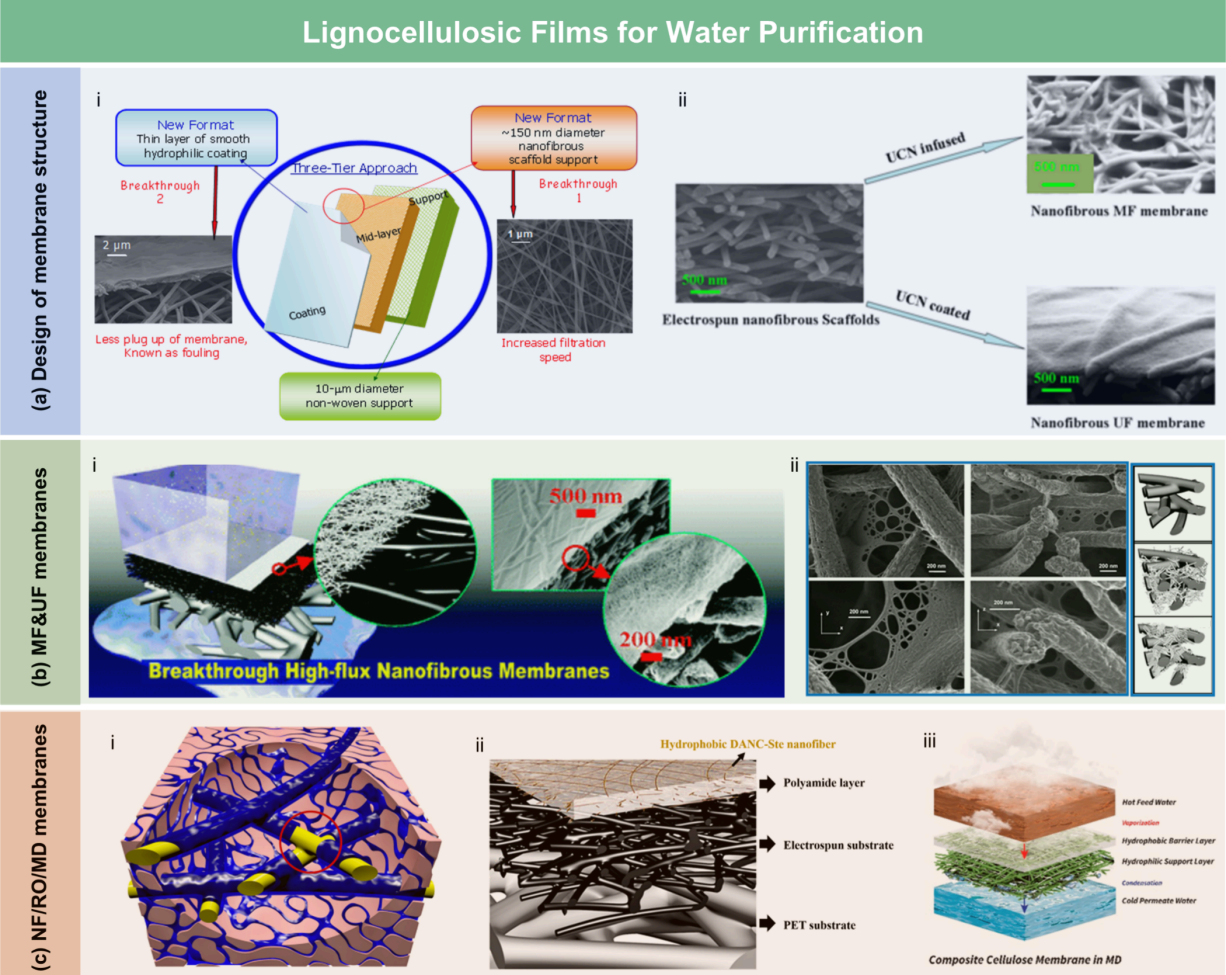
(a) The representative structure of a thin-film nanofibrous composite membrane (i). Adapted with permission from ref 1043. Copyright 2009 Wiley Periodicals, Inc. Nanocellulose composite membranes fabricated by different integration approaches: infusion of nanocellulose into electrospun scaffold (MF membrane), and coating of nanocellulose on the electrospun scaffold (UF membrane) (ii). Adapted with permission from ref 1044. Copyright 2011 Royal Society of Chemistry. (b) Highly permeable nanocellulose composite membrane for oily wastewater treatment (i). Adapted with permission from ref 1045. Copyright 2011 American Chemical Society. SEM images and schematic diagrams of nanocellulose infused MF membranes (ii). Adapted with permission from ref 1046. Copyright 2011 Oxford Academic. (c) Schematic representation of water channels in the nanocomposite barrier layer: nanocellulose (yellow) defines three-dimensional networks where the gap (blue) between the nanocellulose and polymer matrix (pink) creates water channels; the cut-out in the red circle exhibits the cross-linked nature of the nanocellulose interconnects (i). Adapted with permission from ref 1047. Copyright 2012 American Chemical Society. Nanocellulose-based thin-film nanofibrous nanocomposite RO membrane containing water channels (ii). Adapted with permission from ref 1048. Copyright 2023 American Chemical Society. Schematic preparation of composite cellulose membrane in membrane distillation for desalination (iii). Adapted with permission from ref 1049. Copyright 2022 American Chemical Society.

Cellulose turns out to be an excellent material that can serve as the barrier layer in the thin-film nanofibrous composite (TFNC) membrane for oily wastewater treatment, achieving high permeation flux, high rejection ratio, and antifouling properties.^1050^ There are two approaches by which cellulose can be coated onto a porous substrate as the barrier layer. The first approach involves the use of cellulose solution to create the barrier layer with RC thin film.^12^ It is well-known that native cellulose cannot be dissolved in typical organic solvents or water due to the strong hydrogen bonding between hydroxyl groups and van der Waal forces between the cellulose chain in the cellulose crystal. To dissolve crystalline cellulose structure, some ion-natural solvents, such as ionic liquids, NMMO, and DMAc/LiCl need to be used.^1051,1052^ In one study, cellulose/ionic liquid solutions were used as the coating solution to create RC-based membrane.^1040^ Specifically, two ionic liquids, BMIMCl and 1-ethyl-3-methylimidazolium acetate, were chosen to dissolve lignocellulose and chitin, to prepare two types of composite ultrafiltration membranes having RC and chitin barrier layers, respectively, for oily wastewater treatment. These cellulose-based membranes exhibited remarkably high filtration efficiency compared to commercially available counterparts in terms of permeation flux, rejection ratio, and antifouling properties during the filtration process.^1024^ For example, the permeation flux of a representative cellulose-based composite membrane was 2–10 times higher than that of commercial membranes PAN10 and PAN400, and it achieved a 99.5%-higher rejection ratio against 1,350 ppm oily wastewater (a model of bilge water for the US Navy) during a 48 to 240 h long-term filtration. The residual oil in the wastewater met the disposal criteria for wastewater as set by the EPA. The thin barrier layer of 300–500 nm and the hydrophilic properties of the RC contributed to the high permeation flux, high rejection ratio, and antifouling advantages of the membranes.

The other approach involved the use of native cellulose nanoparticles in suspension to prepare the barrier layer of the membrane.^1045^ In this case, nanocellulose (e.g., CNFs and CNCs) was first dispersed in water and used as a coating suspension for membrane fabrication. Typically, nanocellulose is prepared by TEMPO-mediated oxidation, nitro-oxidation, or acid-hydrolysis method followed by mild mechanical treatment.^1053–1055^ The fabrication mechanism of nanocellulose relies mainly on the functionalization (e.g., oxidation and sulfonation) of the C6-hydroxymethyl group, which is converted to the carboxylate group during the functionalization process. The resulting negative charges enable nanocellulose to maintain stable dispersity in aqueous solutions due to the repulsion of negative charges. Moreover, the fiber diameter is typically in the range of 5–10 nm, with a length-to-diameter ratio greater than 40, resulting in a relatively low overlap concentration. Consequently, the viscosity of the well-dispersed nanocellulose suspension in water is quite high, at concentrations below 0.2 wt %. As a result, the barrier layer of the nanocellulose can be as thin as 100 nm when coated or sprayed onto the substrate.^1038,1056^ Additionally, the carboxylate groups located on the surface of nanocellulose impart superhydrophilicity to the membrane barrier, combined with its negatively charged nature, providing impressive antifouling properties against oil droplets to the membrane.^1057^ Therefore, nanocellulose-based membranes exhibited advantages over classical cellulose-based membranes, such as tailored pores, a highly hydrophilic and charged surface, and water channels formed in the barrier layer.

The unique format of nanocellulose-based thin-film nanofibrous composite (TFNC) membrane has been demonstrated for purification of oily wastewater.^1045^ This membrane was designed with a three-tiered structure, as shown in Figure 52bi. In such TFNC membranes, nanocellulose or RC was used as the barrier layer. Nanocellulose possesses the unique properties of native cellulose, such as high crystallinity, high thermal stability, and high mechanical properties. Moreover, the thinner barrier layer and superhydrophilic nature are beneficial for antifouling properties. As a result, nanocellulose-based TFNC membrane exhibited 10 times higher permeation flux while maintaining the same rejection ratio of 99.5% when compared to commercially available counterparts, such as PAN10 and PAN400 ultrafiltration membranes, in a 120 h continuous filtration demonstration. The spiral wound UF cartridge based on nanocellulose-based TFNC membrane exhibited a 4-times higher permeation flux and a comparable rejection ratio against oily wastewater, compared to the commercial ceramic filtration cartridge.^1058^ These membranes appeared to be robust against oily wastewater, containing oxidants, reductants, a wide range of pH values, and even bacteria.

It is worth mentioning that if the oily wastewater is stable in the emulsified form, it can be purified by UF filtration.^1022^ In the case of unstable mixtures of oil and water, they can be separated by adsorption.^1059,1060^ The unstable mixture of oil and water is typically in the form of oil droplets suspended in water, and over time, these oil droplets can aggregate together. The size of the oil droplets is typically greater than microns and can even reach millimeter size. The separation mechanism is based on the affinity of oil droplets for hydrophobic surfaces and the high porous structure of the adsorbents. As discussed previously, one major advantage of cellulose is its functionalizability, where the cellulose surface is naturally hydrophilic but can be readily converted to hydrophobic. Hydrophobic nanocellulose materials can be used as absorbents to separate the oil and water mixtures. Additionally, cellulose-based materials can be easily processed into high porous structures, for example, through freeze-drying, phase separation, solvent evaporation, fiber manufacturing, and natural generation.

Recently, a hydrophobic luffa sponge has been demonstrated for the separation of oils with different viscosities from water.^1061^ The hydrophobicity was derived by the surface modification of luffa (a natural cellulose scaffold) with POSS nanoparticles. The superhydrophobic nature of the modified luffa sponge exhibited an 8 to 12-times higher absorption capacity for the removal of oil from water, depending on the porosity of the sponge and the viscosity of the oil. Similarly, hydrophilic nanocellulose can be converted to hydrophobic using a similar approach. Additionally, the surface of nanocellulose can also be modified by esterification, etherification, grafting polymerization, and carbonization to be converted into hydrophobic for oil and water separation.^549^ The high separation efficiency can be achieved with superhydrophobicity and high surface area.

#### 5.6.2 Membrane Filtration

Cellulose adopted in membrane filtration applications is mainly present in two formats: (i) RC, typically used in microfiltration and ultrafiltration; and (ii) nanocellulose, typically used in ultrafiltration, nanofiltration and RO desalination.^1022^ The major challenge in the membrane filtration efficiency involves balancing the trade-off between permeation flux and rejection ratio, especially for nanofiltration and RO operations that can achieve high permeation flux and maintain high rejection ratio simultaneously.^1062^

Conventional cellulose-based microfiltration membranes are fabricated by the phase inversion method, using the mixtures of nitro-cellulose and cellulose esters, to produce porous membranes with pore sizes ranging from 0.05 to 10 μm.^1063^ Such membranes are commercially available from the Millipore company (Massachusetts, USA), which are extensively used for separation of nanoparticles from water. Other commercially available cellulose-based UF membranes include membranes made from hydrolyzed CA, where the pore size and distribution of the membrane can be controlled by the degree of hydrolysis.^1064^ These membranes are primarily used for the separation of proteins. Additionally, RC-based composite UF membranes have been demonstrated for oily wastewater treatment (Section 5.6.1), where the barrier layer in these membranes is fabricated from cellulose solution and regenerated through the phase separation approach.^1022^ The high permeation flux, high rejection ratio, and low fouling tendency of cellulose-based composite membranes can provide a new pathway for the development of next generation membranes with high sustainability for water purification.

An additional approach, using nanocellulose was employed to fabricate a composite barrier layer to create a new class of TFNC nanofiltration (NF) and RO membranes.^1044^ The unique feature of these membranes includes the simultaneous improvement of permeation flux and rejection ratio. Specifically, in this new class of membrane, the concept of “directed water channels” was introduced by integration of nanocellulose scaffold with polymer matrix to form a composite barrier layer on the membrane, as shown in Figure 52ci.

In Figure 52ci, nanocellulose fibers were dispersed in the polymer (e.g., cross-linked polyamide) matrix, creating a three-dimensional nanofibrous network structure in the barrier layer. The “gap” between the surface of nanocellulose and the polymer matrix, formed by naturally occurring interface, served as “water channels” for transport of water molecules.^1065^ The existence of water channels can reduce the hydraulic resistance and shorten the pathway of water passage, thus promoting the water permeability of the barrier layer. Meanwhile, the size of water channels could be controlled and adjusted by the surface modification of nanocellulose, enabling selective passage of target molecules. As a result, the concept of water channels can offer the possibility of increasing the permeation flux while retaining the rejection ratio.

In one study, nanocellulose prepared by TEMPO-mediated oxidation was employed to introduce water channels in PAN and PVA barrier matrices to form composite barrier layers.^1039,1057^ During the fabrication process, it is noted that the mixture of nanocellulose suspension and PVA aqueous solution should be compatible and form a homogeneous coating suspension to ensure the fabrication of a uniform barrier layer. In the case of mixing nanocellulose into PAN solution with organic solvent, such as dimethylformamide (DMF), the nanocellulose aqueous suspension should undergo a solvent-exchange process first to replace water with DMF. The PVA/nanocellulose and PAN/nanocellulose composite barrier layers were found to offer 2 to 5 times higher permeation flux while maintaining a high rejection ratio of 99.7% (against bovine serum albumin (BSA) and oil emulsions, respectively) than convention membranes having the barrier layer without nanocellulose additives. The enhanced filtration performance could be attributed to the nanocellulose-induced water channels in the barrier layer of the membrane. Moreover, the surface robustness and antifouling properties of the PAN/nanocellulose membrane were also significantly improved, due to the formation of a three-dimensional network structure in the barrier layer leading to better mechanical properties, and the incorporation of negatively charged nanocellulose leading to greater hydrophilicity.

Water channels can also be created by coating a nanocellulose scaffold on the electrospun nanofibrous substrate followed by interfacial polymerization of trimesoyl chloride (TMC) and piperazine (PIP) or m-phenylenediamine (MPD) monomers to form a composite polyamide barrier layer. In this barrier layer, the nanocellulose scaffold is dispersed in the cross-linked polyamide matrix forming a composite barrier layer. It is well-known that the formation of the polyamide matrix is initiated at the interface between the organic and the aqueous phase. Specifically, MPD is diffused from the aqueous phase and reacts with TMC in the organic phase during interfacial polymerization, where only partial integration of the nanocellulose scaffold (in the aqueous phase) can take place. This network structure will create directed water channels to enhance water transportation without sacrificing the rejection ratio. A study conducted in our group validated this hypothesis, where the RO membrane containing the nanocellulose-PA barrier layer showed a high rejection ratio of 99% against NaCl salts, where the membrane without nanocellulose in the barrier layer only exhibited a rejection ratio of 98%.^1066^

Nanocellulose can also be directly incorporated into the polyamide barrier layer by using a nanocellulose aqueous suspension containing MPD monomers in interfacial polymerization with TMC (in the organic phase). In this scenario, nanocellulose accumulated at the interface can also be partially incorporated in the barrier layer, leading to an increase in the permeation flux. A TFNC RO membrane fabricated by this manner showed a 50% increase in flux, when compared to the RO membrane without nanocellulose, while retaining a high rejection ratio of 96% against NaCl. However, beside the favorable mechanism of water channels, other possibility is that the accumulation of nanocellulose at the interface can retard the diffusion of MPD into the organic phase, resulting in a relatively thin barrier layer after interfacial polymerization.^1067^

In the above approach, it is important to control the position of the interface in polymerization, in which the growth of the polyamide layer can incorporate most nanocellulose scaffold. This problem has been addressed by introducing a hydrophobic nanocellulose scaffold on the substrate. In this scenario, the aqueous phase containing MPD is confined within the porous substrate, where the organic phase with TMC monomer can fill the hydrophobic nanocellulose scaffold during interfacial polymerization, as shown in Figure 52cii.^1048^ As a result, the polyamide layer can grow toward the nanocellulose layer, and the nanocellulose nanofibrous scaffold can be greatly integrated with the polyamide, creating a large content of water channels in the composite barrier layer. Such a composite structure has also been demonstrated by MD simulations. The RO membrane containing the above composite barriers exhibited a 3 to 5-times higher permeation flux and a comparable rejection ratio against NaCl even after a 120-h long-term desalination process when compared to those of the pristine membrane and commercially available counterparts.^1048^

#### 5.6.3 Water Disinfection

Current water disinfection technologies are effective for removal of chemical contaminants such as pharmaceuticals, pesticides, and industrial pollutants like dyes and heavy metal ions.^1068,1069^ New innovations in water disinfection solutions using functionalized cellulose scaffolds (including nanocellulose) for adsorption, chemical oxidation, and the degradation of organic and inorganic pollutants have been demonstrated as a sustainable and more environmentally friendly pathway.^1070–1072^ This is because the major challenge in the existing adsorption media is that their adsorption sites can become saturated by targeted contaminants and thus require frequent renewal. In contrast, the low-cost cellulose adsorbents can be used as one-time basis materials, and some spent cellulose scaffolds can even be repurposed for other applications. Therefore, high adsorption capacity and good selectivity are two primary targets for the modification of cellulose scaffolds in water disinfection.

The unique chemical structure in cellulose makes it easy to be functionalized, where the suitable surface functionality can enhance its adsorption of pollutants through electrostatic interaction, hydrogen bonding and hydrophobic interaction.^1013,1073^ In a recent study, a new cellulose-based porous membrane system has been fabricated by surface grafting polymerization of functional monomers containing carboxyl and amino groups.^591,1074^ The grafting approach utilized free radical polymerization initiated by cerium ammonium nitrate (CAN) and APS. In this system, the degree of grafting on the cellulose membrane approached to 2–3 mmol·g^–1^, resulting in good adsorption capacities from 0.3 to 1.0 mmol·g^–1^ against dyes and heavy metal ions (such as methylene blue (MB) and Pb­(II), Cr­(VI), Cu­(II), Cd­(II), etc.) removal.^1075^ In another study, cellulose fabrics were used to prepare sustainable membranes using oxidized cellulose fibers prepared by TEMPO-mediated oxidation, where these membranes exhibited impressive adsorption capacities of 0.39 and 0.24 mmol·g^–1^ against Pb­(II) and La­(III), respectively, in the static adsorption study.^1076,1077^ Additionally, adsorption cartridges based on spiral wound oxidized cellulose membranes were also demonstrated to remove methylene blue (MB) contaminated wastewater in a dynamic adsorption study, where tons of wastewater were treated. In this study, the cartridge exhibited a near 100% retention ratio to adsorb MB with, high permeation flux of 11.8 L·m^–2^·h^–1^ and low-pressure drop of 150 kPa.^1078^

Nanocellulose has been proven to be an excellent candidate for adsorption applications due to its high surface area and presence of abundant carboxylate, aldehyde and hydroxyl groups on the fiber surface (nanofibers or nanowhiskers).^1045,1079^ The carboxylate group can be readily converted into other functional groups such as amino groups and thiol groups to change the surface charge properties.^1080,1081^ To prepare carboxylated nanocellulose, the typical chemical treatments involve TEMPO-mediated oxidation,^160^ carboxymethylation,^1082^ nitro-oxidation,^1054^ and sodium periodate-oxidation methods.^1083^ The presence of anionic carboxylate groups on the nanocellulose surface can render the scaffold to remove cationic dye molecules, heavy metal ions and even some biomacromolecules from contaminated water.^1079,1084–1088^ To fabricate cationic nanocellulose, the approach of sodium periodate-oxidation followed by grafting with Girard’s T agent through a Schiff-base reaction has been demonstrated.^1089,1090^ Such membranes are effective for removal of negatively charged Cr­(VI) oxyanions (e.g., the adsorption capacity = 1.5 mmol·g^–1^) based on the charge interaction and coagulation mechanism.

Metal oxides nanoparticles, such as TiO_2_, can be created *in situ* in the nanocellulose scaffold. In one study, adsorbing the titanium oxysulfate precursor onto the carboxylated cellulose nanofiber (CNF) scaffold, followed by *in situ* growth of TiO_2_ crystals.^1091^ The resulting composite CNF-TiO_2_ scaffold can be used to degrade organic pollutants such as MB dye in water disinfection through (UV) light-induced oxidation. In these composite membranes, embedded TiO_2_ nanoparticles (with an average diameter of 3 to 4 nm) were firmly anchored on the nanocellulose surface and served as catalysts for the degradation of MB (removal efficiency >98%). In addition, the membrane also exhibited a high filtration efficiency with a 2-log reduction value (LRV) against *E. coli*.^1091^ In another composite membrane system, suspensions of CNCs were directly mixed with TiO_2_ nanoparticles, where the resulting nanocomposite membranes were tested to evaluate the adsorption and photoreduction capability against Cr­(VI) oxyanions from contaminated water.^1092^ It was found that this membrane system could also achieve about 96% of removal ratio. Additionally, the membranes showed 90% recyclability after 5 cycles of usage. Finally, a regenerated MFC and ZnO composite membrane system was demonstrated by an in situ fabrication method with alkaline solutions.^1093^ In this system flower-like ZnO crystals with high surface area were found to tightly anchor on the nanocellulose surface, exhibiting remarkable adsorption capacity of 58.9 mmol·g^–1^ against As­(V).

The above modified nanocellulose membranes can be used for simultaneous filtration and adsorption to remove contaminants from water. The filtration performance is determined by the average pore size of the barrier layer where the contaminant size is the dominant factor, while the adsorption performance is determined by the interactions between the contaminants and the scaffold while the contaminant size is no longer a dominant factor.^1044^ There are two approaches to prepare such nanocellulose membranes, as shown in Figure 52ai. In one approach, nanocellulose can be coated on the top of the substrate (e.g., an electrospun nanofibrous membrane), where the nanocellulose coating becomes a barrier layer to render the system into a UF membrane.^1045^ In another approach, nanocellulose can be infused (or penetrated) into a porous substrate (e.g., an electrospun nanofibrous membrane), and then cross-linked to create a stable three-dimensional nanoweb structure in the substrate scaffold. This format renders the system into an MF membrane with excellent adsorption properties. Specifically, the electrospun nanofiber scaffold can serve as a skeleton, and the nanocellulose nanoweb can provide adsorption sites to attract contaminants, as shown in Figure 52bii.^1046^ Such composite nanofibrous structure offers high performance (i.e., high permeation flux and low pressure drop) for microfiltration while maintaining good adsorption capability to remove much smaller size contaminants (e.g., molecules and ions). The demonstrated nanocellulose-based composite MF membrane exhibited a high permeation flux of 192 L·m^–2^·h^–1^, a low-pressure drop of 3.0 kPa, and high rejection ratios of 4 and 6 log reduction values (LRV) against *B. diminata* and *E. coli*, respectively. The unique structure in this membrane offers opportunities to treat multiple contaminants in wastewater as the system offers both filtration and adsorption functions. In another demonstrated composite MF membranes, the system exhibited a rejection ratio against 0.2-μm nanoparticles of 99.7% with a permeation flux of 59 L·m^–2^·h^–1^, as well as high adsorption capacities of 68.0 mg/g and 2-LRV against crystal violet and MS2, respectively.^1085^ Compared with conventional disinfection approaches such as ozonation and UV-irradiation, nanocellulose-containing MF membranes can also achieve high disinfection efficiency, where the secondary contaminants can also be avoided.^75^

The adsorption selectivity of adsorbents and adsorptive membranes depends mainly on the interactions between the adsorptive sites and the target contaminants, as well as the competing agents, but is less associated with the substrate itself, i.e., cellulose. Therefore, the adsorption selectivity relies on the comparative adsorption interactions between the target contaminants and the interferents.^1094^ Typical interferents are monovalent and divalent ions, which are commonly present in contaminated water. Thus, the electrostatic interaction-based adsorption can be significantly affected by these coexisting ions. However, the adsorption pathways through hydrophobic interactions, hydrogen bonding, ligand exchanging, and chemical reactions will be less affected by these coexisting ions. In a recent study, the cellulose scaffold containing lanthanum and zirconium (hydr)­oxide components exhibited excellent adsorption selectivity against P­(V).^1095^ It was found that the adsorption mechanism of this system is mainly based on ligand exchange, resulting in a high adsorption selectivity between 70 and 95% even under coexisting anions. In another study, a DAC-based membrane system exhibited good adsorption selectivity for hydrazine in the presence of different metal ions.^1096,1097^ The adsorption selectivity of this system was higher than 95.8% because the adsorption mechanism was due to the reaction between the aldehyde groups and amino groups instead of charge interaction. Finally, the adsorption of a carboxylated CA nanofibrous membrane system was found to be unaffected by different coexisting ions against Bismarck Brown Y probably because hydrogen bonding was the primary mechanism.^1073^

The post-treatment of the spent adsorptive membrane should be considered. To meet the requirements of sustainability and cost-effectiveness, the pollutants in the spent nanocellulose containing MF membrane system should be able to be collected by desorption, where the regenerated membrane should be able to reuse for at least 5 cycles.^1098^ Furthermore, typical desorption approaches, such as the use of acid, alkaline, salt, and organic solvents as desorption agent should be applicable. Of course, this will also depend on the desorption mechanism and the nature of the adsorptive scaffold.

In summary, cellulose-based adsorbents (i.e., membranes and scaffolds) have achieved significant breakthroughs, including high adsorption capacity, high selectivity, reusability, and sustainability, when compared to conventional adsorbents. However, several challenges remain to be addressed for specific applications, such as poor acid/base tolerance, limited mechanical stability, and restricted adsorption capacity.

#### 5.6.4 Solar Powered Desalination

Solar powered desalination based on energy harvesting and membrane distillation techniques has attracted a great deal of attention due to the low capital requirement of the system, versatility and energy-saving potential in operation when compared with conventional desalination processes such as RO and thermal distillation.^1099–1101^ However, one current challenge to advancing solar desalination is the development of sustainable photothermal and membrane distillation (MD) materials that can lower the system cost, ease the optimization of heat management and enhance the evaporation rate. Specifically, there is a need to demonstrate sustainable, lower cost and functional membrane materials that can lead to interference-resistant, rapidly responsive, and all-weather desalination operational conditions. The ideal MD membrane characteristics thus should include (1) superhydrophobicity, but with water channels in the membrane that can enhance water vapor transportation efficiency, (2) antifouling properties on the membrane side against the feed solution, (3) good heat transfer efficiency of the membrane, and (4) environmental friendliness and cost effectiveness of the membrane.

Recently, several studies of using cellulose-based nanocomposite membranes for solar desalination has offered some desired characteristics listed above, that is the cost effectiveness, environmental friendliness and simultaneous possession of water passage within the membrane body and superhydrophobicity on the membrane surface.^1102–1104^ The conversion of hydrophilic cellulose into superhydrophobic surface can be readily achieved by suitable modification of cellulose chains. In one study, pristine natural wood was cut along the transverse direction or longitudinal direction and then underwent surface carbonization at 500 °C or was decorated with fine metal nanoparticles to create a bilayer structure.^1105–1107^ In this structure, the top layer (∼3 mm) containing open wood channels was made hydrophobic to promote water vapor passage, while the bottom layer containing wood channels served as water pumps for rapid water extraction and transport. This tree-inspired device opens a new pathway for developing a highly efficient solar steam device. The natural wood channels of the wood membrane can serve as a highway for water vapor and liquid transportation, while the engineered surface layer can harvest light with 85% solar conversion efficiency.

In another study, a cellulose-based MD membrane was prepared by MFC through the carboxymethylation and defibrillation of wood pulp, followed by surface modification of MC surface for desalination of seawater.^1049^ In this membrane system, a two-layered fibrous structure of the cellulose-based composite membrane with high porosity was used. The upper layer, composed of MC, was made superhydrophobic by the addition of hydrophobic inorganic fillers and a sizing agent, which promoted a high vapor transportation rate and a high salt rejection ratio against seawater desalination. The bottom layer, also composed of MFC, maintained its hydrophilic nature and served as a water reservoir, exhibiting antifouling properties against the solution, as shown in Figure 52ciii. The system exhibited a permeation flux (∼23 L·m^–2^·h^–1^ at 60 °C) and rejection ratio (>97.5%) throughout the MD operation, which were comparable to those of commercially available PTFE membranes. It is worth noting that the wet tensile strength of the cellulose-based composite membrane could be enhanced by cross-linking with polyamide epichlorohydrin (PAE), a wet-strength resin used in papermaking, to achieve water resistant requirement for MD operations.

Finally, an all-weather solar still apparatus was designed based on a two-layered structure using cellulose materials.^1108^ In the material design, the top layer was composed of carbonized rice leaves bound by bacterial cellulose to serve as a light harvester and to maintain low thermal conductivity to avoid thermal loss. The bottom layer was hydrophilic rice straw and was designed as water pumps to promote water transport for surface evaporation in the still. The system exhibited high water harvest efficiency with 1.2 L·m^–2^·h^–1^ flux and 75.8% conversion efficiency, which are quite good compared with commercial solar stills. These properties, including high water vapor transportation, low thermal loss for heat transfer, and high antifouling properties, further offer the opportunity for industrial applications of the solar desalination process.

### 5.7 Agricultural Applications

#### 5.7.1 Mulch Films

Mulch films are increasingly used in the production of crops due to their multifunctional benefits including optimization of the soil and canopy microclimate (e.g., temperature augmentation and soil moisture conservation), weed suppression, and overall ability to efficiently promote yield and crop quality.^1109–1115^ Nonbiodegradable PE is the predominate polymeric feedstock used to manufacture mulch films due to its relatively low price and the resulting product is easy to apply by mechanical mulch layers and possesses high durability.^1116^ Globally, approximately 2.5 million tons of plastic mulch film are used annually in agriculture.^1117^ Seldom is this mulch film reused, and annual applications results in large volumes of plastic waste being generated every year with few sustainable disposal options (Figure 53a). Rarely is plastic mulch film recycled due to high contaminant loads (30% to 80% by weight) from soil and plant debris that adds weight and increases transportation costs to a recycler, can damage recycling equipment, and negatively impacts downstream recyclate yields and quality.^1118–1122^ In the United States, most used mulch film is landfilled although some growers will dispose of used agricultural plastic waste, including mulch film, by open-burning or burial on farm.^1123^ Incomplete removal due to rips and tears leads to fragments of mulch film remining in the soil, creating micro- and nanoplastics that threaten soil and broader ecosystem health.^1124,1125^

**Figure 53:**
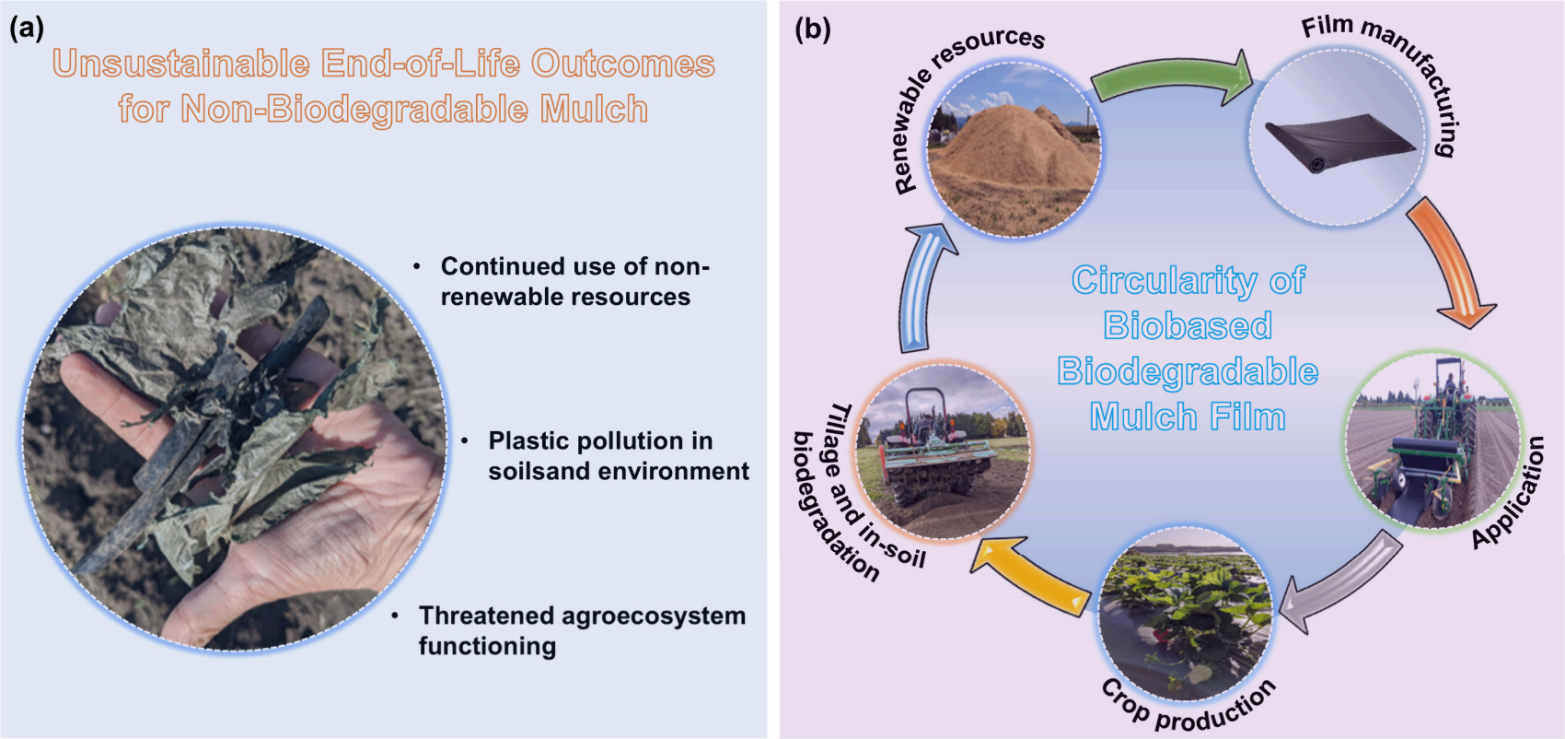
(a) Conventional plastic mulch film is not biodegradable and becomes a persistent, environmental pollutant that threatens agroecosystem health and functioning. (b) Circularity of biobased, soil-biodegradable mulch film encompasses utilization of renewable resource, film manufacturing, utilization in crop production, and eventually soil-incorporation in the field whereby renewable feedstock resources can eventually be reharvested and utilized.

Soil-biodegradable plastic mulch (BDMs) films made with a blend of compostable or soil-biodegradable feedstocks such as poly­(butylene adipate-*co*-terephthalate) (PBAT), polyhydroxyalkanoate (PHA), polylactic acid (PLA), and thermoplastic starch are a promising alternative as they are designed to biodegrade in soils after tillage, thus eliminating waste generation and saving costs by avoiding mulch film removal and disposal fees incurred by growers. Meta-analyses indicate BDMs function similarly to PE mulch,^1126^ however adoption among the farmer community remains low despite its commercial availability since the 1990s. BDMs are considered “risky”^1116^ with barriers to adoption including higher initial costs, questionable durability, unknown breakdown times that could negatively impact the environment, and poor on-farm aesthetics as the material deteriorates.^1123,1127–1129^ Furthermore, certified organic growers in some countries including the United States and Canada are not permitted to use commercially available BDMs primarily due to requirements for 100% biobased content.^1130,1131^ Additional requirements for use in certified organic agriculture in the United States includes production without organisms or feedstocks derived from excluded methods, achieving at least 90% biodegradation in soil within 2 years (based on ISO 17556 or ASTM D5988 standards), and meeting composability specifications (following ASTM D6400, ASTM D6868, EN 13432, EN 14995, or ISO 17088).^1132^ PE mulch, however, is permitted in certified organic agriculture as long as the mulch film is completely removed at the end of the season.

Application constraints of commercially available BDMs and increasing focus on circular economies has inspired renewed effort to develop fully biobased mulches made with renewable resources (Figure 53b). The development of RC films using modified cellulose is more advanced relative to biocomposites containing cellulose with lignin. Cellulose from corn stover, cassava peelings, cotton fibers, bacteria, and other plant materials have been explored for food packaging^1133^ and other agricultural applications including mulch film and seed germination covers.^335,1134,1135^ Cellulose films show good degradation in soil burial tests in part due to their capacity to absorb moisture, which causes microcracking and facilitates biodegradation.^1136^ Biodegradation rates of up to 90% in 30 days after soil burial has been observed for cellulose films made for food packaging^1133^ with complete degradation observed within 63 and 112 days after burial in late summer and spring, respectively, for film made with cotton fibers.^1134^ These degradation rates may be too fast for agricultural applications as a mulch, where films should undergo minimal visible deterioration on the soil surface to maintain the weed suppressive and horticultural benefits throughout the crop cycle, which may be over a year depending on the crop species. Maintaining mulch mechanical and structural integrity throughout the growing season and balancing biodegradability with durability will be key challenges in the development of fully biobased mulches made with renewable resources.

Biocomposites of cellulose have the potential to improve material and functional properties. Moreover, additives can be included to create multifunctional mulch films with added value such as antimicrobial properties to prevent plant disease or foodborne illness as well as nutrient and plant growth promotor release. Alginate is one promising biopolymer derived from the cell walls of brown algae that has demonstrated superior initial mechanical properties (as measured by tensile strength) when combined with HEC and saponin- AgNPs relative to low density PE.^1137^ Hybrid cellulose films with degradable AgNWs have also been fabricated with improved heat preservation properties that would be beneficial under cold-climate conditions or for cold-sensitive crops.^1138^ Inclusion of AgNWs also provided antimicrobial effects by reducing the growth of bacterial pathogens, *Escherichia coli* and *Staphylococcus aureus*. Keratin derived from chicken feather waste has also been incorporated into cellulose mulch film with calcium carbonate added to improve keratin material properties and partially crystalline polycaprolactone to act as potential nutrient carrier for plants.^1139^ Additions such as keratin and calcium carbonate have the potential to provide plants with small amounts of essential nutrients and reduce grower dependency on fertilizer, although they were found to reduce some mechanical properties such as tensile strength and modulus of elasticity relative to film made only with RC. While many of these biocomposites of cellulose report overall good mechanical properties including stretchability, a requirement for mulch film application, how long these mechanical properties persist following field application and exposure to the crop growing environment is a critical next step to better understand their compatibility with agricultural production.

Biocomposites of cellulose containing modified, pretreated lignin as an additive can improve mulch film properties due to its optical and mechanical properties while still maintaining biodegradability.^478,1140^ However, it will continue to be important to assess these properties relative to PE mulch throughout a crop cycle as these properties may change over time due to weathering. The high UV resistance of lignin should contribute to reduced weathering and deterioration when deployed in agricultural fields and improve overall film functionality for agricultural mulch applications, yet this remains to be more systematically evaluated.^1141,1142^ Lignin-containing films also tend to be dense due to molecular cross-linking which in turn can be beneficial where moisture conservation is important either through regulatory or environmental pressures.^478^ Biocomposite films with cellulose and lignosulfonate also show good antifouling properties and can be made black in color, which is more traditional for mulch film in many growing environments.^321,1143,1144^ Color and transmissivity are important for optimizing crop growth as well as preventing weed seed germination and growth underneath the mulch.^1145^ Addition of CuNWs to lignocellulosic nanofibers has also shown potential to improve hydrophobicity and overall water resistance, insulation ability, some light transmittance, flexibility, and antimicrobial properties.^1142^ Hollow SiO_2_ microspheres have also been blended into lignocellulostic nanofibers to improve thermal and optimal properties.^1146^

As a natural biopolymer, lignin is biodegradable. However, biodegradability tests in most studies using cellulose-containing films are limited to burial of unweathered samples under a range of environmental conditions that ultimately influence decomposition rates. Standardizing degradation tests so they are in greater alignment with the European Standard EN 17033 would be a more representative and comparable way to assess biodegradation for agricultural applications.^1147^ Although EN17033 focuses on BDMs made with plastic feedstocks, it regulates and provides testing methods for not only biodegradation in soil, but also mechanical and optical properties, composition, and ecotoxicity. In addition, agricultural-oriented evaluations of cellulose-based and biocomposite mulch films are primarily limited to seed germination tests, which is not a realistic test of mulch performance. Seed germination tests entail covering seeds with a film for a set duration of time, removal, and observations of seed germination and seedling growth. In contrast, mulch films are applied over the soil with the crop traditionally planted after mulch application so that the crop is grown with the mulch. Seed germination tests are much shorter, using a film for a few days to weeks, whereas a mulch film is expected to be in place for several months or even beyond a year. The lack of studies that deploy experimental cellulose-based and biocomposite films as a mulch limits critical evaluation of its performance in agriculture. Future studies should evaluate emerging mulch technologies in experiments that better replicate mulch application conditions in open fields to better characterize impacts on crop growth and durability, as maintaining mechanical and structural integrity throughout the growing season will be an important challenge to address. The highly biodegradable composition of cellulose-based and biocomposite films will likely result in rapid degradation and the trade-off between durability and biodegradability will be another significant challenge. Commercialization of cellulose-based and biocomposite mulch films should also consider economics. Although the price of cellulose and lignin is competitive, scaling up production of a cast film may be costly and price point is an important factor influencing grower adoption.^1128,1129^ Tests of films with multifunctional attributes should also systematically evaluate impacts on the crop and soil environment to avoid unintended consequences such as over fertilization or disrupting the balance of nutrient delivery to plants.

#### 5.7.2 Other Applications

Agriculture depends heavily on plastics, many of which are single use in nature. Of the estimated 359 million tonnes of plastic produced globally, 3–5% of that share is used in agriculture.^1117^ Examples of other plastic products used in agriculture includes trays and pots used in nursery and substrate production, film coverings used for polyculture and high/low tunnels, drip irrigation tape and tubes, pesticide containers, fertilizer coatings and dust repellants, nets and ropes used in fishing and aquiculture, twine and ropes, and livestock feedbags. With increased attention on circularity and extended producer responsibility to enhance sustainability, there has been a shift to utilize alternative feedstocks in place of nonbiodegradable polymers to reduce plastic waste generation. Furthermore, biobased alternatives are being promoted to reduce the utilization of fossil-fuel derived plastics. Polylactic acid (PLA), polyhydroxyalkanoates (PHA), and thermoplastic starch (TPS) blends are common biobased alternatives and have biodegradable properties. However, nonbiobased and biodegradable polymers are also available including polybutylene succinate (PBS), polybutylene adipate terephthalate (PBAT), and polycaprolactone (PCL). Lignocellulose is a promising biobased polymer and offers circularity given it is derived from plant biomass which could be sourced from agroforestry or other agricultural operations. However, lignocellulose-based materials are deficient in thermoplastic properties. As such, they are currently not suitable for applications that require shaping and molding (e.g., drip tube, pots, trays, etc.) using standard plastic processing techniques. Film extrusion also benefits from thermoplastic properties within the feedstock, but casting films may overcome this constraint and permits manufacturing of LCF. The lack of inherent thermoplastic properties within lignocellulose limits its application unless it can be modified to exhibit thermoplastic properties. Pretreatments to obtain optimal fiber dimensions and fiber composition, the addition of plasticizers to the masterbatch, and chemical modifications such as acetylation with anhydrides show the most promise when it comes to improving thermoplasticity of lignocellulose, but the final product may suffer from negative effects on final material properties.^277,1148^

Like other biobased and nonbiobased feedstocks, well developed life cycle assessments are critical to evaluate the environmental impacts of lignocellulosic materials from cradle to grave (i.e., raw material extraction end of life). Some literature reviews suggest biobased polymers such as PLA, PHA, and TPS have similar environmental impacts as fossil-fuel derived plastics.^1149^ More recent literature by Ita-Nagy^1150^ indicates biomaterials are expected to lead to higher environmental impacts relative to fossil-fuel derived counterparts due to increased fertilizer use and associated land-use changes. Improving the production of these biobased materials should lessen these environmental impacts and continued life cycle assessments will be needed to assess and refine the production of biobased materials including LCFs that have the potential to improve sustainable agriculture.

## 6 Feasibility and Sustainability of Lignocellulosic Films

Sustainability encompasses economic, environmental, and social aspects. This section reviews the economic feasibility and environmental implications of LCFs as assessed in the literature. Social impacts have rarely been assessed in previous studies, highlighting a research gap that needs to be addressed in future research.

### 6.1 Economic Feasibility of Lignocellulosic Films

Techno-economic analysis (TEA) is a widely utilized methodology for evaluating the technical and economic performance of a project or product as they approach commercialization. TEA typically involves four primary steps: defining goal and scope, conducting inventory analysis, calculating TEA indicators, and interpreting the results.^1151^ The goal of TEA addresses the core questions, target products, and audiences. The scope delineates the system boundaries and comparative scenarios. Inventory analysis compiles economic data, such as costs of labor, materials and equipment, and technical factors, such as production capacity and mass and energy balances. Using inventory data, key indicators are quantified in TEA calculation. Common TEA indicators include capital investment, operation cost, minimum selling price (MSP), net present value, internal rate of return, and profits. The interpretation phase assesses the quality and robustness of results and derives conclusions and practical recommendations. For emerging materials, TEA provides critical insights from technical and economic perspectives to guide the scaling up and commercialization of technology.

No TEA studies have been published for LCFs, although some studies have used TEA to assess the use of cellulose in producing the monomer of conventional polymers.^1152,1153^ Given the lack of existing TEA literature, we break down LCF production into three main subprocesses: biomass treatment, cellulose-based material production, and film preparation (see Figure 54). We then review existing TEA studies that, while not focused on LCFs, address one or more of these subprocesses. This approach allows us to provide a clear roadmap for future work by identifying available TEA data that could be integrated into future analyses and pinpointing missing subprocesses or variables for LCFs.

**Figure 54:**
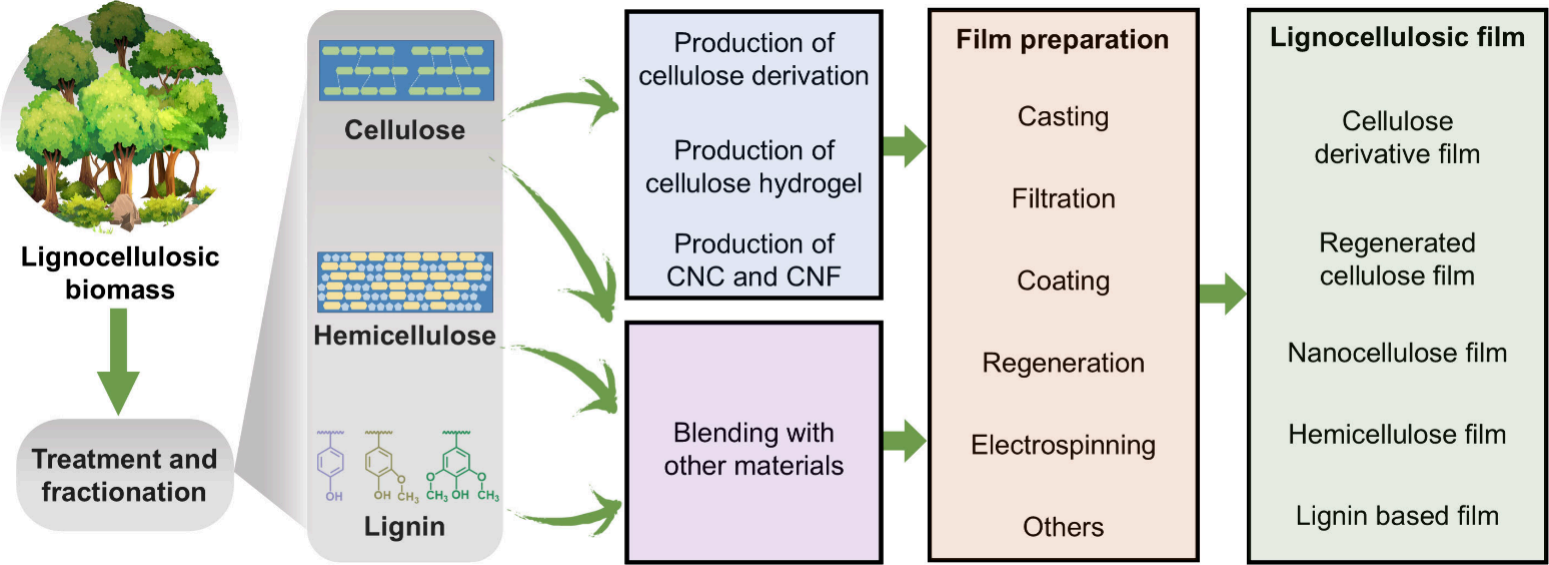
A generic flowchart for producing common lignocellulosic films.

Figure 54 presents generic processes for fabricating various LCFs, including cellulose derivative, RC, nanocellulose, hemicellulose, and lignin-based films. No TEA has been published for nanocellulose films, but previous TEAs have evaluated the costs of CNC or CNF, which are input materials for film preparation and expected to be the primary cost drivers. These studies highlight high production costs for CNC but suggest potential improvements to reduce them. For example, Rajendran et al.^1154^ investigated the techno-economic performance of CNC production with 100 dry metric tons per day from wood pulp. Their results showed that the MSP of CNC (US$4,890 per dry metric ton) can be reduced by adding the acid recovery process (US$4,690 per dry metric ton). Another TEA study assessed a CNC production plant with 50 dry metric ton per day from dissolving pulp, considering plant locations and acid recovery as variables.^1155^ Their results indicated that colocating a CNC plant with a pulp mill without acid recovery result in lower MSP (US$4,829 per dry metric ton of CNC) due to lower capital investment, compared to an isolated CNC plant with acid recovery (US$6,070 per dry metric ton of CNC). Other TEAs have explored the economic implications of alternative process design. For instance, Bondancia et al.^1156^ evaluated the economic performance of CNC production from sugar cane bagasse. In their study, sugar cane bagasse was pretreated and enzymatically hydrolyzed to extract sugar for ethanol production. The solid residues were used to synthesize CNC with acid recovery. The authors considered a traditional solvent, sulfuric acid, and an alternative, citric acid, which is expected to be more environmentally friendly. Their study concluded that using citric acid increased the MSP from US$6,897 (using traditional solvent) to US$10,452–10,974 per dry metric ton, due to the high cost of citric acid recovery. A more recent study, Lan et al.,^1157^ conducted both TEA and life cycle assessment (LCA, see section 6.2 for details) for CNC and CNF productions with alternative scenarios of process design using Green Chemistry principles. Those alternative scenarios include the use of calcium oxide to substitute sodium hydroxide, the incorporation of acid recovery, the use of citric acid and subcritical water rather than sulfuric acid, and different energy sources. Their citric acid scenario reached similar conclusions as Bondancia et al.,^1156^ showing citric acid as an economically unfavorable option. Furthermore, their LCA results pointed out that the citric acid scenario is not environmentally favorable, based on the current recovery technologies, due to higher water and electricity consumption. Across scenarios, substituting sodium hydroxide with calcium oxide for acid neutralization brings the largest cost reduction (decrease MSP from US$7,540 to US$4,286 per dry metric ton), which has additional benefits of producing valuable gypsums as byproducts. For CNF, the baseline scenario considers mechanical treatment using grid electricity, while the alternative process scenarios include the substitution of mechanical treatment by enzymatic hydrolysis with energy supply from natural gas, the use of wood pellets and wind power rather than natural gas and grid electricity. The authors found that these alternative processes yield more cost (US$2,985 per dry metric ton) than the baseline (US$ 2,873 per dry metric ton), due to the expensive enzymatic hydrolysis process and wood pellets. There is a lack of TEA research investigating nanocellulose films, although TEA studies have explored various raw materials, solvents, processes, and energy mix for producing CNC and CNF dry powders. Addressing this gap requires assessing the techno-economic performance of film preparation processes such as casting, filtration, coating, and electrospinning, as shown in Figure 54. Once the data of these processes are available, they can be integrated into the TEA of CNC and CNF powders to enable a comprehensive economic assessment of nanocellulose films. Another gap in the previous TEA studies is the lack of consideration of end-use applications. As a result, most TEAs use MSP, an indicator reflecting the production costs. Other important indicators, such as the net present value that is widely used to access economic feasibility, will require a quantitative assessment of revenue sources, particularly the values of downstream products derived from CNC and CNF. Nanocellulose films fabricated from CNC or CNF can be used for packaging, fuel cells, flexible and skin electronics.^866^ Some of these are high value-added applications, while the others have large production volumes in the current market. Considering the diverse applications and their respective revenue differences in future TEAs of nanocellulose films will shed light on priority areas for technology development and guide deployment toward more economically viable directions.

Similar to nanocellulose films, no TEA has been conducted for lignin-based films. TEA studies are only available for the subprocess, the fractionation of lignin from various sources. Jiang et al.^1158^ conducted TEA of lignin fractionation from kraft pulping, using sequential extraction with organic solvents. The authors identified the solvent choice as a main cost drivers and compared the use of four solvents, including acetone, ethanol, methanol, and methyl ethyl ketone. Their results showed that the MSP per metric ton of fractionated lignin was the lowest using acetone (US$729) and highest using methyl ethyl ketone (US$999). Kulas et al.^1159^ focus on lignin fractionation from waste streams of a corn stover-to-ethanol plant, using aqueous lignin purification with hot agents. Their results revealed that the choice of solvent significantly impacts TEA outcomes, with acetic acid resulting in a higher MSP (US$838) than ethanol (US$463). These findings highlight the importance of solvent selection, similar to these TEA studies of CNC and CNF discussed above. As lignin is commonly blended with other materials for producing composite films, future research can leverage the TEA results of lignin to quantify the techno-economic performance of composite films.

Only one TEA for hemicellulose film has been published to date. Fernández Méndez et al.^1160^ assumed the cost of hemicellulose to be 18% of the cost of thermos-mechanical pulping. Since hemicellulose is often blended with modifiers or reinforcing agents to produce composite films, its cost contribution may vary depending on the blending ratio, which should be taken into account in future TEA research. Different biomass sources typically have varying cost contributions, future studies should investigate the impact of biomass sourcing on the cost of hemicellulose films.

Few studies have conducted TEAs for RC films. However, since pulp is generally used as the raw materials, their cost information can provide some insights and be integrated into the future TEA of RC films. Previous studies of CNC and CNF have considered the cost of US$763–804 for dissolving pulp and US$1,055 for northern bleached softwood kraft pulp per dry metric ton. Future TEAs will need to quantify the cost of synthesizing regenerated hydrogel and the following film formation processes. Table 11 summarizes the availability of TEA studies that cover the subprocesses in LCFs production.

**Table 11:** Availability of TEA Studies Covering Subprocesses in LCF Production (N/A: Not Available)

LCF type	raw material acquisition	cellulose-based material production	film preparation	ref
nanocellulose film	yes	yes	N/A	^1154–1157^
lignin-based film	yes	N/A	N/A	^1158,1159^
hemicellulose film	yes	N/A	N/A	^1160^
regenerated cellulose film	yes	N/A	N/A	^1154–1157^

Although previous TEA studies have not assessed the cost-effectiveness of LCFs, some cellulose derivative films have been commercialized. Examples include CA, a versatile material with broad applications in textiles, adsorbents, and membranes.^1161^ The prices of CA films are around US$1,800–2,000 per metric ton.^1162^ The price of commercialized fossil-based films can also be a reference. For example, previous research has shown the prices of nondegradable films such as low-density PE (US$840–1,680 per metric ton), and biodegradable films such as polylactic acid (US$2,200 per metric ton), polybutylene adipate terephthalate (US$1,940–2,560 per metric ton), and PHA (US$5,510 per metric ton).^1163,1164^ These can provide comparative benchmarks. LCFs would be considered technically and economically feasible if their prices are comparable to or lower than these competitive alternatives. Future TEA studies should build upon previous LCA studies of subprocesses, with a focus on cost data collection for subprocesses that were not included in earlier LCA studies. Additionally, efforts should be made to evaluate the cost feasibility of potential strategies such as the functionalization of low-cost base materials or the modular coating of commodity substrates.

### 6.2 Environmental Impact of Lignocellulosic Films

LCA is a standardized method for assessing the environmental impacts of a product, adhering to standards such as ISO 14040 and 14044.^1165,1166^ An LCA consists of four phases. The first phase, goal and scope definition, establishes the objectives and system boundaries, encompassing all processes analyzed within an LCA. For example, a cradle-to-grave system boundary includes raw material acquisition, manufacturing, transportation, use-phase, and end-of-life. The second phase, inventory analysis, complies inputs and outputs for each process and aggregates them as the life cycle inventory. In the third phase, life cycle impacts assessment, the life cycle inventory data are converted into environmental impact indicators, such as global warming potential (GWP), which quantifies the climate change impact in the unit of CO_2_-equivalent (CO_2_e). Depending on the goal and scope of the LCA study, the final phase involves interpreting the results, drawing conclusions and making recommendations that support eco-design, supply chain improvement, or process optimization.

Several LCA studies have been conducted for LCFs, including cellulose derivatives,^1167–1169^ nanocellulose,^1157,1170–1174^ and RC films.^1175^ Most of these studies have cradle-to-gate system boundaries that mainly includes upstream production from raw material acquisition to material production, focusing on the environmental implications of different synthesis routes and material compositions. Araújo et al.^1167^ assessed the environmental impacts of synthesizing 10 g of CA from corncob. They compared a conventional synthesis routine, which uses alkali bleaching pretreatment and acetic acid-based acetylation, with a green approach using liquid hot water and dilute sodium hydroxide pretreatment and solvent free acetylation. Their results showed that the green approach had lower impacts than the conventional routine in all 13 indicators except freshwater ecotoxicity. This difference was attributed to the lower consumptions of energy and chemicals but higher copper emissions in the green approach. Kramar et al.^1168^ conducted an LCA for 1 kg of CA film, both with and without the reinforcement by adding chitin nanofibrils. Their results concluded that incorporating 1.5 wt % of chitin nanofibrils improved the tensile strength, Young’s modulus, and ductility of CA film. However, this reinforcement also resulted in higher GWP, marine eutrophication, terrestrial acidification, and water consumption. These trade-offs between technical performance and environmental costs are not uncommon. Developing performance-based environmental indicators is critical for understanding such trade-offs and supporting green design. LCAs of other materials (e.g., bioplastics) have incorporated material properties (e.g., tensile strength) into the LCA results, enabling simultaneous consideration of technical and environmental performance.^370^ Future LCAs of LCFs should consider these approaches to account for film-specific performances in combination with environmental sustainability.

Other LCA studies have focused on investigating different end-of-life pathways of CA film.^1169^ These studies often have gate-to-grave system boundaries that examine different end-of-life treatments of LCFs. Gadaleta et al.^1169^ conducted case studies in Italy where waste CA films were treated along with other waste materials, including anaerobic digestion with organic waste, incineration with mixed waste, and recycling with conventional plastic waste. Their LCA results showed that anaerobic digestion had the highest impacts in most environmental impact categories, such as GWP, fine particulate matter formation, and freshwater eutrophication. This can be explained by the limited degradation of the studied CA film during anaerobic digestion, resulting in low-quality compost with a higher fraction of CA residue than the local standard. To improve the quality of compost, screening processes were employed to remove most of the CA residues. These processes consume additional energy and lead to higher environmental burdens in the anaerobic digestion scenario. This highlights the significant effects of biodegradation on the environmental performance of CA film at EoL.

Previous LCAs of nanocellulose films have focused on understanding their potential environmental impacts compared to fossil-based films and how these impacts differ by end-of-life treatment, feedstock, and production pathways. Nadeem et al.^1170^ quantified the embodied energy for producing and recycling CNF films (2 g with an area of 16 cm × 16 cm). Their results showed that the embodied energy for producing a virgin CNF film (0.48 MJ) is higher than that of a virgin PET film (0.18 MJ) due to the high energy demand for extracting virgin CNF. However, recycling a CNF film (0.04 MJ) is less energy-intensive than recycling a PET film (0.14 MJ). If a virgin film is used and recycled three times, the overall embodied energy will be lower for CNF than PET films. Another LCA study compared the environmental performance of four CNF film preparation methods, including spray deposition, spray deposition with homogenization, vacuum filtration, and vacuum filtration with homogenization.^1171^ Their results showed that spray deposition has the lowest GWP, cumulative energy demand, and water usage. The study also reported that the CNF production is the largest contributor to GWP and cumulative energy demand, surpassing the impacts from pulp production and film preparation. In addition, Nadeem et al.^1172^ investigated the effects of various factors on the environmental performance of CNF films, including feedstock (eucalyptus or waste carrots), drying method (ambient or tunnel), solid content (1.5 or 4.5 wt %), composition (pure CNF or CNF/CMC composite), and locations of grid electricity (China, USA, Australia, Germany, and the UK). The baseline scenario used eucalyptus in pulp production, ambient drying method, 1.5 wt % of solid content, pure CNF without composite, and Australian grid electricity. Their results showed that the environmental impacts (e.g., GWP, water depletion, and ozone formation) of an alternative scenario, which involved waste carrots for pulp production, drying in a tunnel, CNF/carboxymethylcellulose composite (with a CNF/carboxymethylcellulose ratio of 1:1), 9 wt % of solid content (4.5 wt % from CNF), and British grid electricity, resulted in environmental impacts at least 80% lower than the baseline. The study of Lan et al.^1157^ (with scenarios described in section 6.1) showed that the substitution of neutralization agent from sodium hydroxide to calcium oxide could largely mitigate the environmental burdens of CNC production. These impacts can be further reduced by incorporating acid recovery, using subcritical water as a solvent, and using wood pellets and wind power for energy supply. For CNF production, Lan et al.^1157^ concluded that pretreatment with enzymatic hydrolysis and renewable energy sources lead to significant reduction of environmental impacts.

In addition to pure CNC or CNF, previous LCAs have examined the use of nanocellulose in composite films. For example, Rivière et al.^1173^ investigated 1 kg of lignin-CNF composite films sourced from recalcitrant hydrolysis lignin, a byproduct of bioethanol production. A selective extraction method using acetone and water was applied to the recalcitrant hydrolysis lignin to synthesize CNF and colloidal lignin particles, which were further prepared into lignin-CNF composite films. The authors considered lab-scale production as the baseline scenario and assumed that the energy demand of mixing and stirring for the scaled-up production was 16.7% of that for the lab-scale. Their LCA results showed that lab-scale production leads to 179 kg CO_2_e per kg film, which can be reduced to 26 kg CO_2_e per kg film in the scale-up production and be further mitigated to 3 kg CO_2_e per kg film using renewable energy. Ponnusamy and Mani focused on 1 kg of CS-based packaging films reinforced by CNF.^1174^ CS, manufactured from shrimp shell, was mixed with CNF, followed by a solvent casting method to prepare films. Their results showed that the CS production had the largest contribution to all the impact categories except GWP and fossil fuel depletion, where the film casting had the highest impacts. The authors further reported that the GWP of CS-CNF films is slightly lower than that of commercial films such as low-density PE and poly­(lactic acid).

To date, only one LCA has been published for RC film. Wang et al.^1175^ synthesized cellulose hydrogels from cotton linter pulp in an alkali hydroxide/urea aqueous system and applied hot pressing to prepare RC films. The authors applied an aggregated impact indicator, total environment load, which combines various environmental impacts such as global warming, acidification, eutrophication, human toxicity, and others. Their results showed that the RC film achieves more than 70% reduction in the total environment load compared with fossil-based PE films.

To compare different LCA studies for LCFs, Table 12 presents a summary of key information including GWP, system boundaries, technology readiness levels, and intended applications. Our review shows that most existing studies adopt a cradle-to-gate scope, with end-of-life (EoL) stages rarely addressed. Future LCA should include a full cradle-to-grave system boundary and consider various EoL scenarios like landfilling, recycling, and composting. Furthermore, the applications of LCFs are typically limited to packaging or are not explicitly defined. Future research should expand to include other promising applications, such as electronic substrates and agricultural mulch films, where LCFs may offer notable environmental benefits.

**Table 12:** Summary of LCA Studies for LCFs (TRL, Technology Readiness Level; GWP: Global Warming Potential)

LCF type	GWP [kg CO_2_e/kg]	system boundary	TRL	application	ref
CA film	70	cradle-to-gate	9	not specified	^1168^
CA reinforced film^a^	129–173	cradle-to-gate	7–9	not specified	^1168^
CA film^b^	0.13	end-of-life	9	packaging	^1169^
CNF film^c^	9–17	cradle-to-gate	1–3	not specified	^1171^
CNF film^d^	6	cradle-to-gate	1–3	packaging	^1172^
CNF^e^	8	cradle-to-gate	1–3	not specified	^1157^
CNC^e^	18	cradle-to-gate	1–3	not specified	^1157^
lignin-CNF film^f^	3–179	cradle-to-gate	1–3	packaging	^1173^
chitosan-CNF film	4	cradle-to-gate	1–3	packaging	^1174^

a Reinforced by chitin nanofibrils.

b Results are adjusted from 18.8 kg of waste CA films in the original paper.

c Results are adjusted from 2 g of CNF films in the original paper.

d Results are adjusted from 1 t of CNF films in the original paper.

e Results are based on 1 kg of dry CNF or CNC without considering film preparation.

f Lab-scale: 179 kg CO_2_e·kg^–1^; Large-scale: 26 kg CO_2_e·kg^–1^; Large-scale with renewable energy: 3 kg CO_2_e·kg^–1^

### 6.3 Environmental and Economic Trade-offs

Although individual TEA or LCA studies have quantified the techno-economic or environmental performance of LCFs, their potential trade-offs or cobenefits remain unclear. Technologies with lower environmental burdens often tend to be more expensive than conventional technologies. The concept of eco-efficiency has been developed to evaluate the relationships between economic values and environmental impacts.^1176^ Various eco-efficiency analysis frameworks and indicators have been proposed and applied to different industrial systems, including energy systems,^1177,1178^ fuels, wastewater treatment,^1179^ agriculture systems,^1180^ and materials.^1181,1182^ However, none of the previous LCF studies have applied eco-efficiency, with the exception of a study by Lan et al.^1157^ They proposed a framework that integrates TEA, LCA, Green Chemistry principles, and eco-efficiency analysis to identify lower-cost and greener manufacturing pathways for nanomaterials. They demonstrated this framework through case studies for CNC and CNF.^1157^ Their framework is shown in Figure 55 where TEA and LCA are interactively employed to determine the economic and environmental impacts. Improvement strategies and scenarios are identified by applying Green Chemistry principles. Eco-efficiency analysis quantifies potential environmental-economic trade-offs and cobenefits by assessing the differences in environmental impacts divided by the differences in production costs of the improved scenario against a baseline. This framework can be adapted to different materials, and more LCF case studies are needed to understand the potential environmental-economic implications of various synthesis routes, applications, and end-of-life options. For example, while CNF films exhibit lower environmental impacts than CA films in packaging applications, it is essential to incorporate economic analysis to assess their overall eco-efficiency. Assessing the eco-efficiency of different types of LCF in future research is crucial for determining the most promising ones from both environmental and economic perspectives. In broader applications such as flexible electronics and energy storage, varying degrees of functionalization may be needed to enhance the physical properties of LCFs. Scenario analysis with different functionalization levels can be conducted to understand how they simultaneously affect economic and environmental performance, informing further optimization. Additionally, advanced models are needed to evaluate the EoL pathways for used LCFs, including recycling, biodegradation, and incineration, and integrated into a combined TEA-LCA framework to holistically assess multiple dimensions of sustainability.

**Figure 55:**
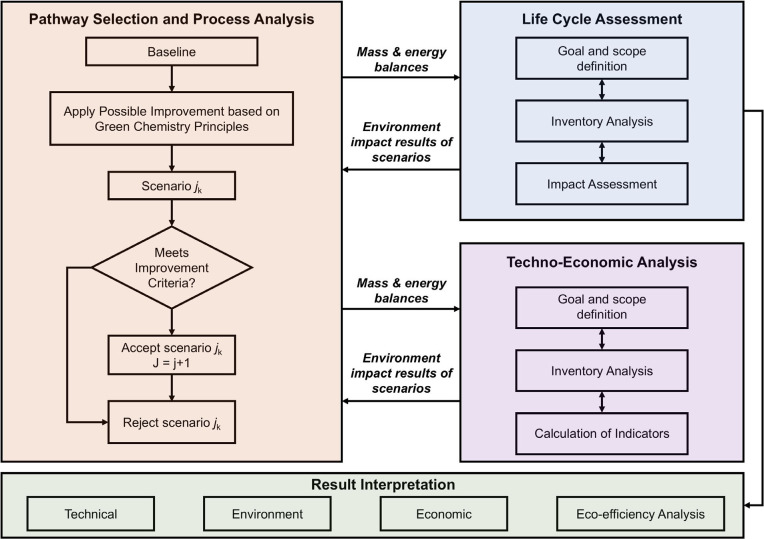
Framework to explore the trade-offs or cobenefits between economic and environmental performance. Adapted with permission from ref 1157. Copyright 2024 Royal Society of Chemistry under CC BY 3.0 (https://creativecommons.org/licenses/by/3.0/).

The future commercialization of LCF presents a significant opportunity for advancements in sustainable materials. A strategic approach to their production is crucial. One potential route is establishing new manufacturing plants dedicated to LCF production. While this may seem straightforward, it is essential to consider market demands and appropriate plant capacity to scale economically, considering capital and operational costs as well as supply chain constraints. An alternative approach is integrating LCF production into existing industrial plants, e.g., pulp and paper mills or plastic film manufacturing facilities. This strategy leverages existing infrastructure and can reduce costs. These strategies will need to be assessed on a case-by-case basis, depending on local markets and resources. Future LCA and TEA should evaluate different process and supply chain design strategies for LCF production to inform commercialization decisions. For example, future research can investigate the integration of cellulose acetylation and solvent casting processes, which are necessary for LCF fabrication, into pulp and paper mills or retrofitting existing plastic film production plants to utilize cellulose as a feedstock for CNF films. These comparative analyses will be invaluable in developing strategies that balance innovation, sustainability, and cost-effectiveness in LCF commercialization.

## 7 Challenges and the Way Forward

### 7.1 Challenges in Large-Scale Production

#### 7.1.1 Processing Limits and Scale-Up

Scaling up the production of LCFs from the laboratory to an industrial scale presents significant challenges. While methods such as casting and filtration are effective in laboratory settings, they often encounter issues at larger scales, including uneven film formation and defects. For instance, simple solvent casting can produce small-size uniform films, but maintaining consistent thickness and quality in large-scale production is difficult. Similarly, drying CNF or CNC suspensions without aggregation is challenging due to strong hydrogen bonding between fibers, which can hinder nanoscale dispersion and lead to inconsistent material properties.

To address these challenges, researchers are exploring advanced coating techniques, such as slot-die coating and spraying, to enhance uniformity and production efficiency. These methods offer better control over film thickness and are inherently more suitable for large-scale manufacturing. However, process optimization remains crucial to preventing defects such as cracks and bubbles, as well as ensuring strong adhesion between layers in multilayer coatings. Further refinement of process parameters continues to be a key focus in current research.

#### 7.1.2 Cost Factors

Cost competitiveness remains a significant hurdle for the large-scale production of LCFs, especially for those made from nanocellulose, which are currently more expensive than conventional plastics. The TEA literature indicates that even under optimized conditions, the lowest selling price of CNC materials ranges from $4,000 to $6,000 per ton. For instance, a TEA estimate for a CNC plant producing 100 tons per day reports a MSP of approximately $4,890 per ton.^1154^ This is significantly higher than the cost of traditional plastics like PE, highlighting the economic challenge.

Several factors contribute to these high production costs, including the energy-intensive nature of fiber processing, the requirement for specific solvents or reagents, and the complexity of multistep processing. Recent studies suggest that certain process innovations can help reduce costs. For example, replacing traditional chemicals with more cost-effective alternatives has shown promising results. One study demonstrated that using calcium oxide instead of sodium hydroxide for acid neutralization in CNC production significantly lowered costs, reducing the MSP from approximately $7,540 per ton to $4,286 per ton while generating valuable gypsum as a byproduct.^1157^ Although such improvements help narrow the cost gap, further technological advancements and process optimization are still required to make LCFs cost-competitive with petroleum-based plastics.

#### 7.1.3 Material Consistency and Quality Control

Ensuring batch-to-batch consistency is another significant challenge in large-scale production. Lignocellulosic raw materials, such as wood pulps and agricultural residues, naturally vary in composition, which can lead to inconsistencies in film color, transparency, and mechanical strength during mass production. Strict raw material quality control and standardized preprocessing steps, such as pulping and bleaching, are essential for maintaining uniform material properties. Continuous production methods, such as roll-to-roll processing, also present engineering challenges related to temperature control, solvent evaporation rate, and film tension management.

For example, in the large-scale production of RC films, certain solvent systems, such as ionic liquids and NaOH/urea, may face difficulties in solvent recovery and reuse. Low recovery efficiency not only increases production costs but also poses environmental and safety risks. To enable the industrial-scale production of LCFs, strong process engineering capabilities are required to optimize chemical recycling and minimize material variability. While many high-performance LCFs have been successfully demonstrated at the laboratory scale, achieving reliable, cost-effective large-scale production remains a critical challenge for the future.

### 7.2 Performance Limitations: Mechanical Properties and Functionalization

#### 7.2.1 Mechanical Strength and Durability

LCFs differ from plastic ones in mechanical properties, which can be a limitation in certain applications. A common issue is their low fracture strain, meaning they tend to be more brittle and less stretchable than plastics. For example, pure cellulose or nanocellulose films typically exhibit high elastic modulus and tensile strength but low elongation at break, making them prone to fracturing under tension. This brittleness poses challenges for packaging applications that require flexibility or the ability to withstand deformation during processing and use. Additionally, while pure cellulose films offer excellent strength-to-weight ratios, their mechanical properties are highly sensitive to humidity. In moist environments, these hydrophilic films readily absorb water, leading to a plasticizing effect that reduces strength and stiffness. Maintaining mechanical integrity under varying environmental conditions remains a significant challenge.

Strategies to enhance toughness include the addition of plasticizers, blending with more ductile biopolymers, and designing composite structures. For example, incorporating small amounts of glycerol or other plasticizers can improve flexibility but often comes at the cost of reduced strength. Alternatively, reinforcing LCFs with fibers or fillers, such as nanoclay or lignin nanoparticles, can enhance both strength and toughness, though this may impact transparency or uniformity. Achieving an optimal balance between these factors is essential to tailoring mechanical properties for specific applications.

#### 7.2.2 Barrier Properties and Humidity Sensitivity

A common limitation of LCFs is their poor water vapor barrier performance. Due to their hydrophilic nature, polysaccharide-based materials like cellulose films exhibit high WVP, and their oxygen barrier properties can further deteriorate when exposed to moisture. In contrast, plastics such as PE offer excellent moisture resistance. However, untreated LCFs typically provide poor water vapor barrier properties. This limits their use in applications like food packaging, where humidity control is critical. To address this issue, researchers have explored various functionalization methods. One approach involves coating or impregnating the film surface with hydrophobic compounds, such as waxes, fatty acids, or biopolymer coatings, to create a water-resistant layer. Another strategy is the development of multilayer structures. In this approach, cellulose films are combined with biodegradable polyesters or other hydrophobic layers to enhance barrier performance. However, these methods pose challenges. One challenge is maintaining biodegradability while ensuring a simple and scalable manufacturing process. Additional coatings or layered structures can increase production complexity and may impact the material’s recyclability or degradation behavior. Despite these challenges, continuous advancements in functional coatings and composite film designs are gradually narrowing the performance gap between biobased films and conventional plastic films.

#### 7.2.3 Functionalization Limitations

While LCFs are valued for being biobased and biodegradable, integrating additional functionalities such as conductivity, antibacterial properties, or environmental responsiveness remains challenging. Many high-value applications, including flexible electronics and smart packaging, require films with electrical conductivity, antimicrobial properties, or stimuli-responsive behavior. Achieving these functionalities often necessitates incorporating nonlignocellulosic components, such as metal nanoparticles, conductive polymers, or chemically modified cellulose molecules. However, the chemical functionalization of cellulose through methods such as grafting or nanoparticle loading frequently involves harsh reagents or processing conditions that may compromise the material’s sustainability. Excessive modification can also reduce biodegradability or biocompatibility. For instance, some nanoparticles or synthetic polymers may persist in the environment after degradation, potentially leading to long-term pollution concerns. To develop multifunctional LCFs while maintaining their sustainability, advances in materials science and green chemistry are essential. Innovative functionalization strategies that minimize environmental impact and maintain biodegradability will be key to overcoming these challenges.

### 7.3 Biodegradability and End-of-Life Considerations

#### 7.3.1 Balancing Biodegradability and Performance

One of the key advantages of LCFs is their biodegradability, allowing them to break down into natural substances (CO_2_, water, and biomass) through microbial activity under suitable conditions. Unlike plastics, which can persist in the environment for hundreds of years, cellulose-based films typically degrade within months to years in compost or soil, depending on their thickness and environmental conditions. However, biodegradability can be a double-edged sword. If a material degrades too quickly or in unintended environments, it may compromise product shelf life or stability. Therefore, achieving a balance between durability during use and controlled degradation after disposal is essential. Many LCFs are engineered to remain stable in dry, room-temperature conditions while degrading efficiently in humid, microbe-rich environments such as industrial composting facilities or soil. Achieving this balance may involve incorporating specific biodegradation enhancers that trigger decomposition only under certain conditions, such as increased humidity or microbial activity. However, hydrophobic coatings that enhance water resistance can also slow down biodegradation. Any surface treatment or additive must ensure that the material continues to meet compostability standards, such as ASTM D6400 or EN 13432, which are required for compostable packaging.^1132^

#### 7.3.2 Environmental Degradation and Microplastic Risks

One of the key motivations for using LCFs is to reduce plastic pollution, particularly microplastic contamination, due to their superior degradability. Unlike conventional plastics, LCFs typically degrade completely without leaving behind harmful microplastics. For example, cellulose-based agricultural mulch films can be broken down and assimilated by soil microbes within a few weeks, whereas PE mulch films tend to fragment into persistent microplastics that may persist for centuries. However, the rate and completeness of biodegradation depend on environmental conditions and material modifications. In marine environments or cold climates, even materials labeled as “biodegradable” may persist longer than expected. Recent studies indicate that biodegradability is not a “magic solution” to plastic pollution—materials do not vanish instantly and still require suitable waste disposal conditions.^1183^ If LCFs end up in environments with low microbial activity, such as deep-sea waters, their degradation may slow down significantly.^1184^ Moreover, chemical modifications during LCF production, such as a high degree of substitution that replaces the hydroxyl groups on the cellulose backbone with other functional groups, can further hinder degradation in natural water.^1185^ Therefore, simply replacing plastics with biodegradable alternatives without improving waste management practices may only shift the form of pollution rather than eliminate it. To fully realize the benefits of biodegradability, effective collection and disposal systems are necessary to ensure that these films reach environments where they can properly degrade, such as composting facilities or well-managed soil applications.

#### 7.3.3 Standards and Regulatory Environment

The end-of-life treatment of biobased films is closely tied to regulations and standards that define and regulate biodegradability. Globally, support for compostable and biodegradable packaging is increasing, along with stricter scrutiny against “greenwashing”.^1^ Certification bodies such as OK Compost, BPI, and the EU’s Seedling label ensure that products meet specific industrial or home composting standards. However, a key challenge is that not all regions have large-scale composting or organic waste treatment infrastructure. If a city lacks industrial composting facilities, compostable films may end up in regular landfills or recycling systems, which are not ideal disposal routes. This is why regulators remain cautious about promoting biodegradable plastics, especially in applications where they could contaminate recycling streams or where composting facilities are unavailable. Recent policy evaluations suggest that biodegradable plastics should be targeted for specific applications, such as agricultural mulch films or food waste bags, to ensure they are properly collected and processed rather than being used indiscriminately. To maximize the environmental benefits of LCFs, a comprehensive approach is needed. This includes designing materials that degrade in appropriate environments, implementing waste management systems to direct them to suitable disposal routes, and ensuring clear labeling and public education. Looking ahead, more standardized biodegradation tests for different environments (soil, marine, composting) will be essential to support credible end-of-life solutions for these materials.

### 7.4 Commercialization and Market Adoption Barriers

#### 7.4.1 Market Competition and Application Expansion

Despite significant advancements in material performance, LCFs still face challenges in achieving widespread market adoption. One of the major hurdles is competition with well-established and cost-effective traditional plastics. Fossil-based plastics have benefited from decades of optimization and large-scale production, leading to lower costs and extensive market penetration. In contrast, biobased films, including lignocellulosic alternatives, have yet to reach the same level of industrial maturity. As a result, many lignocellulosic and other biobased films remain more expensive than petroleum-based plastics and are produced on a much smaller scale. Currently, the global annual production of 100% biobased plastics is only about 2 million tons, with biodegradable plastics making up approximately two-thirds of this total.^1186–1188^ In stark contrast, global fossil-based plastic production exceeds 380 million tons per year.^1189^ This vast disparity highlights the early stage development of the biobased plastics industry. High production costs, as discussed in section 7.1, lead to higher prices for end products, which can discourage consumers and businesses from adopting lignocellulosic film packaging and related products. Until large-scale production helps lower costs, many companies may struggle to justify replacing petroleum-based plastics except in high-value markets or sectors where regulations mandate the use of sustainable alternatives.

#### 7.4.2 Infrastructure and Compatibility

Another significant challenge for LCFs is their compatibility with existing plastic processing and recycling infrastructure. The current industrial system is heavily optimized for traditional plastic materials, with packaging production lines designed to accommodate specific properties such as heat-sealing behavior, strength, and thickness. Introducing a new type of film may require equipment adjustments or modifications, which industries are unlikely to adopt unless driven by strong market demand or regulatory pressure. Additionally, existing recycling systems are primarily designed for common plastics such as PET, PE, and PP. If LCFs are not properly sorted, they could contaminate plastic recycling streams or be excluded from recycling altogether, as they are primarily intended for biodegradation or composting rather than traditional mechanical recycling. Without parallel developments in composting infrastructure or dedicated recycling channels, the market appeal of biodegradable films may remain limited. Thus, widespread market adoption of LCFs depends not only on material performance but also on the development of supporting industry infrastructure, including advancements in production equipment and waste management systems to facilitate their integration into the existing packaging landscape.

#### 7.4.3 Regulatory and Policy Barriers

For LCFs, the regulatory landscape presents both opportunities and challenges. On one hand, governments worldwide are tightening restrictions on plastic pollution, creating growth opportunities for sustainable materials. Many regions have implemented bans on single-use plastics and introduced mandates requiring packaging to contain a minimum percentage of biodegradable or biobased content, which could accelerate the market adoption of LCFs. On the other hand, regulatory approval processes can be complex, particularly in sectors such as food packaging, where materials must meet stringent safety standards set by agencies like the FDA (U.S.) or EFSA (EU). These requirements include chemical safety testing, allergen assessments, and migration tests to ensure that no harmful substances leach into food. Natural-origin films may raise concerns regarding residual components, such as lignin or extractives, and their safety for direct food contact. Another challenge is the lack of global regulatory consistency. A film certified as compostable in the EU may not be recognized in the U.S. or Asian markets, leading to trade barriers and confusion for manufacturers. Additionally, plastic-related policies are evolving rapidly, with new standards emerging for biodegradability, recycling, labeling, and biobased plastic trade. This regulatory uncertainty makes long-term investment planning more difficult for businesses. For example, if a country mandates that only materials capable of degrading in home composting (low-temperature conditions) can be labeled as compostable, LCFs requiring industrial composting may be excluded from the market. Despite these uncertainties, the overall regulatory trend favors green materials, and companies that successfully obtain certifications and compliance approvals will gain a competitive advantage in the growing market for sustainable packaging solutions.

#### 7.4.4 Consumer Awareness

Consumer acceptance is critical for the successful adoption of LCFs. With public concern over plastic pollution at an all-time high, there is strong support for biobased and biodegradable packaging. However, past failures of so-called “degradable” plastics, such as products that only break down under industrial conditions but were misleadingly marketed as universally biodegradable, have led to skepticism about eco-friendly materials. Educating consumers on the proper disposal of lignocellulosic film products is essential. For example, compostable packaging should be directed to composting facilities, rather than being casually discarded or mixed with conventional plastic waste. Clear communication can help ensure these materials deliver their intended environmental benefits. Additionally, industries must practice transparency when promoting the sustainability of these films. Misleading claims or greenwashing could further erode consumer trust and slow adoption. Overall, LCFs hold strong market potential, but for large-scale adoption, they must be cost-competitive, compatible with existing infrastructure, compliant with regulations, and trusted by end users.

### 7.5 Future Directions: Technological Advancements and Strategies

#### 7.5.1 Technological Innovation

Overcoming the challenges discussed above requires targeted technological advancements. One key area is large-scale process engineering. Developing continuous manufacturing techniques, such as roll-to-roll processing and advanced coating strategies, can significantly enhance production efficiency and reduce costs. For instance, industrial coating methods like slot-die coating and spray deposition are being optimized for high-throughput, uniform lignocellulosic film production. Integrating these methods with real-time quality control technologies, such as optical thickness monitoring and defect detection, will help ensure consistent large-scale production. Additionally, innovations in green chemistry for biomass pretreatment and film fabrication can improve sustainability. Researchers are exploring recyclable and environmentally friendly solvent systems, such as ionic liquids and DES, as alternatives to traditional harsh chemicals. Enzymatic pretreatment and mechanochemical processes may also help reduce the energy consumption of nanocellulose production. However, current studies indicate that enzyme costs remain high, requiring further optimization for commercial viability.

On the materials side, developing composite films offers a promising direction. For example, combining a cellulose layer with starch-based or polyhydroxyalkanoate (PHA) layers can leverage the strengths of each material to enhance overall performance. These composites can be engineered to either separate or degrade together, ensuring an eco-friendly end-of-life process. Nanotechnology also provides new opportunities. The incorporation of nanofillers such as nanoclay, graphene, or CS nanofibers can significantly improve barrier properties and mechanical strength even at low concentrations. However, the challenge lies in achieving these enhancements without compromising transparency or biodegradability. Research on surface functionalization and bionanocomposites is steadily progressing to address these concerns.

Overall, continuous innovation in processing techniques and material development will gradually narrow the performance and cost gap between LCFs and conventional plastics, bringing them closer to widespread commercial adoption.

#### 7.5.2 Sustainability Strategies

A comprehensive sustainability strategy is essential for enhancing the competitiveness of LCFs. In research and development, life cycle assessment (LCA) and techno-economic analysis (TEA) should become standard practices to ensure that innovations not only improve performance but also optimize environmental and economic performance. By identifying key contributors to environmental impacts, such as carbon footprint, and production costs, researchers can identify improvement strategies, such as incorporating renewable energy sources, recycling water and solvents. Moreover, LCA and TEA help evaluate whether a process modification enhances environmental and economic performance, providing insights into potential environmental-economic trade-offs or cobenefits. For example, a more environmentally friendly process design may incur higher costs, or, ideally, an alternative design could simultaneously reduce both environmental impacts and costs. Studies have shown that industrial-scale production usually has lower environmental impacts than that of lab-scale experiments. One LCA study found that the carbon footprint of lab-produced CNF films was 179 kg CO_2_·kg^–1^, but this could drop to 26 kg CO_2_·kg^–1^ at an industrial scale due to an improved energy efficiency, and even further to 3 kg CO_2_/·kg^–1^ if renewable energy is used.^1173^ This highlights the potential for large-scale production and clean energy adoption to minimize LCFs’ carbon footprint. Industrialization efforts should adopt circular economic strategies by improving energy and material efficiency. Renewable energy can also be an additional support of eco-design if its availability is reliable.

Another key sustainability strategy is the use of waste or nonfood biomass as raw materials. Utilizing agricultural residues or forestry byproducts avoids competition with food resources while creating value from low-cost biomass. This “waste valorization” approach can avoid emissions from waste (e.g., decay from residue biomass) potentially reduce production costs. Additionally, designing biodegradable end-use products is crucial. Researchers are developing films that can break down more efficiently in composting environments and even be safely digested by microorganisms, achieving true “zero waste”. This “eco-design” approach that integrates considerations of raw materials, production processes, and disposal methods is essential for future development of lignocellulosic materials.

#### 7.5.3 Policy Recommendations

Policies play a crucial role in promoting the adoption of LCFs. Governments and international organizations should consider implementing financial incentives, such as subsidies or tax benefits for biobased film manufacturers. Just as the renewable energy industry has expanded through feed-in tariffs and subsidies, biobased materials could become more economically competitive through similar financial support, helping to close the cost gap with petroleum-based plastics. Another key policy recommendation is the establishment of clear standards and labeling for biodegradable and biobased products. When consumers recognize certified labels (such as OK Compost or BPI certification) and manufacturers adhere to strict standards, market trust will increase, reducing confusion about proper disposal. Additionally, targeted regulations could mandate biodegradable films in specific sectors. For instance: 1) Requiring agricultural mulch films to be biodegradable to prevent plastic accumulation in soil; 2) Mandating compostable food packaging to ensure it enters organic waste processing systems rather than contaminating conventional recycling streams. Such regulations would create direct market demand for LCFs.

Expanding EPR programs is another viable policy tool.^1^ This would require manufacturers to take responsibility for the end-of-life management of biobased packaging, including funding composting facilities or waste collection programs. Furthermore, policy decisions should be grounded in scientific evidence. Not all biobased materials are inherently sustainable, and if new data indicate that a particular film degrades poorly in natural environments, regulations should be adjusted accordingly. Therefore, maintaining continuous dialogue between scientists, industry, and policymakers is essential to ensure that regulations evolve alongside technological advancements. Overall, policies should accelerate the transition to sustainable materials by incentivizing innovation, building supporting infrastructure (such as composting and recycling facilities), and restricting polluting plastics through taxes or bans. By implementing these measures, governments can create a market landscape where LCFs become viable, scalable, and environmentally beneficial alternatives to traditional plastics.

#### 7.5.4 Interdisciplinary Research and Collaboration

LCFs involve complex challenges spanning materials science, environmental science, and economics, making it difficult for a single discipline to address all issues independently. Therefore, future development requires interdisciplinary collaboration. Chemists, chemical engineers, and materials scientists must work together to develop new processing techniques and film formulations. Meanwhile, environmental scientists and industrial ecologists play a key role in ensuring these materials truly reduce ecological impact by studying their degradation mechanisms in marine and composting environments. Engineers and economists can use techno-economic modeling to guide scalable and cost-effective innovations. Biotechnology will also play an important role. For example, genetic engineering can be used to cultivate plants with higher cellulose yields or to design specific enzymes that efficiently break down lignin. Recent studies suggest that genetic editing can optimize biomass utilization and even improve the production and recyclability of biobased polymers. These advances highlight how biotechnology can enhance both the raw material supply and the degradation properties of LCFs. Collaboration with industry is equally critical. Companies can test prototype films in real-world packaging production lines to identify practical challenges and provide feedback for targeted improvements. Additionally, social scientists and policy experts can help address market acceptance issues and regulatory barriers, ensuring that policies support rather than hinder sustainable material adoption. Overall, bringing LCFs from the lab to large-scale application will require integrating multiple disciplines, combining scientific advancements with industrial innovations and policy development to create viable, scalable, and sustainable solutions.

### 7.6 Conclusion

While LCFs still face challenges in large-scale production, performance, and commercialization, ongoing research and innovation are actively addressing these issues. In the coming years, advancements in manufacturing techniques and material properties, along with progress in chemistry, engineering, and biotechnology, are expected to drive LCFs toward becoming a mainstream sustainable material. Furthermore, integrating sustainability strategies with forward-thinking policies can facilitate the transition of these materials from concept to real-world application, helping to reduce dependence on conventional plastics. Although further efforts are needed to lower costs and overcome technical barriers, LCFs emerge as one of the most promising directions in sustainable materials science.

## ▲

Department of Forestry, Michigan State University, East Lansing, MI 48824, USA

## Abbreviations

AA: acrylic acid
ACC: all-cellulose composite
ACNs: anthocyanins
AcOH: acetic acid
AgNPs: silver nanoparticles
AgNWs: silver nanowires
AKD: alkylketene dimer
AL: alkali lignin
ALD: atomic layer deposition
AMIM: 1-allyl-3-methylimidazolium
AMP: antimicrobial peptides
APS: ammonium persulfate
APTES: 3-aminopropyltriethoxysilane
ASLS: alkyl chain sulfobutylated lignosulfonate
ASSC: all-solid-state supercapacitor
ATRP: atom-transfer polymerization
AuNPs: Au nanoparticles
AVF: alternating vacuum filtration
AZIBs: aqueous zinc-ion batteries
BCNF: bacterial cellulose nanofiber
BCP: bromocresol purple
BDMs: soil-biodegradable plastic mulch
BMIM: 1-butyl-3-methylimidazolium
BMIMCl: 1-butyl-3-methylimidazolium chloride
BNC: bacterial nanocellulose
BSA: bovine serum albumin
BW: beeswax
CA: cellulose acetate
CAB: cellulose acetate butyrate
CAN: cerium ammonium nitrate
CAP: cellulose acetate phthalate
CB: carbon black
CCNF: cross-linked cellulose nanofiber
CD: carbon dots
CE: coulombic efficiency
Cel: cellulose
Cel-Ag: cellulose-silver composite films
CFs: carbon fibers
CM: cellulose membrane
CMC: carboxymethyl cellulose
CMCS: carboxymethyl chitosan
CMNF: cellulose micro- and nanofiber
CN: cellulose nitrate
CNCs: cellulose nanocrystals
CNFs: cellulose nanofibrils
CNP: cellulose nanopaper
CNS: cellulose nanosheets
CNTs: carbon nanotubes
CNW: cellulose nanowhiskers
CO_2_e: CO_2_-equivalent
CP: cellulose phosphate
C-PPy-NT: carbonized polypyrrole nanotubes
CQDs: carbon quantum dots
CRP: C-reactive protein
CS: chitosan
CTE: coefficient of thermal expansion
CuNWs: copper nanowires
CVD: chemical vapor deposition
CWNF: cationic wood nanofiber
CWNF: lignin-containing cationic wood nanofiber
DA: dimethyl adipate
DAC: dialdehyde cellulose
DACNFs: dialdehyde cellulose nanofibers
DBU: 1,8-diazabicyclo­[5.4.0]­undec-7-ene
DCA-PMDA: pyromellitic dianhydride-grafted deacetylated cellulose acetate
DCC: dicarboxy cellulose
DCPI: 3,4-dichlorophenyl isocyanate
DDSA: dodecenyl succinic anhydride
DES: deep eutectic solvent
DMF: *N*,*N*-dimethylformamide
DP: degree of polymerization
DPPH: 2,2-diphenyl-1-picrylhydrazyl
DS: degree of substitution
DTZ: dithizone
EC: ethyl cellulose
ECH: epichlorohydrin
e-CNC: esterified cellulose nanocrystals
ECO: electrochemical osmotic
EMI: electromagnetic interference
EMIMCl: 1-ethyl-3-methylimidazolium chloride
EPR: extended producer responsibility
E-skin: electronic skin
ESO: epoxidized polymeric soybean oil
FEP: fluorinated ethylene propylene
g-C: grafted-cellulose
GCD: glucose-derived carbon dots
GLU: glucose
GM: galactomannan
GNS: graphene nanosheet
GNs: graphene nanosheets
GO: graphene oxide
Gox: glucose oxidase
GP: graphite powder
GQDs: graphene quantum dots
GWP: global warming potential
HAP: hydroxyapatite
HC: hexanoyl chloride
HCNFs: holocellulose nanofibrils
HEC: hydroxyethyl cellulose
HMDSO: hexamethyldisiloxane
HNS: heterofibrous network scaffold
HPAMAM: hyperbranched polyamide-amine
HPC: hydroxypropyl cellulose
HPMC: hydroxypropylmethyl cellulose
HPMCP: hydroxypropylmethyl cellulose phthalate
HRC: hydrophobic regenerated cellulose
ICP: intrinsically conducting polymers
ILs: ionic liquids
IMP: intercalation-modulated plasticization
IOAC: inverse opal acetylcellulose
IPA: isopropyl alcohol
KGM: konjac glucomannan
KPS: potassium persulfate
LbL: layer-by-layer
LCA: life cycle assessment
LCC: lignin–carbohydrate complex
LC–CNC: liquid crystalline-cellulose nanocrystal
LCFs: lignocellulosic films
LCNF: lignin-containing cellulose nanofibers
LCO: LiCoO_2_
LiCl/DMAc: lithium chloride/*N*,*N*-dimethylacetamide
LiPS: Li polysulfide
LiTFSI: lithium bis­(trifluoromethanesulfonyl)­imide
LMBs: lithium metal batteries
LNM: lignin nanomicelle
LNPs: lignin nanoparticles
LP: lignin-poly­(ethylene glycol)
LPF: lignin-based polyurethane film
LRV: log reduction value
LSBs: lithium–sulfur batteries
LS/PPy: lignin/polypyrrole
MABs: metal–air batteries
MB: methylene blue
MC: methyl cellulose
MCC: microcrystalline cellulose
MD: molecular dynamics
MFC: microfibrillated cellulose
MH: monolithic heteronanomat
MOFs: metal–organic frameworks
MPD: *M*-phenylenediamine
MPTMS: 3-mercaptopropyltrimethoxysilane
MR: methyl red
MSCs: microsupercapacitors
MSH: molten salt hydrate
MSP: minimum selling price
MTS: methyltrichlorosilane
MW: molecular weight
MWCNTs: multiwalled carbon nanotubes
NCC: nanocrystalline cellulose
NFC: nanofibrillated cellulose
NC-PVL: nitrocellulose passivation layer
NGs: nanogenerators
NGCD: nitrogen-functionalized carbon dots
NIPS: nonsolvent-induced phase separation
NMMO: *N*-methylmorpholine-*N*-oxide
NMP: nitroxide-mediated polymerization
NP: nisin peptide
NPL: anionic phenolated lignin
NPs: nanoparticles
NWs: nanowires
OFET: organic field-effect transistors
OLEDs: organic light-emitting diodes
OSA: *N*-octenyl succinic anhydride
OTR: oxygen transmission rate
OTS: octadecyltrichlorosilane
PAA: poly­(acrylic acid)
PAE: polyamide-epichlorohydrin resin
PAN: polyacrylonitrile
PANI: polyaniline
PBAT: poly­(butylene adipate-*co*-terephthalate)
PBS: polybutylene succinate
PCL: polycaprolactone
PDA: polydiacetylene
PDMAEMA: poly­(*N*,*N*-dimethylaminoethyl methacrylate)
PDMS: polydimethylsiloxane
PE: polyethylene
PECVD: plasma-enhanced chemical vapor deposition
PEDOT: poly­(3,4- ethylenedioxythiophene)
PEG: poly­(ethylene glycol)
PENGs: piezoelectric nanogenerators
PEO: poly­(ethylene oxide)
PET: polyethylene terephthalate
PHA: polyhydroxyalkanoates
PHBV: poly­(3-hydroxybutyrate-*co*-3-hydroxyvalerate)
PI: polyisoprene
PIP: piperazine
ε-PL: ε-polylysine
PLA: poly­(lactic acid)
PMMA: poly­(methyl methacrylate)
PMMA-*b*-PTFEMA: poly­(methyl methacrylate)-*block*-poly­(trifluoroethyl methacrylate)
PMMAZO: poly­(6-[4-(4-methoxyphenylazo) phenoxy] hexyl methacrylate)
PP: polypropylene
PPy: polypyrrole
POSS-NH_2_: aminopropyl polyhedral oligomeric silsesquioxane
PSS: polystyrenesulfonate
PTFE: poly­(tetrafluoroethylene)
PVA: poly­(vinyl alcohol)
PVDF: polyvinylidene fluoride
PVDF-*g*-CMs: poly­(vinylidene fluoride)-grafted cellulose membranes
PVP: polyvinylpyrrolidone
RAFT: reversible addition–fragmentation chain-transfer polymerization
RC: regenerated cellulose
R-CD: resazurin modified carbon dots
RCF: regenerated cellulose films
RCS: regenerated cellulose separator
RO: reverse osmosis
rGO: reduced graphene oxide
RH: relative humidity
ROP: ring-opening
SA: sodium alginate
SCs: supercapacitors
SEA: separator/electrode assembly
SEI: solid electrolyte interphase
SL: sodium lignosulfonate
SPEs: solid polymer electrolytes
SSC: symmetric supercapacitor
SWCNTs: single-walled carbon nanotubes
TBD: triazabicyclodecene
TDI: toluene diisocyanate
TE: thermoelectric
TEA: techno-economic analysis
TEMACl: triethylmethylammonium chloride
TENGs: triboelectric nanogenerators
TFAA: trifluoroacetic anhydride
TFNC: thin-film nanofibrous composite
TFT: thin-film transistors
THz: terahertz
TMC: trimesoyl chloride
TMPTMA: trimethylolpropane trimethacrylate
TOC: TEMPO oxidized cellulose
TOCNF: TEMPO-oxidized cellulose nanofibrils
TOF-SIMS: time-of-flight secondary ion mass spectrometry
TPS: thermoplastic starch
TSPOSS: trisilanolisobutyl-polyhedral oligomeric silsesquioxane
TSPs: touchscreen panels
TPY: terpyridine
TWF: transparent wood film
UV: ultraviolet
VBB: Victoria Blue B
WCA: water contact angle
WLF: Williams–Landel–Ferry
WVP: water vapor permeability
WVTR: water vapor transmission rate
ZIBs: Zn-ion batteries
